# The Homeobox Genes of *Caenorhabditis elegans* and Insights into Their Spatio-Temporal Expression Dynamics during Embryogenesis

**DOI:** 10.1371/journal.pone.0126947

**Published:** 2015-05-29

**Authors:** Jürgen Hench, Johan Henriksson, Akram M. Abou-Zied, Martin Lüppert, Johan Dethlefsen, Krishanu Mukherjee, Yong Guang Tong, Lois Tang, Umesh Gangishetti, David L. Baillie, Thomas R. Bürglin

**Affiliations:** 1 Dept. of Biosciences and Nutrition & Center for Biosciences, Karolinska Institutet, Hälsovägen 7, Novum, SE-141 83, Huddinge, Sweden; 2 School of Life Sciences, Södertörns Högskola, Huddinge, Sweden; 3 Dept. of Molecular Biology and Biochemistry, Simon Fraser University, Burnaby, British Columbia, V5A 1S6, Canada; Foundation for Research and Technology-Hellas, GREECE

## Abstract

Homeobox genes play crucial roles for the development of multicellular eukaryotes. We have generated a revised list of all homeobox genes for *Caenorhabditis elegans* and provide a nomenclature for the previously unnamed ones. We show that, out of 103 homeobox genes, 70 are co-orthologous to human homeobox genes. 14 are highly divergent, lacking an obvious ortholog even in other *Caenorhabditis* species. One of these homeobox genes encodes 12 homeodomains, while three other highly divergent homeobox genes encode a novel type of double homeodomain, termed HOCHOB. To understand how transcription factors regulate cell fate during development, precise spatio-temporal expression data need to be obtained. Using a new imaging framework that we developed, Endrov, we have generated spatio-temporal expression profiles during embryogenesis of over 60 homeobox genes, as well as a number of other developmental control genes using GFP reporters. We used dynamic feedback during recording to automatically adjust the camera exposure time in order to increase the dynamic range beyond the limitations of the camera. We have applied the new framework to examine homeobox gene expression patterns and provide an analysis of these patterns. The methods we developed to analyze and quantify expression data are not only suitable for *C*. *elegans*, but can be applied to other model systems or even to tissue culture systems.

## Introduction

During embryogenesis, cells divide and their fates become successively more restricted to give rise to different cell types and tissues. Transcription factors play crucial roles in this process by selectively activating specific target genes only in the correct cell types. Homeodomain (HD) proteins are a class of transcription factors that are intimately involved in developmental decisions both in animals and plants (e.g., [1, 2]). Thus, understanding their regulation and function will provide important insights into the cell fate decisions in which they partake. With the completion of the genome sequence of the nematode *Caenorhabditis elegans*, compilations of the complement of homeobox genes in *C*. *elegans* have become available [3]. A previous list identified 99 homeobox genes [4]. Here we provide an updated list of the homeobox genes, provide a completed nomenclature, and assign them to their human orthologs.

*C*. *elegans* is a widely used model system for understanding metazoan biology (e.g., [5]). Due to its invariant cell lineage [6, 7], fast development, small cell number, and transparency, it is an ideal system for *in vivo* observation of embryonic and post-embryonic development, where events can be studied at the single cell level. Cell lineaging using differential interference contrast (DIC) microscopy has been successfully applied to gain many insights into the biology of *C*. *elegans* and other species (e.g., [8–13]). With the advent of green fluorescent protein, it has become feasible to monitor gene expression *in vivo* [14], and it has been applied to obtain time-lapse 3D recordings of gene expression [15, 16]. More recently, automated lineaging has become feasible using fluorescent-tagged histone as markers for tracing [17–19]. These facts, as well as the large number of available mutant alleles and transgenic reporter strains, make *C*. *elegans* well suited for systematic approaches towards unraveling developmental events at the cellular level.

**Fig 1:**
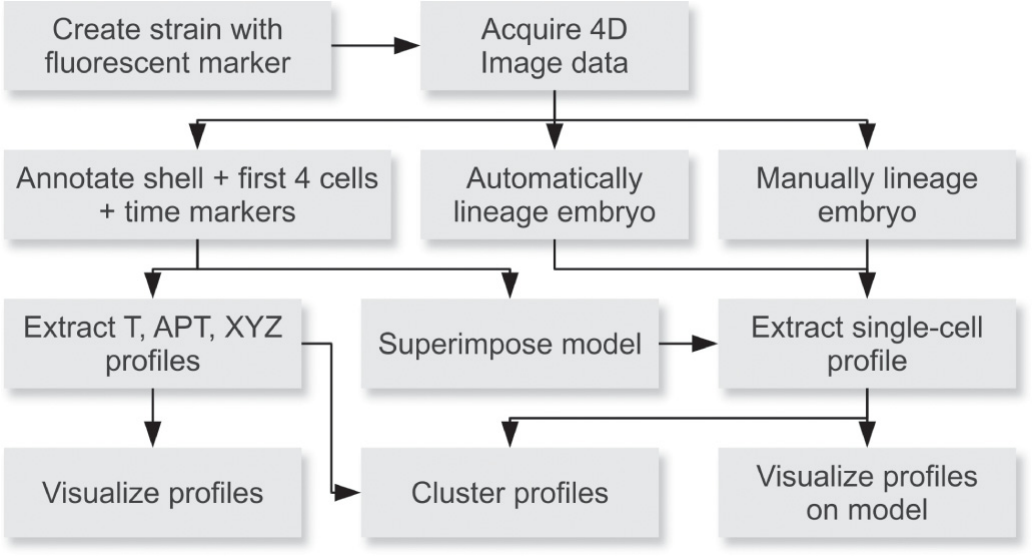
The 4D analysis workflow. Multiple strategies for profiling expression patterns have been implemented in Endrov. The most basic strategy extracts “fingerprint” profiles over anterior-posterior and time, ignoring cell coordinates. At a higher level, a reference model is superimposed after annotating the first four cells and several reference time points. The pipeline also allows manual lineaging.

Given our interest in understanding how homeobox genes regulate cell fates (e.g., [20–24]), we endeavored to develop a workflow that allowed us to examine *C*. *elegans* gene expression in a reproducible fashion during embryogenesis (Fig 1). A major issue with 4D recordings is sample viability, e.g., *C*. *elegans* embryos are sensitive to light exposure and die when overexposed (e.g., [11, 25]). No existing software provided the necessary flexibility to allow optimal parameter choices to reduce sample exposure with standard fluorescent microscopes. Further, we intended to create a more general microscopy framework that would be suitable to record images from a number of different microscopy platforms using DIC and standard fluorescent microscopy, which are widely available. This led us to develop an imaging framework, Endrov, which we use here to also examine the spatio-temporal expression of homeobox genes during embryogenesis [26]. We have already used an early version of Endrov to develop a new 4D model of *C*. *elegans* development [12]. A key difference to previous models was that we did not compress the embryo during recording, which changes the cell contacts, and, more importantly, the non-compressed embryos are more comparable to each other with respect to translation, rotation and scale. While DIC images provide morphological data, they are not well suited for automated lineage analysis. Of the algorithms we know, the best one for automatic tracking of cells using DIC images reaches only 24 cells [27]. Tracking using fluorescently labeled histone has proven much more feasible [18, 28, 29]. But in this case, double-labeled strains need to be used, and unwanted phenotypes may develop over time due to the histone marker [12]. Thus, having the possibility of obtaining spatio-temporal expression recordings with less invasive single GFP or RFP strains, especially also when monitored in mutant backgrounds or after RNAi treatment, is a useful complement that works with standard microscopes available in many laboratories.

Here, we have used our imaging workflow to examine expression patterns of homeobox during *C*. *elegans* embryogenesis. Many of them have already been analyzed using classical approaches (see S1 Text), but for many, no high-resolution spatio-temporal recordings have been done, and some of them have not been studied at all.

The purpose of this study was to provide a definitive list of homeobox genes for *C*. *elegans* and identify their human orthologs. Further, we used the microscopy imaging software, Endrov [26], that we developed to conduct a survey of the embryonic expression patterns of many of these genes with high spatio-temporal resolution.

## Materials and Methods

### Sequence analysis

Sequence analyses and protein logo creation with LogoBar were carried out as previously described. [1, 30–33]. To generate an updated list of homeobox genes in *C*. *elegans*, we conducted PSI-Blast searchers of the *C*. *elegans* protein sequences in Genbank. All sequences presented here were detected with this method. Furthermore, to detect also multiple HD sequences, we conducted a HMMER [34] search of all protein coding ORFs in WormBase release WS220; the profile was generated from the known HDs. The classification of homeobox genes was performed according to established procedures based on domain structure and HD phylogenetic analyses [2, 31, 35, 36]. For the phylogenetic analysis, a large sample size of metazoan sequences were used (Mukherjee et al., in preparation). Here we present a phylogenetic tree based on the *C*. *elegans* HD sequences, which recapitulates the general classification remarkably well. Since the chromosomal location of genes can provide additional clues, we developed a small Java utility to prepare the chromosomal location figures.

### Strains

Most transgenic *C*. *elegans* strains analyzed were created by PCR stitching the promoter sequences to GFP as described [25]. Other sources are: *ceh-1*::*GFP* [37], *ceh-2*::*GFP* [38], *ceh-10*::*GFP* [39], *ceh-13*::*GFP* [40], *ceh-14*::*GFP* [24, 41], *ceh-22*::*GFP* [42], *ceh-23*::*GFP* [43], *ceh-26*::*GFP* [44], *ceh-30*::*GFP* [45], *ceh-32*::*GFP* [21], *ceh-34*::*GFP* [46], *ceh-43*::*GFP* [47], *lim-4*::*GFP* [48], *mls-2*::*GFP* [49], *mec-3*::*GFP* [50], *unc-4*::*GFP* [51]. Sources for additional strains are provided in S1 Table. Non-integrated strains were integrated by gamma irradiation unless stated otherwise [52]. Lines that were confirmed to show homozygous transmission over 2–3 generations of the transgenic marker allele were considered suitable for recording and embryonic recordings were directly obtained. Selection of candidate lines was performed on NGM plates that were poured in multi-well plates (48 or 96 wells) that had been seeded with a drop of *E*. *coli* (OP50) bacterial broth. The cultures were semi-liquid and allowed for fast and efficient visual screening of the Dpy phenotype. Between 500 and 1000 animals were selected from the progeny of gamma-irradiated animals as a start, then approximately 20 progeny of a potentially heterozygous animal were singled onto new plates in seek of homozygotes. Homozygosity was confirmed by putting single progeny of a highly transmitting animal onto 5 cm NGM plates seeded with OP50. If the non-transgenic phenotype re-occurred even in a minority of animals, the line was not considered integrated.

Only well growing wild-type behaving lines were isolated and considered. A minimum of two independent lines from different irradiated P0s were isolated for each construct. Differences in the absolute expression level were expected and regularly occurred among unrelated lines that originated from the same extrachromosomal array.

Gamma irradiation causes double strand breaks and chromosomal rearrangements—an effect that is used for integration of extrachromosomal transgenes [53]. Crossing a line with a wild-type strain will remove unlinked damage, however this is unlikely to occur in the proximity of the transgene integration site. Closely linked mutations, or mutations at the integration site are nearly impossible to remove. Thus we decided against performing outcrossing and instead invested more time in obtaining integrated, stable and wild-type behaving lines. Our strategy aimed for selection against impairing phenotypes right after mutagenesis by only allowing healthy behaving animals to stay in the pool of candidates. If the reporter is expressed in the same way in two independent, wild-type behaving lines then we reasoned it is legitimate to consider the reporter expression as independent of the genetic background. This made further outcrossing after integration unnecessary for our purpose. While most analyzed strains were integrated, we also recorded some original non-integrated strains (annotated as BC strains).

### Microscopy

The microscope used is a Zeiss Axioplan 2, equipped with an Applied Scientific Instrumentation (ASI) ASI-S1630 piezo Z-stage, controlled by an ASI PZM-2000 controller. Images are acquired by an Hamamatsu ORCA ER (C4742-95-12ER) through an Active Silicon Snapper-DIG16 frame grabber installed in a PowerPC Macintosh computer running Mac OS X 10.4. Most images were acquired at 63x using a Zeiss 440762 oil-immersion objective and an Optivar attachment, usually set at 1.6x. For GFP a Zeiss filter set 38 HE or 09 was used. To reduce phototoxicity, particular with mercury light bulbs [11], we used either Halogen 100W lamps (HAL 100 light housing, Zeiss) or custom-made LED light sources, which were placed in the fluorescent light path, as well as the transmitted light path. Since LEDs are monochromatic, chromatic distortions through the optics should also be reduced. For DIC, we used 4 green LEDs (LXHL-MW1D). Two of the green LEDs are connected in parallel, with a 0.5W, 50Ω resistor in series. The LEDs, assembled in a LXHL-BM01 holder, were connected to the 0–12V adjustable voltage regulator of the microscope. For GFP, we used a blue LED (LXHL-MB1C) assembled on a CPU heat-sink for cooling. This LED was controlled by a C-Control Main Unit 1 station (http://www.c-control.de), programmed to accept serial commands sent from the computer. Whenever we recorded RFP, we used the Zeiss halogen light source both for GFP and RFP. The acquisition software was OpenLab (Improvision, now PerkinElmer).

For the initial recordings, an OpenLab Automator script was created. However, with long overnight recordings, we found that every so often, an error would cause the software to stall. Further, on-the-fly image analysis is not possible with Openlab. Subsequently, we used Openlab only to record a single stack at a time. The main control loop was implemented as an AppleScript that simulated user input, which passed on all the relevant parameters such as binning, slice number, slice spacing, exposure time, and light and filter configuration to Openlab. For automatic exposure control, the algorithm regulates exposure time by examining the signal intensity of the last acquired frame. The maximum intensity is a usable solution, but taking, e.g., the 10^th^ largest intensity instead protects against shot noise. Exposure should NOT be adjusted every frame as intensity is not entirely linear against exposure time, instead it should be changed when light goes above or below certain thresholds. When this happens, the new exposure time is the last exposure time multiplied or divided by a correcting factor. The thresholds and the correcting factor are provided by the user and can be adjusted for every recording. Typically, we allow the exposure time in the fluorescent channel to fluctuate between 200ms and 15ms.

Much effort was spent on reducing light exposure for viability, while capturing as much information as possible. This was achieved by increasing camera binning and reducing the number of Z slices and the stack sampling rate in the fluorescent channel. Further, halogen or LED light sources were used. Routinely, we acquired 70 DIC slices and 35 fluorescent slices. Time resolution is an important parameter for lineaging. Similar to Schnabel et al. 1997 [9] we found it sufficient to have 40 seconds between DIC stacks, and to acquire a fluorescent stack after every third DIC stack. It is possible to acquire fewer fluorescent stacks at the beginning, if no expression is seen early, as this appears to be the most light-sensitive period of embryogenesis.

The flexibility of the recording parameters (unlimited number of channels each with different parameters, i.e. binning, number of Z-slices, and temporal intervals) is a key feature of our imaging platform Endrov to obtain optimal sample acquisition. In addition, the on-the-fly adjustable exposure times allow a vastly increased dynamic range for capturing fluorescent signals that are not limited by the camera hardware. Endrov is open source software in Java available at www.endrov.net.

### Dynamic range extension of the signal by post-processing

Sensors in a digital camera count incident light on a quantized integer scale, e.g., 0–255 for an 8-bit camera. If a long exposure time is used to acquire a weak signal, often overexposure results later in development when the signal becomes strong. We have developed an algorithm that expands the effective sensitive range by dynamically adjusting the exposure time during the recording. Each new stack is analyzed during recording, and when the signal is becoming too bright or weak the exposure time is decreased or increased, respectively. The exposure time and other settings are stored in the metadata of the recording so that the overall intensity of the expression can be reconstructed later. In this fashion, we obtained about 10-fold increase in dynamic range [26].

Dynamic range expansion method: Each recording has been annotated with the embryo outline. The background signal is first subtracted for each frame. The background signal has to be estimated very conservatively to avoid artifacts, e.g., hatched worms that crawl by the embryo. The total average of the background is rather sensitive to such perturbations, unlike the median. However, the median does not change continuously over time. Instead, we take the average of the 40–60-percentile (call it the filtered average), since it changes more continuously with the background signal distribution over time and is insensitive to extreme outliers. We use the minimum value of the filtered average inside and outside the egg as the background signal; while normally the region outside the embryo represents the background sufficiently well, checking the embryo area also avoids some rare cases with negative values. The signal is almost linear to the exposure time but occasional discontinuities can be avoided by demanding that the average signal is the same between two frames at those time points when the exposure changes. It is important to note that the exposure time is not changed every frame during acquisition, but only when the signal is moving out of the sensitive range. We have also tried to fit the signal over the entire embryo from the last frame to the next frame by means of a linear model. This produces very smooth expression patterns but it has a severe problem: the signal intensity of the expression pattern converges to 0 over time. The reason is that linear least squares has a systematic bias towards zero, of a proportion that is related to the level of noise (see also regression towards the mean [54]).

### Annotation and normalization of recordings

The first four cells were manually annotated. Further, the location and time of the gastrulation, ventral enclosure, and the 2-fold stage were marked. To make annotation more convenient in 3D space, we have expanded the manual annotation with a novel feature that allows annotation in 3D rendered volumes [26]. To normalize time between recordings, the time of the recording was mapped to the time of the model by means of piecewise linear interpolation and extrapolation. For single-cell annotation, this is done on the level of each cell. Otherwise the following annotated time points from 0 to 100 were used instead. 0: ABa (or EMS), 10: Gastrulation “gast”, 43: Ventral enclosure “venc” and 54: 2-fold tail “2ftail”. The mapping ends when the normalized time reaches 100 or the recording ends.

### Comparison and clustering of recordings

Based on the normalized data, we evaluated both how to best summarize (reduce) the data, and how to compare the recordings based on the reductions. In addition to the **T**, **APT**, **XYZ**, and **SC** summary methods, we also explored Dorsal-Ventral-Time (**DVT**) and Left-Right-Time (**LRT**) profiles. The data for the latter two is presented in the Supplementary Material website, but were not further analyzed.

To compute pair-wise similarity, we attempted traditional methods, for example, using Pearson's colocalization coefficient, Manders' coefficient [55], or k-coefficients. These are normally used for samples with multiple labeling, but we assume that our normalization of the embryos allows comparison of the different samples and recordings. We also tried the Euclidian (l_2_)-distance. The raw comparison data are available online (see online data). Based on the pair-wise similarity, we performed clustering to visualize the results. We have qualitatively found that none of the algorithms we tried are strongly discriminatory. Neighbor-joining gave trees with long unlikely branches (data not shown). We also implemented our own algorithm of weighted spring-clustering [55], but the nodes did not separate well (data not shown). The PHYLIP Kitsch algorithm produces more balanced trees with good discrimination. To objectively assess the quality of the trees produced by the above algorithms we compared them quantitatively. The accuracy of the clustering can be assessed from the reporter constructs that have been recorded multiple times. Two recordings of the same construct normally end up next to each other, even for different strains, although not all recordings of the same reporter constructs do (see below, the dendrogram of recordings clustered based on APT profiles). A measure of quality is the distance between two recordings of the same type compared with the expected distance in a random tree. This has been calculated as shown in Table 1. Pearson turned out to be the best comparison metric.

**Table 1:** Clustering performance for different space partitionings and metrics.

	T		APT		XYZ		SC	
	l_2_	Pearson	l_2_	Pearson	l_2_	Pearson	l_2_	Pearson
Radius r	26	42	32	43	55	53	37	42
Average distance d	4.92	6.05	5.14	**4.4**	7.95	7.93	8.56	6.96
Quality q	0.17	0.22	0.15	**0.1**	0.23	0.2	0.34	0.26

To assess the quality of a tree, the distance μ between two recordings is the number of edges in-between. The shortest distance between two recordings of the same gene is thus 2. A well-balanced tree avoids long branches and should minimize the radius **r = max(⌠(v**_**i**_ **,v**_**j**_ **))**. The average closest distance between recordings of the same time is found to be **d = E[min(⌠(v**_**i**_ **,v**_**j**_ **))], v**_**i**_ **~ v**_**j**_. To find the expected distance in a random tree, the expression becomes only **D = E[min(⌠(v**_**i**_ **,v**_**j**_ **))]**. Both of these values were calculated by bootstrapping. Finally, to compare the quality of trees, the ratio **q = (d-2)/(D/2)** should be small. The best values are highlighted in bold.

### Comparison with microarray data

The microarray dataset GSE15234 for staged *C*. *elegans* embryos [56] was downloaded from NCBI GEO [57]. The dataset has multiple entries for each gene and the averages were used. The following time points were available: 4 cells, 28 cells, 55 cells, 95 cells and 190 cells. Each stage was compared to the time-only (**T**) expression summary of each gene, with time points taken from the mapped **SC** model. The significance was assessed by bootstrapping against random pairing of genes from our model versus the microarray. The code for loading the SOFT microarray file, comparing, and bootstrapping was written in Java.

### Calculations and Plots

Gnuplot (version 4.2) was used for plotting expression patterns (http://www.gnuplot.info/), except for **XYZ** summaries that were generated directly in Java. Expressions on the lineage and on the 3D model are shown with Endrov. Calculations and scripts were prototyped with Matlab (ver. 7.5.0.338, The Mathworks) and Octave (version 3.x, http://www.gnu.org/software/octave/). Final implementation is in Java 1.5 using Endrov as a library and host [26]. Endrov flows were used to prototype lineaging algorithms. The Debian Phylip package [58] was used for the clustering and the bootstrapping was implemented in Java. The tree was rendered with Njplot [59].

## Results

### The complement of C. elegans homeobox genes

**Fig 2:**
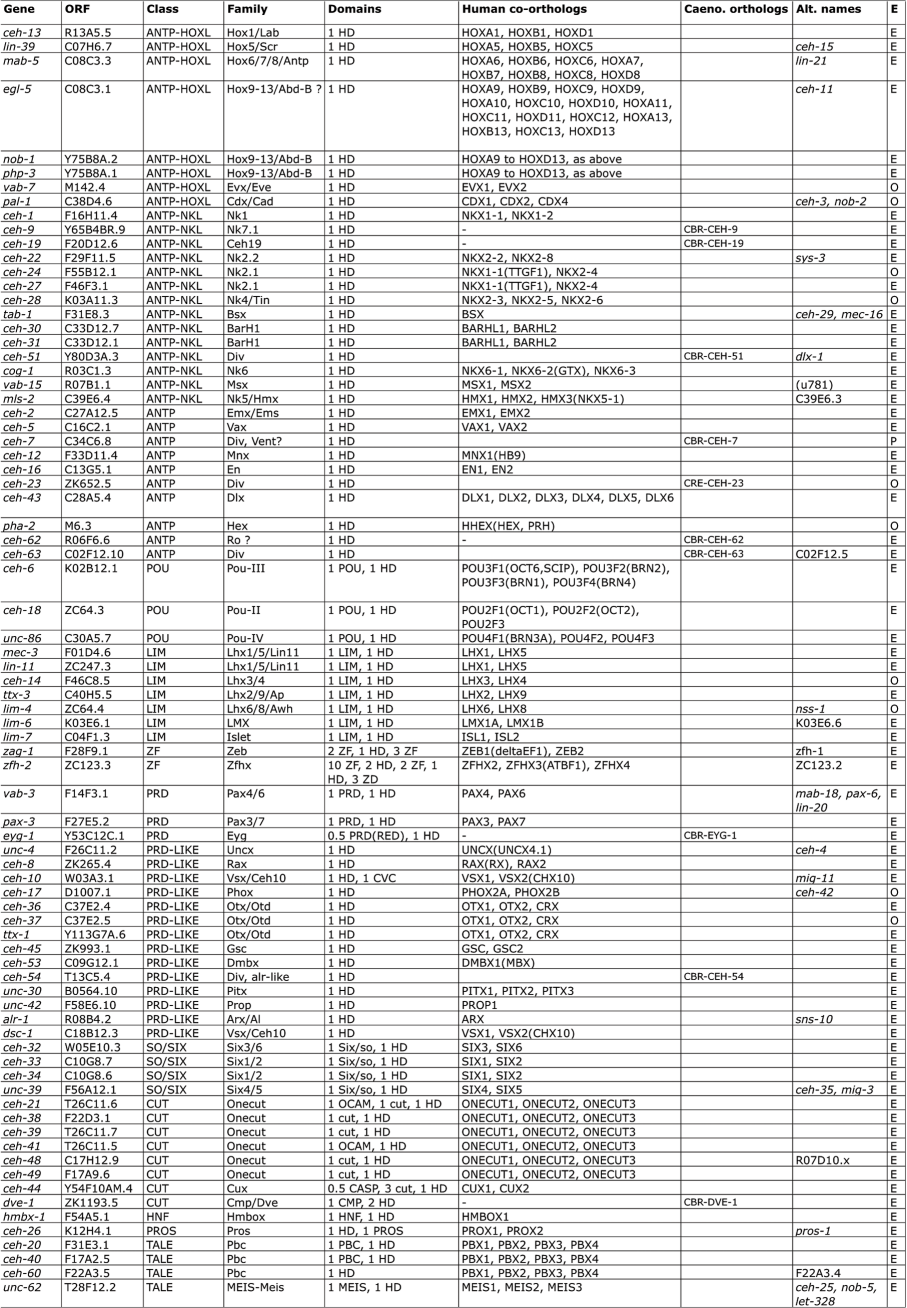
List of *C*. *elegans* homeobox genes and human orthologs. Gene names (gene) as well as WormBase sequence names (ORF) are given. At the bottom of the list under the “No HD” heading are genes related to homeobox genes that lack a HD. *psa-3* is a TALE homeobox gene with a MEIS domain that secondarily lost its HD. *egl-38*, *pax-1*, *pax-2* encode a Paired (PRD) domain only (*Pax* genes in vertebrates encode a PRD domain and may or may not encode a HD), and several *npax* genes encode only the first half of a PRD domain (PAI) [60]. *ocam-1* encodes an OCAM domain (Onecut associated motif) also found in some *C*. *elegans* Onecut genes [61]. The class column gives the class or superclass based on previous classifications [2, 31, 35, 36]. In the case of the Antennapedia (ANTP) superclass, the class division into NK-like (NKL) and HOX and related genes (HOXL) is indicated. ANTP genes that cannot be confidently assigned to one or the other family are simply designated as ANTP superclass genes. Family refers to the specific gene families that individual homeobox genes can be assigned to. A family is ideally conserved across the bilaterian divide. In some cases, it was possible to assign a class, but not a family. “Div.” indicates divergent genes that could not be classified confidently at the class or family level. The domain column lists the various domains found within the protein product of a gene as previously defined [2, 31, 35, 36]. The CVC domain is specific to the Vsx/Ceh10 family [62, 63]. The THAP domain is a zinc-binding motif [64], HOCHOB is defined here, and “UCM” is a presently uncharacterized motif with conserved cysteine residues (S4 Fig). Some smaller motifs (e.g., hexapeptide aka pentapeptide, octapeptide aka EH1 aka TN, etc.) are not indicated. Note that several proteins have multiple HDs, the number of each domain is given. In cases where a 0.5 is given, the domain is split, i.e. *eyg-1* encodes only the second half of the PRD domain (RED), and *ceh-44* incorporates the N-terminal half of CASP through alternative splicing [61]. The human co-orthologs column lists the human orthologs for the *C*. *elegans* genes. In many cases, there is no direct one-to-one correspondence, because of gene duplication in the vertebrate lineage, and in some instances also due to gene duplication within the nematode lineage. Hence, *vab-7* has two orthologs in humans, i.e. it is co-orthologous to EVX1 and EVX2. A number of homeobox genes lacked obvious human orthologs. In these cases, in order to examine the level of conservation of these divergent (Div.) homeobox genes, we conducted reciprocal blast searches against other *Caenorhabditis* species. In several instances we found matches in, e.g., *C*. *remanei*, *C*. *brenneri*, and *C*. *briggsae*. The “Caeno. orthologs” column lists selected orthologs that were found, indicating at least conservation to other Caenorhabditis species. Most importantly, a dash indicates that no ortholog was found in any other species, revealing fast evolving genes that must have arisen recently in the *C*. *elegans* lineage. The penultimate column lists alternative gene or ORF names. The last column (E) indicates whether a gene is transcribed based on transcript data. E indicates ESTs (WormBase). If no ESTs are present, OSTs (O), or Race (R) are taken as evidence for transcription. P indicates evidence based on RT-PCR [65].

**Fig 3:**
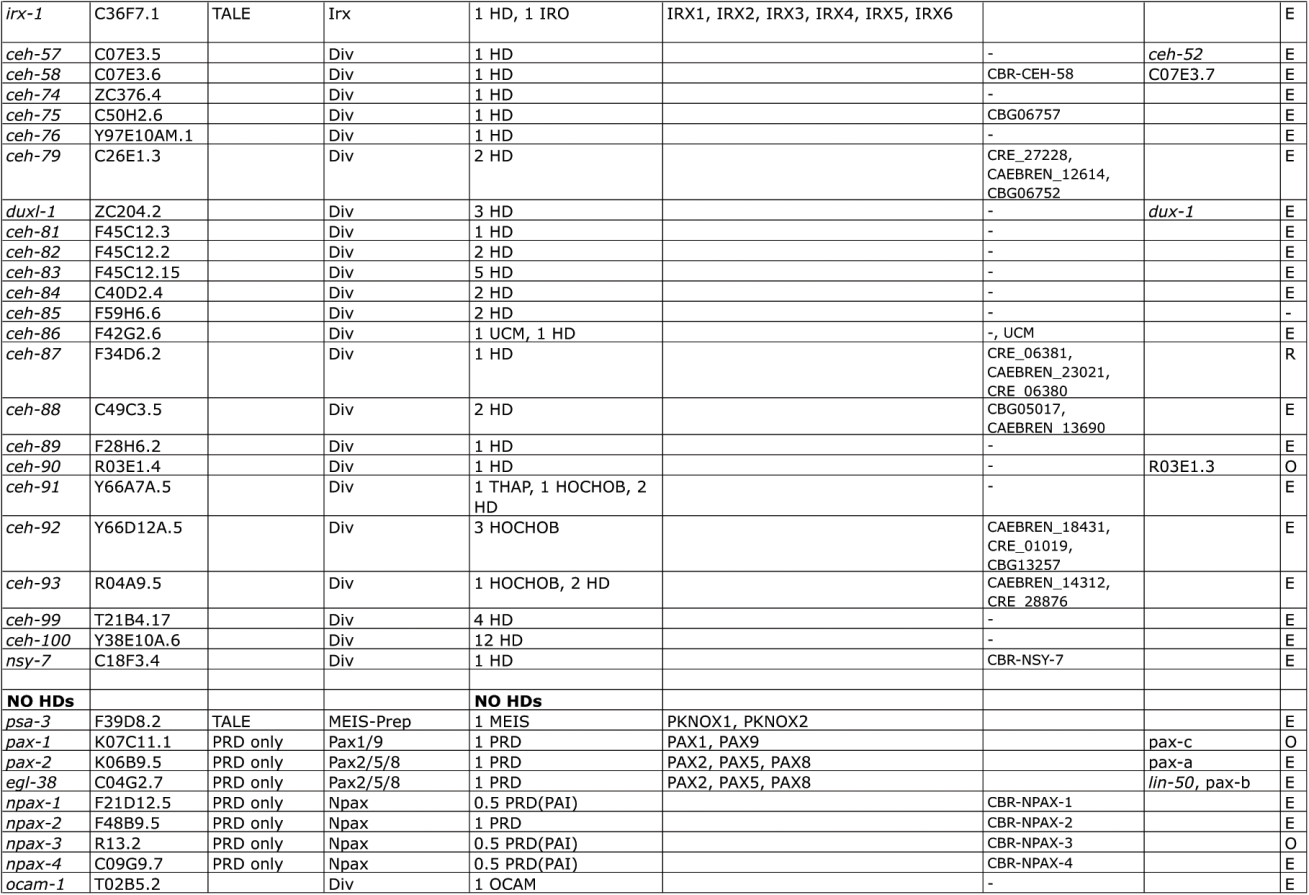
Second part of Fig 2.

**Fig 4:**
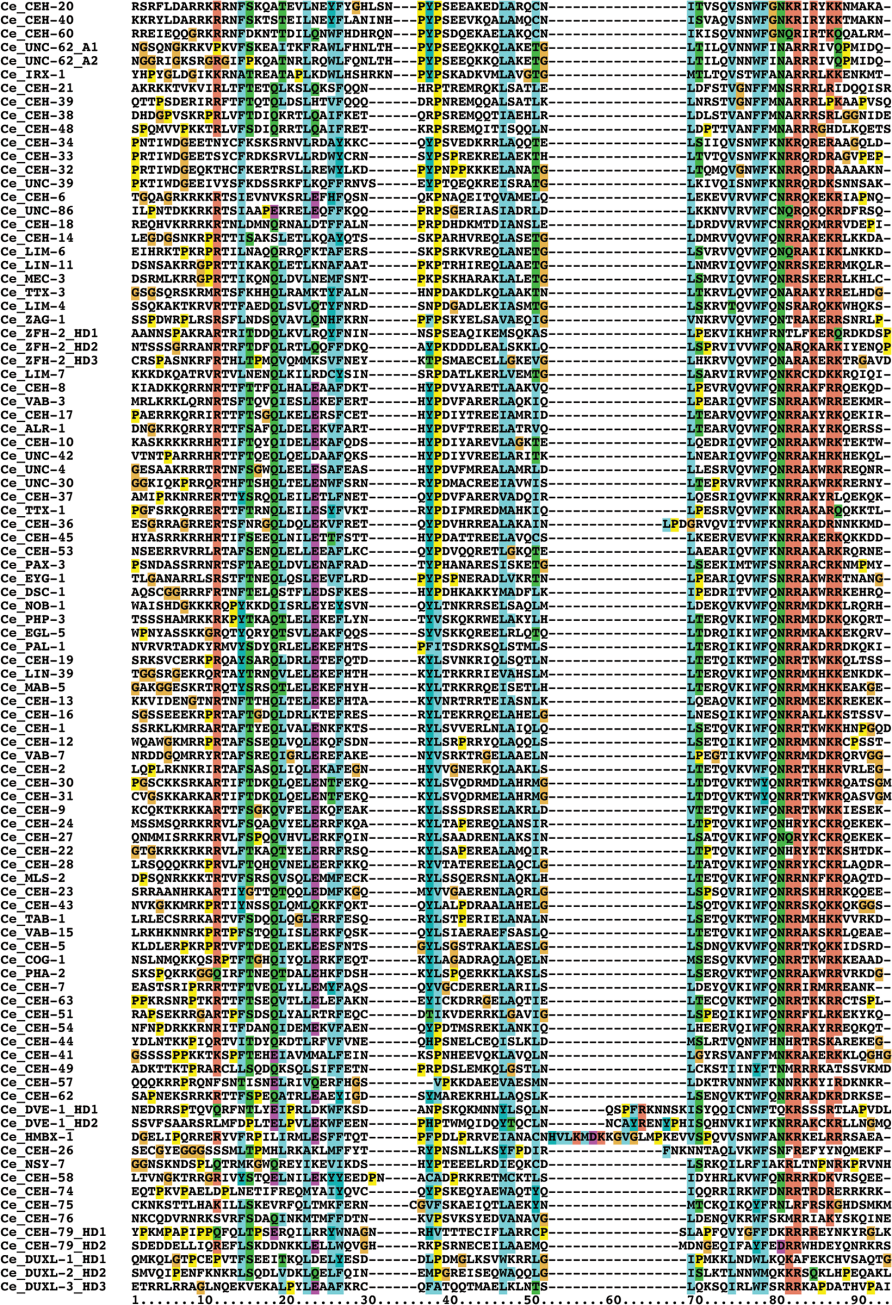
Multiple sequence alignment of *C*. *elegans* HDs. The standard numbering of a typical HD with 60 residues is given at the bottom, and the grey bars denote the extent of the three alpha helixes of the HD. Multiple HD within the same protein are denoted with HD1, HD2 etc. Note that a number of sequences have extra residues in loop 1 and/or loop 2 of the HD. UNC-62 has two different isoforms of the HD (suffixed as A1 and A2) due to alternative splicing [66, 67]. Unusually, three extra residues (ITV) in the HD of CEH-36 are inserted just upstream of the conserved WF (S1 Fig) through a shift in the location of a splice site. The three residues conform with residues expected at that position of the HD. Thus, it is likely that the N-terminal region of helix 3 is shifted so that the extra residues are effectively accommodated in the loop region between helix 2 and 3, as shown here, which allows the structure to be maintained. The currently predicted ORF of CEH-85 starts with the methionine residue in the middle of the HD1. Extending the ORF on the genome gives a good match to helix 1 of the HD, but presently no further upstream methionine or splice site can be found, hence the HD may only be partial (we thank John Spieth for the analysis). In a few of the proteins, some of the HDs are tightly packed with no space between the domains, and they can be as short as, e.g., 55 residues instead of the normal 60 in CEH-100_HD7. Overall we find 137 HDs plus 10 HOCHOB HDs (see below). Note that the first HDs of HOCHOB are not presented in this alignment, due to their lack of conservation of the WF motif. This alignment (except UNC-62_A2 and CEH-83_HD2) was used for creating a protein logo (see S1 Fig) and the phylogenetic tree (Fig 6).

**Fig 5:**
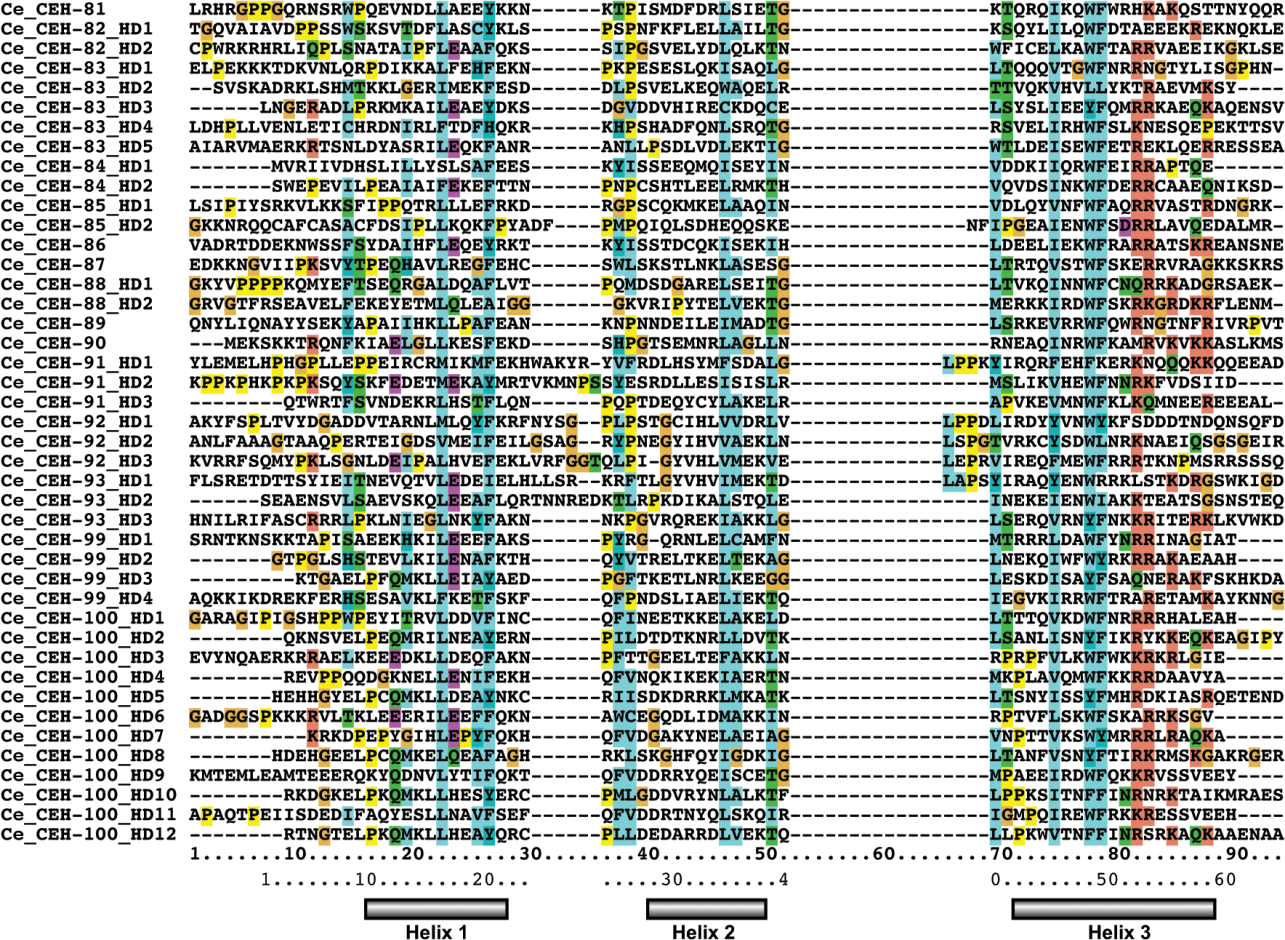
Second part of Fig 4.

In order to provide an updated list of homeobox genes we conducted TBLASTN searches of the *C*. *elegans* genome, which were subsequently complemented with PSI-BLAST searches. This was further verified by creating a HMMER profile that was used to search all ORFs of *C*. *elegans*. We identified 103 homeobox genes that conform to the HD profile (Figs 2–5, S1 Fig). Genes having only a sequence name until now were named using sequence classifications as criteria, if possible.

**Fig 6:**
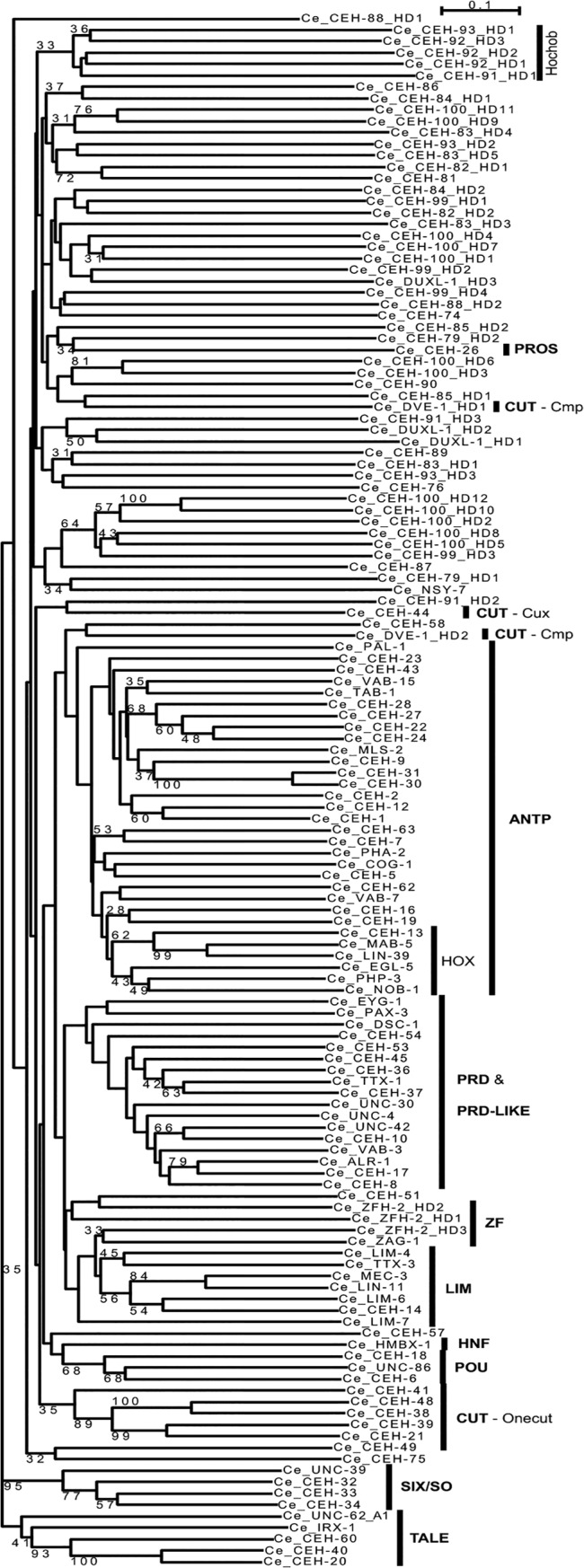
Phylogenetic tree of the HD sequences. Neighbor joining was carried out using the sequences from Figs 4 and 5. 100 bootstrap runs were carried out and bootstrap values larger than 30 are shown in the figure. The root was placed between the TALE HDs and the other HDs. The different classes/superclasses are indicated.

While most homeobox genes encode only a single HD (Figs 2–6), a number of exceptions are known (see e.g., [2]). In *C*. *elegans* we find two ZF (zinc finger) class homeobox genes, one with five HDs (*zag-1*) and one with three HDs (*zfh-2*, S2 Fig). Further a Cmp (Compass) family gene with two HDs (*dve-1*) is present. A number of homeobox genes encode multiple HDs that tend to be also rather divergent, i.e. *ceh-79* (2), *duxl-1* (3, two of which arose through an intragenic duplication), *ceh-82* (2), *ceh-83* (5), *ceh-84* (2), *ceh-85* (2), *ceh-88* (2). *ceh-99* has four HDs, while the related gene *ceh-100* has a record-setting 12 HDs that are tightly packed. None of these genes apart from *ceh-79* have obvious orthologs in other *Caenorhabditis* species. This lack of conservation suggests that these homeobox genes have mostly arisen *de novo* in the *C*. *elegans* lineage, and several of them are located on a duplication-rich chromosome arm (see below). Double homeobox genes have also been identified in mammals (DUX), but these seem to have originated in early mammalian evolution [68], and there is no evidence for direct orthology to *C*. *elegans* genes. We also identified a special subgroup of homeobox genes (*ceh-91*, *ceh-92*, *ceh-93*) that are so far specific to *Caenorhabditis* species and encode a novel double HD motif, which we term HOCHOB (see below).

Overall, there are 137 HDs plus 10 HDs in HOCHOB present in the *C*. *elegans* genome. Furthermore, there are nine HD-related proteins in *C*. *elegans*. Seven of them belong to the PRD domain group of proteins (often called PAX). Four of these have been named NPAX, because they only have the N-terminal PAI subdomain of the PRD domain (NPAX [60]). However, recent reexamination showed that the revised ORF of NPAX-2 does contain a divergent RED subdomain (Bürglin and Affolter, in preparation). PRD domain proteins merit being grouped together with HD proteins, since loss of the HD is secondary [69] (Bürglin and Affolter, in preparation). Loss of the HD is not unique. Two other genes seem to have lost their HDs relatively recently: *psa-3* is a Prep (TALE—MEIS class) family protein whose orthologs in other phyla have a highly conserved HD, and *ocam-1* has an OCAM motif otherwise found only in *ceh-21* and *ceh-41*. Using phylogenetic analyses (Fig 6) we classified the sequences into established categories [2, 35, 36]. In some cases it is clear that a gene belongs to the larger group of Antennapedia (ANTP) homeobox genes, but precise assignation to conserved families in other phyla is not (yet) possible, e.g., *ceh-23*, *ceh-63*.

We find that 70 (68%) *C*. *elegans* genes have recognizable orthologs in the human genome (Table 2, Figs 2 and 3). In most cases, a single *C*. *elegans* gene is orthologous to multiple human genes that duplicated during vertebrate evolution. Conversely, *C*. *elegans* has a number of paralogous genes that duplicated in nematode evolution, i.e. the families Abd-B, Pbc, Six1/2, Onecut, BarH1, Nk2.1, Lhx1/5/Lin11, and Otx/Otd. 23 genes are so divergent that they cannot reliably be assigned to existing classes in other phyla. While most genes have orthologs in *C*. *briggsae* or other *Caenorhabditis* species [37], 15 do not have obvious orthologs, indicating rapid evolutionary change. Many of these 15 divergent genes (*ceh-57*, *ceh-74*, *ceh-76*, *duxl-1*, *ceh-82*, *ceh-84*, *ceh-85*, *ceh-89*, *ceh-91*) have also been classified as *C*. *elegans* orphans by the *C*. *briggsae* genome project [70].

**Table 2:** Summary of different types of homeobox genes in *C*. *elegans*.

(Super)classes	Nr.	Human co-orthologs	Not conserved
ANTP	32	25	
PRD	3	2	
PRD-LIKE	14	13	
POU	3	3	
HNF	1	1	
LIM	7	7	
ZF	2	2	
SO/SIX	4	4	
CUT	8	7	
TALE	5 (+1^a^)	5 (+1^a^)	
PROS	1	1	
Div.	23	0	15
**Total hb genes**	**103**	**70**	
PRD domain only	7	3	

^a^ One TALE homeobox gene of the Prep family in *C*. *elegans* lost its HD, but retained its MEIS domain and is still orthologous to human genes.

The left column shows the classes or superclasses [2, 35, 36]. Class “Div.” are highly divergent genes that do not fall into existing classifications. The number (Nr.) of homeobox genes in each group is given, as well as the number of the genes that are co-orthologous to human homeobox genes. The right column shows the number of divergent homeobox genes that are not even conserved in other *Caenorhabditis* species. The bottom row lists genes with only a PRD domain.

### A novel double HD, the HOCHOB domain

**Fig 7:**
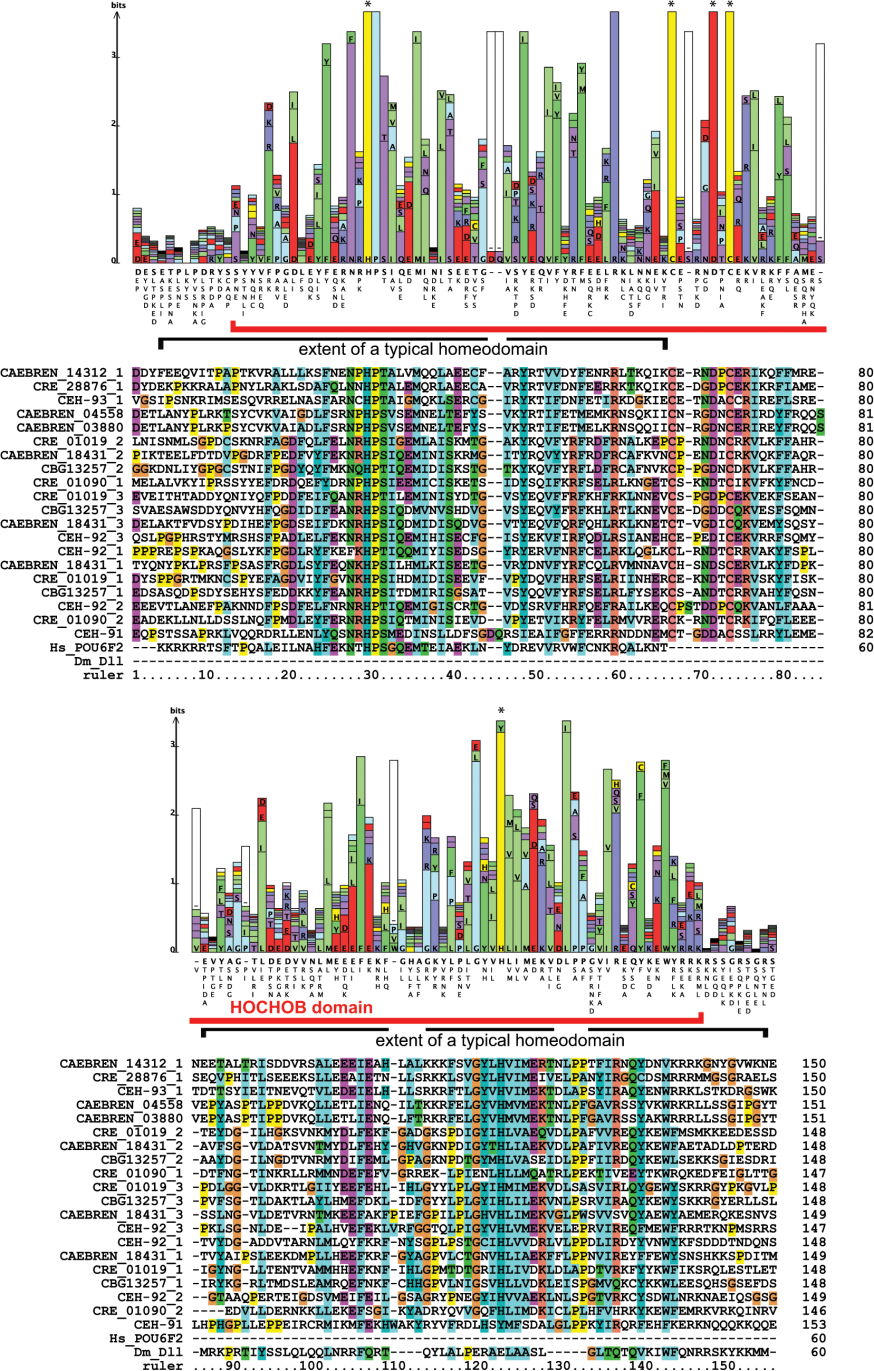
The HOCHOB domain. Multiple sequence alignment of *Caenorhabditis* HOCHOB domains. Multiple HOCHOB domains in the same protein are indexed with 1, 2, and 3. The matching protein logo above the alignment was generated using LogoBar. Stars denote highly conserved cysteine, histidine and aspartic acid residues. The red bar denotes the HOCHOB domain, and the extent of normal HDs is indicated underneath.

During the analysis of the divergent HD proteins, we identified two proteins, CEH-91 and CEH-93 that shared extended sequence similarity with each other upstream of their typical HDs (CEH-91_HD3 and CEH-93_HD3). Just upstream of these HDs each has a divergent HD (CEH-91_HD1, CEH-93_HD2), which has an insertion in loop 1 of the HD. Such insertions have also been observed in other HDs [2]. Additional sequence similarity extends further upstream, and PSI-blast searches with only this region retrieved the protein sequences shown in Fig 7. It includes CEH-92, which has three copies of this new motif, as well as several homologs found in *C*. *briggsae*, *C*. *remanei*, and *C*. *brenneri*. The new motif consists of two divergent HDs that are separated by a linker of about 17 residues (Fig 7). The linker has a number of conserved positions, two of which are cysteine residues. Hence, we term this motif HOCHOB (Homeobox—cysteine loop—homeobox). The second HOCHOB HD has extra residues inserted in loop 1 and loop 2 of the HD. The HD similarity of HOCHOB was initially detected by PSI-blast searches that detected the second HOCHOB HD. When the first HOCHOB HD of *C*. *brenneri* CAEBREN_14312 is used as query in a PSI-blast search, fungal HDs can be detected in the second iteration with P-values of < 0.001, supporting the notion that the first motif is also a divergent HD.

The key features of the HOCHOB HDs are shown in the protein logo in Fig 7. The pattern of conservation, in particular for the first HD sequence, is different from the normal HD profile, where conservation is highest in the third alpha helix (S1 Fig, [2, 71]). For this reason we did not include the first HD-like sequences of HOCHOB in the HD alignment of Figs 4 and 5. The fact that in particular helix 3 has changed substantially may mean that the DNA binding activity of the first HD may have been lost. It appears that the HOCHOB domain as a whole represents a functional unit, since it is duplicated as a unit in, for example, CEH-92. Further, these genes seem to be evolving fast, since no orthologs have been found yet outside the *Caenorhabditis* genus. The two absolutely conserved cysteine residues in the linker region between the two HDs suggest they could be involved in metal binding. However, additional residues would be required to form, for example, a zinc finger. There are two conserved histidine residues, one in each HD (in CEH-91 displaced by two positions), and there is also a conserved aspartic acid (marked with asterisks, Fig 7). Possibly two of these residues could contribute to zinc binding. We speculate that the HOCHOB domain is an evolutionary novelty that is derived from two HDs and may have gained metal-binding capacity.

In this context it is worth noting that *ceh-91* is predicted to encode a THAP domain at its amino-terminus, which has been shown to be a zinc-dependent C_2_CH DNA-binding domain [64]. Blastp searches using this N-terminus do not result in any matches in other nematodes, but do detect a few THAP domains in arthropods at not-significant levels. This suggests that either this domain has significantly diverged in CEH_91 and may be a novel acquisition, or that the sequence similarity is simply fortuitous.

### Chromosomal organization of homeobox genes

**Fig 8:**
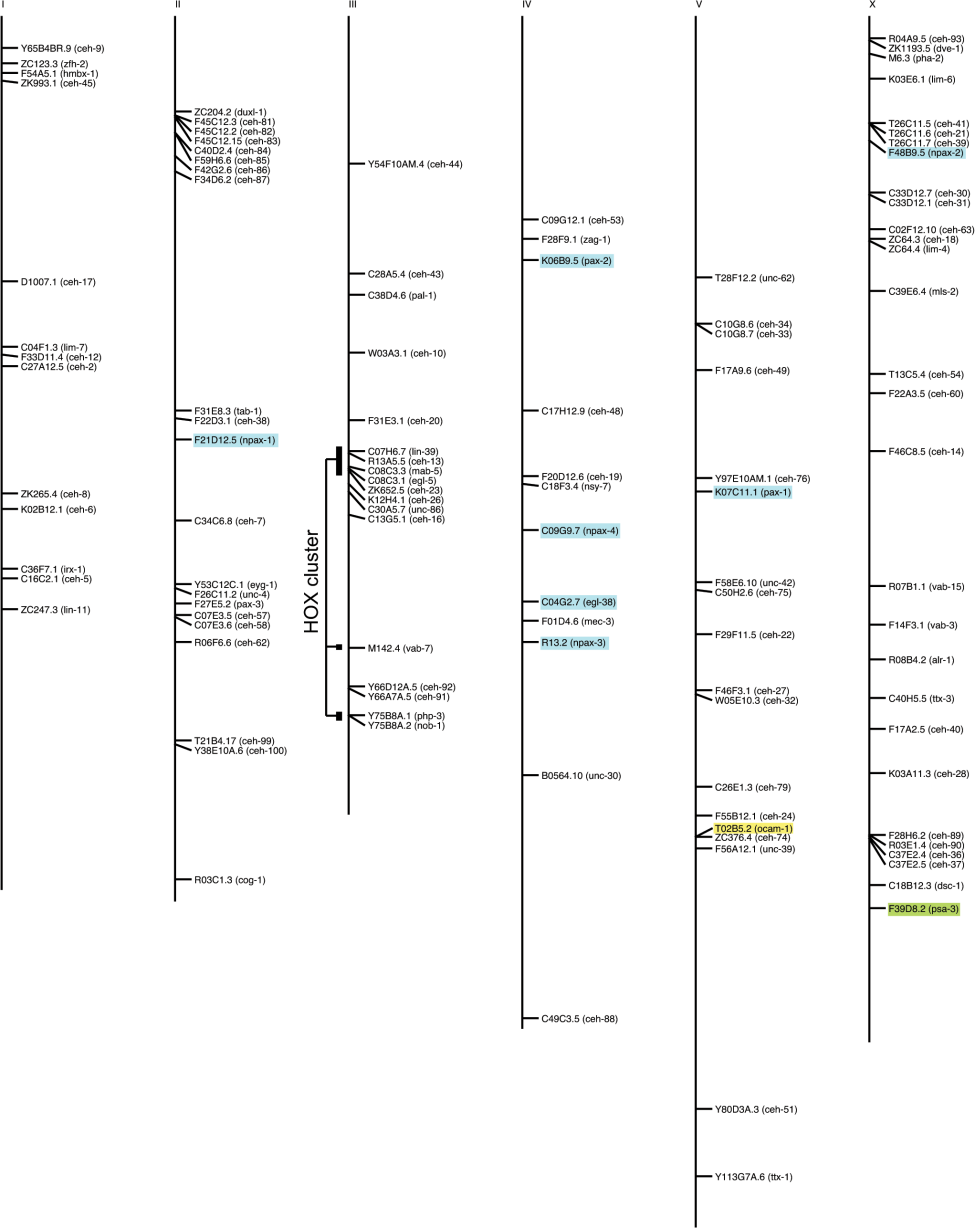
Chromosomal location of homeobox genes and related genes. The HOX cluster genes are indicated. PRD domain only encoding genes are marked in blue, the TALE gene *psa-3* that lost its homeobox is marked in green, and the *ocam-1* gene is marked in yellow. Clusters of homeobox genes are described in Table 3. Noteworthy are the grouped genes on the left arm of chromosome II, i.e., *ceh-81* to *ceh-87* and *duxl-1*. Most of these genes are all highly divergent, except *ceh-81* and *ceh-82*, which show similarity to each other. Many have multiple homeoboxes, and most do not have an ortholog in other *Caenorhabditis* species, except *ceh-87*.

We mapped the chromosomal location of the homeobox genes (Fig 8). No large-scale clusters are present. However, a number of genes are located next to each other, or are in close proximity (Table 3). Often such neighbors are closely related phylogenetically, indicating that they are indeed tandem duplicated genes. The HOX cluster (including Evx family genes) has been split into four fragments. The Evx split may already represent an old event, since also in arthropods *eve* is split from the HOX cluster [72]. Two Abd-B type genes (*nob-1*, *php-3*) have also separated far from the cluster, while the main HOX cluster is split into two parts (*ceh-13*, *lin-39*, and *mab-5*, *egl-5*, *ceh-23*).

**Table 3:** Homeobox gene clusters.

HOX cluster: (*lin-39, ceh-13*), (*mab-5, egl-5, ceh-23*), (*php-3, nob-1*), *vab-7*	The HOX cluster is located on chromosome III and has split into several parts in *C. elegans*. One cluster is formed by *lin-39* and *ceh-13*, which is separated by 250kb from the second cluster with *mab-5, egl-5* and the divergent homeobox gene *ceh-23*. A third cluster is about 4.3 Megabases away, formed by two Abd-B paralogs that duplicated within the nematode lineage, *php-3* and *nob-1*. In between lies *vab-7*, an Evx/Eve ortholog; Evx genes are part of the HOX cluster in vertebrates.
*ceh-91, ceh-92*	Two HOCHOB genes, separated by 5 ORFs (Figs 7 and 8).
*ceh-81, ceh-82, ceh-83*	Cluster of divergent homeobox genes, *ceh-81* and *ceh-82* are significantly similar to each other. See also S3 Fig.
*ceh-84, ceh-85*	*ceh-85* lies in the intron of math-32 in opposite orientation. *ceh-84* lies left of *math-19*, also in opposite orientation. The *ceh/math* genes are separated by one ORF. It suggests that *ceh-84* and *ceh-85* are duplicates, despite divergent sequence.
*ceh-57, ceh-58*	The genes lie next to each other. Although their HDs are very divergent, it is likely that *ceh-57*, which has no ortholog in other *Caenorhabditis* sp. is a highly diverged duplicate of *ceh-58*.
*ceh-99, ceh-100*	The two genes are separated by about 20 ORFs, but because some of their multiple HDs are similar to each other (Fig 8), they are recent duplicates.
*ceh-33, ceh-34*	Tandem duplication of Six1/2 homeobox genes.
*ceh-74, ocam-1*	*ceh-74* lies in the intron of a carboxylesterase gene, and is separated by two other carboxylesterase genes from *ocam-1*. Possibly *ocam-1* and *ceh-74* arose by a split from a single *ceh-41* like ancestor.
*ceh-21, ceh-39, ceh-41*	Cluster of Onecut homeobox genes [61].
*ceh-30, ceh-31*	Tandem duplication of BarH1 homeobox genes.
*ceh-89, ceh-90*	The two divergent genes are separated by a single gene (*akt-2*).
*ceh-36, ceh-37*	Tandem duplication of Otx/Otd homeobox genes.

Several homeobox genes, i.e. *duxl-1* and *ceh-81* to *ceh-86*, are located on the left arm of chromosome II (Fig 8, S3 Fig). These genes are mostly highly divergent, often encode multiple HDs, do not have orthologs in other *Caenorhabditis* species, and are embedded within other highly duplicated gene families (e.g., *fbxa*, *fbxb*, *fbxc*, *btb*, *math*). Thus, this region of chromosome II has been subject to rapid evolution with many duplication events, which probably also gave rise to these divergent homeobox genes. While CEH-86 does not have a direct ortholog with a HD protein in other *Caenorhabditis* species, it does share sequence similarity upstream of the HD with several uncharacterized ORFs that are clustered on cosmid C35E7 (S4 Fig). This region is conserved in ORFs of other *Caenorhabditis* species, and contains conserved cysteine residues. Presently, this uncharacterized cysteine motif (“UCM”) is not obviously related to known cysteine motifs. *ceh-86* might have arisen by a duplication event, where a homeobox translocated into a UCM family gene, or vice versa.

### Gene expression analysis

In order to examine the expression patterns of the homeobox genes during embryogenesis, primarily ones that have not been studied much, we took the GFP reporter constructs described by Hunt-Newbury et al. (2007) as starting point [25], and supplemented this with additional strains (see Materials and Methods, S1 Table). Additional strains were used to test our recording sensitivity and for other projects, e.g., *polg-1* [73]. The strains where subjected to 4D (spatio-temporal) microscopy; embryos were recorded over time by generating stacks of DIC and fluorescent images. A fundamental issue for continuous GFP recordings through *C*. *elegans* embryogenesis with a conventional fluorescent microscope is sample viability [11, 25]. We overcame this obstacle by using LED lights combined with judicious use of different parameters for DIC and fluorescent channels (see Materials and Methods, [12]). Further, we introduced a method to extend the dynamic range of the GFP signal intensity of the recordings to reduce overexposure when the GFP signal became strong at later times (see Materials and Methods, [26]). To manage this intricate recording scheme we developed the imaging framework Endrov [26]. The 4D stacks of DIC and GFP images can be viewed and played back in Endrov as original 4D image data. Further, we made summary movies for simple viewing. We have recorded 440 embryos in total, representing over 60 homeobox genes and over 85 genes in total (Table 4, see online movies). Most strains were recorded multiple times, and we observe very consistent results from these recordings. The best ones, which display good orientation and exposure times were selected for further quantification (see below). Using published examples, such as *pie-1*::*Histone*::*GFP* or *nmy-2*::*NYM-2*::*GFP*, we found that our system can detect early 1 to 4 cell expression (see online movies, [74, 75]).

**Table 4:** List of genes analyzed.

***ceh-1***	*ceh-54* (T13C5.4)*	clh-4*
***ceh-2***	***ceh-57* (C07E3.5)**	*die-1*
***ceh-5***	***ceh-74* (ZC376.4)**	*efn-4*
ceh-6*	***ceh-81* (F45C12.3)**	*egl-19*
***ceh-8***	***ceh-83* (F45C12.15)**	*hbl-1*
***ceh-10***	***ceh-84* (C40D2.4)**	his-24*
***ceh-12***	***ceh-85* (F59H6.6)**	his-72*
***ceh-13***	***ceh-87* (F34D6.2)**	*ifb-1*
***ceh-14***	***ceh-88* (C49C3.5)**	*ina-1*
***ceh-16***	***ceh-89* (F28H6.2)**	*kel-3*
ceh-19*	***ceh-93* (R04A9.5)**	*lat-1*
ceh-20*	***ceh-99* (T21B4.17)**	*lip-1*
***ceh-22***	***ceh-100* (Y38E10A.6)**	*mec-18*
***ceh-23***	***cog-1***	*mig-13*
***ceh-24***	***dsc-1***	*nmy-2*
***ceh-26***	***duxl-1* (ZC204.2)**	*nuo-1*
***ceh-27***	***eyg-1* (Y53C12C.1)**	*pie-1*
***ceh-28***	***lim-4***	polg-1*
ceh-30*	***lim-6***	*rgef-1*
***ceh-32***	***lim-7***	*tbx-2*
ceh-33*	***lin-11***	*unc-119*
ceh-34*	***mab-5***	*vab-1*
***ceh-36***	***mec-3***	xbx-1*
***ceh-37***	mls-2*	F55A4.3
***ceh-40***	***nob-1***	Y32H12A.8
ceh-41*	***ttx-1***	
***ceh-43***	ttx-3*	
ceh-44*	***unc-4***	
***ceh-45***	***vab-3***	
***ceh-48***	***zag-1***	
***ceh-49***	***zfh-2* (ZC123.3)**	
ceh-53*	***npax-3***	

Genes in bold are homeobox and Pax genes. Genes that have been given names are shown followed with the ORF in brackets. Some of the non-homeobox genes in the table were recorded to confirm our method with previously published data as well as for other interests. Asterisks indicate genes not analyzed with the global **T**, **APT**, **XYZ** methods.

### Methods for automatic extraction of expression patterns

While the ultimate goal of gene expression analysis in *C*. *elegans* is at the lineage level, many biological systems are not amenable to single-cell lineaging. Further, often one would like to perform global gene expression analysis and comparison of large datasets, e.g., clustering, which requires extraction of a suitable set of parameters from the images. As previously described, we have developed plug-ins for manual lineaging [12]. Here, we developed several methods for automated analysis. We investigated four different automated GFP signal extraction methods: Integrated signal intensity over the entire embryo over time (**T**); signal intensity in slices along the anterior-posterior (AP) body axis over time (**APT**); signal intensity of cubes that are aligned with the AP and left-right (LR)-axes (**XYZ**); Finally, we explored the possibility of superimposing the Ce2008 4D model [12] onto the recordings to identify the closest matching cells by approximation (**SC**). To apply these methods the recordings were normalized with respect to time. When mapping time from an annotated lineage, the life span of individual cells was used. For the other methods, several annotated time points based on the morphology of the embryo were used (see Materials and Methods). We have previously shown that uncompressed embryos are much less prone to rotation around the AP axis [12]. For the **APT** and **SC** analysis we had to make the assumption that uncompressed embryos do not rotate during development and stay fixed, which allowed us to define a coordinate system at the beginning of a recording. All extraction procedures used the shell annotation as the limiting area over which the expression signal is integrated. This total volume is used to represent the total signal of the embryo for method **T**. For **APT** the AP axis was defined as the major axis of the shell ellipsoid, and the embryo was divided into 20 slices along this axis. For **XYZ** we chose 20x20x20 voxels cubes to subdivide the embryo area. The center of the cubes is the center of ABa, ABp, EMS and P2. The vector EMS-ABp just prior to cell division was used in addition to create the left-right (LR) and dorsal-ventral (DV) axes. The total number of cubes arises from the distance between EMS-ABp and ABa, P2, enlarged by 35% to cover the embryo. For the **SC** method, in the absence of an annotated lineage, we superimposed the 4D model Ce2008 using the first four cells. In the few cases where the recording started later (up to eight cells), the coordinates of these cells were found by averaging the daughter cell coordinates. The cell geometry was approximated by Voronoi polyhedrons, as previously described [12]. It ensures that every pixel can be assigned to exactly one nucleus (the presumed closest one), but requires that all cells at that time point have been annotated in the model, otherwise signal from a missing cell will be assigned to neighboring cells.

### Comparison of expression pattern extraction methods

**Fig 9:**
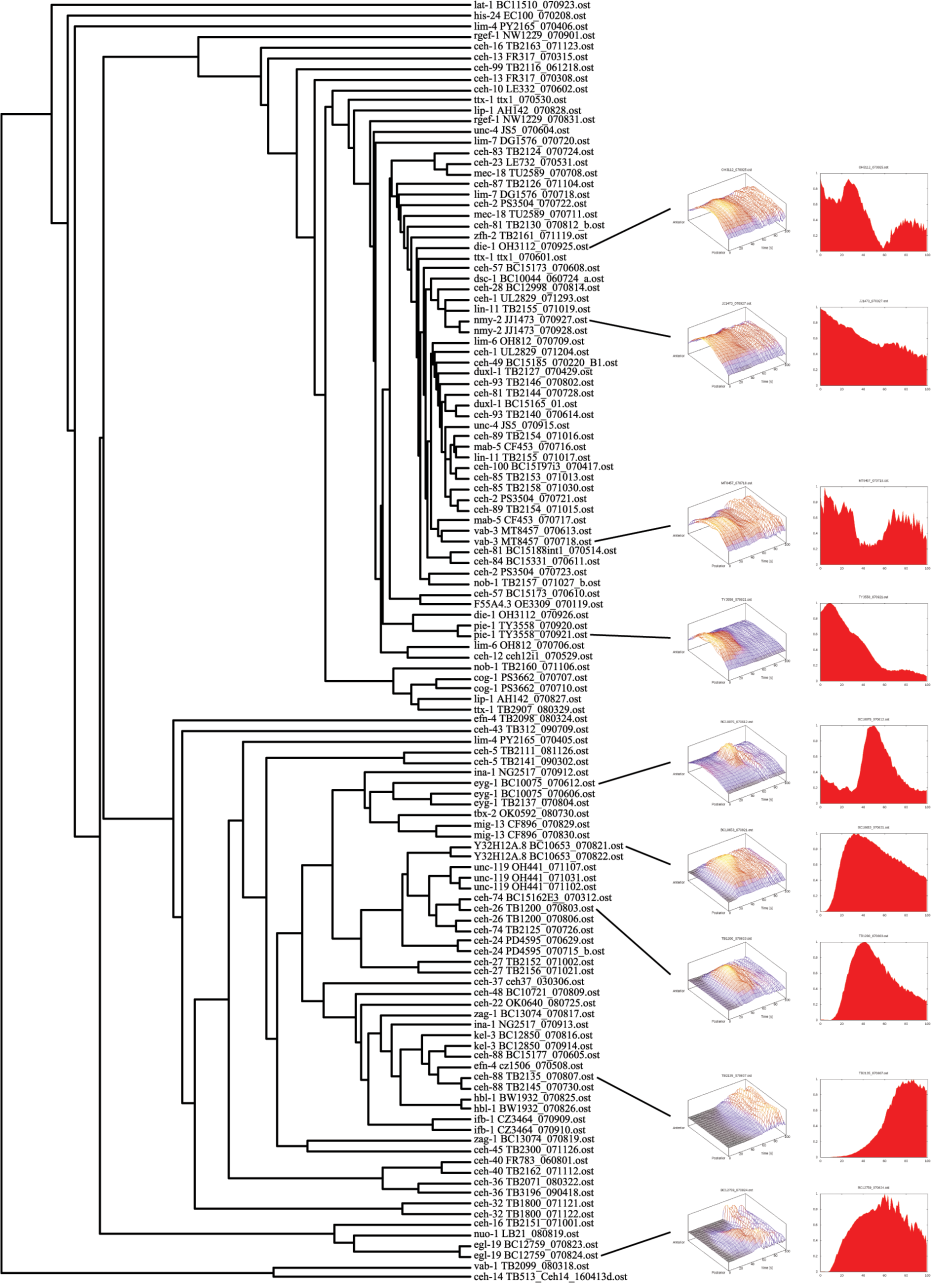
Dendrogram of recordings clustered based on APT profiles and Pearson correlation. Clustering based on Pearson correlation was carried out using 122 **APT** expression profiles. Leaves indicate the gene, strain, and recording. Example expression patterns as **APT** and **T** profiles are shown on the right. Recordings of the same or similar reporter constructs usually group together. The clades in the upper half of the tree with short branch lengths (approximately between the “ttx-1 TB2901_080329” and “ceh-10 LE332_070602” leaves) is comprised primarily of recordings that have no or late expression. The **APT** profiles of late expression patterns are subject to substantial variations, due to the moving embryo. This can even mask restricted expression patterns, since the location of the signal can change between individual Z-planes and is therefore subject to an averaging effect over the whole stack.

The global expression pattern extraction methods were assessed for their ability to discern different types of expression pattern in a reliable way. Several clustering methods were examined as described in Materials and Methods. In summary, **APT**, i.e. slicing along the AP axis over time is the best method, followed by the single-cell (**SC**) approximation. Adding more parameters (subdividing more) as in **XYZ** enables better discrimination of recordings of different genes, however at the cost of lower reproducibility. One way of representing cluster data is with a dendrogram. Using the **APT** data, a tree was generated from 122 selected recordings (Fig 9). Even though the **APT** profile has limited information, it is sufficient to cluster many of the duplicates of the same strain or related GFP reporter constructs. It shows not only that the 4D recordings themselves are reproducible, but also that the clustering method can identify similar expression patterns. When presumed duplicates do not closely cluster, it often could be associated with a problem with one of the recordings (data not shown). It is easy to rapidly scan **T** and **APT** profiles, for example, it is easy to see how the expression of *eyg-1* turns on before comma stage and later fades in late larval stages Fig 9). Thus, the tree can also be used as a global method to identify outliers or problems in the experiments.

A further important, but subjective aspect is also which of the four methods produces the best visual summary. Examples of **T** and **APT** profiles are included in Fig 9. **T** data is easy to view, but the information content is low and does not distinguish well between genes. **APT** is easy to view as a 2D heat map or a 3D graph. It is hard to visualize **XYZ** data in a way that captures both time and spatial information (see online data). **XYZ** does however give additional information about expression localization lacking in **APT**. However, the resolution-limits of the microscope in the Z-direction introduce errors in DV and LR subdivisions. Therefore **XYZ** is also subject to large variation and is less reproducible than **APT**. The **SC** method can be rendered on the 4D model of the embryo, giving good spatial information (see *ceh-37*::*GFP* below), and on the lineage, giving time information. If the lineage has not been determined, then the **SC** method is powerful and can yield tentative cell identifications. However, like **XYZ**, it is critically dependent on the precise annotation of the initial coordinate system and that the embryo does not deviate from the Ce2008 model. If rotation around the AP axis is observed, a rotation of the model could realign the cells again, although we have not explored this.

**Fig 10:**
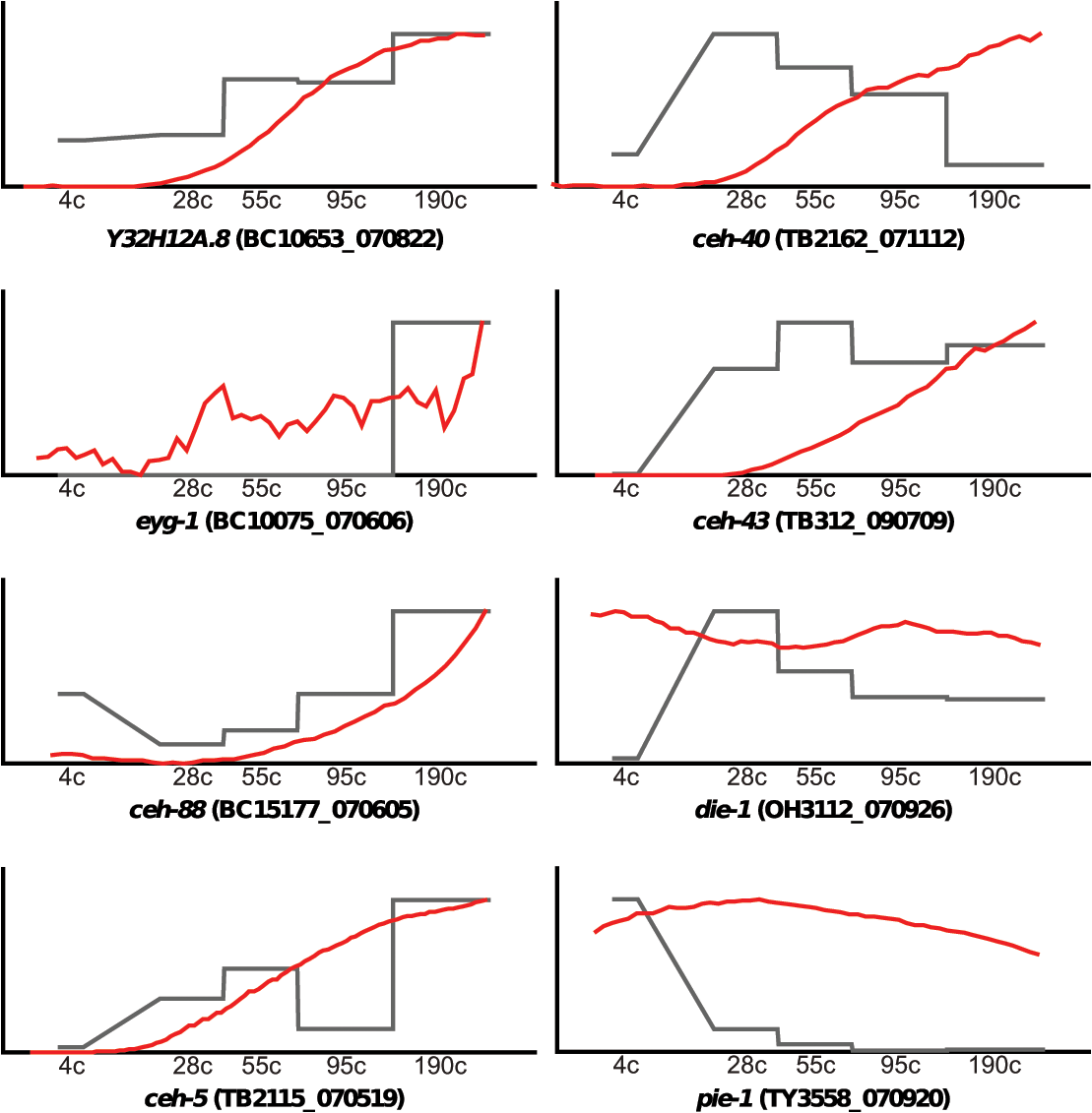
Comparison of T profiles against microarray data of staged embryos. Profiles of eight genes are shown, the remainder is available in the online material. The X-axis shows the different staged embryos according [56] and the microarray data are plotted in grey. The T profiles (red) have been cropped to only show the corresponding time points. For the Y-axis a relative scale had to be used, normalized for the maximal signal within the examined time period. Overall, most of the profiles agree qualitatively, but there are exceptions. For example, the recording for *ceh-5* shows a continuous increase in signal while microarrays show a temporary dip in transcription. Unless this is an experimental artifact, it could hypothetically mean that the GFP protein remains stable, while transcription turns off and is restarted again. However, we do not have enough data points and samples to prove this statistically. Similarly, GFP protein stability may also explain the persistence of *pie-1*::*GFP* expression. Given that all profiles have been rescaled for the Y-axis, this can sometimes give the appearance of a signal due to autofluorescence background that is expanded (e.g., for *ceh-10*). Overall, when taking special conditions into account (low level, extraneous signal, shift in time, etc.) the data are comparable.

The **T** profile is useful for comparing data to other global data derived from sources such as microarrays, SAGE or deep sequencing. Staged *C*. *elegans* embryonic gene expression levels have previously been analyzed using microarrays [56, 76]. We have compared our **T** data with the microarray embryo data at the gene level. Even though a delay between mRNA levels (microarray) and GFP production is expected, we find with 94% statistical significance a low correlation of 0.14. Qualitatively, when examining individual genes, we often find good correspondence (Fig 10).

### Expression patterns of homeobox genes

The 4D expression patterns can be viewed at http://www.endrov.net/paper/4d/ in their summary form as **T**, **APT**, and **XYZ** profiles, and as thumbnail movies. The **SC** data can be downloaded and overlaid on the *C*. *elegans* model and viewed in Endrov. The original 4D image data can be viewed with Endrov.

**Fig 11:**
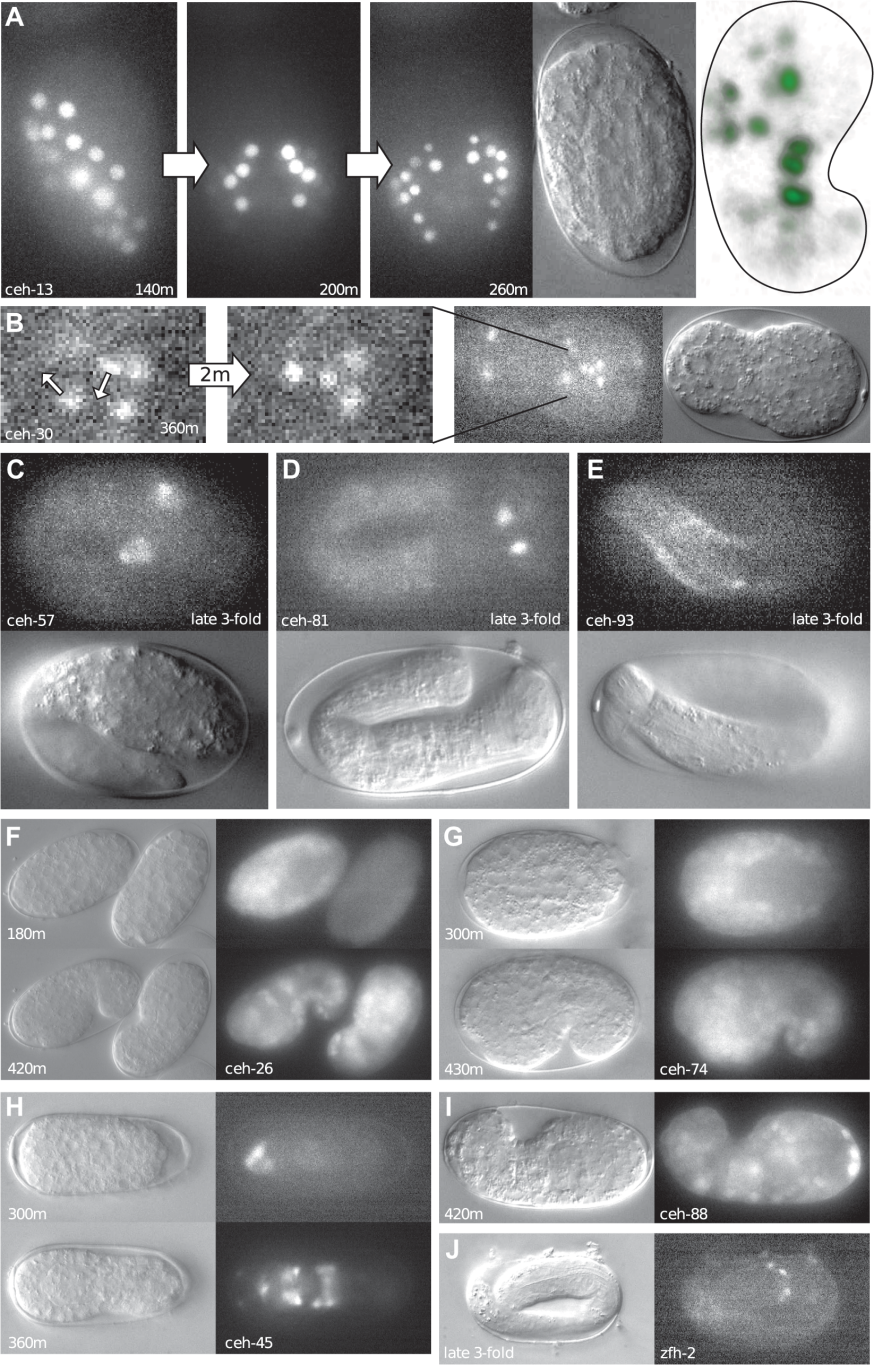
Examples of homeobox::GFP expression patterns. (A) Spatio-temporal expression of *ceh-13*::*GFP* (Recording: FR317_070308). The last panel on the right shows a 3D rendering from the side at the last time point. Time points are given in minutes (B). An example of cell migration revealed by *ceh-30*::*GFP* expression (Recording: ceh30_reco2). A group of four cells in the head region is arranged in a rhomboid-shaped pattern. Within a few minutes, the posterior cell moves further posteriorly and centrally so that the cells form now a Y-shape. (C) Expression of *ceh-57*::*GFP* in bilateral symmetric cells in the head at two-fold stage (Recording: BC15173_070608). (D) Expression of *ceh-81*::*GFP* in the head at the three-fold stage (Recording: BC15188_070614). (E) Diffuse expression of *ceh-93*::*GFP* in cells near the embryo surface (maybe hypodermis or body muscle) at the three-fold stage (Recording: TB2146_070811). (F) Expression of *ceh-26*::*GFP* (Recording: TB1200_070803), broad expression is seen from gastrulation on. (G) *ceh-74*::*GFP* (Recording: BC15162E3_070312) shows a similar expression pattern to *ceh-26*::*GFP* and hence clusters together with it. (H) Expression of *ceh-45*:*GFP*, early in anterior, expanding to more cells at comma stage (Recording: TB2300_071126). (I) Expression of *ceh-88*::*GFP* in numerous cells at the comma stage (Recording: TB2145_070730). (J) Expression of *zfh-2*::*GFP* in the head at the three-fold stage (Recording: TB2161_071120).

We find that most homeobox genes are expressed later than the 100 cell stage. Examples of early expression are the paralogs *ceh-20* and *ceh-40*, which belong to the PBC group of TALE superclass of homeobox genes [31]. Both *ceh-20* and *ceh-40* are expressed broadly in an overlapping fashion during embryogenesis (see movies), and RNAi experiments have revealed that they have a redundant function during embryogenesis [67]. The PBC-TALE homeobox genes are known interactors of the HOX cluster genes [77–79]. In *C*. *elegans*, the Hox gene *ceh-13*, the labial/Hox1 ortholog, is expressed in early embryogenesis [40, 80, 81]. Our 4D recordings confirm the observed early expression (Fig 11A, movies).

**Fig 12:**
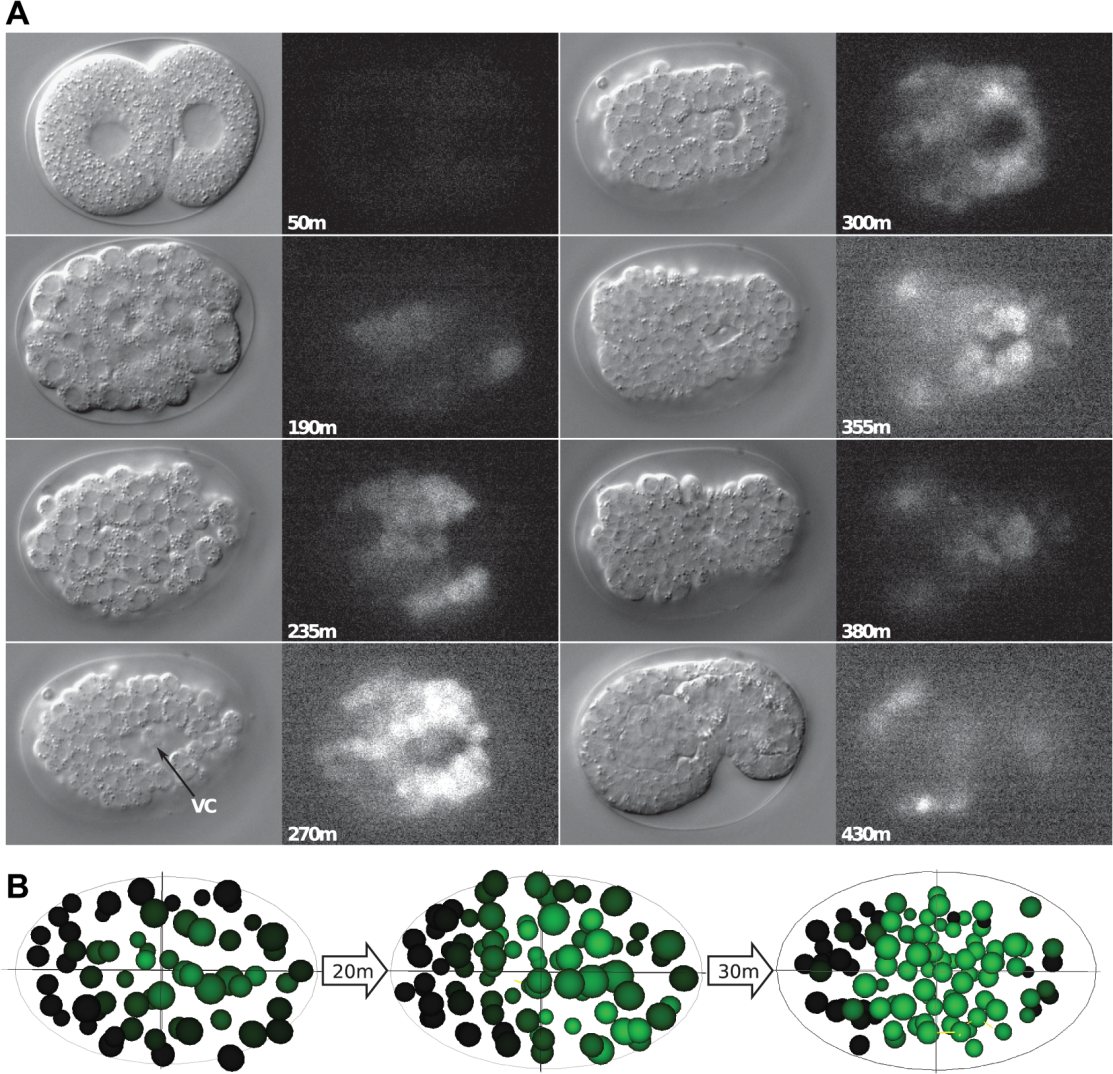
Expression pattern of *ceh-36*::*GFP*. (A) DIC and GFP channels for different time points during gastrulation (Recording: TB2071_080322). Expression is broad, interestingly there is expression around the ventral cleft. (B) **SC** expression mapping of *ceh-36*::*GFP* derived by superimposing the Ce2008 model [12] to extract approximate single-cell expression levels. The mapping suggests that one of the cells expressing *ceh-36*::*GFP* is AB.araap, in the posterior daughter of which *ceh-36* was shown to be responsible for neuronal asymmetry [86].

**Fig 13:**
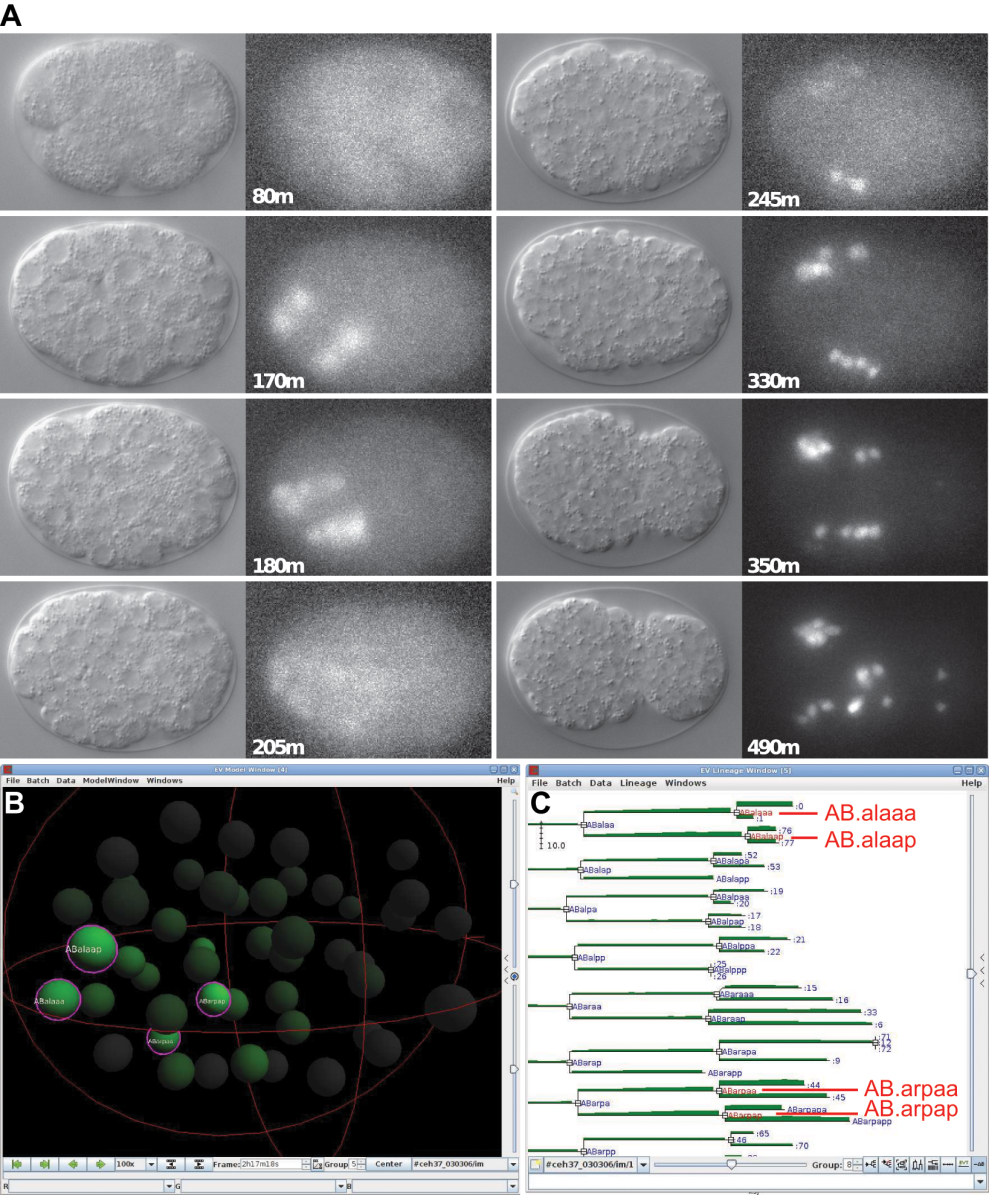
Expression pattern of *ceh-37*::*GFP*. (A) Embryonic expression time points of *ceh-37*::*GFP* (Recording: ceh37_030307). DIC and GFP channels are shown. An early phase of expression is seen in four cells AB.alaaa, AB.alaap, AB.arpaa, ABarpap as determined by manual lineaging, and very weakly in their mothers. This expression fades and later expression arises in neuroblasts that give rise to the cells described ([48], Tong et al., in preparation). (B) **SC** expression mapped onto the Ce2008 model [12]. (C) **SC** expression mapped onto the lineage tree, green above the lineage line represents the GFP signal levels. The same cells as determined by manual lineaging show strong signal.

*ceh-37*, *ttx-1*, and *ceh-36* are Otx/Otd family homeobox genes of the PRD-LIKE class and have been shown to be involved in neurogenesis [48, 82]. In vertebrates, the paralogs OTX1 and OTX2 are required for brain development in a redundant fashion. In addition, OTX2 also plays a role during gastrulation in *Xenopus* and mouse [83–85]. *ceh-36*::*GFP* is expressed during gastrulation, most notably in a region surrounding the ventral cleft (Fig 12A). Deletion mutations in *ceh-36* also show embryonic lethality (Tong et al., in preparation), suggesting a role beyond neurogenesis. Recently, *ceh-36* has been shown to be expressed in the MI progenitor cell AB.araap [86]. SC mapping of *ceh-36*::*GFP* expression supports this finding (Fig 12B). Overall, *ceh-36* might have a role in gastrulation like vertebrate OTX2. *ceh-37*::*GFP* expression is seen early starting at around 40 cells in the daughters of AB.alaa and AB.arpa, and in their daughters in the next division. Then the expression fades (Fig 13). Later expression is seen in the precursors of the neurons, in which *ceh-37* has been shown to be expressed and function ([48], Tong et al., in preparation). The early expression is in different blast cells than those where the later expression is seen, which are daughters of AB.p, AB.alp, and AB.ara. Thus, the early expression is not a precursor for that in later neuroblasts.

Another gene, which has been shown to have a role in gastrulation in vertebrates is the PRD-LIKE homeobox gene *goosecoid* (*gsc*)[87]. However, based on the *ceh-45*::*GFP* expression pattern (Fig 11H), *C*. *elegans gsc* is not involved in gastrulation, but seems involved in neurogenesis, another function of *gsc* [88, 89]. Like *Drosophila*, *C*. *elegans* has a second zinc finger HD protein that we named *zfh-2*. It plays a role in the nervous system in *Drosophila* (see e.g., [90]), and we also see neuronal expression the head in *C*. *elegans* (Fig 11J).

*ceh-26* is an ortholog of the Drosophila gene *prospero*, which is also involved in nervous system specification [91–94]. In addition to neurons [44], *ceh-26* also functions in the excretory cell [95]. However, the expression pattern shows that it is expressed rather broadly, in many neurons and other cells, starting from gastrulation (Fig 11F). In the APT dendrogram (Fig 9), *ceh-26* clusters tightly together with divergent homeobox gene *ceh-74*. It indeed has a broad expression pattern like *ceh-26* (Fig 11G). It would be interesting to see, whether there is a functional link between these two homeobox genes.

We also note that some of the other highly divergent genes are expressed, supporting the transcript data that they are not pseudogenes. For example, *ceh-57* is expressed in bilaterally symmetric neuroblasts in the head (Fig 11C), while the cluster gene *ceh-81* is expressed in a pair of cells in the head, and also the gut (Fig 11D), while *ceh-88* is expressed in many cells (Fig 11I). The HOCHOB gene *ceh-93* is expressed in a number of cells in late embryogenesis (Fig 11E). The diversity of patterns observed for divergent homeobox genes suggests that evolutionary novel innovations are possible at many ontogenetic steps.

## Discussion

Homeobox genes are key developmental regulators. Here, we provide an updated list as well as nomenclature. About 70% of 103 genes have been highly conserved from worms to humans, indicating their fundamental roles in bilaterian development. 15 genes lack orthologs in other *Caenorhabditis* species, indicating fast evolution and divergence, possibly involved in species-specific functions. It is interesting to note therefore that, while many homeobox genes are highly conserved, about 15% are evolving rapidly and thereby possibly contributing to evolutionary diversification. For almost all 15 genes, expression has been demonstrated in the form of transcripts (WormBase). We obtained GFP expression data for some, indicating that at least some are probably functional. Several of these genes are clustered on the left arm of chromosome II that has been subject to substantial gene duplication, demonstrating ongoing evolution. We find that most of the homeobox genes are expressed later in embryogenesis, most likely reflecting the fact they are involved in final cell fate specification events. However, those genes that are expressed during gastrulation, or even earlier, such as the TALE and HOX homeobox genes (Figs 2 and 3, S1 Text), have been shown or can be suspected to play roles during the establishment of the body plan [96].

In order to understand how transcription factors regulate cell fates during development, it is essential to obtain their precise spatio-temporal expression profiles. Due to the lack of suitable tools to achieve that goal, we have developed a workflow for the 4D imaging framework, Endrov. We aimed to develop a general tool that can be used with many different microscopy platforms, in particular also with regular DIC fluorescent microscopes that are widely available in the field. An important limitation during recording is sample viability. We have addressed this by employing halogen and LED light sources. Further, the recording parameters, such as binning, number of slices, and temporal spacing of the fluorescent stacks can be flexibly adjusted to reduce sample damage. In addition, we can also adjust exposure time during recording to increase the dynamic range of signal intensities that can be recorded. An unlimited number of channels can be recorded, allowing 4D recordings with multiple markers, or parallel recordings of the same channel with different exposure times, allowing visualization of strong and weak expression at the same time. No other software offers such flexibility.

We find that the expression patterns of individual GFP lines are, like the lineage, qualitatively very reproducible. These normal, non-deconvoluted fluorescent microscopy images can provide very detailed expression information, if expression is not broad and highly overlapping. For example, in the case of *ceh-30*, rearrangement of cells during the final stages of neurogenesis can be followed in the anterior of the embryo (Fig 11B, see online movies).

Through normalization procedures, it is possible to compare recordings, and data can be extracted and viewed on an abstract model of *C*. *elegans*. Super-imposing the standard *C*. *elegans* model to obtain single-cell resolution, instead of lineaging the recording, works surprisingly well. Super-imposed lineages can probably be compared with hand-annotated lineages, making them a very quick estimate and potentially good enough to identify patterns worth pursuing. For *C*. *elegans*, gene expression patterns will ultimately always be mapped to single cells (e.g., [29]).

Our method of summarizing and comparing expression patterns is not restricted to *C*. *elegans*. Most biological model systems do not have a precise lineage like *C*. *elegans*. The gene expression extracting algorithms we have developed as part of Endrov can certainly be applied to other systems, i.e. embryogenesis in other species, or *in vitro* organ development, where precise cell lineage is not available, but spatial patterns of gene expression can be observed.

Our expression survey of homeobox genes contributes to the ongoing efforts to determine gene expression patterns and functions of developmental control genes [25, 29, 97]. Comparison with the EPIC data from Murray et al. (2012) [29] shows that 13 homeobox genes are in both data sets. Visual inspection shows that most patterns look comparable. There are some differences though, for example *ceh-16* and *ceh-14* look different, with *ceh-14* almost completely lacking expression in EPIC embryos [24]. Also, *pal-1* expression in EPIC starts later than the early expression seen with antibodies [98]. Certainly, some of the differences are due to different methodologies, e.g., different types of reporter constructs can show different expression (e.g., [37]). Nevertheless, it highlights the fact that the more data, best using different methodologies, can be acquired, the better. Our 4D system is not particularly demanding on hardware, so can be used widely in the field for a variety of purposes to complement other efforts.

## Supporting Information

S1 Fig Protein logo created from *C*. *elegans* HD sequences. Sequences from Figs 3 and 4 were analyzed using LogoBar. Due to the fact that this alignment includes a number of divergent HDs, the sequence conservation is not as strong as in the original profile of 346 HDs [2, 71], although it still follows the same pattern with the strongest conservation in the DNA-binding helix 3.
(PDF)

S2 Fig Sequence of ZFH-2, a C_2_H_2_ zinc finger HD protein. The three HDs are marked in red, the 15 zinc fingers are marked in green, cysteine and histidine residues are yellow. Two partial fingers are underlined, the second may form a finger using an Asp (D) residue.
(PDF)

S3 Fig Map of chromosome II. Expanded view of chromosome II (expanded from Fig 8), showing additional gene families, i.e. *math*, *btb*, *fbxa*, *fbxb*, and *fbxc* genes. Homeobox genes are marked in red.
(PDF)

S4 Fig A new conserved cysteine motif upstream of CEH-86. (A) Multiple sequence alignment of *Caenorhabditis* ORFs that share sequence similarity with the upstream region of CEH-86. A blastp search with CEH-86 retrieved three *C*. *elegans* ORFs, all located on cosmid C35E7, as well as related genes from other *Caenorhabditis* species. No similarity was found beyond *Caenorhabditis*. The sequence similarity starts at the N-terminus and extends to the HD of CEH-86. Furthermore, the newly identified ORFs extend their sequence similarity into the region that corresponds to the HD of CEH-86 and beyond. The sequence conservation is characterized by conserved cysteine residues, suggesting that multiple metal (usually zinc) binding fingers may be present. However, further analysis will be necessary to define the motif in depth, at present we refer to it as UCM (uncharacterized cysteine motif). The location of a HD attached to another protein coding region suggests that *ceh-86* may have arisen by a duplication event (maybe from the *ceh-84* homeobox), where a homeobox translocated into a UCM gene, or an N-terminal section of a UCM gene translocated upstream of a homeobox. (B) Neighbor-joining tree of the conserved region of the sequences in (A). Numbers show bootstrap values for 1000 trial runs. The tree shows that three clades exist that share orthologous genes in different *Caenorhabditis* species. A chromosomal cluster with multiple genes must have already existed before the divergence of *C*. *elegans*, *C*. *briggsae*, and *C*. *remanei*. It appears that CEH-86 does not seem to have a direct ortholog, supporting the notion of a recent duplication event.
(PDF)

S1 Table List of strains used for 4D analysis. The third column gives TB strain designations, the fourth column are strains from other sources. TB strains were often derived from BC strains by integration. Some strains were obtained from CGC. Sources of additional strains: *tbx-2*::*GFP* [99], *xbx-1*::*GFP* [100], *F55A4*.*3*::*GFP* (+ *elt-2*::*mCherry*) [101], *efn-4*::*GFP* [102], *pie-1*::*GFP*::*HIS-11* [103], *mec-18*::*GFP* [104].
(DOC)

S1 Text References for *C*. *elegans* homeobox genes. Extracted from WormBase release WS220 with BioMart. Microsoft Word document, zipped.
(ZIP)

## References

[1] Mukherjee K, Brocchieri L, Bürglin TR. A comprehensive classification and evolutionary analysis of plant homeobox genes. Molecular biology and evolution. 2009;26(12):2775–94 10.1093/molbev/msp201 19734295 PMC2775110

[2] Bürglin TR. Homeodomain subtypes and functional diversity. Subcell Biochem. 2011;52:95–122 10.1007/978-90-481-9069-0_5 21557080

[3] Ruvkun G, Hobert O. The taxonomy of developmental control in *Caenorhabditis elegans* . Science (New York, NY). 1998;282:2033–41. 9851920 10.1126/science.282.5396.2033

[4] Reece-Hoyes JS, Deplancke B, Shingles J, Grove CA, Hope IA, Walhout AJ. A compendium of *Caenorhabditis elegans* regulatory transcription factors: a resource for mapping transcription regulatory networks. Genome biology. 2005;6(13):R110 . 16420670 10.1186/gb-2005-6-13-r110 PMC1414109

[5] Riddle DL, Blumenthal T, Meyer BJ, Priess JR. *C. elegans* II Cold Spring Harbor, New York: Cold Spring Harbor Laboratory Press; 1997. 1222 p.

[6] Sulston JE, Horvitz HR. Post-embryonic cell lineages of the nematode, *Caenorhabditis elegans* . Dev Biol. 1977;56:110–56. 838129 10.1016/0012-1606(77)90158-0

[7] Sulston JE, Schierenberg E, White JG, Thomson JN. The embryonic cell lineage of the nematode *Caenorhabditis elegans* . Dev Biol. 1983;100:64–119. 6684600 10.1016/0012-1606(83)90201-4

[8] Thomas C, DeVries P, Hardin J, White J. Four-dimensional imaging: computer visualization of 3D movements in living specimens. Science (New York, NY. 1996;273(5275):603–7 . 8662545 10.1126/science.273.5275.603

[9] Schnabel R, Hutter H, Moerman D, Schnabel H. Assessing normal embryogenesis in *Caenorhabditis elegans* using a 4D microscope: variability of development and regional specification. Developmental biology. 1997;184(2):234–65 . 9133433 10.1006/dbio.1997.8509

[10] Hejnol A, Schnabel R. What a couple of dimensions can do for you: Comparative developmental studies using 4D microscopy—examples from tardigrade development. Integr Comp Biol. 2006;46(2):151–61 10.1093/icb/icj012 21672732

[11] Bürglin TR. A two-channel four-dimensional image recording and viewing system with automatic drift correction. J Microsc. 2000;200(Pt 1):75–80. 11012831 10.1046/j.1365-2818.2000.00741.x

[12] Hench J, Henriksson J, Lüppert M, Bürglin TR. Spatio-temporal reference model of *Caenorhabditis elegans* embryogenesis with cell contact maps. Developmental biology. 2009;333(1):1–13 10.1016/j.ydbio.2009.06.014 19527702

[13] Schulze J, Schierenberg E. Evolution of embryonic development in nematodes. EvoDevo. 2011;2(1):18 10.1186/2041-9139-2-18 21929824 PMC3195109

[14] Chalfie M, Tu Y, Euskirchen G, Ward WW, Prasher DC. Green Fluorescent Protein as a marker for gene expression. Science (New York, NY). 1994;263:802–5. 10.1126/science.8303295 8303295

[15] Mohler WA, White JG. Stereo-4-D reconstruction and animation from living fluorescent specimens. Biotechniques. 1998;24:1006 9631195

[16] Thomas CF, White JG. Four-dimensional imaging: the exploration of space and time. Trends Biotechnol. 1998;16(4):175–82. 9586240 10.1016/s0167-7799(97)01169-4

[17] Boyle TJ, Bao Z, Murray JI, Araya CL, Waterston RH. AceTree: a tool for visual analysis of *Caenorhabditis elegans* embryogenesis. BMC bioinformatics. 2006;7:275 . 16740163 10.1186/1471-2105-7-275 PMC1501046

[18] Murray JI, Bao Z, Boyle TJ, Waterston RH. The lineaging of fluorescently-labeled *Caenorhabditis elegans* embryos with StarryNite and AceTree. Nat Protoc. 2006;1(3):1468–76 . 17406437 10.1038/nprot.2006.222

[19] Zhao Z, Boyle TJ, Bao Z, Murray JI, Mericle B, Waterston RH. Comparative analysis of embryonic cell lineage between *Caenorhabditis briggsae* and *Caenorhabditis elegans* . Developmental biology. 2008;314(1):93–9 10.1016/j.ydbio.2007.11.015 18164284 PMC2696483

[20] Bürglin TR, Finney M, Coulson A, Ruvkun G. *Caenorhabditis elegans* has scores of homoeobox-containing genes. Nature. 1989;341:239–43. 2571091 10.1038/341239a0

[21] Dozier C, Kagoshima H, Niklaus G, Cassata G, Bürglin TR. The *Caenorhabditis elegans* Six/sine oculis class homeobox gene *ceh-32* is required for head morphogenesis. Dev Biol. 2001;236:289–303. 11476572 10.1006/dbio.2001.0325

[22] Bürglin TR, Ruvkun G. Regulation of ectodermal and excretory function by the *C. elegans* POU homeobox gene *ceh-6* . Development (Cambridge, England). 2001;128:779–90. 11171402 10.1242/dev.128.5.779

[23] Aspöck G, Ruvkun G, Bürglin TR. The *Caenorhabditis elegans* ems class homeobox gene *ceh-2* is required for M3 pharynx motoneuron function. Development (Cambridge, England). 2003;130(15):3369–78 . 12810585 10.1242/dev.00551

[24] Kagoshima H, Cassata G, Tong YG, Pujol N, Niklaus G, Bürglin TR. The LIM homeobox gene *ceh-14* is required for phasmid function and neurite outgrowth. Developmental biology. 2013;380(2):314–23 10.1016/j.ydbio.2013.04.009 23608457

[25] Hunt-Newbury R, Viveiros R, Johnsen R, Mah A, Anastas D, Fang L, et al High-throughput in vivo analysis of gene expression in *Caenorhabditis elegans* . PLoS Biol. 2007;5(9):e237 . 17850180 10.1371/journal.pbio.0050237 PMC1971126

[26] Henriksson J, Hench J, Tong YG, Johansson A, Johansson D, Bürglin TR. Endrov: an integrated platform for image analysis. Nature methods. 2013;10(6):454–6 10.1038/nmeth.2478 23722203

[27] Hamahashi S, Onami S, Kitano H. Detection of nuclei in 4D Nomarski DIC microscope images of early Caenorhabditis elegans embryos using local image entropy and object tracking. BMC bioinformatics. 2005;6:125 . 15910690 10.1186/1471-2105-6-125 PMC1175842

[28] Bao Z, Murray JI, Boyle T, Ooi SL, Sandel MJ, Waterston RH. Automated cell lineage tracing in *Caenorhabditis elegans* . Proceedings of the National Academy of Sciences of the United States of America. 2006;103(8):2707–12 . 16477039 10.1073/pnas.0511111103 PMC1413828

[29] Murray JI, Boyle TJ, Preston E, Vafeados D, Mericle B, Weisdepp P, et al Multidimensional regulation of gene expression in the *C. elegans* embryo. Genome research. 2012;22(7):1282–94 10.1101/gr.131920.111 22508763 PMC3396369

[30] Pérez-Bercoff Å, Koch J, Bürglin TR. LogoBar: bar graph visualization of protein logos with gaps. Bioinformatics (Oxford, England). 2006;22(1):112–4. 16269415 10.1093/bioinformatics/bti761

[31] Mukherjee K, Bürglin TR. Comprehensive Analysis of Animal TALE Homeobox Genes: New Conserved Motifs and Cases of Accelerated Evolution. Journal of molecular evolution. 2007;65(2):137–53 . 17665086 10.1007/s00239-006-0023-0

[32] Bürglin TR. Evolution of hedgehog and hedgehog-related genes, their origin from Hog proteins in ancestral eukaryotes and discovery of a novel Hint motif. BMC genomics. 2008;9:127 10.1186/1471-2164-9-127 18334026 PMC2362128

[33] Pérez-Bercoff Å, Bürglin TR. LogoBar—Visualizing protein sequence logos with gaps In: Fung GPC, editor. Sequence and Genome Analysis: Methods and Applications II. Hong Kong: iConcept Press Ltd.; 2010.

[34] Finn RD, Clements J, Eddy SR. HMMER web server: interactive sequence similarity searching. Nucleic acids research. 2011;39(Web Server issue):W29–37 10.1093/nar/gkr367 21593126 PMC3125773

[35] Bürglin TR. Homeodomain proteins In: Meyers RA, editor. Encyclopedia of Molecular Cell Biology and Molecular Medicine. 6 2nd Edition ed. Weinheim: Wiley-VCH Verlag GmbH & Co.; 2005 p. 179–222.

[36] Holland PW, Booth HA, Bruford EA. Classification and nomenclature of all human homeobox genes. BMC biology. 2007;5:47 . 17963489 10.1186/1741-7007-5-47 PMC2211742

[37] Reece-Hoyes JS, Shingles J, Dupuy D, Grove CA, Walhout AJ, Vidal M, et al Insight into transcription factor gene duplication from *Caenorhabditis elegans* Promoterome-driven expression patterns. BMC genomics. 2007;8:27 . 17244357 10.1186/1471-2164-8-27 PMC1785375

[38] Inoue T, Sherwood DR, Aspöck G, Butler JA, Gupta BP, Kirouac M, et al Gene expression markers for *Caenorhabditis elegans* vulval cells. Mechanisms of development. 2002;119 Suppl 1:S203–9 . 14516686 10.1016/s0925-4773(03)00117-5

[39] Struckhoff EC, Lundquist EA. The actin-binding protein UNC-115 is an effector of Rac signaling during axon pathfinding in *C. elegans* . Development (Cambridge, England). 2003;130(4):693–704 . 12506000 10.1242/dev.00300

[40] Streit A, Kohler R, Marty T, Belfiore M, Takacs-Vellai K, Vigano MA, et al Conserved regulation of the *Caenorhabditis elegans* labial/Hox1 gene *ceh-13* . Dev Biol. 2002;242(2):96–108 . 11820809 10.1006/dbio.2001.0544

[41] Cassata G, Kagoshima H, Andachi Y, Kohara Y, Dürrenberger MB, Hall DH, et al The LIM homeobox gene *ceh-14* confers thermosensory function to the AFD neurons in *Caenorhabditis elegans* . Neuron. 2000;25:587–97. 10774727 10.1016/s0896-6273(00)81062-4

[42] Okkema PG, Fire A. The *Caenorhabditis elegans* NK-2 class homeoprotein CEH-22 is involved in combinatorial activation of gene expression in pharyngeal muscle. Development (Cambridge, England). 1994;120:2175–86. 7925019 10.1242/dev.120.8.2175

[43] Yanowitz JL, Shakir MA, Hedgecock E, Hutter H, Fire AZ, Lundquist EA. UNC-39, the *C. elegans* homolog of the human myotonic dystrophy-associated homeodomain protein Six5, regulates cell motility and differentiation. Developmental biology. 2004;272(2):389–402 . 15282156 10.1016/j.ydbio.2004.05.010

[44] Yu H, Prétôt RF, Bürglin TR, Sternberg PW. Distinct roles of transcription factors EGL-46 and DAF-19 in specifying the functionality of a polycystin-expressing sensory neuron necessary for *C. elegans* male vulva location behavior. Development (Cambridge, England). 2003;130(21):5217–27 . 12954713 10.1242/dev.00678

[45] Peden E, Kimberly E, Gengyo-Ando K, Mitani S, Xue D. Control of sex-specific apoptosis in *C. elegans* by the BarH homeodomain protein CEH-30 and the transcriptional repressor UNC-37/Groucho. Genes & development. 2007;21(23):3195–207 . 18056429 10.1101/gad.1607807 PMC2081983

[46] Cassata G, Kagoshima H, Prétôt RF, Aspöck G, Niklaus G, Bürglin TR. Rapid expression screening of *C. elegans* homeobox genes using a two-step polymerase chain reaction promoter-GFP reporter construction technique. Gene. 1998;212(1):127–35. 9661672 10.1016/s0378-1119(98)00137-1

[47] Aspöck G, Bürglin TR. The *Caenorhabditis elegans distal-less* ortholog *ceh-43* is required for development of the anterior hypodermis. Dev Dyn. 2001;222(3):403–9 . 11747075 10.1002/dvdy.1201

[48] Lanjuin A, VanHoven MK, Bargmann CI, Thompson JK, Sengupta P. Otx/otd homeobox genes specify distinct sensory neuron identities in *C. elegans* . Developmental cell. 2003;5(4):621–33 . 14536063 10.1016/s1534-5807(03)00293-4

[49] Abdus-Saboor I, Mancuso VP, Murray JI, Palozola K, Norris C, Hall DH, et al Notch and Ras promote sequential steps of excretory tube development in *C. elegans* . Development (Cambridge, England). 2011;138(16):3545–55 10.1242/dev.068148 21771815 PMC3143567

[50] Röhrig S, Röckelein I, Donhauser R, Baumeister R. Protein interaction surface of the POU transcription factor UNC-86 selectively used in touch neurons. The EMBO journal. 2000;19(14):3694–703 . 10899123 10.1093/emboj/19.14.3694 PMC313964

[51] Zhang S, Ma C, Chalfie M. Combinatorial marking of cells and organelles with reconstituted fluorescent proteins. Cell. 2004;119(1):137–44 . 15454087 10.1016/j.cell.2004.09.012

[52] Evans TC. Transformation and microinjection. In: The C. elegans Research Community, editor. WormBook2006. p. 10.1895/wormbook.1.108.1, http://www.wormbook.org.

[53] Mello C, Fire A. DNA transformation In: Epstein HF, Shakes DC, editors. *Caenorhabditis elegans*: Modern Biological Analysis of an Organism. Methods in Cell Biology 48 San Diego, London: Academic Press, Inc.; 1995 p. 451–82. 8531738

[54] Barnett AG, van der Pols JC, Dobson AJ. Regression to the mean: what it is and how to deal with it. Int J Epidemiol. 2005;34(1):215–20. 10.1093/ije/dyh299 . 15333621

[55] Manders EMM, Verbeek FJ, Aten JA. Measurement of co-localization of objects in dual-colour confocal images. J of Microscopy. 1993;169(Pt 3):375–82. 10.1111/j.1365-2818.1993.tb03313.x 33930978

[56] Yanai I, Hunter CP. Comparison of diverse developmental transcriptomes reveals that coexpression of gene neighbors is not evolutionarily conserved. Genome research. 2009;19(12):2214–20 10.1101/gr.093815.109 19745112 PMC2792179

[57] Barrett T, Wilhite SE, Ledoux P, Evangelista C, Kim IF, Tomashevsky M, et al NCBI GEO: archive for functional genomics data sets—update. Nucleic acids research. 2013;41(Database issue):D991–5 10.1093/nar/gks1193 23193258 PMC3531084

[58] Felsenstein J. PHYLIP—Phylogeny Inference Package (Version 3.2). Cladistics. 1989;5:164–6.

[59] Perrière G, Gouy M. WWW-query: an on-line retrieval system for biological sequence banks. Biochimie. 1996;78(5):364–9 . 8905155 10.1016/0300-9084(96)84768-7

[60] Hobert O, Ruvkun G. Pax genes in *Caenorhabditis elegans*: a new twist. Trends Genet. 1999;15(6):214–6 . 10354580 10.1016/s0168-9525(99)01731-x

[61] Bürglin TR, Cassata G. Loss and gain of domains during evolution of cut superclass homeobox genes. Int J Dev Biol. 2002;46:115–23. 11902672

[62] Svendsen PC, McGhee JD. The *C. elegans* neuronally expressed homeobox gene *ceh-10* is closely related to genes expressed in the vertebrate eye. Develoment. 1995;121:1253–62. 7789259 10.1242/dev.121.5.1253

[63] Chow RL, Snow B, Novak J, Looser J, Freund C, Vidgen D, et al *Vsx1*, a rapidly evolving *paired*-like homeobox gene expressed in cone bipolar cells. Mechanisms of development. 2001;109(2):315–22 . 11731243 10.1016/s0925-4773(01)00585-8

[64] Clouaire T, Roussigne M, Ecochard V, Mathe C, Amalric F, Girard JP. The THAP domain of THAP1 is a large C2CH module with zinc-dependent sequence-specific DNA-binding activity. Proceedings of the National Academy of Sciences of the United States of America. 2005;102(19):6907–12 . 15863623 10.1073/pnas.0406882102 PMC1100732

[65] Kagoshima H, Cassata G, Bürglin TR. A *Caenorhabditis elegans* homeobox gene expressed in the male tail, a link between pattern formation and sexual dimophism? Dev Genes Evol. 1999;209:59–62. 9914419 10.1007/s004270050227

[66] Bürglin TR. Analysis of TALE superclass homeobox genes (MEIS, PBC, KNOX, Iroquois, TGIF) reveals a novel domain conserved between plants and animals. Nucl Acids Res. 1997;25:4173–80. 9336443 10.1093/nar/25.21.4173 PMC147054

[67] Van Auken K, Weaver D, Robertson B, Sundaram M, Saldi T, Edgar L, et al Roles of the Homothorax/Meis/Prep homolog UNC-62 and the Exd/Pbx homologs CEH-20 and CEH-40 in *C. elegans* embryogenesis. Development (Cambridge, England). 2002;129(22):5255–68 . 12399316 10.1242/dev.129.22.5255

[68] Leidenroth A, Hewitt JE. A family history of DUX4: phylogenetic analysis of DUXA, B, C and Duxbl reveals the ancestral DUX gene. BMC evolutionary biology. 2010;10:364 10.1186/1471-2148-10-364 21110847 PMC3004920

[69] Underhill DA. PAX proteins and fables of their reconstruction. Critical reviews in eukaryotic gene expression. 2012;22(2):161–77 . 22856433 10.1615/critreveukargeneexpr.v22.i2.70

[70] Stein LD, Bao Z, Blasiar D, Blumenthal T, Brent MR, Chen N, et al The genome sequence of *Caenorhabditis briggsae*: a platform for comparative genomics. PLoS Biol. 2003;1(2):E45 . 14624247 10.1371/journal.pbio.0000045 PMC261899

[71] Bürglin TR. A comprehensive classification of homeobox genes In: Duboule D, editor. Guidebook to the Homeobox Genes. Oxford: Oxford University Press; 1994 p. 25–71.

[72] Minguillon C, Garcia-Fernandez J. Genesis and evolution of the Evx and Mox genes and the extended Hox and ParaHox gene clusters. Genome biology. 2003;4(2):R12 . 12620122 10.1186/gb-2003-4-2-r12 PMC151302

[73] Bratic I, Hench J, Henriksson J, Antebi A, Bürglin TR, Trifunovic A. Mitochondrial DNA level, but not active replicase, is essential for *Caenorhabditis elegans* development. Nucleic acids research. 2009;37(6):1817–28 10.1093/nar/gkp018 19181702 PMC2665216

[74] Tenenhaus C, Schubert C, Seydoux G. Genetic requirements for PIE-1 localization and inhibition of gene expression in the embryonic germ lineage of *Caenorhabditis elegans* . Developmental biology. 1998;200(2):212–24 . 9705228 10.1006/dbio.1998.8940

[75] Nance J, Munro EM, Priess JR. *C. elegans* PAR-3 and PAR-6 are required for apicobasal asymmetries associated with cell adhesion and gastrulation. Development (Cambridge, England). 2003;130(22):5339–50 . 13129846 10.1242/dev.00735

[76] Baugh LR, Hill AA, Slonim DK, Brown EL, Hunter CP. Composition and dynamics of the *Caenorhabditis elegans* early embryonic transcriptome. Development (Cambridge, England). 2003;130(5):889–900 . 12538516 10.1242/dev.00302

[77] Mann RS, Affolter M. Hox proteins meet more partners. Current opinion in genetics & development. 1998;8(4):423–9. 9729718 10.1016/s0959-437x(98)80113-5

[78] Liu J, Fire A. Overlapping roles of two Hox genes and the exd ortholog *ceh-20* in diversification of the *C. elegans* postembryonic mesoderm. Development (Cambridge, England). 2000;127(23):5179–90 . 11060243 10.1242/dev.127.23.5179

[79] Mann RS, Lelli KM, Joshi R. Hox specificity: unique roles for cofactors and collaborators. Current topics in developmental biology. 2009;88:63–101. 10.1016/S0070-2153(09)88003-4 19651302 PMC2810641

[80] Brunschwig K, Wittmann C, Schnabel R, Bürglin TR, Tobler H, Müller F. Anterior organization of the *Caenorhabditis elegans* embryo by the *labial*-like Hox gene *ceh-13* . Development (Cambridge, England). 1999;126(7):1537–46. 10068646 10.1242/dev.126.7.1537

[81] Wittmann C, Bossinger O, Goldstein B, Fleischmann M, Kohler R, Brunschwig K, et al The expression of the *C. elegans* labial-like Hox gene *ceh-13* during early embryogenesis relies on cell fate and on anteroposterior cell polarity. Development (Cambridge, England). 1997;124(21):4193–200 . 9334268 10.1242/dev.124.21.4193

[82] Satterlee JS, Sasakura H, Kuhara A, Berkeley M, Mori I, Sengupta P. Specification of thermosensory neuron fate in *C. elegans* requires *ttx-1*, a Homolog of otd/Otx. Neuron. 2001;31:943–56. 11580895 10.1016/s0896-6273(01)00431-7

[83] Simeone A, Acampora D. The role of Otx2 in organizing the anterior patterning in mouse. The International journal of developmental biology. 2001;45(1):337–45 . 11291864

[84] De Robertis EM, Wessely O, Oelgeschlager M, Brizuela B, Pera E, Larrain J, et al Molecular mechanisms of cell-cell signaling by the Spemann-Mangold organizer. The International journal of developmental biology. 2001;45(1):189–97 . 11291846 PMC2354921

[85] Boyl PP, Signore M, Annino A, Barbera JP, Acampora D, Simeone A. Otx genes in the development and evolution of the vertebrate brain. Int J Dev Neurosci. 2001;19(4):353–63 . 11378295 10.1016/s0736-5748(01)00003-x

[86] Nakano S, Ellis RE, Horvitz HR. Otx-dependent expression of proneural bHLH genes establishes a neuronal bilateral asymmetry in *C. elegans* . Development (Cambridge, England). 2010;137(23):4017–27 10.1242/dev.058834 21041366 PMC2976285

[87] Blum M, Gaunt SJ, Cho KWY, Steinbeisser H, Blumberg B, Bittner D, et al Gastrulation in the mouse: the role of the homeobox gene *goosecoid* . Cell. 1992;69:1097–106. 1352187 10.1016/0092-8674(92)90632-m

[88] Goriely A, Stella M, Coffinier C, Kessler D, Mailhos C, Dessain S, et al A functional homologue of *goosecoid* in *Drosophila* . Development (Cambridge, England). 1996;122(5):1641–50 . 8625850 10.1242/dev.122.5.1641

[89] Lemaire L, Roeser T, Izpisúa-Belmonte JC, Kessel M. Segregating expression domains of two *goosecoid* genes during the transition from gastrulation to neurulation in chick embryos. Development (Cambridge, England). 1997;124:1443–52. 9108361 10.1242/dev.124.8.1443

[90] Lundell MJ, Hirsh J. The *zfh-2* gene product is a potential regulator of neuron-specific DOPA decarboxylase gene expression in *Drosophila* . Dev Biology. 1992;154:84–94. 1426635 10.1016/0012-1606(92)90050-q

[91] Chu-Lagraff Q, Wright DM, McNeil LK, Doe CQ. The *prospero* gene encodes a divergent homeodomain protein that controls neuronal identity in *Drosophila* . Development (Cambridge, England). 1991;Supplement 2:79–85. 1842358

[92] Doe CQ, Chu-LaGraff Q, Wright DM, Scott MP. The *prospero* gene specifies cell fates in the *Drosophila* central nervous system. Cell. 1991;65:451–64. 1673362 10.1016/0092-8674(91)90463-9

[93] Vaessin H, Grell E, Wolff E, Bier E, Jan LY, Jan YN. *prospero* is expressed in neuronal precursors and encodes a nuclear protein that is involved in the control of axonal outgrowth in *Drosophila* . Cell. 1991;67:941–53. 1720353 10.1016/0092-8674(91)90367-8

[94] Matsuzaki F, Koizumi K, Hama C, Yoshioka T, Nabeshima Y. Cloning of the *Drosophila prospero* gene and its expression in ganglion mother cells. Biochem Biophys Res Commun. 1992;182:1326–32. 1540176 10.1016/0006-291x(92)91878-t

[95] Kolotuev I, Hyenne V, Schwab Y, Rodriguez D, Labouesse M. A pathway for unicellular tube extension depending on the lymphatic vessel determinant Prox1 and on osmoregulation. Nature cell biology. 2013;15(2):157–68 10.1038/ncb2662 23334499

[96] Duboule D. The rise and fall of Hox gene clusters. Development (Cambridge, England). 2007;134(14):2549–60. 10.1242/dev.001065 . 17553908

[97] McKay SJ, Johnsen R, Khattra J, Asano J, Baillie DL, Chan S, et al Gene expression profiling of cells, tissues, and developmental stages of the nematode *C. elegans* . Cold Spring Harb Symp Quant Biol. 2003;68:159–69 . 15338614 10.1101/sqb.2003.68.159

[98] Hunter CP, Kenyon C. Spatial and temporal controls target *pal-1* blastomere-specification activity to a single blastomere lineage in *C. elegans* embryos. Cell. 1996;87:217–26. 8861906 10.1016/s0092-8674(00)81340-9

[99] Roy Chowdhuri S, Crum T, Woollard A, Aslam S, Okkema PG. The T-box factor TBX-2 and the SUMO conjugating enzyme UBC-9 are required for ABa-derived pharyngeal muscle in *C. elegans* . Developmental biology. 2006;295(2):664–77 . 16701625 10.1016/j.ydbio.2006.04.001

[100] Schafer JC, Haycraft CJ, Thomas JH, Yoder BK, Swoboda P. XBX-1 encodes a dynein light intermediate chain required for retrograde intraflagellar transport and cilia assembly in *Caenorhabditis elegans* . Mol Biol Cell. 2003;14(5):2057–70 . 12802075 10.1091/mbc.E02-10-0677 PMC165097

[101] Phirke P, Efimenko E, Mohan S, Burghoorn J, Crona F, Bakhoum MW, et al Transcriptional profiling of *C. elegans* DAF-19 uncovers a ciliary base-associated protein and a CDK/CCRK/LF2p-related kinase required for intraflagellar transport. Developmental biology. 2011;357(1):235–47 10.1016/j.ydbio.2011.06.028 21740898 PMC3888451

[102] Chin-Sang ID, Moseley SL, Ding M, Harrington RJ, George SE, Chisholm AD. The divergent *C. elegans* ephrin EFN-4 functions inembryonic morphogenesis in a pathway independent of the VAB-1 Eph receptor. Development (Cambridge, England). 2002;129(23):5499–510 . 12403719 10.1242/dev.00122

[103] Strome S, Powers J, Dunn M, Reese K, Malone CJ, White J, et al Spindle dynamics and the role of gamma-tubulin in early *Caenorhabditis elegans* embryos. Mol Biol Cell. 2001;12(6):1751–64 . 11408582 10.1091/mbc.12.6.1751 PMC37338

[104] Qin H, Powell-Coffman JA. The *Caenorhabditis elegans* aryl hydrocarbon receptor, AHR-1, regulates neuronal development. Developmental biology. 2004;270(1):64–75 . 15136141 10.1016/j.ydbio.2004.02.004

