# Supplementary material for: The Homeobox Genes of Caenorhabditis elegans and Insights into Their Spatio-Temporal Expression Dynamics during Embryogenesis
Source: PLoS One. 2015 May 29;10(5):e0126947. doi: 10.1371/journal.pone.0126947 (PMC4448998; doi:10.1371/journal.pone.0126947)
Supplement: S2 Fig — The three HDs are marked in red, the 15 zinc fingers are marked in green, cysteine and histidine residues are yellow. Two partial fingers are underlined, the second may form a finger using an Asp (D) residue. (PDF) [file pone.0126947.s002.pdf]

ZFH-2

MDVALLTMDDNNKPPTSSESTESRETSPPMMPDSAEIMRLLTDPSTAQMFANENTKCQLGRILAASGFDEASLSSSFPFDPTLGAFADISQFSSLRNSSK 100

TLKCPKCNWHYKYQETLEIHMKEKHNDVDVKCMFCAENRPHPKLARGETYSCGYKPYRCDLCRYSTTTKGNLSIHMQSDKHLHAVQELPNSIEFAANFAC 200

GAPVSRSSPIEESDGSLVCLICGIFATESIAEMMEHVEQDRSRTFQGDVTVLNGSFRCHLCPYNTTLKANFQLHTRTDKHLQKVQIDKSGDQIFKARIHN 300

FFLRNFEEVSNSPNSLLFLNLIFDIPDFKTMEDAAYACAGCEFTTKDVSDFEEHAKGHEEEAGASSQKACALCLEPTDDLEKHLVEQHRIAESAIEKFLL 400

TERTAKEASCSSSSEPSSEFAHRCSRCSMAFRSESQLQTHSLQHVFNTFHKCPTCGDSFDENTIVTHMLEHTKEECEMCSETFATKEAFLSHLNSARHLQ 500

QAKKQLENSLVDLNSQDVEKQRIYVCNVCKQSYPQAANLDVHMRSMTHQSRMSRLSELVATGELNGEKAVFEQPGIPAPTINSFIETTTNQSSLNDLMSL 600

LTRSESDEIREEVNGLQVMTQIKVYGESKITSLVPELAGKIDNIALFDDSRAAEVSKIDCSACGQQISGILALNLHYEESHSSKIPSDVLRKFGERLLAA 700

LEDGLSRENSVKNGSQSPLSNDDEPMEKKMRLENMLPEMDKNAAASQFAMFQQMMNCFPFMAPPGTSSGNFGFSPEMINQLMNPAAAAAAATAAAAAHAA 800

NNSPAKRARTRITDDQLKVLRQYFNINNSPSEAQIKEMSQKASLPEKVIKHWFRNTLFKERQRDKDSPYNFSIPPQMGIDLDIYEKTGETKVLSLSNEAP 900

KSELSSARATPTIPTPIPLIVEEKKPEPKSQTPSSSSSQLNLQAMLSQMQGSFFDASNFMSASVNPMTPSTPSCNTSSSGRRANRTRFTDFQLRTLQQFF 1000

DKQAYPKDDDLEALSKKLQLSPRVIVVWFQNARQKARKIYENQPNHESSDRFVRTPGSNFQCKRCSQVFQRYYELIQHQQKKCYKDDVAALASDNKSVEE 1100

SLTEEEKSQLLAQQQVAQLASTLELPKFQPAELLKMIGANSVTPSSSAASQKSSNDVLLKMCESIVGGSTPSTSSFHKLCIFCAQDFKDRSAMSEHMAQK 1200

HPQHMLLPNFDLDMMPDAGGAELLMDLKDSALDLSGSSVDYRDSISTSPSRSEDDVLTEALDDSAFAAFGLQLANSTSGSECRSPASNKRFRTHLTPMQV 1300

QMMKSVFNEYKTPSMAECELLGKEVGLHKRVVQVWFQNARAKERKTRGAVDEDSRSGELHCEICDKTFATRLSLQDHLFAEQHIEFLRNNLKREGVSELT 1400

TASVTECSPEKKAKLPNPLDLASFPFLNTFDIQIYGTPIAFLQVPDEIKKQITDDITAGKSRTTFSQDGFPLDKLKESLPEDEKSNLTVTQKDVGWACPA 1500

CTFVFQEEKKLREHQKAMCQSDKTLTLVQTHYSCKSCQSDFCLQSEYQFHLSMPPHTTTTTTTTP 1565

S2 Figure
