# Supplementary figures and images for: The Homeobox Genes of Caenorhabditis elegans and Insights into Their Spatio-Temporal Expression Dynamics during Embryogenesis
Source: PLoS One. 2015 May 29;10(5):e0126947. doi: 10.1371/journal.pone.0126947 (PMC4448998; doi:10.1371/journal.pone.0126947)

■

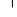

Supplement: S3 Fig — Expanded view of chromosome II (expanded from Fig 8), showing additional gene families, i.e. math, btb, fbxa, fbxb, and fbxc genes. Homeobox genes are marked in red. (PDF) [file pone.0126947.s003.pdf]
