# Supplementary material for: The Homeobox Genes of Caenorhabditis elegans and Insights into Their Spatio-Temporal Expression Dynamics during Embryogenesis
Source: PLoS One. 2015 May 29;10(5):e0126947. doi: 10.1371/journal.pone.0126947 (PMC4448998; doi:10.1371/journal.pone.0126947)
Supplement: S4 Fig — (A) Multiple sequence alignment of Caenorhabditis ORFs that share sequence similarity with the upstream region of CEH-86. A blastp search with CEH-86 retrieved three C. elegans ORFs, all located on cosmid C35E7, as well as related genes from other Caenorhabditis species. No similarity was found beyond Caenorhabditis. The sequence similarity starts at the N-terminus and extends to the HD of CEH-86. Furthermore, the newly identified ORFs extend their sequence similarity into the region that corresponds to the HD of CEH-86 and beyond. The sequence conservation is characterized by conserved cysteine residues, suggesting that multiple metal (usually zinc) binding fingers may be present. However, further analysis will be necessary to define the motif in depth, at present we refer to it as UCM (uncharacterized cysteine motif). The location of a HD attached to another protein coding region suggests that ceh-86 may have arisen by a duplication event (maybe from the ceh-84 homeobox), where a homeobox translocated into a UCM gene, or an N-terminal section of a UCM gene translocated upstream of a homeobox. (B) Neighbor-joining tree of the conserved region of the sequences in (A). Numbers show bootstrap values for 1000 trial runs. The tree shows that three clades exist that share orthologous genes in different Caenorhabditis species. A chromosomal cluster with multiple genes must have already existed before the divergence of C. elegans, C. briggsae, and C. remanei. It appears that CEH-86 does not seem to have a direct ortholog, supporting the notion of a recent duplication event. (PDF) [file pone.0126947.s004.pdf]

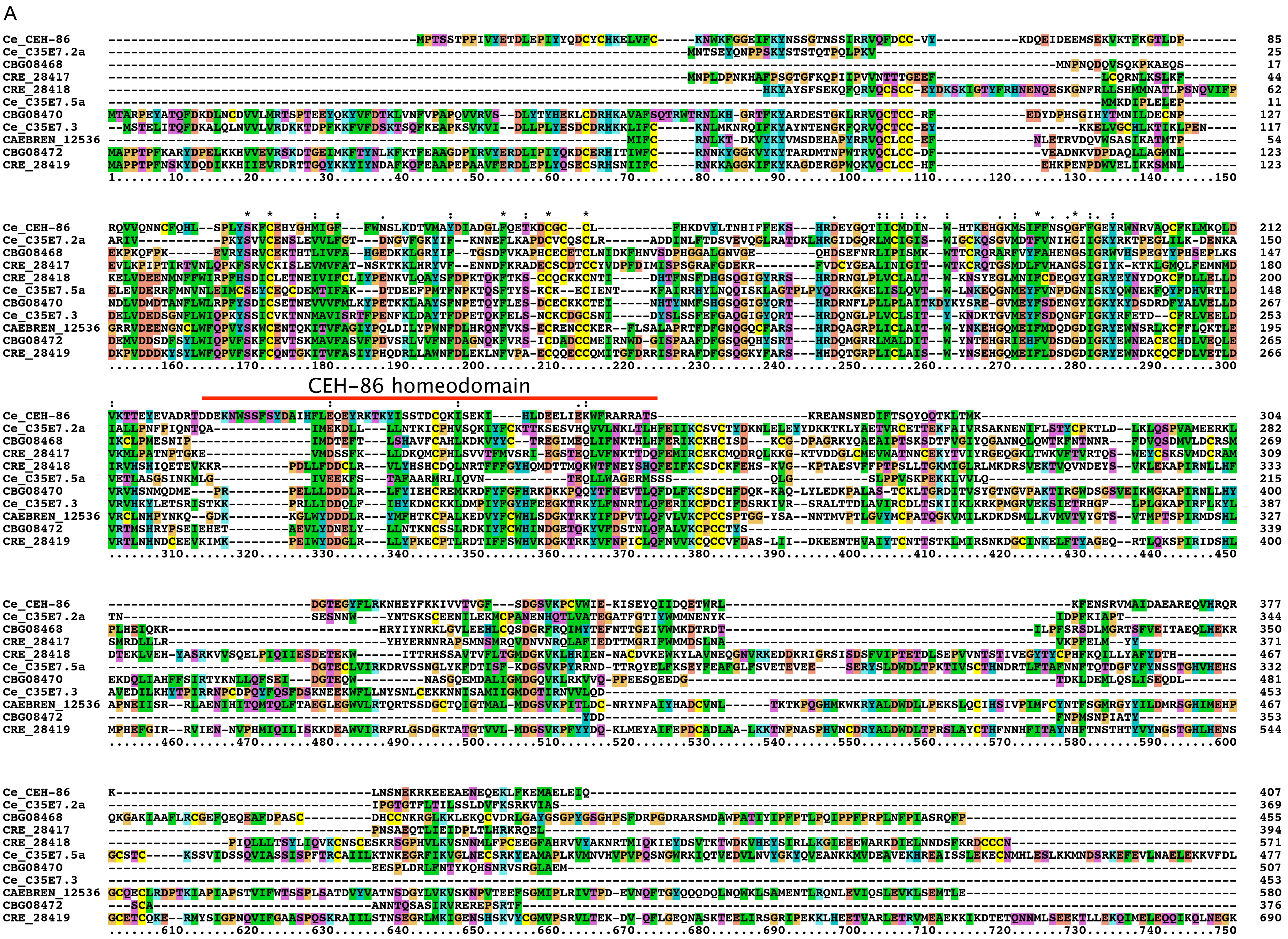
A
CEH-86 homeodomain
Ce_CEH-86
Ce_C35E7.2a
CBG08468
CRE_28417
CRE_28418
Ce_C35E7.5a
CBG08470
Ce_C35E7.3
CAEBREN_12536
CBG08472
CRE_28419

```
Ce_CEH-86     -------------------------------------NYRILPN----------------------------------------------------------------------------------------------------------  414
Ce_C35E7.2a   -------------------------------------KFTLF------------------------------------------------------------------------------------------------------------  374
CBG08468      -------------------------------------MPYVLPSYQPF------------------------------------------------------------------------------------------------------  466
CRE_28417     -------------------------------------YHALNPDYKNSTDY---------------------------------------------------------------------------------------------------  408
CRE_28418     -------------------------------------RPSITSVFNHF------------------------------------------------------------------------------------------------------  582
Ce_C35E7.5a   KKVLEMEQGKIAEMLKNKEREAAHKKQQEMINAPPVFDFNLFPKCCSFCPHIGQFVITAFDEVTKSTRSYIFSPKTAHFHYQSTCELCPRVICYSTVLPNWTIMPMQCERSKWPCLLLTNQEGRLIKVAFDAKINTFIAVKPSQVLEEIV  627
CBG08470      -------------------------KER-----PNEPEISIWKLY---------------------------------------------------------------------------------------------------------  522
Ce_C35E7.3    ------------------------------------------------------------------------------------------------------------------------------------------------------  453
CAEBREN_12536 --------AERERIRADVAENHIVNRDN-----NPLMDMDLSPMYRNYCPHTGHFYITSLNNRNQSIGHFVFNPTTGHFHQQDYCEACGDYTQGIKVQNFVTLMPAESPLTKFPFLVMTDDNGRLVKIGFCNEARGFVSQPASVIQPAVQ  717
CBG08472      -------------------------------------DHTQSGAY---------------------------------------------------------------------------------------------------------  384
CRE_28419     KEMEKEEVEEKEELPVEEEKVTIVQKHQ-----PLSTDHDLSPMYRNFCKHTGHFLITAFNRITETTSHYVFNPDTLLFHHQDYCSLCPSYENGVKVNSFVIIMPIQSMRCKFPYLIMTDETRRLVKIGFDSISMSFVLISPAVIQNQVE  835
              .......760.......770.......780.......790.......800.......810.......820.......830.......840.......850.......860.......870.......880.......890.......900

Ce_CEH-86     ------------------------------------------------------------------------------------------------------------------------------------------------------  414
Ce_C35E7.2a   ------------------------------------------------------------------------------------------------------------------------------------------------------  374
CBG08468      ------------------------------------------------------------------------------------------------------------------------------------------------------  466
CRE_28417     ------------------------------------------------------------------------------------------------------------------------------------------------------  408
CRE_28418     ------------------------------------------------------------------------------------------------------------------------------------------------------  582
Ce_C35E7.5a   EVQNSGMTTKKTLEIPSYDTTRP-----VLYPNFRVYCSHIKDVLINAYNSHAKSNAFYYYNKITGSFEEHVGCDHCSKSSLNHSIPSLQNVITSTSPYTDNAVILVSSKDRSLNILGFRSNNMGFANFPYNVVML--------------  758
CBG08470      ------------------------------------------------------------------------------------------------------------------------------------------------------  522
Ce_C35E7.3    ------------------------------------------------------------------------------------------------------------------------------------------------------  453
CAEBREN_12536 DIRMIPNSMRSIPTCIEAPVDQGGPYDCYLVPQYRVYCLHCKEFLLYGFHKELQANGYFMYNSQTGHLEEHPGCDSCEAINPRYDIDTMRSVMPVVSPYSKWPLMLTTNIYGELFPIAFKTENHRFNSMPRLKIVMMNERSPRVYPQELK  867
CBG08472      ------------------------------------------------------------------------------------------------------------------------------------------------------  384
CRE_28419     SITEITNSI-CYSHALEIP-NTPGPHDTDLCPKFRIFCTHISEFLIFAIDCFSGTEGFYLFNSATGHMEEHDDCPSCRTRSPVYIIEKMRSIMAISSPYSRWPIMLTTDKDGMFVPIGYDTSSQKFEKMHPLAIKLLKNKEEK-------  976
              .......910.......920.......930.......940.......950.......960.......970.......980.......990......1000......1010......1020......1030......1040......1050

Ce_CEH-86     ------------------------------------------------------------------------------------------------------------------------------------------------------  414
Ce_C35E7.2a   ---------------------------------------------------------------------------------------------------------------------------------ENVTPKDLIDH----------  385
CBG08468      ------------------------------------------------------------------------------------------------------------------QWVPQEPTRAPAAKREQPRKETVPKTEKEEKINDID  502
CRE_28417     ----------------------------------------------------------------------------------------------------------------------SSVVRRFTELNCDHCAQLASNALRSLNPGYSD  440
CRE_28418     ----------------------------------------------------------------------------------------------------------------------HAEQKRGQEIQKRKQMETEIDKELKLAVKEKE  614
Ce_C35E7.5a   ----------------------------------------------------------------------------------------------------------------------KEDLKGSEEPLTDLTVESLVEKVETLEVTKKV  790
CBG08470      ---------------------------------------------------------------------------------------------------------LQLGGFDESKQSGSGNGSDGIEEEAEVASDKDNDSDESSDESSED  567
Ce_C35E7.3    ------------------------------------------------------------------------------------------------------------------------------------------------------  453
CAEBREN_12536 YMMPVTQESAKVPPVIDEPVLDVNPPAPVPNVVPEVVPEETIPKLVKEFEKFEGSLVYNILPAADVPKLVKEFELLPANSYVGSKSPVPVRDLILQAQPVENKPEEQLEDTEDEDYSDEEDEEYSDEEDEEYSDEEEEDEEPKAEQTKAA 1017
CBG08472      ------------------------------------------------------------------------------------------------------------------------------------------------------  384
CRE_28419     ---------------------------------------------------------------------------------TEEKSEVNFVTDTVQMNPVENEDDDDECSDEEEEYSDEEEE--YSDEEEEYSDEEESGEDEEDEEEEVD 1043
              ......1060......1070......1080......1090......1100......1110......1120......1130......1140......1150......1160......1170......1180......1190......1200

Ce_CEH-86     ---------------------------------------------------------------------------------------------SSDKRLEEH-------------ELKTQSSTLPKPCAHAQAQAHAQAQAHAQ--AHET  456
Ce_C35E7.2a   ---------------------------------------------------------------------------------------------RCEDCENNVRPTDAWLESDKGKPTLRNQMHPDYHPTYKKNSEYEDAQEDF---DDIE  439
CBG08468      EILVQL---------------------------------------------------------------------------------------KEDDRLDQI-TAEVETPQVHLLKSQLQEIFQDKEPQEKPEYHSKDEEARAMIFAKHP  564
CRE_28417     AGAPHGLPTQGGAYQ------------------------------------------------------------------------------PYWNGTYAHQYMTVPVSTQYSLSYYMDPIAQSVPRQYSNNNTQAPVYQSMSYMNGNA  512
CRE_28418     EDVVSGVCTPQL---------------------------------------------------------------------------------QITDSNSVQKINKCEVSEEGKVITVSESESESDSESDSESDSESDSESDSE--SDSE  681
Ce_C35E7.5a   EVE------------------------------------------------------------------------------------------DDSDCLDED---------------EDEEEYEDEDEEESEEDEDEEEDEDSE--EDED  833
CBG08470      DV-------------------------------------------------------------------------------------------DSDDVSDEASTDEHKANRERIFHSDIWEQYRKSRCYDERAGSKQSGSGNGS--DGIE  624
Ce_C35E7.3    ---------------------------------------------------------------------------------------------KMEKELEQF----------------------------------------------ED  464
CAEBREN_12536 DVEPIVGPVDSLAKPEESDNSDEDYSDEDEEEEEYTDEEEEEEEEEEVEEAKPHSTFTFQMLQQTTQHLASTARAFTNFISAGLPSTSTEALEDDEEDCSDEE--EWCCSDEECS---CSDEECSDEECSDEEESDGECSDEDCS--DEEC 1160
CBG08472      ---------------------------------------------------------------------------------------------CHEAVADSH------------------------------------------------  393
CRE_28419     EDLLVGAQIINEIRHMQIAPNESEHDDEDCSDEEYYCSDEE-------------------------------------------------CSDCYEECSDEECSDEECSDEECSDEECSDEECSDEECSDEECSDEECSDEECS--DEED 1142
              ......1210......1220......1230......1240......1250......1260......1270......1280......1290......1300......1310......1320......1330......1340......1350

Ce_CEH-86     NDNWPKLMTELFRALLQGQSLTQQHMQRLVHLRTQPDFNRRSYAGHELTAME----------------------------MEALKQLLNLLNGKEMPTGKKEEVEGSGLTISEAHGTVGSPMLIEKKEELEGSLEHQPTMANEEIDQNLK  578
Ce_C35E7.2a   LKVFQKLCNMEDNLFKQIIDFEKPKEQFTAPPHPQMTHRVAQ--------------------------------PHYVPVEYFNHTISDLLRQISGLQHEQAMQNGQMQHMANEIRSIGNAVCSLHSSQLQQPPNSAASMFPYPIHPMPS  557
CBG08468      ETEVRELPSEFFTMNTVDSKLSKDKDDFHLPGLPSAKDIMTNYADMKK-----------------------NWPTEYQRLQDRCRVELEKIKKLEKLAELETKSIQAELVKRQQEENIARKCRLEKRLLEASKQFEETATGIKKNQEALA  691
CRE_28417     TTAYPYYCSYPRG--TNPSLGSVAHSYLANPMDPSCIRYPYQYTNR--------------------LGQHGIPTPNVPYHRYPKPRINPPVKRTEQPVAM--------------ASDKEQTPRIPTPEGIRGTGYTQPVEALSSLEELQR  626
CRE_28418     SDSESDSESDSSD--SESVDLEDEQTPKLDSLAPCLVKKEVTNFKKPIVTTADLTPLHSEFCIKLHKVFFFAFNNVTRGYGKYAPTSSKTLEEVESPIEYKPKKESEFNQKRTVVFEGGAEYLVDSNGRLLRVQYDKVKKILMRVEPEVV  829
Ce_C35E7.5a   EEEYEDEESEYED--EEEDEEEEEEEGKENNEEKKVQNEIKHYIKLVNSKAA-------------------IFDQLAIAFSLAKPETQSNFKEM--------FSNKKAFNERVGVEFDNNTWKKMQSVLAKVTREDDVESTTTALEPSDI  954
CBG08470      E---EDEVASDEG--NDSDESSDESSEDDVDSDDVSDEASTD-------------------------------EHKADRERIFHSDIWEQYRKSRCYDER---------AGSKQSGSGNGSDGIEEEDEVASDEGNDSDESSDESSEDDV  729
Ce_C35E7.3    ETSWSQDSDYSFL--NEIDRELTNTDKKNV------------------------------------------------------------------------------------------------------------------------  492
CAEBREN_12536 ECHWTDDDGEDAG--EDSGKSAEEKAAEEMAAEAKAAEERRKKAEEELRKVERVCNFEPTATTSTELNLHPDFSINQRKFEQARAELRRQEELEKSAQEQVFYLPAEVLEDPNYARVVEGDWRVPYYKDVSDEEVSDEEEMSDEEEVDDE 1308
CBG08472      ------------------------------------------------------------------------------------------------------------------------------------------------------  393
CRE_28419     ECCSDEECCCDEE--DECAEEAEDEDSEALKKEATNADKAEDNGNFVLVTEEDIERDVPEFISN--LYRFGIVDQKIQRIIDNSADMLEKYKKLKSFIDQMYDLSSKKTVEATEGDDVPNSSQNPTEQHEIRENNDVVEVAADDNVSADM 1288
              ......1360......1370......1380......1390......1400......1410......1420......1430......1440......1450......1460......1470......1480......1490......1500
```

```
Ce_CEH-86        EETTTKPEYRKKLRMS-------------KRKQNELLQKLWLKEKNTENTHAEKTNQEKKLTENAVQ--------------------------------EQLEQMMKMIQEERRAADSKNLKLEQMVLEMKDKLDAQETRIKELEEVIKS  683
Ce_C35E7.2a      TFPQQAPHAPNAPQMT-----------------------ANSMQSIAELILAQRGIAPRRERRLTEGPKK----------------------------YNRVIPPSPRIPSSTDKKEMLKKSNDRFDKLREEVSDVRTQLSGLEDTTFKK  656
CBG08468         KLYEMDPAIKTCVHNV---------------------------LAAGHQLAEKRELANEHLLKSIEKAKQ-----------------------------LQMHLYEPSEIMTHDDLLKYDARVKEVYFGESESNDEKKNGSGPSTSSDPE  785
CRE_28417        QLAALVPPTNYQHYPF------------------------------------RTEDPREKLENELKNLEE---------------------------MFRDNVTLCASSHGESSHAAVYAPRTKKTVRMDMDRSPMEKNQETEQPNLLVE  713
CRE_28418        KMLYEMDDVETGTFKTLYFLRFSLKNFEFSEQYQETEDFAGPTFVPEESERIHKFYSNANVLSTSIFMKS-----------------------SESVFGEDDDLDSEHSSTNSYYSDVGGVGNNVITRSHSSVYEGAFERSSENMNSVDD  956
Ce_C35E7.5a      EDNEDAEYTSTALEPS---------DFEDNCDFESTATAVEPLDFEESLSMDVTQQNESFEGVEHVEAED--------------------------DTQSTTTALEPSDIEDNCDVESTTTAVEPLDFEDDNSLDITQESDSLELMEHVE 1069
CBG08470         DSGEVSDEASTDEHKA---------NRENRDGPDDSETSIW-------KLYLQGGFDEPKQSNDSDAIEE------------------------IWDLLRKNGYSDEPAGSRQSGLENCADGIEEEDEVASDKGDVADEISDENAEDDVD  839
Ce_C35E7.3       ------------------------------------------------------------------------------------------------------------------------------------------------------  492
CAEBREN_12536    DEEEEATQPSTSSVDF---------EKMAKERGQTLEEFSD--------HLVSRFYSEVVASNDVSQPPPLRKLPEAPSDESEDSDLSRVESIGIRDISDTESEETDDSSDISRIESIGIQDISDTETALEPELDISELKIDEESDDSSD 1441
CBG08472         ------------------------------------------------------------------------------------------------------------------------------------------------------  393
CRE_28419        THQEETATTSNDIKCV---------ELIENDREDAESTATALENDQEDVESTTTALDHSDVEEDVEVIDEDQMSTATATDGEQDDVESTVTALDISDISDSAEFVNEHDIDDASSTATAIEGDQDDVESTATALDVSDISDSAEFINGHD 1429
                 ......1510......1520......1530......1540......1550......1560......1570......1580......1590......1600......1610......1620......1630......1640......1650

Ce_CEH-86        DKNAKKGEQ------------------------------------------------LTDPTEPQYTEAPNAEVGH--------------------------------------------------------KSNISSSIGNDVDLLEDD  729
Ce_C35E7.2a      PKKLHFLEPDEESESD-----------------------------------------ENGNDVDEFSEADDDDEDV------------------------------------------------------------DREMKVLVESLKNR  705
CBG08468         EEEEEEGSSEDSDISD-----------------------------------------EEESSDDSDLEQFD--------------------------------------------------------------------MKTIVDSLHNR  826
CRE_28417        QKKVEKKEDVPEEDSS-----------------------------------------DDDLEDEDYSSEDDDSDDM----------------------------------------------------------NDEEQLKTLLDSLQNR  764
CRE_28418        EKDEEKYTTISLDSSG-----------------------------------------EEEEDEDKEMDQSVEENDY-------------------------------------------------------------SRVGVIDETEEET 1004
Ce_C35E7.5a      AEDDTKSTTTALEPSD-----------------------------------------IEDNDLELVSDLSDDNSSI------------------------------------------SMISSTSTAIEFSIADDSGSSMGSDISYLDRD 1136
CBG08470         SGEVSDEASPDDDDDK-----------------------------------------ESTNTEDSYSDMSDADPEND-----------------------------------------------------EIGGPPESVIGRILGFF---  892
Ce_C35E7.3       ------------------------------------------------------------------------------------------------------------------------------------------------------  492
CAEBREN_12536    ISHVESIGIQDISDTE-----------TALEPELDVTQDSVSTEPEQELDLTDLVAPLNETSSDSESELSICESTA------------------------TALEPE---SEEIDEDNLSILSTTSTAKEFSIAHDSGSSAGSDISYLDDL 1553
CBG08472         ------------------------------------------------------------------------------------------------------------------------------------------------------  393
CRE_28419        IDDASSTATAIEGEQDDVESVTTALDHSDLEDDIEIIGEAPEEDPQETSSLELDITQDNEDTQSTTTALEISDSESDVSLVDAEPDVSDIEDREDTESVHTALEPDVATEDDIEMDNVSMLSSTSTAREPSIANDDESSIGSDISYLDTD 1579
                 ......1660......1670......1680......1690......1700......1710......1720......1730......1740......1750......1760......1770......1780......1790......1800

Ce_CEH-86        SGED------------------------------------------  733
Ce_C35E7.2a      VQ--------------------------------------------  707
CBG08468         MNLDLD----------------------------------------  832
CRE_28417        VNLDLD----------------------------------------  770
CRE_28418        VDAANVPPT---------------------------------CTIC 1017
Ce_C35E7.5a      MDFASDKESLVSESNYQDFMDPTIHASFTASQIAEADQNFPGCILS 1182
CBG08470         ----------------------------------------------  892
Ce_C35E7.3       ----------------------------------------------  492
CAEBREN_12536    SGMDGDEESFAEQEPSGAHPDDVPAQVDPINQQ---------CVIA 1590
CBG08472         ----------------------------------------------  393
CRE_28419        FSVNGDEPESEMVKSNYEDCNDQLGEAEPEGEERPVAQDANTCIIS 1625
                 ......1810......1820......1830......1840......
```

B

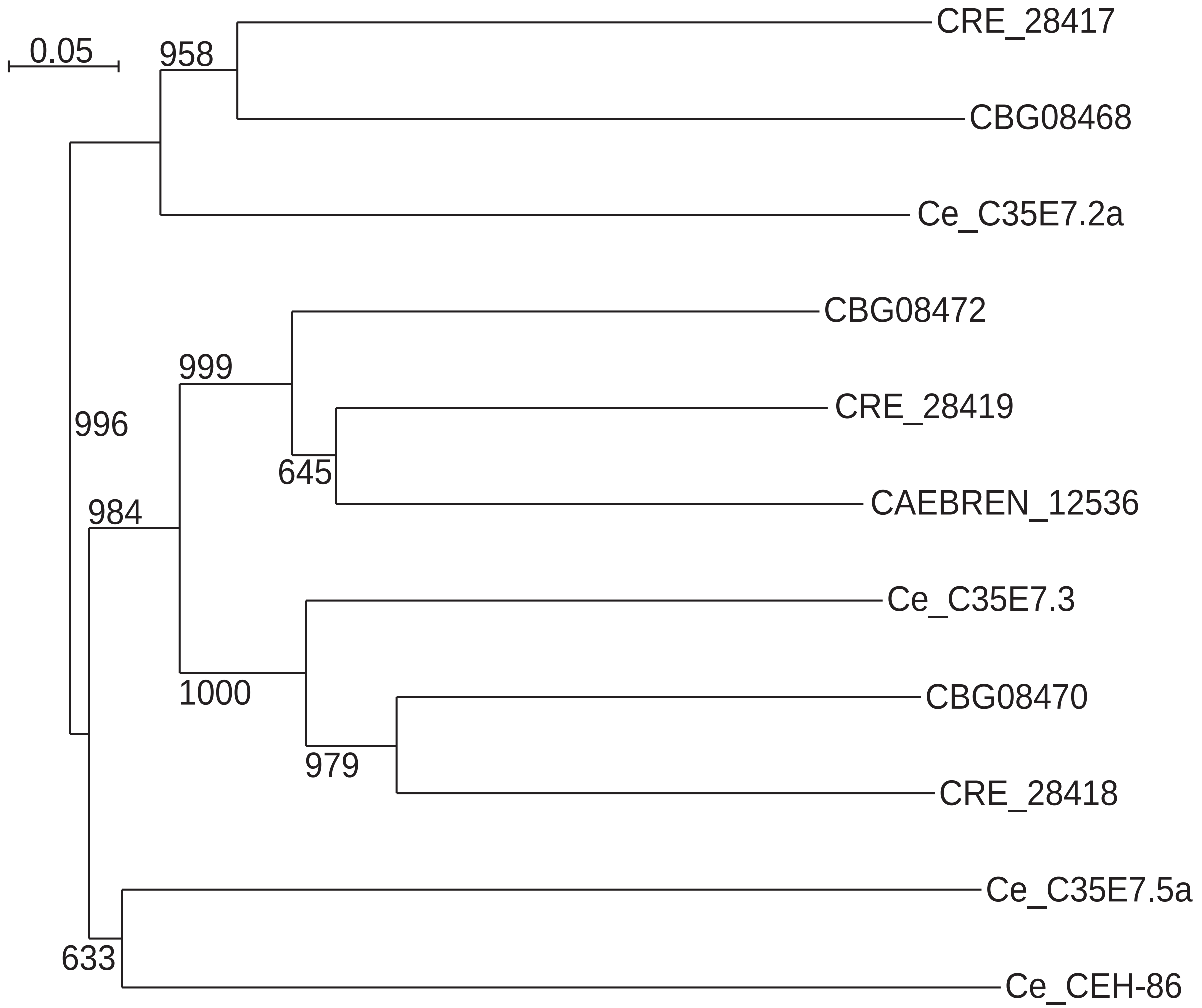
0.05
958
CRE_28417
CBG08468
Ce_C35E7.2a
996
999
CBG08472
CRE_28419
645
CAEBREN_12536
984
Ce_C35E7.3
1000
CBG08470
979
CRE_28418
Ce_C35E7.5a
633
Ce_CEH-86
