# Supplementary material for: The Homeobox Genes of Caenorhabditis elegans and Insights into Their Spatio-Temporal Expression Dynamics during Embryogenesis
Source: PLoS One. 2015 May 29;10(5):e0126947. doi: 10.1371/journal.pone.0126947 (PMC4448998; doi:10.1371/journal.pone.0126947)
Supplement: S1 Table — The third column gives TB strain designations, the fourth column are strains from other sources. TB strains were often derived from BC strains by integration. Some strains were obtained from CGC. Sources of additional strains: tbx-2::GFP [99], xbx-1::GFP [100], F55A4.3::GFP (+ elt-2::mCherry) [101], efn-4::GFP [102], pie-1::GFP::HIS-11 [103], mec-18::GFP [104]. (DOC) [file pone.0126947.s005.doc]

# S1 Table. Strain list

| **Gene name** | **ORF** | **TB strain name** | **Strain name** |
| --- | --- | --- | --- |
|  | Y32H12A.8 |  | BC10653 |
| *ceh-1* | F16H11.4 |  | UL2829 |
| *ceh-2* | C27A12.5 |  | PS3504 |
| *ceh-5* | C16C2.1 | TB2115 | BC10763 |
| *ceh-6* | K02B12.1 | TB2076 | - |
| *ceh-8* | ZK265.4 | TB2105 + | BC10756 |
| *ceh-10* | W03A3.1 |  | LE332 |
| *ceh-12* | F33D11.4 | TB2132 | - |
| *ceh-13* | R13A5.5 |  | FR317 |
| *ceh-14* | F46C8.5 | TB513 | BC14913 |
| *ceh-16* | C13G5.1 | TB2151, TB2163 | BC12234 |
| *ceh-19* | F20D12.6 |  | BC10766 |
| *ceh-20* | F31E3.1 | TB1801 |  |
| *ceh-22* | F29F11.5 |  | OK0640 |
| *ceh-23* | W03A3.1 |  | LE732 |
| *ceh-24* | F55B12.1 |  | PD4595 |
| *ceh-26* | K12H4.1 | TB1200 |  |
| *ceh-27* | F46F3.1 | TB2156 | BC12270 |
| *ceh-28* | K03A11.3 |  | BC12998 |
| *ceh-30* | C33D12.7 |  | “ceh-30” |
| *ceh-32* | W05E10.3 | TB1800 | - |
| *ceh-33* | C10G8.7 | TB2302 | BC11180 |
| *ceh-34* | C10G8.6 | TB3249 |  |
| *ceh-36* | C37E2.4 | TB2071, TB3196 | - |
| *ceh-37* | C37E2.5 |  | BC14784 |
| *ceh-40* | F17A2.5 | TB2162, FR783 | BC10765 |
| *ceh-41* | T26C11.5 |  | BC10782 |
| *ceh-43* | C28A5.4 | TB312 | - |
| *ceh-44* | Y54F10AM.4 |  | BC10759, BC12056 |
| *ceh-45* | ZK993.1 |  | BC10493 |
| *ceh-48* | C17H12.9 |  | BC10721 |
| *ceh-49* | F17A9.6 |  | BC15185 |
| *ceh-53* | C09G12.1 |  | BC10754A |
| *ceh-54* | T13C5.4 | TB2117 | - |
| *ceh-57* | C07E3.5 |  | BC15173 |
| *ceh-74* | ZC376.4 | TB2125, TB2118 | BC15162 |
| *ceh-81* | F45C12.3 | TB2144 | BC15188 |
| *ceh-83* | F45C12.15 | TB2124 | - |
| *ceh-84* | C40D2.4 |  | BC15331 |
| *ceh-85* | F59H6.6 | TB2153, TB2158 | - |
| *ceh-87* | F34D6.2 | TB2126 | - |
| *ceh-88* | C49C3.5 | TB2135, TB2145 | BC15177 |
| *ceh-89* | F28H6.2 | TB2154 | - |
| *ceh-93* | R04A9.5 | TB2140, TB2146 | BC15310 |
| *ceh-99* | T21B4.17 | TB2116 | - |
| *ceh-100* | Y38E10A.6 |  | BC15197 |
| *clh-4* | T06F4.2 | TB3197 | - |
| *cog-1* | R03C1.3 |  | PS3662 |
| *die-1* | C18D1.1 |  | OH3112 |
| *dsc-1* | C18B12.3 |  | BC10044 |
| *duxl-1* | ZC204.2 | TB2127 | BC15156 |
| *efn-4* | F56A11.3 | TB2098 | CZ1506 (juIs109) (transl.) |
| *egl-19* | C48A7.1 |  | BC12759 |
| *eyg-1* | Y53C12C.1 | TB2137 | BC10075 |
| *hbl-1* | F13D11.2 |  | BW1932 |
| *his-24* | M163.3 |  | EC100 |
| *his-72* | Y49E10.6 | TB2141 | - |
| *ifb-1* | F10C1.2 |  | CZ3464 |
| *ina-1* | F54G8.3 |  | NG2517 |
| *kel-3* | T27E9.4a |  | BC12850 |
| *lat-1* | B0457.1 |  | BC11510 |
| *lim-4* | ZC64.4 |  | PY2165 (oyIs35) |
| *lim-6* | K03E6.1 |  | OH812 |
| *lim-7* | C04F1.3 |  | DG1576 |
| *lin-11* | ZC247.3 | TB2155 | BC14491 |
| *lip-1* | C05B10.1 |  | AH142 |
| *mab-5* | C08C3.3 |  | CF453 |
| *mec-3* | F01D4.6 |  | “mec-3” |
| *mec-18* | C52B9.9 |  | TU2589 |
| *mig-13* | F43C9.4 |  | CF896 |
| *mls-2* | C39E6.4 |  | UP1619 |
| *nmy-2* | F20G4.3 |  | JJ1473 |
| *nob-1* | Y75B8A.2a | TB2160 | BC11887 |
| *npax-3* | R13.2 |  | BC16316 |
| *nuo-1* | C09H10.3 |  | LB21 |
| *pie-1* | Y49E10.14 |  | TY3558 |
| *polg-1* | Y57A10A.15 | TB2601 | - |
| *rgef-1* | F25B3.3 |  | NW1229 |
| *tbx-2* | F21H11.3 |  | OK0592 |
| *ttx-1* | Y113G7A.6 | TB2907 | - |
| *ttx-3* | C40H5.5 |  | OH99 |
| *unc-4* | F26C11.2 |  | JS5 |
| *unc-119* | M142.1 |  | OH441 |
| *vab-1* | M03A1.1 | TB2099 | - |
| *vab-3* | F14F3.1 |  | MT8457 |
| *xbx-1* | F02D8.3 |  | OE3914 |
| *zag-1* | F28F9.1 |  | BC13074 |
| *zfh-2* | ZC123.3 | TB2161 | - |
|  | F55A4.3 |  | OE3309 |
