# Supplementary material for: The Homeobox Genes of Caenorhabditis elegans and Insights into Their Spatio-Temporal Expression Dynamics during Embryogenesis
Source: PLoS One. 2015 May 29;10(5):e0126947. doi: 10.1371/journal.pone.0126947 (PMC4448998; doi:10.1371/journal.pone.0126947)
Supplement: S1 Fig — Sequences from Figs 3 and 4 were analyzed using LogoBar. Due to the fact that this alignment includes a number of divergent HDs, the sequence conservation is not as strong as in the original profile of 346 HDs [2, 71], although it still follows the same pattern with the strongest conservation in the DNA-binding helix 3. (PDF) [file pone.0126947.s001.pdf]

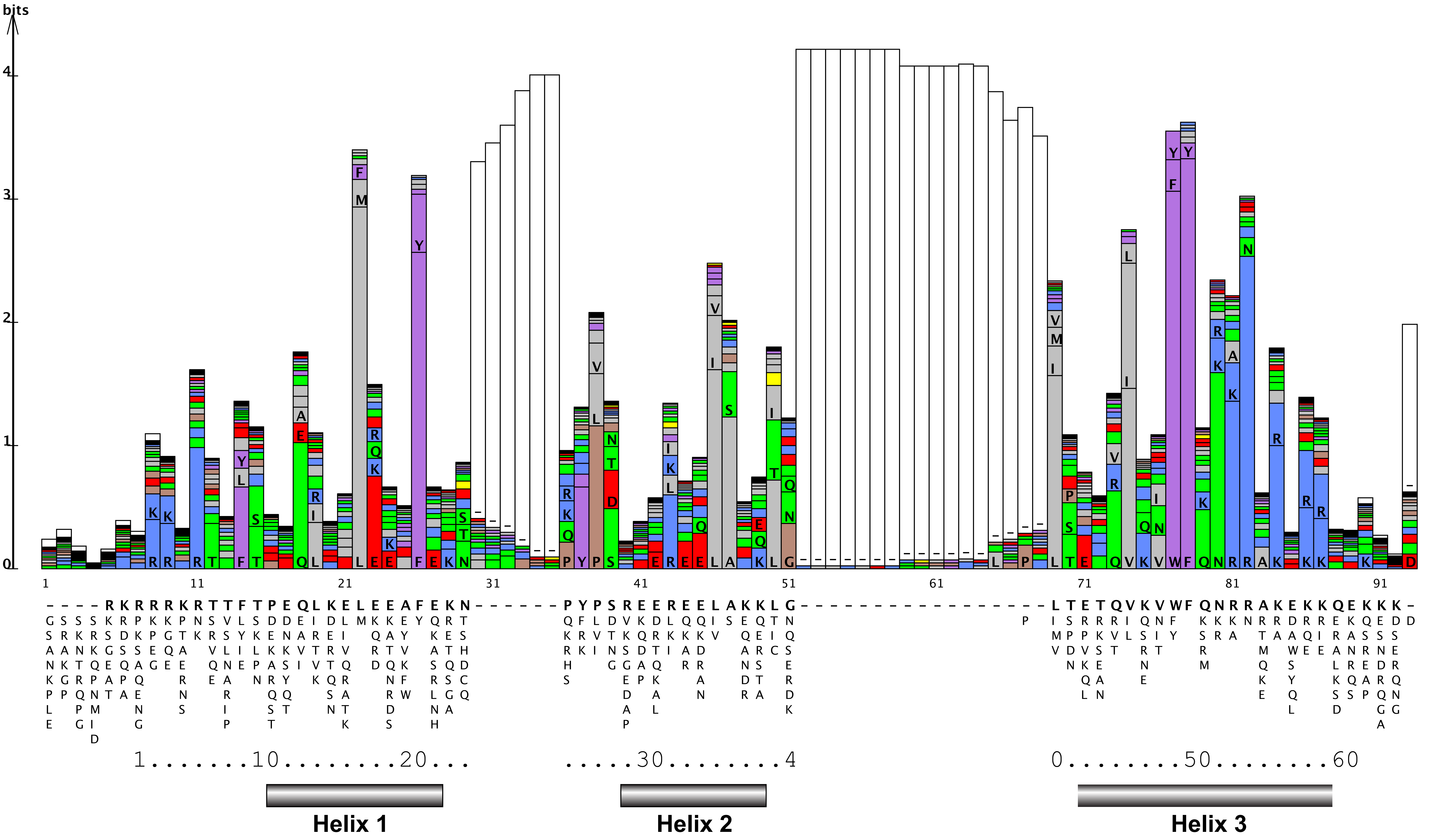

S1 Figure
