# Supplementary material for: The Homeobox Genes of Caenorhabditis elegans and Insights into Their Spatio-Temporal Expression Dynamics during Embryogenesis
Source: PLoS One. 2015 May 29;10(5):e0126947. doi: 10.1371/journal.pone.0126947 (PMC4448998; doi:10.1371/journal.pone.0126947)
Supplement: S1 Text — Extracted from WormBase release WS220 with BioMart. Microsoft Word document, zipped. (ZIP) [file pone.0126947.s006.zip › S1Text.doc]

**S1 Text. List of homeobox publications until WormBase release WS220**

| alr-1 | R08B4.2 | Melkman, T. and Sengupta, P. Regulation of chemosensory and GABAergic motor neuron development by the C. elegans Aristaless/Arx homolog alr-1. Development 132, 1935-1949. |
| --- | --- | --- |
|  |  | Tucker, M., Sieber, M., Morphew, M. and Han, M. The Caenorhabditis elegans aristaless orthologue, alr-1, is required for maintaining the functional and structural integrity of the amphid sensory organs. Mol Biol Cell 16, 4695-4704. |
|  |  | Okkema, P.G. and Krause, M. Transcriptional regulation. WormBook, 1-40. |
|  |  | Jovelin, R. Rapid sequence evolution of transcription factors controlling neuron differentiation in Caenorhabditis. Mol Biol Evol 26, 2373-2386. |
|  |  | Vavouri, T., Semple, J.I., Garcia-Verdugo, R. and Lehner, B. Intrinsic protein disorder and interaction promiscuity are widely associated with dosage sensitivity. Cell 138, 198-208. |
| ceh-1 | F16H11.4 | Hawkins, N.C. and McGhee, J.D. Homeobox containing genes in the nematode Caenorhabditis elegans. Nucleic Acids Res 18, 6101-6106. |
|  |  | Hubbard, E.J. and Greenstein, D. The Caenorhabditis elegans gonad: a test tube for cell and developmental biology. Dev Dyn 218, 2-22. |
|  |  | Hukema, R.K., Rademakers, S., Dekkers, M.P., Burghoorn, J. and Jansen, G. Antagonistic sensory cues generate gustatory plasticity in Caenorhabditis elegans. Embo J 25, 312-322. |
|  |  | Okkema, P.G. and Krause, M. Transcriptional regulation. WormBook, 1-40. |
|  |  | Jovelin, R. Rapid sequence evolution of transcription factors controlling neuron differentiation in Caenorhabditis. Mol Biol Evol 26, 2373-2386. |
| ceh-2 | C27A12.5 | Bürglin, T.R., Finney, M., Coulson, A. and Ruvkun, G. Caenorhabditis elegans has scores of homoeobox-containing genes. Nature 341, 239-243. |
|  |  | Schaller, D., Wittmann, C., Linning, R., Spicher, A., Muller, F. and Tobler, H. Cloning and expression in vitro of a gene encoding tRNAArgACG from the nematode Caenorhabditis elegans. Gene 97, 273-276. |
|  |  | Aspöck, G., Ruvkun, G. and Bürglin, T.R. The Caenorhabditis elegans ems class homeobox gene ceh-2 is required for M3 pharynx motoneuron function. Development 130, 3369-3378. |
|  |  | Inoue, T., Sherwood, D.R., Aspöck, G., Butler, J.A., Gupta, B.P., Kirouac, M., Wang, M., Lee, P.Y., Kramer, J.M., Hope, I. et al. Gene expression markers for Caenorhabditis elegans vulval cells. Mech Dev 119 Suppl 1, S203-209. |
|  |  | Inoue, T., Wang, M., Ririe, T.O., Fernandes, J.S. and Sternberg, P.W. Transcriptional network underlying Caenorhabditis elegans vulval development. Proc Natl Acad Sci U S A 102, 4972-4977. |
|  |  | Hobert, O. Specification of the nervous system. WormBook, 1-19. |
|  |  | Okkema, P.G. and Krause, M. Transcriptional regulation. WormBook, 1-40. |
|  |  | Mango, S.E. The C. elegans pharynx: a model for organogenesis. WormBook, 1-26. |
|  |  | Fernandes, J.S. and Sternberg, P.W. The tailless ortholog nhr-67 regulates patterning of gene expression and morphogenesis in the C. elegans vulva. PLoS Genet 3, e69. |
|  |  | Rauthan, M., Morck, C. and Pilon, M. The C. elegans M3 neuron guides the growth cone of its sister cell M2 via the Kruppel-like zinc finger protein MNM-2. Dev Biol 311, 185-199. |
|  |  | Van Hoffelen, S. and Herman, M.A. Analysis of Wnt signaling during Caenorhabditis elegans postembryonic development. Methods Mol Biol 469, 87-102. |
|  |  | Jovelin, R. Rapid sequence evolution of transcription factors controlling neuron differentiation in Caenorhabditis. Mol Biol Evol 26, 2373-2386. |
| ceh-5 | C16C2.1 | Bürglin, T.R., Finney, M., Coulson, A. and Ruvkun, G. Caenorhabditis elegans has scores of homoeobox-containing genes. Nature 341, 239-243. |
|  |  | Okkema, P.G. and Krause, M. Transcriptional regulation. WormBook, 1-40. |
| ceh-6 | K02B12.1 | Bürglin, T.R., Finney, M., Coulson, A. and Ruvkun, G. Caenorhabditis elegans has scores of homoeobox-containing genes. Nature 341, 239-243. |
|  |  | Zwaal, R.R., Broeks, A., van Meurs, J., Groenen, J.T. and Plasterk, R.H. Target-selected gene inactivation in Caenorhabditis elegans by using a frozen transposon insertion mutant bank. Proc Natl Acad Sci U S A 90, 7431-7435. |
|  |  | Moss, E.G., Lee, R.C. and Ambros, V. The cold shock domain protein LIN-28 controls developmental timing in C. elegans and is regulated by the lin-4 RNA. Cell 88, 637-646. |
|  |  | Fraser, A.G., Kamath, R.S., Zipperlen, P., Martinez-Campos, M., Sohrmann, M. and Ahringer, J. Functional genomic analysis of C. elegans chromosome I by systematic RNA interference. Nature 408, 325-330. |
|  |  | Kamath, R.S., Martinez-Campos, M., Zipperlen, P., Fraser, A.G. and Ahringer, J. Effectiveness of specific RNA-mediated interference through ingested double-stranded RNA in Caenorhabditis elegans. Genome Biol 2, RESEARCH0002. |
|  |  | Bürglin, T.R. and Ruvkun, G. Regulation of ectodermal and excretory function by the C. elegans POU homeobox gene ceh-6. Development 128, 779-790. |
|  |  | Sewell, S.T., Zhang, G., Uttam, A. and Chamberlin, H.M. Developmental patterning in the Caenorhabditis elegans hindgut. Dev Biol 262, 88-93. |
|  |  | Nelson, C.E., Hersh, B.M. and Carroll, S.B. The regulatory content of intergenic DNA shapes genome architecture. Genome Biol 5, R25. |
|  |  | Okkema, P.G. and Krause, M. Transcriptional regulation. WormBook, 1-40. |
|  |  | Mah, A.K., Armstrong, K.R., Chew, D.S., Chu, J.S., Tu, D.K., Johnsen, R.C., Chen, N., Chamberlin, H.M. and Baillie, D.L. Transcriptional regulation of AQP-8, a Caenorhabditis elegans aquaporin exclusively expressed in the excretory system, by the POU homeobox transcription factor CEH-6. J Biol Chem 282, 28074-28086. |
|  |  | Armstrong, K.R. and Chamberlin, H.M. Coordinate regulation of gene expression in the C. elegans excretory cell by the POU domain protein CEH-6. Mol Genet Genomics 283, 73-87. |
|  |  | Mah, A.K., Tu, D.K., Johnsen, R.C., Chu, J.S., Chen, N. and Baillie, D.L. Characterization of the octamer, a cis-regulatory element that modulates excretory cell gene-expression in Caenorhabditis elegans. BMC Mol Biol 11, 19. |
| ceh-7 | C34C6.8 | Bürglin, T.R., Finney, M., Coulson, A. and Ruvkun, G. Caenorhabditis elegans has scores of homoeobox-containing genes. Nature 341, 239-243. |
|  |  | Kagoshima, H., Cassata, G. and Bürglin, T.R. A Caenorhabditis elegans homeobox gene expressed in the male tail, a link between pattern formation and sexual dimorphism? Dev Genes Evol 209, 59-62. |
|  |  | Thoemke, K., Yi, W., Ross, J.M., Kim, S., Reinke, V. and Zarkower, D. Genome-wide analysis of sex-enriched gene expression during C. elegans larval development. Dev Biol 284, 500-508. |
|  |  | Okkema, P.G. and Krause, M. Transcriptional regulation. WormBook, 1-40. |
|  |  | Broitman-Maduro, G., Owraghi, M., Hung, W.W., Kuntz, S., Sternberg, P.W. and Maduro, M.F. The NK-2 class homeodomain factor CEH-51 and the T-box factor TBX-35 have overlapping function in C. elegans mesoderm development. Development 136, 2735-2746. |
| ceh-8 | ZK265.4 | Bürglin, T.R., Finney, M., Coulson, A. and Ruvkun, G. Caenorhabditis elegans has scores of homoeobox-containing genes. Nature 341, 239-243. |
|  |  | Pujol, N., Torregrossa, P., Ewbank, J.J. and Brunet, J.F. The homeodomain protein CePHOX2/CEH-17 controls antero-posterior axonal growth in C. elegans. Development 127, 3361-3371. |
|  |  | Okkema, P.G. and Krause, M. Transcriptional regulation. WormBook, 1-40. |
| ceh-9 | Y65B4BR.9 | Hawkins, N.C. and McGhee, J.D. Homeobox containing genes in the nematode Caenorhabditis elegans. Nucleic Acids Res 18, 6101-6106. |
|  |  | Okkema, P.G. and Krause, M. Transcriptional regulation. WormBook, 1-40. |
| ceh-10 | W03A3.1 | Manser, J. and Wood, W.B. Mutations affecting embryonic cell migrations in Caenorhabditis elegans. Dev Genet 11, 49-64. |
|  |  | Hawkins, N.C. and McGhee, J.D. Homeobox containing genes in the nematode Caenorhabditis elegans. Nucleic Acids Res 18, 6101-6106. |
|  |  | Svendsen, P.C. and McGhee, J.D. The C. elegans neuronally expressed homeobox gene ceh-10 is closely related to genes expressed in the vertebrate eye. Development 121, 1253-1262. |
|  |  | Forrester, W.C., Perens, E., Zallen, J.A. and Garriga, G. Identification of Caenorhabditis elegans genes required for neuronal differentiation and migration. Genetics 148, 151-165. |
|  |  | Montell, D.J. The genetics of cell migration in Drosophila melanogaster and Caenorhabditis elegans development. Development 126, 3035-3046. |
|  |  | Pujol, N., Torregrossa, P., Ewbank, J.J. and Brunet, J.F. The homeodomain protein CePHOX2/CEH-17 controls antero-posterior axonal growth in C. elegans. Development 127, 3361-3371. |
|  |  | Altun-Gultekin, Z., Andachi, Y., Tsalik, E.L., Pilgrim, D., Kohara, Y. and Hobert, O. A regulatory cascade of three homeobox genes, ceh-10, ttx-3 and ceh-23, controls cell fate specification of a defined interneuron class in C. elegans. Development 128, 1951-1969. |
|  |  | Tsalik, E.L., Niacaris, T., Wenick, A.S., Pau, K., Avery, L. and Hobert, O. LIM homeobox gene-dependent expression of biogenic amine receptors in restricted regions of the C. elegans nervous system. Dev Biol 263, 81-102. |
|  |  | Huang, X., Powell-Coffman, J.A. and Jin, Y. The AHR-1 aryl hydrocarbon receptor and its co-factor the AHA-1 aryl hydrocarbon receptor nuclear translocator specify GABAergic neuron cell fate in C. elegans. Development 131, 819-828. |
|  |  | Wenick, A.S. and Hobert, O. Genomic cis-regulatory architecture and trans-acting regulators of a single interneuron-specific gene battery in C. elegans. Dev Cell 6, 757-770. |
|  |  | Hobert, O. Specification of the nervous system. WormBook, 1-19. |
|  |  | Okkema, P.G. and Krause, M. Transcriptional regulation. WormBook, 1-40. |
|  |  | Muzzini, D.M., Plevani, P., Boulton, S.J., Cassata, G. and Marini, F. Caenorhabditis elegans POLQ-1 and HEL-308 function in two distinct DNA interstrand cross-link repair pathways. DNA Repair (Amst) 7, 941-950. |
|  |  | Ziel, J.W., Hagedorn, E.J., Audhya, A. and Sherwood, D.R. UNC-6 (netrin) orients the invasive membrane of the anchor cell in C. elegans. Nat Cell Biol 11, 183-189. |
|  |  | Bertrand, V. and Hobert, O. Linking asymmetric cell division to the terminal differentiation program of postmitotic neurons in C. elegans. Dev Cell 16, 563-575. |
|  |  | Jovelin, R. Rapid sequence evolution of transcription factors controlling neuron differentiation in Caenorhabditis. Mol Biol Evol 26, 2373-2386. |
|  |  | Van Buskirk, C. and Sternberg, P.W. Paired and LIM class homeodomain proteins coordinate differentiation of the C. elegans ALA neuron. Development 137, 2065-2074. |
| ceh-12 | F33D11.4 | Bürglin, T.R., Finney, M., Coulson, A. and Ruvkun, G. Caenorhabditis elegans has scores of homoeobox-containing genes. Nature 341, 239-243. |
|  |  | Schaller, D., Wittmann, C., Spicher, A., Muller, F. and Tobler, H. Cloning and analysis of three new homeobox genes from the nematode Caenorhabditis elegans. Nucleic Acids Res 18, 2033-2036. |
|  |  | Schaller, D., Wittmann, C., Linning, R., Spicher, A., Muller, F. and Tobler, H. Cloning and expression in vitro of a gene encoding tRNAArgACG from the nematode Caenorhabditis elegans. Gene 97, 273-276. |
|  |  | Okkema, P.G. and Krause, M. Transcriptional regulation. WormBook, 1-40. |
|  |  | Von Stetina, S.E., Fox, R.M., Watkins, K.L., Starich, T.A., Shaw, J.E. and Miller, D.M., 3rd UNC-4 represses CEH-12/HB9 to specify synaptic inputs to VA motor neurons in C. elegans. Genes Dev 21, 332-346. |
| ceh-13 | R13A5.5 | Schaller, D., Wittmann, C., Spicher, A., Muller, F. and Tobler, H. Cloning and analysis of three new homeobox genes from the nematode Caenorhabditis elegans. Nucleic Acids Res 18, 2033-2036. |
|  |  | Bürglin, T.R., Ruvkun, G., Coulson, A., Hawkins, N.C., McGhee, J.D., Schaller, D., Wittmann, C., Müller, F. and Waterston, R.H. Nematode homeobox cluster. Nature 351, 703. |
|  |  | Naito, M., Kohara, Y. and Kurosawa, Y. Identification of a homeobox-containing gene located between lin-45 and unc-24 on chromosome IV in the nematode Caenorhabditis elegans. Nucleic Acids Res 20, 2967-2969. |
|  |  | Wang, B.B., Muller-Immergluck, M.M., Austin, J., Robinson, N.T., Chisholm, A. and Kenyon, C. A homeotic gene cluster patterns the anteroposterior body axis of C. elegans. Cell 74, 29-42. |
|  |  | Zwaal, R.R., Broeks, A., van Meurs, J., Groenen, J.T. and Plasterk, R.H. Target-selected gene inactivation in Caenorhabditis elegans by using a frozen transposon insertion mutant bank. Proc Natl Acad Sci U S A 90, 7431-7435. |
|  |  | Bürglin, T.R. and Ruvkun, G. The Caenorhabditis elegans homeobox gene cluster. Curr Opin Genet Dev 3, 615-620. |
|  |  | Wood, W.B. and Edgar, L.G. Patterning in the C. elegans embryo. Trends Genet 10, 49-54. |
|  |  | Wilson, R., Ainscough, R., Anderson, K., Baynes, C., Berks, M., Bonfield, J., Burton, J., Connell, M., Copsey, T., Cooper, J. et al. 2.2 Mb of contiguous nucleotide sequence from chromosome III of C. elegans. Nature 368, 32-38. |
|  |  | Duggan, A. and Chalfie, M. Control of neuronal development in Caenorhabditis elegans. Curr Opin Neurobiol 5, 6-9. |
|  |  | Wittmann, C., Bossinger, O., Goldstein, B., Fleischmann, M., Kohler, R., Brunschwig, K., Tobler, H. and Muller, F. The expression of the C. elegans labial-like Hox gene ceh-13 during early embryogenesis relies on cell fate and on anteroposterior cell polarity. Development 124, 4193-4200. |
|  |  | Kenyon, C.J., Austin, J., Costa, M., Cowing, D.W., Harris, J.M., Honigberg, L., Hunter, C.P., Maloof, J.N., Muller-Immergluck, M.M., Salser, S.J. et al. The dance of the Hox genes: patterning the anteroposterior body axis of Caenorhabditis elegans. Cold Spring Harb Symp Quant Biol 62, 293-305. |
|  |  | Newman-Smith, E.D. and Rothman, J.H. The maternal-to-zygotic transition in embryonic patterning of Caenorhabditis elegans. Curr Opin Genet Dev 8, 472-480. |
|  |  | Ruvkun, G. and Hobert, O. The taxonomy of developmental control in Caenorhabditis elegans. Science 282, 2033-2041. |
|  |  | Schroeder, D.F. and McGhee, J.D. Anterior-posterior patterning within the Caenorhabditis elegans endoderm. Development 125, 4877-4887. |
|  |  | Brunschwig, K., Wittmann, C., Schnabel, R., Bürglin, T.R., Tobler, H. and Müller, F. Anterior organization of the Caenorhabditis elegans embryo by the labial-like Hox gene ceh-13. Development 126, 1537-1546. |
|  |  | Kwon, J.Y., Park, J.M., Gim, B.S., Han, S.J., Lee, J. and Kim, Y.J. Caenorhabditis elegans mediator complexes are required for developmental-specific transcriptional activation. Proc Natl Acad Sci U S A 96, 14990-14995. |
|  |  | Hoier, E.F., Mohler, W.A., Kim, S.K. and Hajnal, A. The Caenorhabditis elegans APC-related gene apr-1 is required for epithelial cell migration and Hox gene expression. Genes Dev 14, 874-886. |
|  |  | Van Auken, K., Weaver, D.C., Edgar, L.G. and Wood, W.B. Caenorhabditis elegans embryonic axial patterning requires two recently discovered posterior-group Hox genes. Proc Natl Acad Sci U S A 97, 4499-4503. |
|  |  | Shemer, G. and Podbilewicz, B. Fusomorphogenesis: cell fusion in organ formation. Dev Dyn 218, 30-51. |
|  |  | Hermann, G.J., Leung, B. and Priess, J.R. Left-right asymmetry in C. elegans intestine organogenesis involves a LIN-12/Notch signaling pathway. Development 127, 3429-3440. |
|  |  | Liu, J. and Fire, A. Overlapping roles of two Hox genes and the exd ortholog ceh-20 in diversification of the C. elegans postembryonic mesoderm. Development 127, 5179-5190. |
|  |  | Streit, A., Kohler, R., Marty, T., Belfiore, M., Takacs-Vellai, K., Vigano, M.A., Schnabel, R., Affolter, M. and Muller, F. Conserved regulation of the Caenorhabditis elegans labial/Hox1 gene ceh-13. Dev Biol 242, 96-108. |
|  |  | Aboobaker, A.A. and Blaxter, M.L. Hox Gene Loss during Dynamic Evolution of the Nematode Cluster. Curr Biol 13, 37-40. |
|  |  | Stoyanov, C.N., Fleischmann, M., Suzuki, Y., Tapparel, N., Gautron, F., Streit, A., Wood, W.B. and Muller, F. Expression of the C. elegans labial orthologue ceh-13 during male tail morphogenesis. Dev Biol 259, 137-149. |
|  |  | Zhang, H., Azevedo, R.B., Lints, R., Doyle, C., Teng, Y., Haber, D. and Emmons, S.W. Global regulation of Hox gene expression in C. elegans by a SAM domain protein. Dev Cell 4, 903-915. |
|  |  | Cutter, A.D. and Payseur, B.A. Rates of deleterious mutation and the evolution of sex in Caenorhabditis. J Evol Biol 16, 812-822. |
|  |  | Aboobaker, A. and Blaxter, M. Hox gene evolution in nematodes: novelty conserved. Curr Opin Genet Dev 13, 593-598. |
|  |  | Landmann, F., Quintin, S. and Labouesse, M. Multiple regulatory elements with spatially and temporally distinct activities control the expression of the epithelial differentiation gene lin-26 in C. elegans. Dev Biol 265, 478-490. |
|  |  | Botas, J. Control of morphogenesis and differentiation by HOM/Hox genes. Curr Opin Cell Biol 5, 1015-1022. |
|  |  | Belfiore, M., Pugnale, P., Saudan, Z. and Puoti, A. Roles of the C. elegans cyclophilin-like protein MOG-6 in MEP-1 binding and germline fates. Development 131, 2935-2945. |
|  |  | Podbilewicz, B. Cell fusion. WormBook, 1-32. |
|  |  | Herman, M.A. Hermaphrodite cell-fate specification. WormBook, 1-16. |
|  |  | Savage-Dunn, C. TGF-beta signaling. WormBook, 1-12. |
|  |  | Okkema, P.G. and Krause, M. Transcriptional regulation. WormBook, 1-40. |
|  |  | Deplancke, B., Mukhopadhyay, A., Ao, W., Elewa, A.M., Grove, C.A., Martinez, N.J., Sequerra, R., Doucette-Stamm, L., Reece-Hoyes, J.S., Hope, I.A. et al. A gene-centered C. elegans protein-DNA interaction network. Cell 125, 1193-1205. |
|  |  | Vastenhouw, N.L., Brunschwig, K., Okihara, K.L., Muller, F., Tijsterman, M. and Plasterk, R.H. Gene expression: long-term gene silencing by RNAi. Nature 442, 882. |
|  |  | Minasaki, R. and Streit, A. MEL-47, a novel protein required for early cell divisions in the nematode Caenorhabditis elegans. Mol Genet Genomics 277, 315-328. |
|  |  | Schmitz, C., Kinge, P. and Hutter, H. Axon guidance genes identified in a large-scale RNAi screen using the RNAi-hypersensitive Caenorhabditis elegans strain nre-1(hd20) lin-15b(hd126). Proc Natl Acad Sci U S A 104, 834-839. |
|  |  | Jiang, Y., Shi, H., Amin, N.M., Sultan, I. and Liu, J. Mesodermal expression of the C. elegans HMX homolog mls-2 requires the PBC homolog CEH-20. Mech Dev 125, 451-461. |
|  |  | Kuntz, S.G., Schwarz, E.M., DeModena, J.A., De Buysscher, T., Trout, D., Shizuya, H., Sternberg, P.W. and Wold, B.J. Multigenome DNA sequence conservation identifies Hox cis-regulatory elements. Genome Res 18, 1955-1968. |
|  |  | Seah, A. and Sternberg, P.W. The roles of EGF and Wnt signaling during patterning of the C. elegans Bgamma/delta Equivalence Group. BMC Dev Biol 9, 74. |
|  |  | Hueber, S.D., Weiller, G.F., Djordjevic, M.A. and Frickey, T. Improving Hox protein classification across the major model organisms. PLoS One 5, e10820. |
|  |  | Tihanyi, B., Vellai, T., Regos, A., Ari, E., Muller, F. and Takacs-Vellai, K. The C. elegans Hox gene ceh-13 regulates cell migration and fusion in a non-colinear way. Implications for the early evolution of Hox clusters. BMC Dev Biol 10, 78. |
| ceh-14 | F46C8.5 | Bürglin, T.R., Finney, M., Coulson, A. and Ruvkun, G. Caenorhabditis elegans has scores of homoeobox-containing genes. Nature 341, 239-243. |
|  |  | Freyd, G., Kim, S.K. and Horvitz, H.R. Novel cysteine-rich motif and homeodomain in the product of the Caenorhabditis elegans cell lineage gene lin-11. Nature 344, 876-879. |
|  |  | Shibatohge, M., Kariya, K., Liao, Y., Hu, C.D., Watari, Y., Goshima, M., Shima, F. and Kataoka, T. Identification of PLC210, a Caenorhabditis elegans phospholipase C, as a putative effector of Ras. J Biol Chem 273, 6218-6222. |
|  |  | Hobert, O., Tessmar, K. and Ruvkun, G. The Caenorhabditis elegans lim-6 LIM homeobox gene regulates neurite outgrowth and function of particular GABAergic neurons. Development 126, 1547-1562. |
|  |  | Sagasti, A., Hobert, O., Troemel, E.R., Ruvkun, G. and Bargmann, C.I. Alternative olfactory neuron fates are specified by the LIM homeobox gene lim-4. Genes Dev 13, 1794-1806. |
|  |  | Cassata, G., Kagoshima, H., Andachi, Y., Kohara, Y., Dürrenberger, M.B., Hall, D.H. and Bürglin, T.R. The LIM homeobox gene ceh-14 confers thermosensory function to the AFD neurons in Caenorhabditis elegans. Neuron 25, 587-597. |
|  |  | Cassata, G., Kuhn, F., Witmer, A., Kirchhofer, R. and Bürglin, T.R. A steep thermal gradient thermotaxis assay for the nematode Caenorhabditis elegans. Genesis 27, 141-144. |
|  |  | Cassata, G., Röhrig, S., Kuhn, F., Hauri, H.P., Baumeister, R. and Bürglin, T.R. The Caenorhabditis elegans Ldb/NLI/Clim orthologue ldb-1 is required for neuronal function. Dev Biol 226, 45-56. |
|  |  | Kagoshima, H., Sommer, R., Reinhart, B.J., Cassata, G., Ruvkun, G. and Bürglin, T.R. Graded expression of ceh-14 reporters in the hypodermis is induced by a gonadal signal. Dev Genes Evol 210, 564-569. |
|  |  | Aurelio, O., Boulin, T. and Hobert, O. Identification of spatial and temporal cues that regulate postembryonic expression of axon maintenance factors in the C. elegans ventral nerve cord. Development 130, 599-610. |
|  |  | Melkman, T. and Sengupta, P. The worm's sense of smell. Development of functional diversity in the chemosensory system of Caenorhabditis elegans. Dev Biol 265, 302-319. |
|  |  | Bando, T., Ikeda, T. and Kagawa, H. The homeoproteins MAB-18 and CEH-14 insulate the dauer collagen gene col-43 from activation by the adjacent promoter of the Spermatheca gene sth-1 in Caenorhabditis elegans. J Mol Biol 348, 101-112. |
|  |  | Hobert, O. Specification of the nervous system. WormBook, 1-19. |
|  |  | Okkema, P.G. and Krause, M. Transcriptional regulation. WormBook, 1-40. |
|  |  | Bürglin, T.R. Evolution of hedgehog and hedgehog-related genes, their origin from Hog proteins in ancestral eukaryotes and discovery of a novel Hint motif. BMC Genomics 9, 127. |
|  |  | Jovelin, R. Rapid sequence evolution of transcription factors controlling neuron differentiation in Caenorhabditis. Mol Biol Evol 26, 2373-2386. |
|  |  | Vavouri, T., Semple, J.I., Garcia-Verdugo, R. and Lehner, B. Intrinsic protein disorder and interaction promiscuity are widely associated with dosage sensitivity. Cell 138, 198-208. |
|  |  | Van Buskirk, C. and Sternberg, P.W. Paired and LIM class homeodomain proteins coordinate differentiation of the C. elegans ALA neuron. Development 137, 2065-2074. |
| ceh-16 | C13G5.1 | Wilson, R., Ainscough, R., Anderson, K., Baynes, C., Berks, M., Bonfield, J., Burton, J., Connell, M., Copsey, T., Cooper, J. et al. 2.2 Mb of contiguous nucleotide sequence from chromosome III of C. elegans. Nature 368, 32-38. |
|  |  | Nelson, C.E., Hersh, B.M. and Carroll, S.B. The regulatory content of intergenic DNA shapes genome architecture. Genome Biol 5, R25. |
|  |  | Cassata, G., Shemer, G., Morandi, P., Donhauser, R., Podbilewicz, B. and Baumeister, R. ceh-16/engrailed patterns the embryonic epidermis of Caenorhabditis elegans. Development 132, 739-749. |
|  |  | Baugh, L.R., Hill, A.A., Claggett, J.M., Hill-Harfe, K., Wen, J.C., Slonim, D.K., Brown, E.L. and Hunter, C.P. The homeodomain protein PAL-1 specifies a lineage-specific regulatory network in the C. elegans embryo. Development 132, 1843-1854. |
|  |  | Lall, S., Grun, D., Krek, A., Chen, K., Wang, Y.L., Dewey, C.N., Sood, P., Colombo, T., Bray, N., Macmenamin, P. et al. A genome-wide map of conserved microRNA targets in C. elegans. Curr Biol 16, 460-471. |
|  |  | Podbilewicz, B. Cell fusion. WormBook, 1-32. |
|  |  | Okkema, P.G. and Krause, M. Transcriptional regulation. WormBook, 1-40. |
|  |  | Huang, X., Tian, E., Xu, Y. and Zhang, H. The C. elegans engrailed homolog ceh-16 regulates the self-renewal expansion division of stem cell-like seam cells. Dev Biol 333, 337-347. |
| ceh-17 | D1007.1 | Pujol, N., Torregrossa, P., Ewbank, J.J. and Brunet, J.F. The homeodomain protein CePHOX2/CEH-17 controls antero-posterior axonal growth in C. elegans. Development 127, 3361-3371. |
|  |  | Nelson, C.E., Hersh, B.M. and Carroll, S.B. The regulatory content of intergenic DNA shapes genome architecture. Genome Biol 5, R25. |
|  |  | Hobert, O. Specification of the nervous system. WormBook, 1-19. |
|  |  | Okkema, P.G. and Krause, M. Transcriptional regulation. WormBook, 1-40. |
|  |  | Van Buskirk, C. and Sternberg, P.W. Epidermal growth factor signaling induces behavioral quiescence in Caenorhabditis elegans. Nat Neurosci 10, 1300-1307. |
|  |  | Hallem, E.A. and Sternberg, P.W. Acute carbon dioxide avoidance in Caenorhabditis elegans. Proc Natl Acad Sci U S A 105, 8038-8043. |
|  |  | Jovelin, R. Rapid sequence evolution of transcription factors controlling neuron differentiation in Caenorhabditis. Mol Biol Evol 26, 2373-2386. |
|  |  | Van Buskirk, C. and Sternberg, P.W. Paired and LIM class homeodomain proteins coordinate differentiation of the C. elegans ALA neuron. Development 137, 2065-2074. |
| ceh-18 | ZC64.3 | Bird, D.M. Sequence comparison of the Caenorhabditis elegans dpy-13 and col-34 genes, and their deduced collagen products. Gene 120, 261-266. |
|  |  | Zwaal, R.R., Broeks, A., van Meurs, J., Groenen, J.T. and Plasterk, R.H. Target-selected gene inactivation in Caenorhabditis elegans by using a frozen transposon insertion mutant bank. Proc Natl Acad Sci U S A 90, 7431-7435. |
|  |  | Greenstein, D., Hird, S., Plasterk, R.H., Andachi, Y., Kohara, Y., Wang, B., Finney, M. and Ruvkun, G. Targeted mutations in the Caenorhabditis elegans POU homeo box gene ceh-18 cause defects in oocyte cell cycle arrest, gonad migration, and epidermal differentiation. Genes Dev 8, 1935-1948. |
|  |  | McCarter, J., Bartlett, B., Dang, T. and Schedl, T. Soma-germ cell interactions in Caenorhabditis elegans: multiple events of hermaphrodite germline development require the somatic sheath and spermathecal lineages. Dev Biol 181, 121-143. |
|  |  | Rose, K.L., Winfrey, V.P., Hoffman, L.H., Hall, D.H., Furuta, T. and Greenstein, D. The POU gene ceh-18 promotes gonadal sheath cell differentiation and function required for meiotic maturation and ovulation in Caenorhabditis elegans. Dev Biol 192, 59-77. |
|  |  | Hall, D.H., Winfrey, V.P., Blaeuer, G., Hoffman, L.H., Furuta, T., Rose, K.L., Hobert, O. and Greenstein, D. Ultrastructural features of the adult hermaphrodite gonad of Caenorhabditis elegans: relations between the germ line and soma. Dev Biol 212, 101-123. |
|  |  | Bürglin, T.R. and Ruvkun, G. Regulation of ectodermal and excretory function by the C. elegans POU homeobox gene ceh-6. Development 128, 779-790. |
|  |  | Kuwabara, P.E. The multifaceted C. elegans major sperm protein: an ephrin signaling antagonist in oocyte maturation. Genes Dev 17, 155-161. |
|  |  | Miller, M.A., Ruest, P.J., Kosinski, M., Hanks, S.K. and Greenstein, D. An Eph receptor sperm-sensing control mechanism for oocyte meiotic maturation in Caenorhabditis elegans. Genes Dev 17, 187-200. |
|  |  | Aono, S., Legouis, R., Hoose, W.A. and Kemphues, K.J. PAR-3 is required for epithelial cell polarity in the distal spermatheca of C. elegans. Development 131, 2865-2874. |
|  |  | Hamilton, B., Dong, Y., Shindo, M., Liu, W., Odell, I., Ruvkun, G. and Lee, S.S. A systematic RNAi screen for longevity genes in C. elegans. Genes Dev 19, 1544-1555. |
|  |  | Corrigan, C., Subramanian, R. and Miller, M.A. Eph and NMDA receptors control Ca2+/calmodulin-dependent protein kinase II activation during C. elegans oocyte meiotic maturation. Development 132, 5225-5237. |
|  |  | Suzuki, Y. and Han, M. Genetic redundancy masks diverse functions of the tumor suppressor gene PTEN during C. elegans development. Genes Dev 20, 423-428. |
|  |  | Greenstein, D. Control of oocyte meiotic maturation and fertilization. WormBook, 1-12. |
|  |  | Okkema, P.G. and Krause, M. Transcriptional regulation. WormBook, 1-40. |
|  |  | Labbe, J.C., Pacquelet, A., Marty, T. and Gotta, M. A genomewide screen for suppressors of par-2 uncovers potential regulators of PAR protein-dependent cell polarity in Caenorhabditis elegans. Genetics 174, 285-295. |
|  |  | Govindan, J.A., Cheng, H., Harris, J.E. and Greenstein, D. Galphao/i and Galphas signaling function in parallel with the MSP/Eph receptor to control meiotic diapause in C. elegans. Curr Biol 16, 1257-1268. |
|  |  | Harris, J.E., Govindan, J.A., Yamamoto, I., Schwartz, J., Kaverina, I. and Greenstein, D. Major sperm protein signaling promotes oocyte microtubule reorganization prior to fertilization in Caenorhabditis elegans. Dev Biol 299, 105-121. |
|  |  | Whitten, S.J. and Miller, M.A. The role of gap junctions in Caenorhabditis elegans oocyte maturation and fertilization. Dev Biol 301, 432-446. |
|  |  | Parry, D.H., Xu, J. and Ruvkun, G. A whole-genome RNAi Screen for C. elegans miRNA pathway genes. Curr Biol 17, 2013-2022. |
|  |  | Peden, E., Kimberly, E., Gengyo-Ando, K., Mitani, S. and Xue, D. Control of sex-specific apoptosis in C. elegans by the BarH homeodomain protein CEH-30 and the transcriptional repressor UNC-37/Groucho. Genes Dev 21, 3195-3207. |
|  |  | Jud, M.C., Czerwinski, M.J., Wood, M.P., Young, R.A., Gallo, C.M., Bickel, J.S., Petty, E.L., Mason, J.M., Little, B.A., Padilla, P.A. et al. Large P body-like RNPs form in C. elegans oocytes in response to arrested ovulation, heat shock, osmotic stress, and anoxia and are regulated by the major sperm protein pathway. Dev Biol 318, 38-51. |
|  |  | Voutev, R., Keating, R., Hubbard, E.J. and Vallier, L.G. Characterization of the Caenorhabditis elegans Islet LIM-homeodomain ortholog, lim-7. FEBS Lett 583, 456-464. |
| ceh-19 | F20D12.6 | Naito, M., Kohara, Y. and Kurosawa, Y. Identification of a homeobox-containing gene located between lin-45 and unc-24 on chromosome IV in the nematode Caenorhabditis elegans. Nucleic Acids Res 20, 2967-2969. |
|  |  | Stenico, M., Lloyd, A.T. and Sharp, P.M. Codon usage in Caenorhabditis elegans: delineation of translational selection and mutational biases. Nucleic Acids Res 22, 2437-2446. |
|  |  | Barnes, T.M., Jin, Y., Horvitz, H.R., Ruvkun, G. and Hekimi, S. The Caenorhabditis elegans behavioral gene unc-24 encodes a novel bipartite protein similar to both erythrocyte band 7.2 (stomatin) and nonspecific lipid transfer protein. J Neurochem 67, 46-57. |
|  |  | Okkema, P.G. and Krause, M. Transcriptional regulation. WormBook, 1-40. |
| ceh-20 | F31E3.1 | Bürglin, T.R. and Ruvkun, G. New motif in PBX genes. Nat Genet 1, 319-320. |
|  |  | Liu, J. and Fire, A. Overlapping roles of two Hox genes and the exd ortholog ceh-20 in diversification of the C. elegans postembryonic mesoderm. Development 127, 5179-5190. |
|  |  | Trachtulec, Z. and Forejt, J. Synteny of orthologous genes conserved in mammals, snake, fly, nematode, and fission yeast. Mamm Genome 12, 227-231. |
|  |  | Streit, A., Kohler, R., Marty, T., Belfiore, M., Takacs-Vellai, K., Vigano, M.A., Schnabel, R., Affolter, M. and Muller, F. Conserved regulation of the Caenorhabditis elegans labial/Hox1 gene ceh-13. Dev Biol 242, 96-108. |
|  |  | Van Auken, K., Weaver, D., Robertson, B., Sundaram, M., Saldi, T., Edgar, L., Elling, U., Lee, M., Boese, Q. and Wood, W.B. Roles of the Homothorax/Meis/Prep homolog UNC-62 and the Exd/Pbx homologs CEH-20 and CEH-40 in C. elegans embryogenesis. Development 129, 5255-5268. |
|  |  | Yang, L., Sym, M. and Kenyon, C. The roles of two C. elegans HOX co-factor orthologs in cell migration and vulva development. Development 132, 1413-1428. |
|  |  | Liu, H., Strauss, T.J., Potts, M.B. and Cameron, S. Direct regulation of egl-1 and of programmed cell death by the Hox protein MAB-5 and by CEH-20, a C. elegans homolog of Pbx1. Development 133, 641-650. |
|  |  | Podbilewicz, B. Cell fusion. WormBook, 1-32. |
|  |  | Okkema, P.G. and Krause, M. Transcriptional regulation. WormBook, 1-40. |
|  |  | Sternberg, P.W. Vulval development. WormBook, 1-28. |
|  |  | Wagmaister, J.A., Miley, G.R., Morris, C.A., Gleason, J.E., Miller, L.M., Kornfeld, K. and Eisenmann, D.M. Identification of cis-regulatory elements from the C. elegans Hox gene lin-39 required for embryonic expression and for regulation by the transcription factors LIN-1, LIN-31 and LIN-39. Dev Biol 297, 550-565. |
|  |  | Arata, Y., Kouike, H., Zhang, Y., Herman, M.A., Okano, H. and Sawa, H. Wnt signaling and a Hox protein cooperatively regulate psa-3/Meis to determine daughter cell fate after asymmetric cell division in C. elegans. Dev Cell 11, 105-115. |
|  |  | Takacs-Vellai, K., Vellai, T., Chen, E.B., Zhang, Y., Guerry, F., Stern, M.J. and Muller, F. Transcriptional control of Notch signaling by a HOX and a PBX/EXD protein during vulval development in C. elegans. Dev Biol 302, 661-669. |
|  |  | Amin, N.M., Hu, K., Pruyne, D., Terzic, D., Bretscher, A. and Liu, J. A Zn-finger/FH2-domain containing protein, FOZI-1, acts redundantly with CeMyoD to specify striated body wall muscle fates in the Caenorhabditis elegans postembryonic mesoderm. Development 134, 19-29. |
|  |  | Mukherjee, K. and Bürglin, T.R. Comprehensive analysis of animal TALE homeobox genes: new conserved motifs and cases of accelerated evolution. J Mol Evol 65, 137-153. |
|  |  | Jiang, Y., Shi, H., Amin, N.M., Sultan, I. and Liu, J. Mesodermal expression of the C. elegans HMX homolog mls-2 requires the PBC homolog CEH-20. Mech Dev 125, 451-461. |
|  |  | Potts, M.B., Wang, D.P. and Cameron, S. Trithorax, Hox, and TALE-class homeodomain proteins ensure cell survival through repression of the BH3-only gene egl-1. Dev Biol 329, 374-385. |
|  |  | Jiang, Y., Shi, H. and Liu, J. Two Hox cofactors, the Meis/Hth homolog UNC-62 and the Pbx/Exd homolog CEH-20, function together during C. elegans postembryonic mesodermal development. Dev Biol 334, 535-546. |
| ceh-21 | T26C11.6 | Lannoy, V.J., Bürglin, T.R., Rousseau, G.G. and Lemaigre, F.P. Isoforms of hepatocyte nuclear factor-6 differ in DNA-binding properties, contain a bifunctional homeodomain, and define the new ONECUT class of homeodomain proteins. J Biol Chem 273, 13552-13562. |
|  |  | Bürglin, T.R. and Cassata, G. Loss and gain of domains during evolution of cut superclass homeobox genes. Int J Dev Biol 46, 115-123. |
|  |  | Okkema, P.G. and Krause, M. Transcriptional regulation. WormBook, 1-40. |
|  |  | Gladden, J.M. and Meyer, B.J. A ONECUT homeodomain protein communicates X chromosome dose to specify Caenorhabditis elegans sexual fate by repressing a sex switch gene. Genetics 177, 1621-1637. |
|  |  | Updike, D.L. and Strome, S. A genomewide RNAi screen for genes that affect the stability, distribution and function of P granules in Caenorhabditis elegans. Genetics 183, 1397-1419. |
| ceh-22 | F29F11.5 | Okkema, P.G. and Fire, A. The Caenorhabditis elegans NK-2 class homeoprotein CEH-22 is involved in combinatorial activation of gene expression in pharyngeal muscle. Development 120, 2175-2186. |
|  |  | Krause, M. MyoD and myogenesis in C. elegans. Bioessays 17, 219-228. |
|  |  | Egan, C.R., Chung, M.A., Allen, F.L., Heschl, M.F., Van Buskirk, C.L. and McGhee, J.D. A gut-to-pharynx/tail switch in embryonic expression of the Caenorhabditis elegans ges-1 gene centers on two GATA sequences. Dev Biol 170, 397-419. |
|  |  | Okkema, P.G., Ha, E., Haun, C., Chen, W. and Fire, A. The Caenorhabditis elegans NK-2 homeobox gene ceh-22 activates pharyngeal muscle gene expression in combination with pha-1 and is required for normal pharyngeal development. Development 124, 3965-3973. |
|  |  | Harfe, B.D. and Fire, A. Muscle and nerve-specific regulation of a novel NK-2 class homeodomain factor in Caenorhabditis elegans. Development 125, 421-429. |
|  |  | Haun, C., Alexander, J., Stainier, D.Y. and Okkema, P.G. Rescue of Caenorhabditis elegans pharyngeal development by a vertebrate heart specification gene. Proc Natl Acad Sci U S A 95, 5072-5075. |
|  |  | Kalb, J.M., Lau, K.K., Goszczynski, B., Fukushige, T., Moons, D., Okkema, P.G. and McGhee, J.D. pha-4 is Ce-fkh-1, a fork head/HNF-3alpha,beta,gamma homolog that functions in organogenesis of the C. elegans pharynx. Development 125, 2171-2180. |
|  |  | Newman-Smith, E.D. and Rothman, J.H. The maternal-to-zygotic transition in embryonic patterning of Caenorhabditis elegans. Curr Opin Genet Dev 8, 472-480. |
|  |  | Culetto, E., Combes, D., Fedon, Y., Roig, A., Toutant, J.P. and Arpagaus, M. Structure and promoter activity of the 5' flanking region of ace-1, the gene encoding acetylcholinesterase of class A in Caenorhabditis elegans. J Mol Biol 290, 951-966. |
|  |  | Gower, N.J., Temple, G.R., Schein, J.E., Marra, M., Walker, D.S. and Baylis, H.A. Dissection of the promoter region of the inositol 1,4,5-trisphosphate receptor gene, itr-1, in C. elegans: a molecular basis for cell-specific expression of IP3R isoforms. J Mol Biol 306, 145-157. |
|  |  | Kuchenthal, C.A., Chen, W. and Okkema, P.G. Multiple enhancers contribute to expression of the NK-2 homeobox gene ceh-22 in C. elegans pharyngeal muscle. Genesis 31, 156-166. |
|  |  | Kalb, J.M., Beaster-Jones, L., Fernandez, A.P., Okkema, P.G., Goszczynski, B. and McGhee, J.D. Interference between the PHA-4 and PEB-1 transcription factors in formation of the Caenorhabditis elegans pharynx. J Mol Biol 320, 697-704. |
|  |  | Vilimas, T., Abraham, A. and Okkema, P.G. An early pharyngeal muscle enhancer from the Caenorhabditis elegans ceh-22 gene is targeted by the Forkhead factor PHA-4. Dev Biol 266, 388-398. |
|  |  | Siegfried, K.R., Kidd, A.R., 3rd, Chesney, M.A. and Kimble, J. The sys-1 and sys-3 genes cooperate with Wnt signaling to establish the proximal-distal axis of the Caenorhabditis elegans gonad. Genetics 166, 171-186. |
|  |  | Wenick, A.S. and Hobert, O. Genomic cis-regulatory architecture and trans-acting regulators of a single interneuron-specific gene battery in C. elegans. Dev Cell 6, 757-770. |
|  |  | Morck, C., Rauthan, M., Wagberg, F. and Pilon, M. pha-2 encodes the C. elegans ortholog of the homeodomain protein HEX and is required for the formation of the pharyngeal isthmus. Dev Biol 272, 403-418. |
|  |  | Ao, W., Gaudet, J., Kent, W.J., Muttumu, S. and Mango, S.E. Environmentally induced foregut remodeling by PHA-4/FoxA and DAF-12/NHR. Science 305, 1743-1746. |
|  |  | Pilon, M. and Morck, C. Development of Caenorhabditis elegans pharynx, with emphasis on its nervous system. Acta Pharmacol Sin 26, 396-404. |
|  |  | Lam, N., Chesney, M.A. and Kimble, J. Wnt signaling and CEH-22/tinman/Nkx2.5 specify a stem cell niche in C. elegans. Curr Biol 16, 287-295. |
|  |  | Herman, M.A. Hermaphrodite cell-fate specification. WormBook, 1-16. |
|  |  | Okkema, P.G. and Krause, M. Transcriptional regulation. WormBook, 1-40. |
|  |  | Eisenmann, D.M. Wnt signaling. WormBook, 1-17. |
|  |  | Boulin, T., Etchberger, J.F. and Hobert, O. Reporter gene fusions. WormBook, 1-23. |
|  |  | Roy Chowdhuri, S., Crum, T., Woollard, A., Aslam, S. and Okkema, P.G. The T-box factor TBX-2 and the SUMO conjugating enzyme UBC-9 are required for ABa-derived pharyngeal muscle in C. elegans. Dev Biol 295, 664-677. |
|  |  | Higashibata, A., Szewczyk, N.J., Conley, C.A., Imamizo-Sato, M., Higashitani, A. and Ishioka, N. Decreased expression of myogenic transcription factors and myosin heavy chains in Caenorhabditis elegans muscles developed during spaceflight. J Exp Biol 209, 3209-3218. |
|  |  | Smith, P.A. and Mango, S.E. Role of T-box gene tbx-2 for anterior foregut muscle development in C. elegans. Dev Biol 302, 25-39. |
|  |  | Mango, S.E. The C. elegans pharynx: a model for organogenesis. WormBook, 1-26. |
|  |  | Phillips, B.T., Kidd, A.R., 3rd, King, R., Hardin, J. and Kimble, J. Reciprocal asymmetry of SYS-1/beta-catenin and POP-1/TCF controls asymmetric divisions in Caenorhabditis elegans. Proc Natl Acad Sci U S A 104, 3231-3236. |
|  |  | Berger, M.F., Badis, G., Gehrke, A.R., Talukder, S., Philippakis, A.A., Pena-Castillo, L., Alleyne, T.M., Mnaimneh, S., Botvinnik, O.B., Chan, E.T. et al. Variation in homeodomain DNA binding revealed by high-resolution analysis of sequence preferences. Cell 133, 1266-1276. |
|  |  | Van Hoffelen, S. and Herman, M.A. Analysis of Wnt signaling during Caenorhabditis elegans postembryonic development. Methods Mol Biol 469, 87-102. |
|  |  | Chesney, M.A., Lam, N., Morgan, D.E., Phillips, B.T. and Kimble, J. C. elegans HLH-2/E/Daughterless controls key regulatory cells during gonadogenesis. Dev Biol 331, 14-25. |
|  |  | Vavouri, T., Semple, J.I., Garcia-Verdugo, R. and Lehner, B. Intrinsic protein disorder and interaction promiscuity are widely associated with dosage sensitivity. Cell 138, 198-208. |
|  |  | Raharjo, W.H., Logan, B.C., Wen, S., Kalb, J.M. and Gaudet, J. In vitro and in vivo characterization of Caenorhabditis elegans PHA-4/FoxA response elements. Dev Dyn 239, 2219-2232. |
| ceh-23 | ZK652.5 | Wang, B.B., Muller-Immergluck, M.M., Austin, J., Robinson, N.T., Chisholm, A. and Kenyon, C. A homeotic gene cluster patterns the anteroposterior body axis of C. elegans. Cell 74, 29-42. |
|  |  | Bürglin, T.R. and Ruvkun, G. The Caenorhabditis elegans homeobox gene cluster. Curr Opin Genet Dev 3, 615-620. |
|  |  | Wilson, R., Ainscough, R., Anderson, K., Baynes, C., Berks, M., Bonfield, J., Burton, J., Connell, M., Copsey, T., Cooper, J. et al. 2.2 Mb of contiguous nucleotide sequence from chromosome III of C. elegans. Nature 368, 32-38. |
|  |  | Wightman, B., Clark, S.G., Taskar, A.M., Forrester, W.C., Maricq, A.V., Bargmann, C.I. and Garriga, G. The C. elegans gene vab-8 guides posteriorly directed axon outgrowth and cell migration. Development 122, 671-682. |
|  |  | Zallen, J.A., Kirch, S.A. and Bargmann, C.I. Genes required for axon pathfinding and extension in the C. elegans nerve ring. Development 126, 3679-3692. |
|  |  | Knobel, K.M., Jorgensen, E.M. and Bastiani, M.J. Growth cones stall and collapse during axon outgrowth in Caenorhabditis elegans. Development 126, 4489-4498. |
|  |  | Swoboda, P., Adler, H.T. and Thomas, J.H. The RFX-type transcription factor DAF-19 regulates sensory neuron cilium formation in C. elegans. Mol Cell 5, 411-421. |
|  |  | Altun-Gultekin, Z., Andachi, Y., Tsalik, E.L., Pilgrim, D., Kohara, Y. and Hobert, O. A regulatory cascade of three homeobox genes, ceh-10, ttx-3 and ceh-23, controls cell fate specification of a defined interneuron class in C. elegans. Development 128, 1951-1969. |
|  |  | Uchida, O., Nakano, H., Koga, M. and Ohshima, Y. The C. elegans che-1 gene encodes a zinc finger transcription factor required for specification of the ASE chemosensory neurons. Development 130, 1215-1224. |
|  |  | Tsalik, E.L., Niacaris, T., Wenick, A.S., Pau, K., Avery, L. and Hobert, O. LIM homeobox gene-dependent expression of biogenic amine receptors in restricted regions of the C. elegans nervous system. Dev Biol 263, 81-102. |
|  |  | Wenick, A.S. and Hobert, O. Genomic cis-regulatory architecture and trans-acting regulators of a single interneuron-specific gene battery in C. elegans. Dev Cell 6, 757-770. |
|  |  | Sha, K. and Fire, A. Imprinting capacity of gamete lineages in Caenorhabditis elegans. Genetics 170, 1633-1652. |
|  |  | Hobert, O. Specification of the nervous system. WormBook, 1-19. |
|  |  | Okkema, P.G. and Krause, M. Transcriptional regulation. WormBook, 1-40. |
|  |  | Etchberger, J.F., Lorch, A., Sleumer, M.C., Zapf, R., Jones, S.J., Marra, M.A., Holt, R.A., Moerman, D.G. and Hobert, O. The molecular signature and cis-regulatory architecture of a C. elegans gustatory neuron. Genes Dev 21, 1653-1674. |
|  |  | Hallem, E.A. and Sternberg, P.W. Acute carbon dioxide avoidance in Caenorhabditis elegans. Proc Natl Acad Sci U S A 105, 8038-8043. |
|  |  | Jovelin, R. Rapid sequence evolution of transcription factors controlling neuron differentiation in Caenorhabditis. Mol Biol Evol 26, 2373-2386. |
| ceh-24 | F55B12.1 | Harfe, B.D. and Fire, A. Muscle and nerve-specific regulation of a novel NK-2 class homeodomain factor in Caenorhabditis elegans. Development 125, 421-429. |
|  |  | Harfe, B.D., Branda, C.S., Krause, M., Stern, M.J. and Fire, A. MyoD and the specification of muscle and non-muscle fates during postembryonic development of the C. elegans mesoderm. Development 125, 2479-2488. |
|  |  | Harfe, B.D., Vaz Gomes, A., Kenyon, C., Liu, J., Krause, M. and Fire, A. Analysis of a Caenorhabditis elegans Twist homolog identifies conserved and divergent aspects of mesodermal patterning. Genes Dev 12, 2623-2635. |
|  |  | Corsi, A.K., Kostas, S.A., Fire, A. and Krause, M. Caenorhabditis elegans twist plays an essential role in non-striated muscle development. Development 127, 2041-2051. |
|  |  | Gower, N.J., Temple, G.R., Schein, J.E., Marra, M., Walker, D.S. and Baylis, H.A. Dissection of the promoter region of the inositol 1,4,5-trisphosphate receptor gene, itr-1, in C. elegans: a molecular basis for cell-specific expression of IP3R isoforms. J Mol Biol 306, 145-157. |
|  |  | Kostas, S.A. and Fire, A. The T-box factor MLS-1 acts as a molecular switch during specification of nonstriated muscle in C. elegans. Genes Dev 16, 257-269. |
|  |  | Corsi, A.K., Brodigan, T.M., Jorgensen, E.M. and Krause, M. Characterization of a dominant negative C. elegans Twist mutant protein with implications for human Saethre-Chotzen syndrome. Development 129, 2761-2772. |
|  |  | Wang, P., Zhao, J. and Corsi, A.K. Identification of novel target genes of CeTwist and CeE/DA. Dev Biol 293, 486-498. |
|  |  | Okkema, P.G. and Krause, M. Transcriptional regulation. WormBook, 1-40. |
|  |  | Mango, S.E. The C. elegans pharynx: a model for organogenesis. WormBook, 1-26. |
|  |  | Neves, A., English, K. and Priess, J.R. Notch-GATA synergy promotes endoderm-specific expression of ref-1 in C. elegans. Development 134, 4459-4468. |
|  |  | Rasmussen, J.P., English, K., Tenlen, J.R. and Priess, J.R. Notch signaling and morphogenesis of single-cell tubes in the C. elegans digestive tract. Dev Cell 14, 559-569. |
|  |  | Broitman-Maduro, G., Owraghi, M., Hung, W.W., Kuntz, S., Sternberg, P.W. and Maduro, M.F. The NK-2 class homeodomain factor CEH-51 and the T-box factor TBX-35 have overlapping function in C. elegans mesoderm development. Development 136, 2735-2746. |
| ceh-26 | K12H4.1 | Hofmann, E.R., Milstein, S., Boulton, S.J., Ye, M., Hofmann, J.J., Stergiou, L., Gartner, A., Vidal, M. and Hengartner, M.O. Caenorhabditis elegans HUS-1 is a DNA damage checkpoint protein required for genome stability and EGL-1-mediated apoptosis. Curr Biol 12, 1908-1918. |
|  |  | Van Auken, K., Weaver, D., Robertson, B., Sundaram, M., Saldi, T., Edgar, L., Elling, U., Lee, M., Boese, Q. and Wood, W.B. Roles of the Homothorax/Meis/Prep homolog UNC-62 and the Exd/Pbx homologs CEH-20 and CEH-40 in C. elegans embryogenesis. Development 129, 5255-5268. |
|  |  | Yu, H., Prétôt, R.F., Bürglin, T.R. and Sternberg, P.W. Distinct roles of transcription factors EGL-46 and DAF-19 in specifying the functionality of a polycystin-expressing sensory neuron necessary for C. elegans male vulva location behavior. Development 130, 5217-5227. |
|  |  | Nelson, C.E., Hersh, B.M. and Carroll, S.B. The regulatory content of intergenic DNA shapes genome architecture. Genome Biol 5, R25. |
|  |  | Okkema, P.G. and Krause, M. Transcriptional regulation. WormBook, 1-40. |
| ceh-27 | F46F3.1 | Okkema, P.G. and Krause, M. Transcriptional regulation. WormBook, 1-40. |
| ceh-28 | K03A11.3 | Okkema, P.G. and Krause, M. Transcriptional regulation. WormBook, 1-40. |
|  |  | Ray, P., Schnabel, R. and Okkema, P.G. Behavioral and synaptic defects in C. elegans lacking the NK-2 homeobox gene ceh-28. Dev Neurobiol 68, 421-433. |
| ceh-30 | C33D12.7 | Zarkower, D. Somatic sex determination. WormBook, 1-12. |
|  |  | Okkema, P.G. and Krause, M. Transcriptional regulation. WormBook, 1-40. |
|  |  | Chasnov, J.R., So, W.K., Chan, C.M. and Chow, K.L. The species, sex, and stage specificity of a Caenorhabditis sex pheromone. Proc Natl Acad Sci U S A 104, 6730-6735. |
|  |  | Lee, K. and Portman, D.S. Neural sex modifies the function of a C. elegans sensory circuit. Curr Biol 17, 1858-1863. |
|  |  | White, J.Q., Nicholas, T.J., Gritton, J., Truong, L., Davidson, E.R. and Jorgensen, E.M. The sensory circuitry for sexual attraction in C. elegans males. Curr Biol 17, 1847-1857. |
|  |  | Schwartz, H.T. and Horvitz, H.R. The C. elegans protein CEH-30 protects male-specific neurons from apoptosis independently of the Bcl-2 homolog CED-9. Genes Dev 21, 3181-3194. |
|  |  | Peden, E., Kimberly, E., Gengyo-Ando, K., Mitani, S. and Xue, D. Control of sex-specific apoptosis in C. elegans by the BarH homeodomain protein CEH-30 and the transcriptional repressor UNC-37/Groucho. Genes Dev 21, 3195-3207. |
|  |  | Nehme, R., Grote, P., Tomasi, T., Loser, S., Holzkamp, H., Schnabel, R. and Conradt, B. Transcriptional upregulation of both egl-1 BH3-only and ced-3 caspase is required for the death of the male-specific CEM neurons. Cell Death Differ 17, 1266-1276. |
| ceh-31 | C33D12.1 | Okkema, P.G. and Krause, M. Transcriptional regulation. WormBook, 1-40. |
| ceh-32 | W05E10.3 | Dozier, C., Kagoshima, H., Niklaus, G., Cassata, G. and Bürglin, T.R. The Caenorhabditis elegans Six/sine oculis class homeobox gene ceh-32 is required for head morphogenesis. Dev Biol 236, 289-303. |
|  |  | Kamath, R.S., Fraser, A.G., Dong, Y., Poulin, G., Durbin, R., Gotta, M., Kanapin, A., Le Bot, N., Moreno, S., Sohrmann, M. et al. Systematic functional analysis of the Caenorhabditis elegans genome using RNAi. Nature 421, 231-237. |
|  |  | Yanagi, K., Mizuno, T., Tsuyama, T., Tada, S., Iida, Y., Sugimoto, A., Eki, T., Enomoto, T. and Hanaoka, F. Caenorhabditis elegans geminin homologue participates in cell cycle regulation and germ line development. J Biol Chem 280, 19689-19694. |
|  |  | Okkema, P.G. and Krause, M. Transcriptional regulation. WormBook, 1-40. |
|  |  | Reece-Hoyes, J.S., Shingles, J., Dupuy, D., Grove, C.A., Walhout, A.J., Vidal, M. and Hope, I.A. Insight into transcription factor gene duplication from Caenorhabditis elegans Promoterome-driven expression patterns. BMC Genomics 8, 27. |
|  |  | Amin, N.M., Lim, S.E., Shi, H., Chan, T.L. and Liu, J. A conserved Six-Eya cassette acts downstream of Wnt signaling to direct non-myogenic versus myogenic fates in the C. elegans postembryonic mesoderm. Dev Biol 331, 350-360. |
| ceh-33 | C10G8.7 | Dozier, C., Kagoshima, H., Niklaus, G., Cassata, G. and Bürglin, T.R. The Caenorhabditis elegans Six/sine oculis class homeobox gene ceh-32 is required for head morphogenesis. Dev Biol 236, 289-303. |
|  |  | Okkema, P.G. and Krause, M. Transcriptional regulation. WormBook, 1-40. |
|  |  | Reece-Hoyes, J.S., Shingles, J., Dupuy, D., Grove, C.A., Walhout, A.J., Vidal, M. and Hope, I.A. Insight into transcription factor gene duplication from Caenorhabditis elegans Promoterome-driven expression patterns. BMC Genomics 8, 27. |
|  |  | Amin, N.M., Lim, S.E., Shi, H., Chan, T.L. and Liu, J. A conserved Six-Eya cassette acts downstream of Wnt signaling to direct non-myogenic versus myogenic fates in the C. elegans postembryonic mesoderm. Dev Biol 331, 350-360. |
| ceh-34 | C10G8.6 | Dozier, C., Kagoshima, H., Niklaus, G., Cassata, G. and Bürglin, T.R. The Caenorhabditis elegans Six/sine oculis class homeobox gene ceh-32 is required for head morphogenesis. Dev Biol 236, 289-303. |
|  |  | Okkema, P.G. and Krause, M. Transcriptional regulation. WormBook, 1-40. |
|  |  | Reece-Hoyes, J.S., Shingles, J., Dupuy, D., Grove, C.A., Walhout, A.J., Vidal, M. and Hope, I.A. Insight into transcription factor gene duplication from Caenorhabditis elegans Promoterome-driven expression patterns. BMC Genomics 8, 27. |
|  |  | Amin, N.M., Lim, S.E., Shi, H., Chan, T.L. and Liu, J. A conserved Six-Eya cassette acts downstream of Wnt signaling to direct non-myogenic versus myogenic fates in the C. elegans postembryonic mesoderm. Dev Biol 331, 350-360. |
|  |  | Amin, N.M., Shi, H. and Liu, J. The FoxF/FoxC factor LET-381 directly regulates both cell fate specification and cell differentiation in C. elegans mesoderm development. Development 137, 1451-1460. |
|  |  | Hirose, T., Galvin, B.D. and Horvitz, H.R. Six and Eya promote apoptosis through direct transcriptional activation of the proapoptotic BH3-only gene egl-1 in Caenorhabditis elegans. Proc Natl Acad Sci U S A 107, 15479-15484. |
| ceh-36 | C37E2.4 | Chang, S., Johnston, R.J., Jr. and Hobert, O. A transcriptional regulatory cascade that controls left/right asymmetry in chemosensory neurons of C. elegans. Genes Dev 17, 2123-2137. |
|  |  | Lanjuin, A., VanHoven, M.K., Bargmann, C.I., Thompson, J.K. and Sengupta, P. Otx/otd homeobox genes specify distinct sensory neuron identities in C. elegans. Dev Cell 5, 621-633. |
|  |  | Johnston, R.J. and Hobert, O. A microRNA controlling left/right neuronal asymmetry in Caenorhabditis elegans. Nature 426, 845-849. |
|  |  | Koga, M. and Ohshima, Y. The C. elegans ceh-36 gene encodes a putative homemodomain transcription factor involved in chemosensory functions of ASE and AWC neurons. J Mol Biol 336, 579-587. |
|  |  | Melkman, T. and Sengupta, P. The worm's sense of smell. Development of functional diversity in the chemosensory system of Caenorhabditis elegans. Dev Biol 265, 302-319. |
|  |  | Lanjuin, A. and Sengupta, P. Specification of chemosensory neuron subtype identities in Caenorhabditis elegans. Curr Opin Neurobiol 14, 22-30. |
|  |  | Chang, S., Johnston, R.J., Jr., Frokjaer-Jensen, C., Lockery, S. and Hobert, O. MicroRNAs act sequentially and asymmetrically to control chemosensory laterality in the nematode. Nature 430, 785-789. |
|  |  | Johnston, R.J., Jr., Chang, S., Etchberger, J.F., Ortiz, C.O. and Hobert, O. MicroRNAs acting in a double-negative feedback loop to control a neuronal cell fate decision. Proc Natl Acad Sci U S A 102, 12449-12454. |
|  |  | Kim, K., Colosimo, M.E., Yeung, H. and Sengupta, P. The UNC-3 Olf/EBF protein represses alternate neuronal programs to specify chemosensory neuron identity. Dev Biol 286, 136-148. |
|  |  | Hukema, R.K., Rademakers, S., Dekkers, M.P., Burghoorn, J. and Jansen, G. Antagonistic sensory cues generate gustatory plasticity in Caenorhabditis elegans. Embo J 25, 312-322. |
|  |  | Johnston, R.J., Jr. and Hobert, O. A novel C. elegans zinc finger transcription factor, lsy-2, required for the cell type-specific expression of the lsy-6 microRNA. Development 132, 5451-5460. |
|  |  | Hobert, O. Specification of the nervous system. WormBook, 1-19. |
|  |  | Okkema, P.G. and Krause, M. Transcriptional regulation. WormBook, 1-40. |
|  |  | Bargmann, C.I. Chemosensation in C. elegans. WormBook, 1-29. |
|  |  | Pradel, E., Zhang, Y., Pujol, N., Matsuyama, T., Bargmann, C.I. and Ewbank, J.J. Detection and avoidance of a natural product from the pathogenic bacterium Serratia marcescens by Caenorhabditis elegans. Proc Natl Acad Sci U S A 104, 2295-2300. |
|  |  | Etchberger, J.F., Lorch, A., Sleumer, M.C., Zapf, R., Jones, S.J., Marra, M.A., Holt, R.A., Moerman, D.G. and Hobert, O. The molecular signature and cis-regulatory architecture of a C. elegans gustatory neuron. Genes Dev 21, 1653-1674. |
|  |  | Sarin, S., O'Meara, M.M., Flowers, E.B., Antonio, C., Poole, R.J., Didiano, D., Johnston, R.J., Jr., Chang, S., Narula, S. and Hobert, O. Genetic screens for Caenorhabditis elegans mutants defective in left/right asymmetric neuronal fate specification. Genetics 176, 2109-2130. |
|  |  | White, J.Q., Nicholas, T.J., Gritton, J., Truong, L., Davidson, E.R. and Jorgensen, E.M. The sensory circuitry for sexual attraction in C. elegans males. Curr Biol 17, 1847-1857. |
|  |  | Luo, L., Gabel, C.V., Ha, H.I., Zhang, Y. and Samuel, A.D. Olfactory behavior of swimming C. elegans analyzed by measuring motile responses to temporal variations of odorants. J Neurophysiol 99, 2617-2625. |
|  |  | Hallem, E.A. and Sternberg, P.W. Acute carbon dioxide avoidance in Caenorhabditis elegans. Proc Natl Acad Sci U S A 105, 8038-8043. |
|  |  | Frokjaer-Jensen, C., Ailion, M. and Lockery, S.R. Ammonium-acetate is sensed by gustatory and olfactory neurons in Caenorhabditis elegans. PLoS One 3, e2467. |
|  |  | Tsunozaki, M., Chalasani, S.H. and Bargmann, C.I. A behavioral switch: cGMP and PKC signaling in olfactory neurons reverses odor preference in C. elegans. Neuron 59, 959-971. |
|  |  | Jovelin, R., Dunham, J.P., Sung, F.S. and Phillips, P.C. High nucleotide divergence in developmental regulatory genes contrasts with the structural elements of olfactory pathways in caenorhabditis. Genetics 181, 1387-1397. |
|  |  | Etchberger, J.F., Flowers, E.B., Poole, R.J., Bashllari, E. and Hobert, O. Cis-regulatory mechanisms of left/right asymmetric neuron-subtype specification in C. elegans. Development 136, 147-160. |
|  |  | Jovelin, R. Rapid sequence evolution of transcription factors controlling neuron differentiation in Caenorhabditis. Mol Biol Evol 26, 2373-2386. |
|  |  | Ben Arous, J., Laffont, S. and Chatenay, D. Molecular and sensory basis of a food related two-state behavior in C. elegans. PLoS One 4, e7584. |
|  |  | Kim, K., Kim, R. and Sengupta, P. The HMX/NKX homeodomain protein MLS-2 specifies the identity of the AWC sensory neuron type via regulation of the ceh-36 Otx gene in C. elegans. Development 137, 963-974. |
| ceh-37 | C37E2.5 | Kim, S.H., Hwang, S.B., Chung, I.K. and Lee, J. Sequence-specific binding to telomeric DNA by CEH-37, a homeodomain protein in the nematode Caenorhabditis elegans. J Biol Chem 278, 28038-28044. |
|  |  | Lanjuin, A., VanHoven, M.K., Bargmann, C.I., Thompson, J.K. and Sengupta, P. Otx/otd homeobox genes specify distinct sensory neuron identities in C. elegans. Dev Cell 5, 621-633. |
|  |  | Im, S.H. and Lee, J. Identification of HMG-5 as a double-stranded telomeric DNA-binding protein in the nematode Caenorhabditis elegans. FEBS Lett 554, 455-461. |
|  |  | Melkman, T. and Sengupta, P. The worm's sense of smell. Development of functional diversity in the chemosensory system of Caenorhabditis elegans. Dev Biol 265, 302-319. |
|  |  | Lanjuin, A. and Sengupta, P. Specification of chemosensory neuron subtype identities in Caenorhabditis elegans. Curr Opin Neurobiol 14, 22-30. |
|  |  | Zheng, X., Chung, S., Tanabe, T. and Sze, J.Y. Cell-type specific regulation of serotonergic identity by the C. elegans LIM-homeodomain factor LIM-4. Dev Biol 286, 618-628. |
|  |  | Hobert, O. Specification of the nervous system. WormBook, 1-19. |
|  |  | Okkema, P.G. and Krause, M. Transcriptional regulation. WormBook, 1-40. |
|  |  | Bargmann, C.I. Chemosensation in C. elegans. WormBook, 1-29. |
|  |  | Mukhopadhyay, S., Lu, Y., Qin, H., Lanjuin, A., Shaham, S. and Sengupta, P. Distinct IFT mechanisms contribute to the generation of ciliary structural diversity in C. elegans. Embo J 26, 2966-2980. |
|  |  | Hallem, E.A. and Sternberg, P.W. Acute carbon dioxide avoidance in Caenorhabditis elegans. Proc Natl Acad Sci U S A 105, 8038-8043. |
|  |  | Jovelin, R. Rapid sequence evolution of transcription factors controlling neuron differentiation in Caenorhabditis. Mol Biol Evol 26, 2373-2386. |
| ceh-38 | F22D3.1 | Cassata, G., Kagoshima, H., Prétôt, R.F., Aspöck, G., Niklaus, G. and Bürglin, T.R. Rapid expression screening of Caenorhabditis elegans homeobox open reading frames using a two-step polymerase chain reaction promoter-gfp reporter construction technique. Gene 212, 127-135. |
|  |  | Lannoy, V.J., Bürglin, T.R., Rousseau, G.G. and Lemaigre, F.P. Isoforms of hepatocyte nuclear factor-6 differ in DNA-binding properties, contain a bifunctional homeodomain, and define the new ONECUT class of homeodomain proteins. J Biol Chem 273, 13552-13562. |
|  |  | Bürglin, T.R. and Cassata, G. Loss and gain of domains during evolution of cut superclass homeobox genes. Int J Dev Biol 46, 115-123. |
|  |  | Couillault, C., Pujol, N., Reboul, J., Sabatier, L., Guichou, J.F., Kohara, Y. and Ewbank, J.J. TLR-independent control of innate immunity in Caenorhabditis elegans by the TIR domain adaptor protein TIR-1, an ortholog of human SARM. Nat Immunol 5, 488-494. |
|  |  | Okkema, P.G. and Krause, M. Transcriptional regulation. WormBook, 1-40. |
|  |  | Etchberger, J.F., Lorch, A., Sleumer, M.C., Zapf, R., Jones, S.J., Marra, M.A., Holt, R.A., Moerman, D.G. and Hobert, O. The molecular signature and cis-regulatory architecture of a C. elegans gustatory neuron. Genes Dev 21, 1653-1674. |
|  |  | Lin, B. and Reinke, V. The candidate MAP kinase phosphorylation substrate DPL-1 (DP) promotes expression of the MAP kinase phosphatase LIP-1 in C. elegans germ cells. Dev Biol 316, 50-61. |
| ceh-39 | T26C11.7 | Lannoy, V.J., Bürglin, T.R., Rousseau, G.G. and Lemaigre, F.P. Isoforms of hepatocyte nuclear factor-6 differ in DNA-binding properties, contain a bifunctional homeodomain, and define the new ONECUT class of homeodomain proteins. J Biol Chem 273, 13552-13562. |
|  |  | Bürglin, T.R. and Cassata, G. Loss and gain of domains during evolution of cut superclass homeobox genes. Int J Dev Biol 46, 115-123. |
|  |  | Powell, J.R., Jow, M.M. and Meyer, B.J. The T-box transcription factor SEA-1 is an autosomal element of the X:A signal that determines C. elegans sex. Dev Cell 9, 339-349. |
|  |  | Okkema, P.G. and Krause, M. Transcriptional regulation. WormBook, 1-40. |
|  |  | Maydan, J.S., Flibotte, S., Edgley, M.L., Lau, J., Selzer, R.R., Richmond, T.A., Pofahl, N.J., Thomas, J.H. and Moerman, D.G. Efficient high-resolution deletion discovery in Caenorhabditis elegans by array comparative genomic hybridization. Genome Res 17, 337-347. |
|  |  | Gladden, J.M. and Meyer, B.J. A ONECUT homeodomain protein communicates X chromosome dose to specify Caenorhabditis elegans sexual fate by repressing a sex switch gene. Genetics 177, 1621-1637. |
|  |  | Gladden, J.M., Farboud, B. and Meyer, B.J. Revisiting the X:A signal that specifies Caenorhabditis elegans sexual fate. Genetics 177, 1639-1654. |
| ceh-40 | F17A2.5 | Van Auken, K., Weaver, D., Robertson, B., Sundaram, M., Saldi, T., Edgar, L., Elling, U., Lee, M., Boese, Q. and Wood, W.B. Roles of the Homothorax/Meis/Prep homolog UNC-62 and the Exd/Pbx homologs CEH-20 and CEH-40 in C. elegans embryogenesis. Development 129, 5255-5268. |
|  |  | Aspöck, G., Ruvkun, G. and Bürglin, T.R. The Caenorhabditis elegans ems class homeobox gene ceh-2 is required for M3 pharynx motoneuron function. Development 130, 3369-3378. |
|  |  | Baugh, L.R., Wen, J.C., Hill, A.A., Slonim, D.K., Brown, E.L. and Hunter, C.P. Synthetic lethal analysis of Caenorhabditis elegans posterior embryonic patterning genes identifies conserved genetic interactions. Genome Biol 6, R45. |
|  |  | Liu, H., Strauss, T.J., Potts, M.B. and Cameron, S. Direct regulation of egl-1 and of programmed cell death by the Hox protein MAB-5 and by CEH-20, a C. elegans homolog of Pbx1. Development 133, 641-650. |
|  |  | Okkema, P.G. and Krause, M. Transcriptional regulation. WormBook, 1-40. |
|  |  | Mukherjee, K. and Bürglin, T.R. Comprehensive analysis of animal TALE homeobox genes: new conserved motifs and cases of accelerated evolution. J Mol Evol 65, 137-153. |
|  |  | Jiang, Y., Shi, H., Amin, N.M., Sultan, I. and Liu, J. Mesodermal expression of the C. elegans HMX homolog mls-2 requires the PBC homolog CEH-20. Mech Dev 125, 451-461. |
| ceh-41 | T26C11.5 | Bürglin, T.R. and Cassata, G. Loss and gain of domains during evolution of cut superclass homeobox genes. Int J Dev Biol 46, 115-123. |
|  |  | Okkema, P.G. and Krause, M. Transcriptional regulation. WormBook, 1-40. |
|  |  | Gladden, J.M. and Meyer, B.J. A ONECUT homeodomain protein communicates X chromosome dose to specify Caenorhabditis elegans sexual fate by repressing a sex switch gene. Genetics 177, 1621-1637. |
| ceh-43 | C28A5.4 | Bürglin, T.R. and Aspöck, G. Exon duplication from a fork head to a homeodomain protein. Dev Genes Evol 209, 629-633. |
|  |  | Chamberlin, H.M. and Thomas, J.H. The bromodomain protein LIN-49 and trithorax-related protein LIN-59 affect development and gene expression in Caenorhabditis elegans. Development 127, 713-723. |
|  |  | Aspöck, G. and Bürglin, T.R. The Caenorhabditis elegans distal-less ortholog ceh-43 is required for development of the anterior hypodermis. Dev Dyn 222, 403-409. |
|  |  | Portman, D.S. and Emmons, S.W. Identification of C. elegans sensory ray genes using whole-genome expression profiling. Dev Biol 270, 499-512. |
|  |  | Nelson, C.E., Hersh, B.M. and Carroll, S.B. The regulatory content of intergenic DNA shapes genome architecture. Genome Biol 5, R25. |
|  |  | Kaltenbach, L.S., Updike, D.L. and Mango, S.E. Contribution of the amino and carboxyl termini for PHA-4/FoxA function in Caenorhabditis elegans. Dev Dyn 234, 346-354. |
|  |  | Chisholm, A.D. and Hardin, J. Epidermal morphogenesis. WormBook, 1-22. |
|  |  | Okkema, P.G. and Krause, M. Transcriptional regulation. WormBook, 1-40. |
|  |  | Mango, S.E. The C. elegans pharynx: a model for organogenesis. WormBook, 1-26. |
|  |  | Axang, C., Rauthan, M., Hall, D.H. and Pilon, M. The twisted pharynx phenotype in C. elegans. BMC Dev Biol 7, 61. |
| ceh-44 | Y54F10AM.4 | Bürglin, T.R. and Cassata, G. Loss and gain of domains during evolution of cut superclass homeobox genes. Int J Dev Biol 46, 115-123. |
|  |  | Nelson, C.E., Hersh, B.M. and Carroll, S.B. The regulatory content of intergenic DNA shapes genome architecture. Genome Biol 5, R25. |
|  |  | Okkema, P.G. and Krause, M. Transcriptional regulation. WormBook, 1-40. |
| ceh-45 | ZK993.1 |  |
| ceh-48 | C17H12.9 | Bürglin, T.R. and Cassata, G. Loss and gain of domains during evolution of cut superclass homeobox genes. Int J Dev Biol 46, 115-123. |
|  |  | Okkema, P.G. and Krause, M. Transcriptional regulation. WormBook, 1-40. |
| ceh-49 | F17A9.6 | Bürglin, T.R. and Cassata, G. Loss and gain of domains during evolution of cut superclass homeobox genes. Int J Dev Biol 46, 115-123. |
|  |  | Okkema, P.G. and Krause, M. Transcriptional regulation. WormBook, 1-40. |
| ceh-51 | Y80D3A.3 | Okkema, P.G. and Krause, M. Transcriptional regulation. WormBook, 1-40. |
|  |  | Broitman-Maduro, G., Owraghi, M., Hung, W.W., Kuntz, S., Sternberg, P.W. and Maduro, M.F. The NK-2 class homeodomain factor CEH-51 and the T-box factor TBX-35 have overlapping function in C. elegans mesoderm development. Development 136, 2735-2746. |
|  |  | Owraghi, M., Broitman-Maduro, G., Luu, T., Roberson, H. and Maduro, M.F. Roles of the Wnt effector POP-1/TCF in the C. elegans endomesoderm specification gene network. Dev Biol 340, 209-221. |
| ceh-53 | C09G12.1 | Okkema, P.G. and Krause, M. Transcriptional regulation. WormBook, 1-40. |
| ceh-54 | T13C5.4 | Okkema, P.G. and Krause, M. Transcriptional regulation. WormBook, 1-40. |
| ceh-57 | C07E3.5 |  |
| ceh-58 | C07E3.6 | Okkema, P.G. and Krause, M. Transcriptional regulation. WormBook, 1-40. |
| ceh-60 | F22A3.5 | Okkema, P.G. and Krause, M. Transcriptional regulation. WormBook, 1-40. |
|  |  | Mukherjee, K. and Bürglin, T.R. Comprehensive analysis of animal TALE homeobox genes: new conserved motifs and cases of accelerated evolution. J Mol Evol 65, 137-153. |
| ceh-62 | R06F6.6 | Okkema, P.G. and Krause, M. Transcriptional regulation. WormBook, 1-40. |
| ceh-63 | C02F12.10 |  |
| ceh-74 | ZC376.4 |  |
| ceh-75 | C50H2.6 |  |
| ceh-76 | Y97E10AM.1 |  |
| ceh-79 | C26E1.3 |  |
| ceh-81 | F45C12.3 |  |
| ceh-82 | F45C12.2 |  |
| ceh-83 | F45C12.15 | Okkema, P.G. and Krause, M. Transcriptional regulation. WormBook, 1-40. |
| ceh-84 | C40D2.4 |  |
| ceh-85 | F59H6.6 |  |
| ceh-86 | F42G2.6 | Okkema, P.G. and Krause, M. Transcriptional regulation. WormBook, 1-40. |
|  |  | Kerry, S., TeKippe, M., Gaddis, N.C. and Aballay, A. GATA transcription factor required for immunity to bacterial and fungal pathogens. PLoS One 1, e77. |
| ceh-87 | F34D6.2 |  |
| ceh-88 | C49C3.5 | Okkema, P.G. and Krause, M. Transcriptional regulation. WormBook, 1-40. |
| ceh-89 | F28H6.2 |  |
| ceh-90 | R03E1.4 |  |
| ceh-91 | Y66A7A.5 |  |
| ceh-92 | Y66D12A.5 |  |
| ceh-93 | R04A9.5 | Manning, G. Genomic overview of protein kinases. WormBook, 1-19. |
|  |  | Okkema, P.G. and Krause, M. Transcriptional regulation. WormBook, 1-40. |
| ceh-99 | T21B4.17 |  |
| ceh-100 | Y38E10A.6 |  |
| cog-1 | R03C1.3 | Palmer, R.E., Inoue, T., Sherwood, D.R., Jiang, L.I. and Sternberg, P.W. Caenorhabditis elegans cog-1 locus encodes GTX/Nkx6.1 homeodomain proteins and regulates multiple aspects of reproductive system development. Dev Biol 252, 202-213. |
|  |  | Podbilewicz, B. How does a cell anchor and invade an organ? Dev Cell 5, 5-7. |
|  |  | Chang, S., Johnston, R.J., Jr. and Hobert, O. A transcriptional regulatory cascade that controls left/right asymmetry in chemosensory neurons of C. elegans. Genes Dev 17, 2123-2137. |
|  |  | Johnston, R.J. and Hobert, O. A microRNA controlling left/right neuronal asymmetry in Caenorhabditis elegans. Nature 426, 845-849. |
|  |  | Lanjuin, A. and Sengupta, P. Specification of chemosensory neuron subtype identities in Caenorhabditis elegans. Curr Opin Neurobiol 14, 22-30. |
|  |  | Chang, S., Johnston, R.J., Jr., Frokjaer-Jensen, C., Lockery, S. and Hobert, O. MicroRNAs act sequentially and asymmetrically to control chemosensory laterality in the nematode. Nature 430, 785-789. |
|  |  | Inoue, T., Wang, M., Ririe, T.O., Fernandes, J.S. and Sternberg, P.W. Transcriptional network underlying Caenorhabditis elegans vulval development. Proc Natl Acad Sci U S A 102, 4972-4977. |
|  |  | Johnston, R.J., Jr., Chang, S., Etchberger, J.F., Ortiz, C.O. and Hobert, O. MicroRNAs acting in a double-negative feedback loop to control a neuronal cell fate decision. Proc Natl Acad Sci U S A 102, 12449-12454. |
|  |  | Kim, K., Colosimo, M.E., Yeung, H. and Sengupta, P. The UNC-3 Olf/EBF protein represses alternate neuronal programs to specify chemosensory neuron identity. Dev Biol 286, 136-148. |
|  |  | Johnston, R.J., Jr. and Hobert, O. A novel C. elegans zinc finger transcription factor, lsy-2, required for the cell type-specific expression of the lsy-6 microRNA. Development 132, 5451-5460. |
|  |  | Vella, M.C. and Slack, F.J. C. elegans microRNAs. WormBook, 1-9. |
|  |  | Hobert, O. Specification of the nervous system. WormBook, 1-19. |
|  |  | Okkema, P.G. and Krause, M. Transcriptional regulation. WormBook, 1-40. |
|  |  | Sternberg, P.W. Vulval development. WormBook, 1-28. |
|  |  | Johnston, R.J., Jr., Copeland, J.W., Fasnacht, M., Etchberger, J.F., Liu, J., Honig, B. and Hobert, O. An unusual Zn-finger/FH2 domain protein controls a left/right asymmetric neuronal fate decision in C. elegans. Development 133, 3317-3328. |
|  |  | Didiano, D. and Hobert, O. Perfect seed pairing is not a generally reliable predictor for miRNA-target interactions. Nat Struct Mol Biol 13, 849-851. |
|  |  | Fernandes, J.S. and Sternberg, P.W. The tailless ortholog nhr-67 regulates patterning of gene expression and morphogenesis in the C. elegans vulva. PLoS Genet 3, e69. |
|  |  | Vermeirssen, V., Barrasa, M.I., Hidalgo, C.A., Babon, J.A., Sequerra, R., Doucette-Stamm, L., Barabasi, A.L. and Walhout, A.J. Transcription factor modularity in a gene-centered C. elegans core neuronal protein-DNA interaction network. Genome Res 17, 1061-1071. |
|  |  | Etchberger, J.F., Lorch, A., Sleumer, M.C., Zapf, R., Jones, S.J., Marra, M.A., Holt, R.A., Moerman, D.G. and Hobert, O. The molecular signature and cis-regulatory architecture of a C. elegans gustatory neuron. Genes Dev 21, 1653-1674. |
|  |  | Sarin, S., O'Meara, M.M., Flowers, E.B., Antonio, C., Poole, R.J., Didiano, D., Johnston, R.J., Jr., Chang, S., Narula, S. and Hobert, O. Genetic screens for Caenorhabditis elegans mutants defective in left/right asymmetric neuronal fate specification. Genetics 176, 2109-2130. |
|  |  | Gissendanner, C.R., Kelley, K., Nguyen, T.Q., Hoener, M.C., Sluder, A.E. and Maina, C.V. The Caenorhabditis elegans NR4A nuclear receptor is required for spermatheca morphogenesis. Dev Biol 313, 767-786. |
|  |  | Didiano, D. and Hobert, O. Molecular architecture of a miRNA-regulated 3' UTR. Rna 14, 1297-1317. |
|  |  | Ririe, T.O., Fernandes, J.S. and Sternberg, P.W. The Caenorhabditis elegans vulva: a post-embryonic gene regulatory network controlling organogenesis. Proc Natl Acad Sci U S A 105, 20095-20099. |
|  |  | Ding, X.C. and Grosshans, H. Repression of C. elegans microRNA targets at the initiation level of translation requires GW182 proteins. Embo J 28, 213-222. |
|  |  | O'Meara, M.M., Bigelow, H., Flibotte, S., Etchberger, J.F., Moerman, D.G. and Hobert, O. Cis-regulatory mutations in the Caenorhabditis elegans homeobox gene locus cog-1 affect neuronal development. Genetics 181, 1679-1686. |
|  |  | Hammell, C.M., Lubin, I., Boag, P.R., Blackwell, T.K. and Ambros, V. nhl-2 Modulates microRNA activity in Caenorhabditis elegans. Cell 136, 926-938. |
|  |  | Jovelin, R. Rapid sequence evolution of transcription factors controlling neuron differentiation in Caenorhabditis. Mol Biol Evol 26, 2373-2386. |
|  |  | Sarin, S., Antonio, C., Tursun, B. and Hobert, O. The C. elegans Tailless/TLX transcription factor nhr-67 controls neuronal identity and left/right asymmetric fate diversification. Development 136, 2933-2944. |
|  |  | Inoue, T. and Sternberg, P.W. C. elegans BED domain transcription factor BED-3 controls lineage-specific cell proliferation during organogenesis. Dev Biol 338, 226-236. |
|  |  | Kaufman, E.J. and Miska, E.A. The microRNAs of Caenorhabditis elegans. Semin Cell Dev Biol 21, 728-737. |
| dsc-1 | C18B12.3 | Branicky, R. and Hekimi, S. Specification of muscle neurotransmitter sensitivity by a Paired-like homeodomain protein in Caenorhabditis elegans. Development 132, 4999-5009. |
|  |  | Okkema, P.G. and Krause, M. Transcriptional regulation. WormBook, 1-40. |
|  |  | Etchberger, J.F., Lorch, A., Sleumer, M.C., Zapf, R., Jones, S.J., Marra, M.A., Holt, R.A., Moerman, D.G. and Hobert, O. The molecular signature and cis-regulatory architecture of a C. elegans gustatory neuron. Genes Dev 21, 1653-1674. |
| duxl-1 | ZC204.2 |  |
| dve-1 | ZK1193.5 | Bürglin, T.R. and Cassata, G. Loss and gain of domains during evolution of cut superclass homeobox genes. Int J Dev Biol 46, 115-123. |
|  |  | Okkema, P.G. and Krause, M. Transcriptional regulation. WormBook, 1-40. |
|  |  | Haynes, C.M., Petrova, K., Benedetti, C., Yang, Y. and Ron, D. ClpP mediates activation of a mitochondrial unfolded protein response in C. elegans. Dev Cell 13, 467-480. |
|  |  | Zhang, M., Poplawski, M., Yen, K., Cheng, H., Bloss, E., Zhu, X., Patel, H. and Mobbs, C.V. Role of CBP and SATB-1 in aging, dietary restriction, and insulin-like signaling. PLoS Biol 7, e1000245. |
|  |  | Lee, S.K., Li, W., Ryu, S.E., Rhim, T. and Ahnn, J. Vacuolar (H+)-ATPases in Caenorhabditis elegans: what can we learn about giant H+ pumps from tiny worms? Biochim Biophys Acta 1797, 1687-1695. |
| egl-5 | C08C3.1 | Trent, C., Tsuing, N. and Horvitz, H.R. Egg-laying defective mutants of the nematode Caenorhabditis elegans. Genetics 104, 619-647. |
|  |  | Hedgecock, E.M., Culotti, J.G., Hall, D.H. and Stern, B.D. Genetics of cell and axon migrations in Caenorhabditis elegans. Development 100, 365-382. |
|  |  | Desai, C., Garriga, G., McIntire, S.L. and Horvitz, H.R. A genetic pathway for the development of the Caenorhabditis elegans HSN motor neurons. Nature 336, 638-646. |
|  |  | Chalfie, M. and Au, M. Genetic control of differentiation of the Caenorhabditis elegans touch receptor neurons. Science 243, 1027-1033. |
|  |  | Desai, C. and Horvitz, H.R. Caenorhabditis elegans mutants defective in the functioning of the motor neurons responsible for egg laying. Genetics 121, 703-721. |
|  |  | Bürglin, T.R., Finney, M., Coulson, A. and Ruvkun, G. Caenorhabditis elegans has scores of homoeobox-containing genes. Nature 341, 239-243. |
|  |  | Schaller, D., Wittmann, C., Spicher, A., Muller, F. and Tobler, H. Cloning and analysis of three new homeobox genes from the nematode Caenorhabditis elegans. Nucleic Acids Res 18, 2033-2036. |
|  |  | Hawkins, N.C. and McGhee, J.D. Homeobox containing genes in the nematode Caenorhabditis elegans. Nucleic Acids Res 18, 6101-6106. |
|  |  | Chisholm, A. Control of cell fate in the tail region of C. elegans by the gene egl-5. Development 111, 921-932. |
|  |  | Bürglin, T.R., Ruvkun, G., Coulson, A., Hawkins, N.C., McGhee, J.D., Schaller, D., Wittmann, C., Müller, F. and Waterston, R.H. Nematode homeobox cluster. Nature 351, 703. |
|  |  | Hodgkin, J. The interactive worm. Eighth international C. elegans Meeting, Madison, WI, USA, June 1-5, 1991. New Biol 3, 951-954. |
|  |  | Emmons, S.W. From cell fates to morphology: developmental genetics of the Caenorhabditis elegans male tail. Bioessays 14, 309-316. |
|  |  | Cowing, D.W. and Kenyon, C. Expression of the homeotic gene mab-5 during Caenorhabditis elegans embryogenesis. Development 116, 481-490. |
|  |  | Way, J.C., Run, J.Q. and Wang, A.Y. Regulation of anterior cell-specific mec-3 expression during asymmetric cell division in C. elegans. Dev Dyn 194, 289-302. |
|  |  | Bargmann, C.I. Genetic and cellular analysis of behavior in C. elegans. Annu Rev Neurosci 16, 47-71. |
|  |  | McIntire, S.L., Jorgensen, E., Kaplan, J. and Horvitz, H.R. The GABAergic nervous system of Caenorhabditis elegans. Nature 364, 337-341. |
|  |  | Wang, B.B., Muller-Immergluck, M.M., Austin, J., Robinson, N.T., Chisholm, A. and Kenyon, C. A homeotic gene cluster patterns the anteroposterior body axis of C. elegans. Cell 74, 29-42. |
|  |  | Clark, S.G., Chisholm, A.D. and Horvitz, H.R. Control of cell fates in the central body region of C. elegans by the homeobox gene lin-39. Cell 74, 43-55. |
|  |  | Roehl, H. and Kimble, J. Control of cell fate in C. elegans by a GLP-1 peptide consisting primarily of ankyrin repeats. Nature 364, 632-635. |
|  |  | Salser, S.J., Loer, C.M. and Kenyon, C. Multiple HOM-C gene interactions specify cell fates in the nematode central nervous system. Genes Dev 7, 1714-1724. |
|  |  | Bürglin, T.R. and Ruvkun, G. The Caenorhabditis elegans homeobox gene cluster. Curr Opin Genet Dev 3, 615-620. |
|  |  | Mitani, S., Du, H., Hall, D.H., Driscoll, M. and Chalfie, M. Combinatorial control of touch receptor neuron expression in Caenorhabditis elegans. Development 119, 773-783. |
|  |  | Wood, W.B. and Edgar, L.G. Patterning in the C. elegans embryo. Trends Genet 10, 49-54. |
|  |  | Wilson, R., Ainscough, R., Anderson, K., Baynes, C., Berks, M., Bonfield, J., Burton, J., Connell, M., Copsey, T., Cooper, J. et al. 2.2 Mb of contiguous nucleotide sequence from chromosome III of C. elegans. Nature 368, 32-38. |
|  |  | Austin, J. and Kenyon, C. Cell contact regulates neuroblast formation in the Caenorhabditis elegans lateral epidermis. Development 120, 313-323. |
|  |  | Salser, S.J. and Kenyon, C. Patterning C. elegans: homeotic cluster genes, cell fates and cell migrations. Trends Genet 10, 159-164. |
|  |  | Waterston, R., Ainscough, R., Anderson, K., Berks, M., Blair, D., Connell, M., Cooper, J., Coulson, A., Craxton, M., Dear, S. et al. The genome of the nematode Caenorhabditis elegans. Cold Spring Harb Symp Quant Biol 58, 367-376. |
|  |  | Garriga, G. and Stern, M.J. Hams and Egls: genetic analysis of cell migration in Caenorhabditis elegans. Curr Opin Genet Dev 4, 575-580. |
|  |  | Chow, K.L. and Emmons, S.W. HOM-C/Hox genes and four interacting loci determine the morphogenetic properties of single cells in the nematode male tail. Development 120, 2579-2592. |
|  |  | Duggan, A. and Chalfie, M. Control of neuronal development in Caenorhabditis elegans. Curr Opin Neurobiol 5, 6-9. |
|  |  | Muimo, R. and Isaac, R.E. Metabolism of 5-HT in the nematode Caenorhabditis elegans: identification of a 5-HT N-acetyltransferase that is developmentally regulated. Biochem Soc Trans 21 ( Pt 3), 295S. |
|  |  | Chow, K.L., Hall, D.H. and Emmons, S.W. The mab-21 gene of Caenorhabditis elegans encodes a novel protein required for choice of alternate cell fates. Development 121, 3615-3626. |
|  |  | Sengupta, P. and Bargmann, C.I. Cell fate specification and differentiation in the nervous system of Caenorhabditis elegans. Dev Genet 18, 73-80. |
|  |  | Salser, S.J. and Kenyon, C. A C. elegans Hox gene switches on, off, on and off again to regulate proliferation, differentiation and morphogenesis. Development 122, 1651-1661. |
|  |  | Ahringer, J. Posterior patterning by the Caenorhabditis elegans even-skipped homolog vab-7. Genes Dev 10, 1120-1130. |
|  |  | Baumeister, R., Liu, Y. and Ruvkun, G. Lineage-specific regulators couple cell lineage asymmetry to the transcription of the Caenorhabditis elegans POU gene unc-86 during neurogenesis. Genes Dev 10, 1395-1410. |
|  |  | Emmons, S.W. Simple worms, complex genes. Nature 382, 301-302. |
|  |  | Korswagen, H.C., Durbin, R.M., Smits, M.T. and Plasterk, R.H. Transposon Tc1-derived, sequence-tagged sites in Caenorhabditis elegans as markers for gene mapping. Proc Natl Acad Sci U S A 93, 14680-14685. |
|  |  | Wrischnik, L.A. and Kenyon, C.J. The role of lin-22, a hairy/enhancer of split homolog, in patterning the peripheral nervous system of C. elegans. Development 124, 2875-2888. |
|  |  | Kenyon, C.J., Austin, J., Costa, M., Cowing, D.W., Harris, J.M., Honigberg, L., Hunter, C.P., Maloof, J.N., Muller-Immergluck, M.M., Salser, S.J. et al. The dance of the Hox genes: patterning the anteroposterior body axis of Caenorhabditis elegans. Cold Spring Harb Symp Quant Biol 62, 293-305. |
|  |  | Jiang, L.I. and Sternberg, P.W. Interactions of EGF, Wnt and HOM-C genes specify the P12 neuroectoblast fate in C. elegans. Development 125, 2337-2347. |
|  |  | Harfe, B.D., Vaz Gomes, A., Kenyon, C., Liu, J., Krause, M. and Fire, A. Analysis of a Caenorhabditis elegans Twist homolog identifies conserved and divergent aspects of mesodermal patterning. Genes Dev 12, 2623-2635. |
|  |  | Ruvkun, G. and Hobert, O. The taxonomy of developmental control in Caenorhabditis elegans. Science 282, 2033-2041. |
|  |  | Maloof, J.N., Whangbo, J., Harris, J.M., Jongeward, G.D. and Kenyon, C. A Wnt signaling pathway controls hox gene expression and neuroblast migration in C. elegans. Development 126, 37-49. |
|  |  | Kagoshima, H., Cassata, G. and Bürglin, T.R. A Caenorhabditis elegans homeobox gene expressed in the male tail, a link between pattern formation and sexual dimorphism? Dev Genes Evol 209, 59-62. |
|  |  | Baum, P.D., Guenther, C., Frank, C.A., Pham, B.V. and Garriga, G. The Caenorhabditis elegans gene ham-2 links Hox patterning to migration of the HSN motor neuron. Genes Dev 13, 472-483. |
|  |  | Ferreira, H.B., Zhang, Y., Zhao, C. and Emmons, S.W. Patterning of Caenorhabditis elegans posterior structures by the Abdominal-B homolog, egl-5. Dev Biol 207, 215-228. |
|  |  | Brunschwig, K., Wittmann, C., Schnabel, R., Bürglin, T.R., Tobler, H. and Müller, F. Anterior organization of the Caenorhabditis elegans embryo by the labial-like Hox gene ceh-13. Development 126, 1537-1546. |
|  |  | Baird, S.E. and Ellazar, S.A. TGFbeta-like signaling and spicule development in Caenorhabditis elegans. Dev Biol 212, 93-100. |
|  |  | Montell, D.J. The genetics of cell migration in Drosophila melanogaster and Caenorhabditis elegans development. Development 126, 3035-3046. |
|  |  | Ch'ng, Q. and Kenyon, C. egl-27 generates anteroposterior patterns of cell fusion in C. elegans by regulating Hox gene expression and Hox protein function. Development 126, 3303-3312. |
|  |  | Chamberlin, H.M., Brown, K.B., Sternberg, P.W. and Thomas, J.H. Characterization of seven genes affecting Caenorhabditis elegans hindgut development. Genetics 153, 731-742. |
|  |  | Chow, K.L. and Chan, K.W. Stress-induced phenocopy of C. elegans defines functional steps of sensory organ differentiation. Dev Growth Differ 41, 629-637. |
|  |  | Lints, R. and Emmons, S.W. Patterning of dopaminergic neurotransmitter identity among Caenorhabditis elegans ray sensory neurons by a TGFbeta family signaling pathway and a Hox gene. Development 126, 5819-5831. |
|  |  | Chamberlin, H.M. and Thomas, J.H. The bromodomain protein LIN-49 and trithorax-related protein LIN-59 affect development and gene expression in Caenorhabditis elegans. Development 127, 713-723. |
|  |  | Woollard, A. and Hodgkin, J. The caenorhabditis elegans fate-determining gene mab-9 encodes a T-box protein required to pattern the posterior hindgut. Genes Dev 14, 596-603. |
|  |  | Van Auken, K., Weaver, D.C., Edgar, L.G. and Wood, W.B. Caenorhabditis elegans embryonic axial patterning requires two recently discovered posterior-group Hox genes. Proc Natl Acad Sci U S A 97, 4499-4503. |
|  |  | Zhang, H. and Emmons, S.W. A C. elegans mediator protein confers regulatory selectivity on lineage-specific expression of a transcription factor gene. Genes Dev 14, 2161-2172. |
|  |  | Liu, J. and Fire, A. Overlapping roles of two Hox genes and the exd ortholog ceh-20 in diversification of the C. elegans postembryonic mesoderm. Development 127, 5179-5190. |
|  |  | Zhang, H. and Emmons, S.W. The novel C. elegans gene sop-3 modulates Wnt signaling to regulate Hox gene expression. Development 128, 767-777. |
|  |  | Bürglin, T.R. and Ruvkun, G. Regulation of ectodermal and excretory function by the C. elegans POU homeobox gene ceh-6. Development 128, 779-790. |
|  |  | Alper, S. and Kenyon, C. REF-1, a protein with two bHLH domains, alters the pattern of cell fusion in C. elegans by regulating Hox protein activity. Development 128, 1793-1804. |
|  |  | Kwon, J.Y. and Lee, J. Biological significance of a universally conserved transcription mediator in metazoan developmental signaling pathways. Development 128, 3095-3104. |
|  |  | Cameron, S., Clark, S.G., McDermott, J.B., Aamodt, E. and Horvitz, H.R. PAG-3, a Zn-finger transcription factor, determines neuroblast fate in C. elegans. Development 129, 1763-1774. |
|  |  | Maduro, M.F. and Rothman, J.H. Making worm guts: the gene regulatory network of the Caenorhabditis elegans endoderm. Dev Biol 246, 68-85. |
|  |  | Howard, R.M. and Sundaram, M.V. C. elegans EOR-1/PLZF and EOR-2 positively regulate Ras and Wnt signaling and function redundantly with LIN-25 and the SUR-2 Mediator component. Genes Dev 16, 1815-1827. |
|  |  | Alper, S. and Kenyon, C. The zinc finger protein REF-2 functions with the Hox genes to inhibit cell fusion in the ventral epidermis of C. elegans. Development 129, 3335-3348. |
|  |  | Herman, M.A. Control of cell polarity by noncanonical Wnt signaling in C. elegans. Semin Cell Dev Biol 13, 233-241. |
|  |  | Lints, R. and Emmons, S.W. Regulation of sex-specific differentiation and mating behavior in C. elegans by a new member of the DM domain transcription factor family. Genes Dev 16, 2390-2402. |
|  |  | Aboobaker, A.A. and Blaxter, M.L. Hox Gene Loss during Dynamic Evolution of the Nematode Cluster. Curr Biol 13, 37-40. |
|  |  | Hahn, A.C. and Emmons, S.W. The roles of an ephrin and a semaphorin in patterning cell-cell contacts in C. elegans sensory organ development. Dev Biol 256, 379-388. |
|  |  | Ross, J.M. and Zarkower, D. Polycomb group regulation of Hox gene expression in C. elegans. Dev Cell 4, 891-901. |
|  |  | Zhang, H., Azevedo, R.B., Lints, R., Doyle, C., Teng, Y., Haber, D. and Emmons, S.W. Global regulation of Hox gene expression in C. elegans by a SAM domain protein. Dev Cell 4, 903-915. |
|  |  | Toker, A.S., Teng, Y., Ferreira, H.B., Emmons, S.W. and Chalfie, M. The Caenorhabditis elegans spalt-like gene sem-4 restricts touch cell fate by repressing the selector Hox gene egl-5 and the effector gene mec-3. Development 130, 3831-3840. |
|  |  | Sewell, S.T., Zhang, G., Uttam, A. and Chamberlin, H.M. Developmental patterning in the Caenorhabditis elegans hindgut. Dev Biol 262, 88-93. |
|  |  | Aboobaker, A. and Blaxter, M. Hox gene evolution in nematodes: novelty conserved. Curr Opin Genet Dev 13, 593-598. |
|  |  | Lints, R., Jia, L., Kim, K., Li, C. and Emmons, S.W. Axial patterning of C. elegans male sensilla identities by selector genes. Dev Biol 269, 137-151. |
|  |  | Botas, J. Control of morphogenesis and differentiation by HOM/Hox genes. Curr Opin Cell Biol 5, 1015-1022. |
|  |  | Nicholas, H.R. and Hodgkin, J. The ERK MAP kinase cascade mediates tail swelling and a protective response to rectal infection in C. elegans. Curr Biol 14, 1256-1261. |
|  |  | Teng, Y., Girard, L., Ferreira, H.B., Sternberg, P.W. and Emmons, S.W. Dissection of cis-regulatory elements in the C. elegans Hox gene egl-5 promoter. Dev Biol 276, 476-492. |
|  |  | Thoemke, K., Yi, W., Ross, J.M., Kim, S., Reinke, V. and Zarkower, D. Genome-wide analysis of sex-enriched gene expression during C. elegans larval development. Dev Biol 284, 500-508. |
|  |  | Gravato-Nobre, M.J., Nicholas, H.R., Nijland, R., O'Rourke, D., Whittington, D.E., Yook, K.J. and Hodgkin, J. Multiple genes affect sensitivity of Caenorhabditis elegans to the bacterial pathogen Microbacterium nematophilum. Genetics 171, 1033-1045. |
|  |  | Tiensuu, T., Larsen, M.K., Vernersson, E. and Tuck, S. lin-1 has both positive and negative functions in specifying multiple cell fates induced by Ras/MAP kinase signaling in C. elegans. Dev Biol 286, 338-351. |
|  |  | Zhang, T., Sun, Y., Tian, E., Deng, H., Zhang, Y., Luo, X., Cai, Q., Wang, H., Chai, J. and Zhang, H. RNA-binding proteins SOP-2 and SOR-1 form a novel PcG-like complex in C. elegans. Development 133, 1023-1033. |
|  |  | Goldstein, B., Takeshita, H., Mizumoto, K. and Sawa, H. Wnt signals can function as positional cues in establishing cell polarity. Dev Cell 10, 391-396. |
|  |  | Sundaram, M.V. RTK/Ras/MAPK signaling. WormBook, 1-19. |
|  |  | Podbilewicz, B. Cell fusion. WormBook, 1-32. |
|  |  | Herman, M.A. Hermaphrodite cell-fate specification. WormBook, 1-16. |
|  |  | Emmons, S.W. Male development. WormBook, 1-22. |
|  |  | Hobert, O. Specification of the nervous system. WormBook, 1-19. |
|  |  | Okkema, P.G. and Krause, M. Transcriptional regulation. WormBook, 1-40. |
|  |  | Eisenmann, D.M. Wnt signaling. WormBook, 1-17. |
|  |  | Yang, Y., Sun, Y., Luo, X., Zhang, Y., Chen, Y., Tian, E., Lints, R. and Zhang, H. Polycomb-like genes are necessary for specification of dopaminergic and serotonergic neurons in Caenorhabditis elegans. Proc Natl Acad Sci U S A 104, 852-857. |
|  |  | Deng, H., Sun, Y., Zhang, Y., Luo, X., Hou, W., Yan, L., Chen, Y., Tian, E., Han, J. and Zhang, H. Transcription factor NFY globally represses the expression of the C. elegans Hox gene Abdominal-B homolog egl-5. Dev Biol 308, 583-592. |
|  |  | Cai, Q., Sun, Y., Huang, X., Guo, C., Zhang, Y., Zhu, Z. and Zhang, H. The Caenorhabditis elegans PcG-like gene sop-2 regulates the temporal and sexual specificities of cell fates. Genetics 178, 1445-1456. |
|  |  | Jarriault, S., Schwab, Y. and Greenwald, I. A Caenorhabditis elegans model for epithelial-neuronal transdifferentiation. Proc Natl Acad Sci U S A 105, 3790-3795. |
|  |  | Johnson, R.W. and Chamberlin, H.M. Positive and negative regulatory inputs restrict pax-6/vab-3 transcription to sensory organ precursors in Caenorhabditis elegans. Mech Dev 125, 486-497. |
|  |  | Jiang, Y., Shi, H., Amin, N.M., Sultan, I. and Liu, J. Mesodermal expression of the C. elegans HMX homolog mls-2 requires the PBC homolog CEH-20. Mech Dev 125, 451-461. |
|  |  | Singhvi, A., Frank, C.A. and Garriga, G. The T-box gene tbx-2, the homeobox gene egl-5 and the asymmetric cell division gene ham-1 specify neural fate in the HSN/PHB lineage. Genetics 179, 887-898. |
|  |  | Irazoqui, J.E., Ng, A., Xavier, R.J. and Ausubel, F.M. Role for beta-catenin and HOX transcription factors in Caenorhabditis elegans and mammalian host epithelial-pathogen interactions. Proc Natl Acad Sci U S A 105, 17469-17474. |
|  |  | Van Hoffelen, S. and Herman, M.A. Analysis of Wnt signaling during Caenorhabditis elegans postembryonic development. Methods Mol Biol 469, 87-102. |
|  |  | Nicholas, H.R. and Hodgkin, J. The C. elegans Hox gene egl-5 is required for correct development of the hermaphrodite hindgut and for the response to rectal infection by Microbacterium nematophilum. Dev Biol 329, 16-24. |
|  |  | Zhang, H. and Emmons, S.W. Regulation of the Caenorhabditis elegans posterior Hox gene egl-5 by microRNA and the polycomb-like gene sop-2. Dev Dyn 238, 595-603. |
|  |  | Li, X., Kulkarni, R.P., Hill, R.J. and Chamberlin, H.M. HOM-C genes, Wnt signaling and axial patterning in the C. elegans posterior ventral epidermis. Dev Biol 332, 156-165. |
|  |  | Jovelin, R. Rapid sequence evolution of transcription factors controlling neuron differentiation in Caenorhabditis. Mol Biol Evol 26, 2373-2386. |
|  |  | Zhao, Z., Flibotte, S., Murray, J.I., Blick, D., Boyle, T.J., Gupta, B., Moerman, D.G. and Waterston, R.H. New tools for investigating the comparative biology of Caenorhabditis briggsae and C. elegans. Genetics 184, 853-863. |
|  |  | Kalis, A.K., Kroetz, M.B., Larson, K.M. and Zarkower, D. Functional genomic identification of genes required for male gonadal differentiation in Caenorhabditis elegans. Genetics 185, 523-535. |
|  |  | Hueber, S.D., Weiller, G.F., Djordjevic, M.A. and Frickey, T. Improving Hox protein classification across the major model organisms. PLoS One 5, e10820. |
|  |  | Kalis, A.K., Murphy, M.W. and Zarkower, D. EGL-5/ABD-B plays an instructive role in male cell fate determination in the C. elegans somatic gonad. Dev Biol 344, 827-835. |
| eyg-1 | Y53C12C.1 | Hobert, O. and Ruvkun, G. Pax genes in Caenorhabditis elegans: a new twist. Trends Genet 15, 214-216. |
|  |  | Okkema, P.G. and Krause, M. Transcriptional regulation. WormBook, 1-40. |
| hmbx-1 | F54A5.1 | Lesch, B.J. and Bargmann, C.I. The homeodomain protein hmbx-1 maintains asymmetric gene expression in adult C. elegans olfactory neurons. Genes Dev 24, 1802-1815. |
| irx-1 | C36F7.1 | Bürglin, T.R. Analysis of TALE superclass homeobox genes (MEIS, PBC, KNOX, Iroquois, TGIF) reveals a novel domain conserved between plants and animals. Nucleic Acids Res 25, 4173-4180. |
|  |  | Okkema, P.G. and Krause, M. Transcriptional regulation. WormBook, 1-40. |
|  |  | Mukherjee, K. and Bürglin, T.R. Comprehensive analysis of animal TALE homeobox genes: new conserved motifs and cases of accelerated evolution. J Mol Evol 65, 137-153. |
|  |  | Vavouri, T., Semple, J.I., Garcia-Verdugo, R. and Lehner, B. Intrinsic protein disorder and interaction promiscuity are widely associated with dosage sensitivity. Cell 138, 198-208. |
| lim-4 | ZC64.4 | Sagasti, A., Hobert, O., Troemel, E.R., Ruvkun, G. and Bargmann, C.I. Alternative olfactory neuron fates are specified by the LIM homeobox gene lim-4. Genes Dev 13, 1794-1806. |
|  |  | Sarafi-Reinach, T.R., Melkman, T., Hobert, O. and Sengupta, P. The lin-11 LIM homeobox gene specifies olfactory and chemosensory neuron fates in C. elegans. Development 128, 3269-3281. |
|  |  | Uchida, O., Nakano, H., Koga, M. and Ohshima, Y. The C. elegans che-1 gene encodes a zinc finger transcription factor required for specification of the ASE chemosensory neurons. Development 130, 1215-1224. |
|  |  | Clark, S.G. and Chiu, C. C. elegans ZAG-1, a Zn-finger-homeodomain protein, regulates axonal development and neuronal differentiation. Development 130, 3781-3794. |
|  |  | Lanjuin, A., VanHoven, M.K., Bargmann, C.I., Thompson, J.K. and Sengupta, P. Otx/otd homeobox genes specify distinct sensory neuron identities in C. elegans. Dev Cell 5, 621-633. |
|  |  | Tsalik, E.L., Niacaris, T., Wenick, A.S., Pau, K., Avery, L. and Hobert, O. LIM homeobox gene-dependent expression of biogenic amine receptors in restricted regions of the C. elegans nervous system. Dev Biol 263, 81-102. |
|  |  | Melkman, T. and Sengupta, P. The worm's sense of smell. Development of functional diversity in the chemosensory system of Caenorhabditis elegans. Dev Biol 265, 302-319. |
|  |  | Huang, X., Powell-Coffman, J.A. and Jin, Y. The AHR-1 aryl hydrocarbon receptor and its co-factor the AHA-1 aryl hydrocarbon receptor nuclear translocator specify GABAergic neuron cell fate in C. elegans. Development 131, 819-828. |
|  |  | Lanjuin, A. and Sengupta, P. Specification of chemosensory neuron subtype identities in Caenorhabditis elegans. Curr Opin Neurobiol 14, 22-30. |
|  |  | Kim, K., Colosimo, M.E., Yeung, H. and Sengupta, P. The UNC-3 Olf/EBF protein represses alternate neuronal programs to specify chemosensory neuron identity. Dev Biol 286, 136-148. |
|  |  | Zheng, X., Chung, S., Tanabe, T. and Sze, J.Y. Cell-type specific regulation of serotonergic identity by the C. elegans LIM-homeodomain factor LIM-4. Dev Biol 286, 618-628. |
|  |  | Hobert, O. Specification of the nervous system. WormBook, 1-19. |
|  |  | Okkema, P.G. and Krause, M. Transcriptional regulation. WormBook, 1-40. |
|  |  | van der Linden, A.M., Nolan, K.M. and Sengupta, P. KIN-29 SIK regulates chemoreceptor gene expression via an MEF2 transcription factor and a class II HDAC. Embo J 26, 358-370. |
|  |  | Bargmann, C.I. Chemosensation in C. elegans. WormBook, 1-29. |
|  |  | Torayama, I., Ishihara, T. and Katsura, I. Caenorhabditis elegans integrates the signals of butanone and food to enhance chemotaxis to butanone. J Neurosci 27, 741-750. |
|  |  | Pradel, E., Zhang, Y., Pujol, N., Matsuyama, T., Bargmann, C.I. and Ewbank, J.J. Detection and avoidance of a natural product from the pathogenic bacterium Serratia marcescens by Caenorhabditis elegans. Proc Natl Acad Sci U S A 104, 2295-2300. |
|  |  | Hallem, E.A. and Sternberg, P.W. Acute carbon dioxide avoidance in Caenorhabditis elegans. Proc Natl Acad Sci U S A 105, 8038-8043. |
|  |  | Tsunozaki, M., Chalasani, S.H. and Bargmann, C.I. A behavioral switch: cGMP and PKC signaling in olfactory neurons reverses odor preference in C. elegans. Neuron 59, 959-971. |
|  |  | Jovelin, R. Rapid sequence evolution of transcription factors controlling neuron differentiation in Caenorhabditis. Mol Biol Evol 26, 2373-2386. |
| lim-6 | K03E6.1 | Hobert, O., Tessmar, K. and Ruvkun, G. The Caenorhabditis elegans lim-6 LIM homeobox gene regulates neurite outgrowth and function of particular GABAergic neurons. Development 126, 1547-1562. |
|  |  | Sagasti, A., Hobert, O., Troemel, E.R., Ruvkun, G. and Bargmann, C.I. Alternative olfactory neuron fates are specified by the LIM homeobox gene lim-4. Genes Dev 13, 1794-1806. |
|  |  | Cassata, G., Röhrig, S., Kuhn, F., Hauri, H.P., Baumeister, R. and Bürglin, T.R. The Caenorhabditis elegans Ldb/NLI/Clim orthologue ldb-1 is required for neuronal function. Dev Biol 226, 45-56. |
|  |  | Pierce-Shimomura, J.T., Faumont, S., Gaston, M.R., Pearson, B.J. and Lockery, S.R. The homeobox gene lim-6 is required for distinct chemosensory representations in C. elegans. Nature 410, 694-698. |
|  |  | Hobert, O., Johnston, R.J., Jr. and Chang, S. Left-right asymmetry in the nervous system: the Caenorhabditis elegans model. Nat Rev Neurosci 3, 629-640. |
|  |  | Aurelio, O., Boulin, T. and Hobert, O. Identification of spatial and temporal cues that regulate postembryonic expression of axon maintenance factors in the C. elegans ventral nerve cord. Development 130, 599-610. |
|  |  | Uchida, O., Nakano, H., Koga, M. and Ohshima, Y. The C. elegans che-1 gene encodes a zinc finger transcription factor required for specification of the ASE chemosensory neurons. Development 130, 1215-1224. |
|  |  | Chang, S., Johnston, R.J., Jr. and Hobert, O. A transcriptional regulatory cascade that controls left/right asymmetry in chemosensory neurons of C. elegans. Genes Dev 17, 2123-2137. |
|  |  | Lanjuin, A., VanHoven, M.K., Bargmann, C.I., Thompson, J.K. and Sengupta, P. Otx/otd homeobox genes specify distinct sensory neuron identities in C. elegans. Dev Cell 5, 621-633. |
|  |  | Tsalik, E.L., Niacaris, T., Wenick, A.S., Pau, K., Avery, L. and Hobert, O. LIM homeobox gene-dependent expression of biogenic amine receptors in restricted regions of the C. elegans nervous system. Dev Biol 263, 81-102. |
|  |  | Johnston, R.J. and Hobert, O. A microRNA controlling left/right neuronal asymmetry in Caenorhabditis elegans. Nature 426, 845-849. |
|  |  | Huang, X., Powell-Coffman, J.A. and Jin, Y. The AHR-1 aryl hydrocarbon receptor and its co-factor the AHA-1 aryl hydrocarbon receptor nuclear translocator specify GABAergic neuron cell fate in C. elegans. Development 131, 819-828. |
|  |  | Mehta, N., Loria, P.M. and Hobert, O. A genetic screen for neurite outgrowth mutants in Caenorhabditis elegans reveals a new function for the F-box ubiquitin ligase component LIN-23. Genetics 166, 1253-1267. |
|  |  | Chang, S., Johnston, R.J., Jr., Frokjaer-Jensen, C., Lockery, S. and Hobert, O. MicroRNAs act sequentially and asymmetrically to control chemosensory laterality in the nematode. Nature 430, 785-789. |
|  |  | Liu, T., Zimmerman, K.K. and Patterson, G.I. Regulation of signaling genes by TGFbeta during entry into dauer diapause in C. elegans. BMC Dev Biol 4, 11. |
|  |  | Johnston, R.J., Jr., Chang, S., Etchberger, J.F., Ortiz, C.O. and Hobert, O. MicroRNAs acting in a double-negative feedback loop to control a neuronal cell fate decision. Proc Natl Acad Sci U S A 102, 12449-12454. |
|  |  | Kim, K., Colosimo, M.E., Yeung, H. and Sengupta, P. The UNC-3 Olf/EBF protein represses alternate neuronal programs to specify chemosensory neuron identity. Dev Biol 286, 136-148. |
|  |  | Ortiz, C.O., Etchberger, J.F., Posy, S.L., Frokjaer-Jensen, C., Lockery, S., Honig, B. and Hobert, O. Searching for neuronal left/right asymmetry: genomewide analysis of nematode receptor-type guanylyl cyclases. Genetics 173, 131-149. |
|  |  | Hobert, O. Specification of the nervous system. WormBook, 1-19. |
|  |  | Okkema, P.G. and Krause, M. Transcriptional regulation. WormBook, 1-40. |
|  |  | Johnston, R.J., Jr., Copeland, J.W., Fasnacht, M., Etchberger, J.F., Liu, J., Honig, B. and Hobert, O. An unusual Zn-finger/FH2 domain protein controls a left/right asymmetric neuronal fate decision in C. elegans. Development 133, 3317-3328. |
|  |  | Etchberger, J.F., Lorch, A., Sleumer, M.C., Zapf, R., Jones, S.J., Marra, M.A., Holt, R.A., Moerman, D.G. and Hobert, O. The molecular signature and cis-regulatory architecture of a C. elegans gustatory neuron. Genes Dev 21, 1653-1674. |
|  |  | Sarin, S., O'Meara, M.M., Flowers, E.B., Antonio, C., Poole, R.J., Didiano, D., Johnston, R.J., Jr., Chang, S., Narula, S. and Hobert, O. Genetic screens for Caenorhabditis elegans mutants defective in left/right asymmetric neuronal fate specification. Genetics 176, 2109-2130. |
|  |  | Etchberger, J.F. and Hobert, O. Vector-free DNA constructs improve transgene expression in C. elegans. Nat Methods 5, 3. |
|  |  | Etchberger, J.F., Flowers, E.B., Poole, R.J., Bashllari, E. and Hobert, O. Cis-regulatory mechanisms of left/right asymmetric neuron-subtype specification in C. elegans. Development 136, 147-160. |
|  |  | Jovelin, R. Rapid sequence evolution of transcription factors controlling neuron differentiation in Caenorhabditis. Mol Biol Evol 26, 2373-2386. |
|  |  | Kaufman, E.J. and Miska, E.A. The microRNAs of Caenorhabditis elegans. Semin Cell Dev Biol 21, 728-737. |
| lim-7 | C04F1.3 | Hobert, O., Tessmar, K. and Ruvkun, G. The Caenorhabditis elegans lim-6 LIM homeobox gene regulates neurite outgrowth and function of particular GABAergic neurons. Development 126, 1547-1562. |
|  |  | Sagasti, A., Hobert, O., Troemel, E.R., Ruvkun, G. and Bargmann, C.I. Alternative olfactory neuron fates are specified by the LIM homeobox gene lim-4. Genes Dev 13, 1794-1806. |
|  |  | Hall, D.H., Winfrey, V.P., Blaeuer, G., Hoffman, L.H., Furuta, T., Rose, K.L., Hobert, O. and Greenstein, D. Ultrastructural features of the adult hermaphrodite gonad of Caenorhabditis elegans: relations between the germ line and soma. Dev Biol 212, 101-123. |
|  |  | Blelloch, R., Anna-Arriola, S.S., Gao, D., Li, Y., Hodgkin, J. and Kimble, J. The gon-1 gene is required for gonadal morphogenesis in Caenorhabditis elegans. Dev Biol 216, 382-393. |
|  |  | Hubbard, E.J. and Greenstein, D. The Caenorhabditis elegans gonad: a test tube for cell and developmental biology. Dev Dyn 218, 2-22. |
|  |  | Friedman, L., Santa Anna-Arriola, S., Hodgkin, J. and Kimble, J. gon-4, a cell lineage regulator required for gonadogenesis in Caenorhabditis elegans. Dev Biol 228, 350-362. |
|  |  | Dufourcq, P., Victor, M., Gay, F., Calvo, D., Hodgkin, J. and Shi, Y. Functional requirement for histone deacetylase 1 in Caenorhabditis elegans gonadogenesis. Mol Cell Biol 22, 3024-3034. |
|  |  | Bender, A.M., Wells, O. and Fay, D.S. lin-35/Rb and xnp-1/ATR-X function redundantly to control somatic gonad development in C. elegans. Dev Biol 273, 335-349. |
|  |  | Okkema, P.G. and Krause, M. Transcriptional regulation. WormBook, 1-40. |
|  |  | Voutev, R., Keating, R., Hubbard, E.J. and Vallier, L.G. Characterization of the Caenorhabditis elegans Islet LIM-homeodomain ortholog, lim-7. FEBS Lett 583, 456-464. |
|  |  | Hung, Y.C., Lee, J.H., Chen, H.M. and Huang, G.S. Effects of static magnetic fields on the development and aging of Caenorhabditis elegans. J Exp Biol 213, 2079-2085. |
| lin-11 | ZC247.3 | Trent, C., Tsuing, N. and Horvitz, H.R. Egg-laying defective mutants of the nematode Caenorhabditis elegans. Genetics 104, 619-647. |
|  |  | Ferguson, E.L. and Horvitz, H.R. Identification and characterization of 22 genes that affect the vulval cell lineages of the nematode Caenorhabditis elegans. Genetics 110, 17-72. |
|  |  | Ferguson, E.L., Sternberg, P.W. and Horvitz, H.R. A genetic pathway for the specification of the vulval cell lineages of Caenorhabditis elegans. Nature 326, 259-267. |
|  |  | Freyd, G., Kim, S.K. and Horvitz, H.R. Novel cysteine-rich motif and homeodomain in the product of the Caenorhabditis elegans cell lineage gene lin-11. Nature 344, 876-879. |
|  |  | Sternberg, P.W. Genetic control of cell type and pattern formation in Caenorhabditis elegans. Adv Genet 27, 63-116. |
|  |  | Horvitz, H.R. Genetic control of Caenorhabditis elegans cell lineage. Harvey Lect 84, 65-77. |
|  |  | Horvitz, H.R. and Sternberg, P.W. Multiple intercellular signalling systems control the development of the Caenorhabditis elegans vulva. Nature 351, 535-541. |
|  |  | Hu, E. and Rubin, C.S. Casein kinase II from Caenorhabditis elegans. Cloning, characterization, and developmental regulation of the gene encoding the beta subunit. J Biol Chem 266, 19796-19802. |
|  |  | Li, P.M., Reichert, J., Freyd, G., Horvitz, H.R. and Walsh, C.T. The LIM region of a presumptive Caenorhabditis elegans transcription factor is an iron-sulfur- and zinc-containing metallodomain. Proc Natl Acad Sci U S A 88, 9210-9213. |
|  |  | Zetka, M.C. and Rose, A.M. The meiotic behavior of an inversion in Caenorhabditis elegans. Genetics 131, 321-332. |
|  |  | Naito, M., Kohara, Y. and Kurosawa, Y. Identification of a homeobox-containing gene located between lin-45 and unc-24 on chromosome IV in the nematode Caenorhabditis elegans. Nucleic Acids Res 20, 2967-2969. |
|  |  | Xue, D., Finney, M., Ruvkun, G. and Chalfie, M. Regulation of the mec-3 gene by the C.elegans homeoproteins UNC-86 and MEC-3. Embo J 11, 4969-4979. |
|  |  | Avery, L. The genetics of feeding in Caenorhabditis elegans. Genetics 133, 897-917. |
|  |  | Garriga, G., Desai, C. and Horvitz, H.R. Cell interactions control the direction of outgrowth, branching and fasciculation of the HSN axons of Caenorhabditis elegans. Development 117, 1071-1087. |
|  |  | Clandinin, T.R. and Mains, P.E. Genetic studies of mei-1 gene activity during the transition from meiosis to mitosis in Caenorhabditis elegans. Genetics 134, 199-210. |
|  |  | Chalfie, M. Homeobox genes in Caenorhabditis elegans. Curr Opin Genet Dev 3, 275-277. |
|  |  | Struhl, G., Fitzgerald, K. and Greenwald, I. Intrinsic activity of the Lin-12 and Notch intracellular domains in vivo. Cell 74, 331-345. |
|  |  | Fitzgerald, K., Wilkinson, H.A. and Greenwald, I. glp-1 can substitute for lin-12 in specifying cell fate decisions in Caenorhabditis elegans. Development 119, 1019-1027. |
|  |  | Stenico, M., Lloyd, A.T. and Sharp, P.M. Codon usage in Caenorhabditis elegans: delineation of translational selection and mutational biases. Nucleic Acids Res 22, 2437-2446. |
|  |  | Way, J.C., Wang, L., Run, J.Q. and Hung, M.S. Cell polarity and the mechanism of asymmetric cell division. Bioessays 16, 925-931. |
|  |  | Katz, W.S., Hill, R.J., Clandinin, T.R. and Sternberg, P.W. Different levels of the C. elegans growth factor LIN-3 promote distinct vulval precursor fates. Cell 82, 297-307. |
|  |  | Euling, S. and Ambros, V. Heterochronic genes control cell cycle progress and developmental competence of C. elegans vulva precursor cells. Cell 84, 667-676. |
|  |  | Baumeister, R., Liu, Y. and Ruvkun, G. Lineage-specific regulators couple cell lineage asymmetry to the transcription of the Caenorhabditis elegans POU gene unc-86 during neurogenesis. Genes Dev 10, 1395-1410. |
|  |  | Euling, S. and Ambros, V. Reversal of cell fate determination in Caenorhabditis elegans vulval development. Development 122, 2507-2515. |
|  |  | Newman, A.P. and Sternberg, P.W. Coordinated morphogenesis of epithelia during development of the Caenorhabditis elegans uterine-vulval connection. Proc Natl Acad Sci U S A 93, 9329-9333. |
|  |  | Hobert, O., D'Alberti, T., Liu, Y. and Ruvkun, G. Control of neural development and function in a thermoregulatory network by the LIM homeobox gene lin-11. J Neurosci 18, 2084-2096. |
|  |  | Hanna-Rose, W. and Han, M. COG-2, a sox domain protein necessary for establishing a functional vulval-uterine connection in Caenorhabditis elegans. Development 126, 169-179. |
|  |  | Hobert, O., Tessmar, K. and Ruvkun, G. The Caenorhabditis elegans lim-6 LIM homeobox gene regulates neurite outgrowth and function of particular GABAergic neurons. Development 126, 1547-1562. |
|  |  | Sagasti, A., Hobert, O., Troemel, E.R., Ruvkun, G. and Bargmann, C.I. Alternative olfactory neuron fates are specified by the LIM homeobox gene lim-4. Genes Dev 13, 1794-1806. |
|  |  | Hsieh, J., Liu, J., Kostas, S.A., Chang, C., Sternberg, P.W. and Fire, A. The RING finger/B-box factor TAM-1 and a retinoblastoma-like protein LIN-35 modulate context-dependent gene silencing in Caenorhabditis elegans. Genes Dev 13, 2958-2970. |
|  |  | Newman, A.P., Acton, G.Z., Hartwieg, E., Horvitz, H.R. and Sternberg, P.W. The lin-11 LIM domain transcription factor is necessary for morphogenesis of C. elegans uterine cells. Development 126, 5319-5326. |
|  |  | Mori, I. Genetics of chemotaxis and thermotaxis in the nematode Caenorhabditis elegans. Annu Rev Genet 33, 399-422. |
|  |  | Cassata, G., Kagoshima, H., Andachi, Y., Kohara, Y., Dürrenberger, M.B., Hall, D.H. and Bürglin, T.R. The LIM homeobox gene ceh-14 confers thermosensory function to the AFD neurons in Caenorhabditis elegans. Neuron 25, 587-597. |
|  |  | Hubbard, E.J. and Greenstein, D. The Caenorhabditis elegans gonad: a test tube for cell and developmental biology. Dev Dyn 218, 2-22. |
|  |  | Cassata, G., Röhrig, S., Kuhn, F., Hauri, H.P., Baumeister, R. and Bürglin, T.R. The Caenorhabditis elegans Ldb/NLI/Clim orthologue ldb-1 is required for neuronal function. Dev Biol 226, 45-56. |
|  |  | Fay, D.S. and Han, M. Mutations in cye-1, a Caenorhabditis elegans cyclin E homolog, reveal coordination between cell-cycle control and vulval development. Development 127, 4049-4060. |
|  |  | Fraser, A.G., Kamath, R.S., Zipperlen, P., Martinez-Campos, M., Sohrmann, M. and Ahringer, J. Functional genomic analysis of C. elegans chromosome I by systematic RNA interference. Nature 408, 325-330. |
|  |  | Newman, A.P., Inoue, T., Wang, M. and Sternberg, P.W. The Caenorhabditis elegans heterochronic gene lin-29 coordinates the vulval-uterine-epidermal connections. Curr Biol 10, 1479-1488. |
|  |  | Sarafi-Reinach, T.R., Melkman, T., Hobert, O. and Sengupta, P. The lin-11 LIM homeobox gene specifies olfactory and chemosensory neuron fates in C. elegans. Development 128, 3269-3281. |
|  |  | Michaux, G., Legouis, R. and Labouesse, M. Epithelial biology: lessons from Caenorhabditis elegans. Gene 277, 83-100. |
|  |  | Fay, D.S., Keenan, S. and Han, M. fzr-1 and lin-35/Rb function redundantly to control cell proliferation in C. elegans as revealed by a nonbiased synthetic screen. Genes Dev 16, 503-517. |
|  |  | Gupta, B.P. and Sternberg, P.W. Tissue-specific regulation of the LIM homeobox gene lin-11 during development of the Caenorhabditis elegans egg-laying system. Dev Biol 247, 102-115. |
|  |  | Thor, S. and Thomas, J.B. Motor neuron specification in worms, flies and mice: conserved and 'lost' mechanisms. Curr Opin Genet Dev 12, 558-564. |
|  |  | Hwang, H.Y. and Horvitz, H.R. The Caenorhabditis elegans vulval morphogenesis gene sqv-4 encodes a UDP-glucose dehydrogenase that is temporally and spatially regulated. Proc Natl Acad Sci U S A 99, 14224-14229. |
|  |  | Hurd, D.D. and Kemphues, K.J. PAR-1 is required for morphogenesis of the Caenorhabditis elegans vulva. Dev Biol 253, 54-65. |
|  |  | Gupta, B.P., Wang, M. and Sternberg, P.W. The C. elegans LIM homeobox gene lin-11 specifies multiple cell fates during vulval development. Development 130, 2589-2601. |
|  |  | Podbilewicz, B. How does a cell anchor and invade an organ? Dev Cell 5, 5-7. |
|  |  | Sze, J.Y. and Ruvkun, G. Activity of the Caenorhabditis elegans UNC-86 POU transcription factor modulates olfactory sensitivity. Proc Natl Acad Sci U S A 100, 9560-9565. |
|  |  | Yamada, Y. and Ohshima, Y. Distribution and movement of Caenorhabditis elegans on a thermal gradient. J Exp Biol 206, 2581-2593. |
|  |  | Chang, S., Johnston, R.J., Jr. and Hobert, O. A transcriptional regulatory cascade that controls left/right asymmetry in chemosensory neurons of C. elegans. Genes Dev 17, 2123-2137. |
|  |  | Ailion, M. and Thomas, J.H. Isolation and characterization of high-temperature-induced Dauer formation mutants in Caenorhabditis elegans. Genetics 165, 127-144. |
|  |  | Clark, S.G. and Chiu, C. C. elegans ZAG-1, a Zn-finger-homeodomain protein, regulates axonal development and neuronal differentiation. Development 130, 3781-3794. |
|  |  | Tsalik, E.L., Niacaris, T., Wenick, A.S., Pau, K., Avery, L. and Hobert, O. LIM homeobox gene-dependent expression of biogenic amine receptors in restricted regions of the C. elegans nervous system. Dev Biol 263, 81-102. |
|  |  | Hutter, H. Extracellular cues and pioneers act together to guide axons in the ventral cord of C. elegans. Development 130, 5307-5318. |
|  |  | Cinar, H.N., Richards, K.L., Oommen, K.S. and Newman, A.P. The EGL-13 SOX domain transcription factor affects the uterine pi cell lineages in Caenorhabditis elegans. Genetics 165, 1623-1628. |
|  |  | Melkman, T. and Sengupta, P. The worm's sense of smell. Development of functional diversity in the chemosensory system of Caenorhabditis elegans. Dev Biol 265, 302-319. |
|  |  | Lanjuin, A. and Sengupta, P. Specification of chemosensory neuron subtype identities in Caenorhabditis elegans. Curr Opin Neurobiol 14, 22-30. |
|  |  | Shen, K., Fetter, R.D. and Bargmann, C.I. Synaptic specificity is generated by the synaptic guidepost protein SYG-2 and its receptor, SYG-1. Cell 116, 869-881. |
|  |  | Zhang, S., Sokolchik, I., Blanco, G. and Sze, J.Y. Caenorhabditis elegans TRPV ion channel regulates 5HT biosynthesis in chemosensory neurons. Development 131, 1629-1638. |
|  |  | Chen, Z., Eastburn, D.J. and Han, M. The Caenorhabditis elegans nuclear receptor gene nhr-25 regulates epidermal cell development. Mol Cell Biol 24, 7345-7358. |
|  |  | Broday, L., Kolotuev, I., Didier, C., Bhoumik, A., Gupta, B.P., Sternberg, P.W., Podbilewicz, B. and Ronai, Z. The small ubiquitin-like modifier (SUMO) is required for gonadal and uterine-vulval morphogenesis in Caenorhabditis elegans. Genes Dev 18, 2380-2391. |
|  |  | Lalanne, A.I., Britos, L., Ehrlich, R. and Castillo, E. Mesocestoides corti: a LIM-homeobox gene upregulated during strobilar development. Exp Parasitol 108, 169-175. |
|  |  | Melkman, T. and Sengupta, P. Regulation of chemosensory and GABAergic motor neuron development by the C. elegans Aristaless/Arx homolog alr-1. Development 132, 1935-1949. |
|  |  | Inoue, T., Wang, M., Ririe, T.O., Fernandes, J.S. and Sternberg, P.W. Transcriptional network underlying Caenorhabditis elegans vulval development. Proc Natl Acad Sci U S A 102, 4972-4977. |
|  |  | Sha, K. and Fire, A. Imprinting capacity of gamete lineages in Caenorhabditis elegans. Genetics 170, 1633-1652. |
|  |  | Hutter, H., Wacker, I., Schmid, C. and Hedgecock, E.M. Novel genes controlling ventral cord asymmetry and navigation of pioneer axons in C. elegans. Dev Biol 284, 260-272. |
|  |  | Zheng, X., Chung, S., Tanabe, T. and Sze, J.Y. Cell-type specific regulation of serotonergic identity by the C. elegans LIM-homeodomain factor LIM-4. Dev Biol 286, 618-628. |
|  |  | Nelms, B.L. and Hanna-Rose, W. C. elegans HIM-8 functions outside of meiosis to antagonize EGL-13 Sox protein function. Dev Biol 293, 392-402. |
|  |  | Schmid, C., Schwarz, V. and Hutter, H. AST-1, a novel ETS-box transcription factor, controls axon guidance and pharynx development in C. elegans. Dev Biol 293, 403-413. |
|  |  | Podbilewicz, B. Cell fusion. WormBook, 1-32. |
|  |  | Hobert, O. Specification of the nervous system. WormBook, 1-19. |
|  |  | Okkema, P.G. and Krause, M. Transcriptional regulation. WormBook, 1-40. |
|  |  | Sternberg, P.W. Vulval development. WormBook, 1-28. |
|  |  | Rajakumar, V. and Chamberlin, H.M. The Pax2/5/8 gene egl-38 coordinates organogenesis of the C. elegans egg-laying system. Dev Biol 301, 240-253. |
|  |  | Boulin, T., Pocock, R. and Hobert, O. A novel Eph receptor-interacting IgSF protein provides C. elegans motoneurons with midline guidepost function. Curr Biol 16, 1871-1883. |
|  |  | Fernandes, J.S. and Sternberg, P.W. The tailless ortholog nhr-67 regulates patterning of gene expression and morphogenesis in the C. elegans vulva. PLoS Genet 3, e69. |
|  |  | Oommen, K.S. and Newman, A.P. Co-regulation by Notch and Fos is required for cell fate specification of intermediate precursors during C. elegans uterine development. Development 134, 3999-4009. |
|  |  | Hallem, E.A. and Sternberg, P.W. Acute carbon dioxide avoidance in Caenorhabditis elegans. Proc Natl Acad Sci U S A 105, 8038-8043. |
|  |  | Marri, S. and Gupta, B.P. Dissection of lin-11 enhancer regions in Caenorhabditis elegans and other nematodes. Dev Biol 325, 402-411. |
|  |  | Ririe, T.O., Fernandes, J.S. and Sternberg, P.W. The Caenorhabditis elegans vulva: a post-embryonic gene regulatory network controlling organogenesis. Proc Natl Acad Sci U S A 105, 20095-20099. |
|  |  | Kolotuev, I. and Podbilewicz, B. Changing of the cell division axes drives vulva evolution in nematodes. Dev Biol 313, 142-154. |
|  |  | Jovelin, R. Rapid sequence evolution of transcription factors controlling neuron differentiation in Caenorhabditis. Mol Biol Evol 26, 2373-2386. |
|  |  | Klerkx, E.P., Alarcon, P., Waters, K., Reinke, V., Sternberg, P.W. and Askjaer, P. Protein kinase VRK-1 regulates cell invasion and EGL-17/FGF signaling in Caenorhabditis elegans. Dev Biol 335, 12-21. |
| lin-39 | C07H6.7 | Li, C. and Chalfie, M. Organogenesis in C. elegans: positioning of neurons and muscles in the egg-laying system. Neuron 4, 681-695. |
|  |  | Bürglin, T.R., Ruvkun, G., Coulson, A., Hawkins, N.C., McGhee, J.D., Schaller, D., Wittmann, C., Müller, F. and Waterston, R.H. Nematode homeobox cluster. Nature 351, 703. |
|  |  | Hodgkin, J. The interactive worm. Eighth international C. elegans Meeting, Madison, WI, USA, June 1-5, 1991. New Biol 3, 951-954. |
|  |  | Naito, M., Kohara, Y. and Kurosawa, Y. Identification of a homeobox-containing gene located between lin-45 and unc-24 on chromosome IV in the nematode Caenorhabditis elegans. Nucleic Acids Res 20, 2967-2969. |
|  |  | Garriga, G., Desai, C. and Horvitz, H.R. Cell interactions control the direction of outgrowth, branching and fasciculation of the HSN axons of Caenorhabditis elegans. Development 117, 1071-1087. |
|  |  | Bargmann, C.I. Death from natural and unnatural causes. Curr Biol 1, 388-390. |
|  |  | Wang, B.B., Muller-Immergluck, M.M., Austin, J., Robinson, N.T., Chisholm, A. and Kenyon, C. A homeotic gene cluster patterns the anteroposterior body axis of C. elegans. Cell 74, 29-42. |
|  |  | Clark, S.G., Chisholm, A.D. and Horvitz, H.R. Control of cell fates in the central body region of C. elegans by the homeobox gene lin-39. Cell 74, 43-55. |
|  |  | Salser, S.J., Loer, C.M. and Kenyon, C. Multiple HOM-C gene interactions specify cell fates in the nematode central nervous system. Genes Dev 7, 1714-1724. |
|  |  | Bürglin, T.R. and Ruvkun, G. The Caenorhabditis elegans homeobox gene cluster. Curr Opin Genet Dev 3, 615-620. |
|  |  | Mitani, S., Du, H., Hall, D.H., Driscoll, M. and Chalfie, M. Combinatorial control of touch receptor neuron expression in Caenorhabditis elegans. Development 119, 773-783. |
|  |  | Loer, C.M. and Kenyon, C.J. Serotonin-deficient mutants and male mating behavior in the nematode Caenorhabditis elegans. J Neurosci 13, 5407-5417. |
|  |  | Wood, W.B. and Edgar, L.G. Patterning in the C. elegans embryo. Trends Genet 10, 49-54. |
|  |  | Wilson, R., Ainscough, R., Anderson, K., Baynes, C., Berks, M., Bonfield, J., Burton, J., Connell, M., Copsey, T., Cooper, J. et al. 2.2 Mb of contiguous nucleotide sequence from chromosome III of C. elegans. Nature 368, 32-38. |
|  |  | Austin, J. and Kenyon, C. Cell contact regulates neuroblast formation in the Caenorhabditis elegans lateral epidermis. Development 120, 313-323. |
|  |  | Salser, S.J. and Kenyon, C. Patterning C. elegans: homeotic cluster genes, cell fates and cell migrations. Trends Genet 10, 159-164. |
|  |  | Garriga, G. and Stern, M.J. Hams and Egls: genetic analysis of cell migration in Caenorhabditis elegans. Curr Opin Genet Dev 4, 575-580. |
|  |  | Stenico, M., Lloyd, A.T. and Sharp, P.M. Codon usage in Caenorhabditis elegans: delineation of translational selection and mutational biases. Nucleic Acids Res 22, 2437-2446. |
|  |  | Sommer, R.J., Carta, L.K. and Sternberg, P.W. The evolution of cell lineage in nematodes. Dev Suppl, 85-95. |
|  |  | Duggan, A. and Chalfie, M. Control of neuronal development in Caenorhabditis elegans. Curr Opin Neurobiol 5, 6-9. |
|  |  | Hunter, C.P. and Kenyon, C. Specification of anteroposterior cell fates in Caenorhabditis elegans by Drosophila Hox proteins. Nature 377, 229-232. |
|  |  | Weinshenker, D., Garriga, G. and Thomas, J.H. Genetic and pharmacological analysis of neurotransmitters controlling egg laying in C. elegans. J Neurosci 15, 6975-6985. |
|  |  | Sengupta, P. and Bargmann, C.I. Cell fate specification and differentiation in the nervous system of Caenorhabditis elegans. Dev Genet 18, 73-80. |
|  |  | Miura, M. and Yuan, J. Mechanisms of programmed cell death in Caenorhabditis elegans and vertebrates. Curr Top Dev Biol 32, 139-174. |
|  |  | Salser, S.J. and Kenyon, C. A C. elegans Hox gene switches on, off, on and off again to regulate proliferation, differentiation and morphogenesis. Development 122, 1651-1661. |
|  |  | Baumeister, R., Liu, Y. and Ruvkun, G. Lineage-specific regulators couple cell lineage asymmetry to the transcription of the Caenorhabditis elegans POU gene unc-86 during neurogenesis. Genes Dev 10, 1395-1410. |
|  |  | Harris, J., Honigberg, L., Robinson, N. and Kenyon, C. Neuronal cell migration in C. elegans: regulation of Hox gene expression and cell position. Development 122, 3117-3131. |
|  |  | Clandinin, T.R., Katz, W.S. and Sternberg, P.W. Caenorhabditis elegans HOM-C genes regulate the response of vulval precursor cells to inductive signal. Dev Biol 182, 150-161. |
|  |  | Sommer, R.J. Evolution and development--the nematode vulva as a case study. Bioessays 19, 225-231. |
|  |  | Wrischnik, L.A. and Kenyon, C.J. The role of lin-22, a hairy/enhancer of split homolog, in patterning the peripheral nervous system of C. elegans. Development 124, 2875-2888. |
|  |  | Sternberg, P.W. and Felix, M.A. Evolution of cell lineage. Curr Opin Genet Dev 7, 543-550. |
|  |  | Eizinger, A. and Sommer, R.J. The homeotic gene lin-39 and the evolution of nematode epidermal cell fates. Science 278, 452-455. |
|  |  | Maloof, J.N. and Kenyon, C. The Hox gene lin-39 is required during C. elegans vulval induction to select the outcome of Ras signaling. Development 125, 181-190. |
|  |  | Felix, M.A. and Sternberg, P.W. A gonad-derived survival signal for vulval precursor cells in two nematode species. Curr Biol 8, 287-290. |
|  |  | Burr, A.H. and Gans, C. Mechanical significance of obliquely striated architecture in nematode muscle. Biol Bull 194, 1-6. |
|  |  | Burdine, R.D., Branda, C.S. and Stern, M.J. EGL-17(FGF) expression coordinates the attraction of the migrating sex myoblasts with vulval induction in C. elegans. Development 125, 1083-1093. |
|  |  | Kenyon, C.J., Austin, J., Costa, M., Cowing, D.W., Harris, J.M., Honigberg, L., Hunter, C.P., Maloof, J.N., Muller-Immergluck, M.M., Salser, S.J. et al. The dance of the Hox genes: patterning the anteroposterior body axis of Caenorhabditis elegans. Cold Spring Harb Symp Quant Biol 62, 293-305. |
|  |  | Harfe, B.D., Vaz Gomes, A., Kenyon, C., Liu, J., Krause, M. and Fire, A. Analysis of a Caenorhabditis elegans Twist homolog identifies conserved and divergent aspects of mesodermal patterning. Genes Dev 12, 2623-2635. |
|  |  | Eisenmann, D.M., Maloof, J.N., Simske, J.S., Kenyon, C. and Kim, S.K. The beta-catenin homolog BAR-1 and LET-60 Ras coordinately regulate the Hox gene lin-39 during Caenorhabditis elegans vulval development. Development 125, 3667-3680. |
|  |  | Sommer, R.J., Eizinger, A., Lee, K.Z., Jungblut, B., Bubeck, A. and Schlak, I. The Pristionchus HOX gene Ppa-lin-39 inhibits programmed cell death to specify the vulva equivalence group and is not required during vulval induction. Development 125, 3865-3873. |
|  |  | Ruvkun, G. and Hobert, O. The taxonomy of developmental control in Caenorhabditis elegans. Science 282, 2033-2041. |
|  |  | Nilsson, L., Li, X., Tiensuu, T., Auty, R., Greenwald, I. and Tuck, S. Caenorhabditis elegans lin-25: cellular focus, protein expression and requirement for sur-2 during induction of vulval fates. Development 125, 4809-4819. |
|  |  | Schroeder, D.F. and McGhee, J.D. Anterior-posterior patterning within the Caenorhabditis elegans endoderm. Development 125, 4877-4887. |
|  |  | Maloof, J.N., Whangbo, J., Harris, J.M., Jongeward, G.D. and Kenyon, C. A Wnt signaling pathway controls hox gene expression and neuroblast migration in C. elegans. Development 126, 37-49. |
|  |  | Ferreira, H.B., Zhang, Y., Zhao, C. and Emmons, S.W. Patterning of Caenorhabditis elegans posterior structures by the Abdominal-B homolog, egl-5. Dev Biol 207, 215-228. |
|  |  | Brunschwig, K., Wittmann, C., Schnabel, R., Bürglin, T.R., Tobler, H. and Müller, F. Anterior organization of the Caenorhabditis elegans embryo by the labial-like Hox gene ceh-13. Development 126, 1537-1546. |
|  |  | Sym, M., Robinson, N. and Kenyon, C. MIG-13 positions migrating cells along the anteroposterior body axis of C. elegans. Cell 98, 25-36. |
|  |  | Sigrist, C.B. and Sommer, R.J. Vulva formation in Pristionchus pacificus relies on continuous gonadal induction. Dev Genes Evol 209, 451-459. |
|  |  | Ch'ng, Q. and Kenyon, C. egl-27 generates anteroposterior patterns of cell fusion in C. elegans by regulating Hox gene expression and Hox protein function. Development 126, 3303-3312. |
|  |  | Walhout, A.J., Sordella, R., Lu, X., Hartley, J.L., Temple, G.F., Brasch, M.A., Thierry-Mieg, N. and Vidal, M. Protein interaction mapping in C. elegans using proteins involved in vulval development. Science 287, 116-122. |
|  |  | Hoier, E.F., Mohler, W.A., Kim, S.K. and Hajnal, A. The Caenorhabditis elegans APC-related gene apr-1 is required for epithelial cell migration and Hox gene expression. Genes Dev 14, 874-886. |
|  |  | Shemer, G. and Podbilewicz, B. Fusomorphogenesis: cell fusion in organ formation. Dev Dyn 218, 30-51. |
|  |  | Shemer, G., Kishore, R. and Podbilewicz, B. Ring formation drives invagination of the vulva in Caenorhabditis elegans: Ras, cell fusion, and cell migration determine structural fates. Dev Biol 221, 233-248. |
|  |  | Grant, K., Hanna-Rose, W. and Han, M. sem-4 promotes vulval cell-fate determination in Caenorhabditis elegans through regulation of lin-39 Hox. Dev Biol 224, 496-506. |
|  |  | Hermann, G.J., Leung, B. and Priess, J.R. Left-right asymmetry in C. elegans intestine organogenesis involves a LIN-12/Notch signaling pathway. Development 127, 3429-3440. |
|  |  | Nilsson, L., Tiensuu, T. and Tuck, S. Caenorhabditis elegans lin-25: a study of its role in multiple cell fate specification events involving Ras and the identification and characterization of evolutionarily conserved domains. Genetics 156, 1083-1096. |
|  |  | Liu, J. and Fire, A. Overlapping roles of two Hox genes and the exd ortholog ceh-20 in diversification of the C. elegans postembryonic mesoderm. Development 127, 5179-5190. |
|  |  | Alper, S. and Kenyon, C. REF-1, a protein with two bHLH domains, alters the pattern of cell fusion in C. elegans by regulating Hox protein activity. Development 128, 1793-1804. |
|  |  | Grandien, K. and Sommer, R.J. Functional comparison of the nematode Hox gene lin-39 in C. elegans and P. pacificus reveals evolutionary conservation of protein function despite divergence of primary sequences. Genes Dev 15, 2161-2172. |
|  |  | Michaux, G., Legouis, R. and Labouesse, M. Epithelial biology: lessons from Caenorhabditis elegans. Gene 277, 83-100. |
|  |  | Chen, Z. and Han, M. C. elegans Rb, NuRD, and Ras regulate lin-39-mediated cell fusion during vulval fate specification. Curr Biol 11, 1874-1879. |
|  |  | Chen, Z. and Han, M. Role of C. elegans lin-40 MTA in vulval fate specification and morphogenesis. Development 128, 4911-4921. |
|  |  | Lambie, E.J. Cell proliferation and growth in C. elegans. Bioessays 24, 38-53. |
|  |  | Kostas, S.A. and Fire, A. The T-box factor MLS-1 acts as a molecular switch during specification of nonstriated muscle in C. elegans. Genes Dev 16, 257-269. |
|  |  | Cameron, S., Clark, S.G., McDermott, J.B., Aamodt, E. and Horvitz, H.R. PAG-3, a Zn-finger transcription factor, determines neuroblast fate in C. elegans. Development 129, 1763-1774. |
|  |  | Gleason, J.E., Korswagen, H.C. and Eisenmann, D.M. Activation of Wnt signaling bypasses the requirement for RTK/Ras signaling during C. elegans vulval induction. Genes Dev 16, 1281-1290. |
|  |  | Howard, R.M. and Sundaram, M.V. C. elegans EOR-1/PLZF and EOR-2 positively regulate Ras and Wnt signaling and function redundantly with LIN-25 and the SUR-2 Mediator component. Genes Dev 16, 1815-1827. |
|  |  | Alper, S. and Kenyon, C. The zinc finger protein REF-2 functions with the Hox genes to inhibit cell fusion in the ventral epidermis of C. elegans. Development 129, 3335-3348. |
|  |  | Korswagen, H.C. Canonical and non-canonical Wnt signaling pathways in Caenorhabditis elegans: variations on a common signaling theme. Bioessays 24, 801-810. |
|  |  | Herman, M.A. Control of cell polarity by noncanonical Wnt signaling in C. elegans. Semin Cell Dev Biol 13, 233-241. |
|  |  | Bulik, D.A. and Robbins, P.W. The Caenorhabditis elegans sqv genes and functions of proteoglycans in development. Biochim Biophys Acta 1573, 247-257. |
|  |  | Koh, K., Peyrot, S.M., Wood, C.G., Wagmaister, J.A., Maduro, M.F., Eisenmann, D.M. and Rothman, J.H. Cell fates and fusion in the C. elegans vulval primordium are regulated by the EGL-18 and ELT-6 GATA factors -- apparent direct targets of the LIN-39 Hox protein. Development 129, 5171-5180. |
|  |  | Kamath, R.S., Fraser, A.G., Dong, Y., Poulin, G., Durbin, R., Gotta, M., Kanapin, A., Le Bot, N., Moreno, S., Sohrmann, M. et al. Systematic functional analysis of the Caenorhabditis elegans genome using RNAi. Nature 421, 231-237. |
|  |  | Aboobaker, A.A. and Blaxter, M.L. Hox Gene Loss during Dynamic Evolution of the Nematode Cluster. Curr Biol 13, 37-40. |
|  |  | Louvet-Vallee, S., Kolotuev, I., Podbilewicz, B. and Felix, M.A. Control of vulval competence and centering in the nematode Oscheius sp. 1 CEW1. Genetics 163, 133-146. |
|  |  | Moghal, N. and Sternberg, P.W. The epidermal growth factor system in Caenorhabditis elegans. Exp Cell Res 284, 150-159. |
|  |  | Cui, M. and Han, M. Cis regulatory requirements for vulval cell-specific expression of the Caenorhabditis elegans fibroblast growth factor gene egl-17. Dev Biol 257, 104-116. |
|  |  | Ross, J.M. and Zarkower, D. Polycomb group regulation of Hox gene expression in C. elegans. Dev Cell 4, 891-901. |
|  |  | Zhang, H., Azevedo, R.B., Lints, R., Doyle, C., Teng, Y., Haber, D. and Emmons, S.W. Global regulation of Hox gene expression in C. elegans by a SAM domain protein. Dev Cell 4, 903-915. |
|  |  | Chang, S., Johnston, R.J., Jr. and Hobert, O. A transcriptional regulatory cascade that controls left/right asymmetry in chemosensory neurons of C. elegans. Genes Dev 17, 2123-2137. |
|  |  | Toker, A.S., Teng, Y., Ferreira, H.B., Emmons, S.W. and Chalfie, M. The Caenorhabditis elegans spalt-like gene sem-4 restricts touch cell fate by repressing the selector Hox gene egl-5 and the effector gene mec-3. Development 130, 3831-3840. |
|  |  | Aboobaker, A. and Blaxter, M. Hox gene evolution in nematodes: novelty conserved. Curr Opin Genet Dev 13, 593-598. |
|  |  | Koh, K., Bernstein, Y. and Sundaram, M.V. The nT1 translocation separates vulval regulatory elements from the egl-18 and elt-6 GATA factor genes. Dev Biol 267, 252-263. |
|  |  | Botas, J. Control of morphogenesis and differentiation by HOM/Hox genes. Curr Opin Cell Biol 5, 1015-1022. |
|  |  | Chen, Z., Eastburn, D.J. and Han, M. The Caenorhabditis elegans nuclear receptor gene nhr-25 regulates epidermal cell development. Mol Cell Biol 24, 7345-7358. |
|  |  | Yang, L., Sym, M. and Kenyon, C. The roles of two C. elegans HOX co-factor orthologs in cell migration and vulva development. Development 132, 1413-1428. |
|  |  | Yoda, A., Kouike, H., Okano, H. and Sawa, H. Components of the transcriptional Mediator complex are required for asymmetric cell division in C. elegans. Development 132, 1885-1893. |
|  |  | Felix, M.A. An inversion in the wiring of an intercellular signal: evolution of Wnt signaling in the nematode vulva. Bioessays 27, 765-769. |
|  |  | Gilleard, J.S. The use of Caenorhabditis elegans in parasitic nematode research. Parasitology 128 Suppl 1, S49-70. |
|  |  | Wagmaister, J.A., Gleason, J.E. and Eisenmann, D.M. Transcriptional upregulation of the C. elegans Hox gene lin-39 during vulval cell fate specification. Mech Dev 123, 135-150. |
|  |  | Sundaram, M.V. RTK/Ras/MAPK signaling. WormBook, 1-19. |
|  |  | Podbilewicz, B. Cell fusion. WormBook, 1-32. |
|  |  | Sommer, R.J. Evolution of development in nematodes related to C. elegans. WormBook, 1-17. |
|  |  | Herman, M.A. Hermaphrodite cell-fate specification. WormBook, 1-16. |
|  |  | Antebi, A. Nuclear hormone receptors in C. elegans. WormBook, 1-13. |
|  |  | Hobert, O. Specification of the nervous system. WormBook, 1-19. |
|  |  | Okkema, P.G. and Krause, M. Transcriptional regulation. WormBook, 1-40. |
|  |  | Sternberg, P.W. Vulval development. WormBook, 1-28. |
|  |  | Eisenmann, D.M. Wnt signaling. WormBook, 1-17. |
|  |  | Wagmaister, J.A., Miley, G.R., Morris, C.A., Gleason, J.E., Miller, L.M., Kornfeld, K. and Eisenmann, D.M. Identification of cis-regulatory elements from the C. elegans Hox gene lin-39 required for embryonic expression and for regulation by the transcription factors LIN-1, LIN-31 and LIN-39. Dev Biol 297, 550-565. |
|  |  | Schindelman, G., Whittaker, A.J., Thum, J.Y., Gharib, S. and Sternberg, P.W. Initiation of male sperm-transfer behavior in Caenorhabditis elegans requires input from the ventral nerve cord. BMC Biol 4, 26. |
|  |  | Takacs-Vellai, K., Vellai, T., Chen, E.B., Zhang, Y., Guerry, F., Stern, M.J. and Muller, F. Transcriptional control of Notch signaling by a HOX and a PBX/EXD protein during vulval development in C. elegans. Dev Biol 302, 661-669. |
|  |  | Schmitz, C., Kinge, P. and Hutter, H. Axon guidance genes identified in a large-scale RNAi screen using the RNAi-hypersensitive Caenorhabditis elegans strain nre-1(hd20) lin-15b(hd126). Proc Natl Acad Sci U S A 104, 834-839. |
|  |  | Yang, Y., Sun, Y., Luo, X., Zhang, Y., Chen, Y., Tian, E., Lints, R. and Zhang, H. Polycomb-like genes are necessary for specification of dopaminergic and serotonergic neurons in Caenorhabditis elegans. Proc Natl Acad Sci U S A 104, 852-857. |
|  |  | Huang, L.S. and Sternberg, P.W. Genetic dissection of developmental pathways. WormBook, 1-19. |
|  |  | Felix, M.A. Oscheius tipulae. WormBook, 1-8. |
|  |  | Guerry, F., Marti, C.O., Zhang, Y., Moroni, P.S., Jaquiery, E. and Muller, F. The Mi-2 nucleosome-remodeling protein LET-418 is targeted via LIN-1/ETS to the promoter of lin-39/Hox during vulval development in C. elegans. Dev Biol 306, 469-479. |
|  |  | Meyer, J.N., Boyd, W.A., Azzam, G.A., Haugen, A.C., Freedman, J.H. and Van Houten, B. Decline of nucleotide excision repair capacity in aging Caenorhabditis elegans. Genome Biol 8, R70. |
|  |  | Yi, B. and Sommer, R.J. The pax-3 gene is involved in vulva formation in Pristionchus pacificus and is a target of the Hox gene lin-39. Development 134, 3111-3119. |
|  |  | Sun, H., Nelms, B.L., Sleiman, S.F., Chamberlin, H.M. and Hanna-Rose, W. Modulation of Caenorhabditis elegans transcription factor activity by HIM-8 and the related Zinc-Finger ZIM proteins. Genetics 177, 1221-1226. |
|  |  | Jiang, Y., Shi, H., Amin, N.M., Sultan, I. and Liu, J. Mesodermal expression of the C. elegans HMX homolog mls-2 requires the PBC homolog CEH-20. Mech Dev 125, 451-461. |
|  |  | Hardin, J. and King, R.S. The long and the short of Wnt signaling in C. elegans. Curr Opin Genet Dev 18, 362-367. |
|  |  | Zhang, M., Chung, S.H., Fang-Yen, C., Craig, C., Kerr, R.A., Suzuki, H., Samuel, A.D., Mazur, E. and Schafer, W.R. A self-regulating feed-forward circuit controlling C. elegans egg-laying behavior. Curr Biol 18, 1445-1455. |
|  |  | Kuntz, S.G., Schwarz, E.M., DeModena, J.A., De Buysscher, T., Trout, D., Shizuya, H., Sternberg, P.W. and Wold, B.J. Multigenome DNA sequence conservation identifies Hox cis-regulatory elements. Genome Res 18, 1955-1968. |
|  |  | Irazoqui, J.E., Ng, A., Xavier, R.J. and Ausubel, F.M. Role for beta-catenin and HOX transcription factors in Caenorhabditis elegans and mammalian host epithelial-pathogen interactions. Proc Natl Acad Sci U S A 105, 17469-17474. |
|  |  | Van Hoffelen, S. and Herman, M.A. Analysis of Wnt signaling during Caenorhabditis elegans postembryonic development. Methods Mol Biol 469, 87-102. |
|  |  | Potts, M.B., Wang, D.P. and Cameron, S. Trithorax, Hox, and TALE-class homeodomain proteins ensure cell survival through repression of the BH3-only gene egl-1. Dev Biol 329, 374-385. |
|  |  | Szabo, E., Hargitai, B., Regos, A., Tihanyi, B., Barna, J., Borsos, E., Takacs-Vellai, K. and Vellai, T. TRA-1/GLI controls the expression of the Hox gene lin-39 during C. elegans vulval development. Dev Biol 330, 339-348. |
|  |  | Jovelin, R. Rapid sequence evolution of transcription factors controlling neuron differentiation in Caenorhabditis. Mol Biol Evol 26, 2373-2386. |
|  |  | Howell, K., Arur, S., Schedl, T. and Sundaram, M.V. EOR-2 is an obligate binding partner of the BTB-zinc finger protein EOR-1 in Caenorhabditis elegans. Genetics 184, 899-913. |
|  |  | Hueber, S.D., Weiller, G.F., Djordjevic, M.A. and Frickey, T. Improving Hox protein classification across the major model organisms. PLoS One 5, e10820. |
|  |  | Tihanyi, B., Vellai, T., Regos, A., Ari, E., Muller, F. and Takacs-Vellai, K. The C. elegans Hox gene ceh-13 regulates cell migration and fusion in a non-colinear way. Implications for the early evolution of Hox clusters. BMC Dev Biol 10, 78. |
| mab-5 | C08C3.3 | Hodgkin, J. Male Phenotypes and Mating Efficiency in CAENORHABDITIS ELEGANS. Genetics 103, 43-64. |
|  |  | Chalfie, M., Thomson, J.N. and Sulston, J.E. Induction of neuronal branching in Caenorhabditis elegans. Science 221, 61-63. |
|  |  | Kenyon, C. A gene involved in the development of the posterior body region of C. elegans. Cell 46, 477-487. |
|  |  | Hedgecock, E.M., Culotti, J.G., Hall, D.H. and Stern, B.D. Genetics of cell and axon migrations in Caenorhabditis elegans. Development 100, 365-382. |
|  |  | Hodgkin, J. Sex determination and dosage compensation in Caenorhabditis elegans. Annu Rev Genet 21, 133-154. |
|  |  | Walthall, W.W. and Chalfie, M. Cell-cell interactions in the guidance of late-developing neurons in Caenorhabditis elegans. Science 239, 643-645. |
|  |  | Kenyon, C. The nematode Caenorhabditis elegans. Science 240, 1448-1453. |
|  |  | Link, C.D., Ehrenfels, C.W. and Wood, W.B. Mutant expression of male copulatory bursa surface markers in Caenorhabditis elegans. Development 103, 485-495. |
|  |  | Costa, M., Weir, M., Coulson, A., Sulston, J. and Kenyon, C. Posterior pattern formation in C. elegans involves position-specific expression of a gene containing a homeobox. Cell 55, 747-756. |
|  |  | Herman, R.K. Mosaic analysis in the nematode Caenorhabditis elegans. J Neurogenet 5, 1-24. |
|  |  | Waring, D.A. and Kenyon, C. Selective silencing of cell communication influences anteroposterior pattern formation in C. elegans. Cell 60, 123-131. |
|  |  | Waring, D.A. and Kenyon, C. Regulation of cellular responsiveness to inductive signals in the developing C. elegans nervous system. Nature 350, 712-715. |
|  |  | Chisholm, A. Control of cell fate in the tail region of C. elegans by the gene egl-5. Development 111, 921-932. |
|  |  | Bürglin, T.R., Ruvkun, G., Coulson, A., Hawkins, N.C., McGhee, J.D., Schaller, D., Wittmann, C., Müller, F. and Waterston, R.H. Nematode homeobox cluster. Nature 351, 703. |
|  |  | Siddiqui, S.S. and Culotti, J.G. Examination of neurons in wild type and mutants of Caenorhabditis elegans using antibodies to horseradish peroxidase. J Neurogenet 21, 271-289. |
|  |  | Schedin, P., Hunter, C.P. and Wood, W.B. Autonomy and nonautonomy of sex determination in triploid intersex mosaics of C. elegans. Development 112, 863-879. |
|  |  | Hodgkin, J. The interactive worm. Eighth international C. elegans Meeting, Madison, WI, USA, June 1-5, 1991. New Biol 3, 951-954. |
|  |  | Salser, S.J. and Kenyon, C. Activation of a C. elegans Antennapedia homologue in migrating cells controls their direction of migration. Nature 355, 255-258. |
|  |  | Emmons, S.W. From cell fates to morphology: developmental genetics of the Caenorhabditis elegans male tail. Bioessays 14, 309-316. |
|  |  | Naito, M., Kohara, Y. and Kurosawa, Y. Identification of a homeobox-containing gene located between lin-45 and unc-24 on chromosome IV in the nematode Caenorhabditis elegans. Nucleic Acids Res 20, 2967-2969. |
|  |  | Cowing, D.W. and Kenyon, C. Expression of the homeotic gene mab-5 during Caenorhabditis elegans embryogenesis. Development 116, 481-490. |
|  |  | Way, J.C., Run, J.Q. and Wang, A.Y. Regulation of anterior cell-specific mec-3 expression during asymmetric cell division in C. elegans. Dev Dyn 194, 289-302. |
|  |  | Katz, W.S. and Sternberg, P.W. A plethora of intercellular signals during Caenorhabditis elegans development. Curr Opin Cell Biol 4, 939-947. |
|  |  | Bargmann, C.I. Death from natural and unnatural causes. Curr Biol 1, 388-390. |
|  |  | Chalfie, M. Homeobox genes in Caenorhabditis elegans. Curr Opin Genet Dev 3, 275-277. |
|  |  | Stringham, E.G. and Candido, E.P. Targeted single-cell induction of gene products in Caenorhabditis elegans: a new tool for developmental studies. J Exp Zool 266, 227-233. |
|  |  | Wang, B.B., Muller-Immergluck, M.M., Austin, J., Robinson, N.T., Chisholm, A. and Kenyon, C. A homeotic gene cluster patterns the anteroposterior body axis of C. elegans. Cell 74, 29-42. |
|  |  | Clark, S.G., Chisholm, A.D. and Horvitz, H.R. Control of cell fates in the central body region of C. elegans by the homeobox gene lin-39. Cell 74, 43-55. |
|  |  | Roehl, H. and Kimble, J. Control of cell fate in C. elegans by a GLP-1 peptide consisting primarily of ankyrin repeats. Nature 364, 632-635. |
|  |  | Salser, S.J., Loer, C.M. and Kenyon, C. Multiple HOM-C gene interactions specify cell fates in the nematode central nervous system. Genes Dev 7, 1714-1724. |
|  |  | Bürglin, T.R. and Ruvkun, G. The Caenorhabditis elegans homeobox gene cluster. Curr Opin Genet Dev 3, 615-620. |
|  |  | Mitani, S., Du, H., Hall, D.H., Driscoll, M. and Chalfie, M. Combinatorial control of touch receptor neuron expression in Caenorhabditis elegans. Development 119, 773-783. |
|  |  | Barbazuk, W.B., Johnsen, R.C. and Baillie, D.L. The generation and genetic analysis of suppressors of lethal mutations in the Caenorhabditis elegans rol-3(V) gene. Genetics 136, 129-143. |
|  |  | Sternberg, P.W. Intercellular signaling and signal transduction in C. elegans. Annu Rev Genet 27, 497-521. |
|  |  | Wood, W.B. and Edgar, L.G. Patterning in the C. elegans embryo. Trends Genet 10, 49-54. |
|  |  | Wilson, R., Ainscough, R., Anderson, K., Baynes, C., Berks, M., Bonfield, J., Burton, J., Connell, M., Copsey, T., Cooper, J. et al. 2.2 Mb of contiguous nucleotide sequence from chromosome III of C. elegans. Nature 368, 32-38. |
|  |  | Austin, J. and Kenyon, C. Cell contact regulates neuroblast formation in the Caenorhabditis elegans lateral epidermis. Development 120, 313-323. |
|  |  | Salser, S.J. and Kenyon, C. Patterning C. elegans: homeotic cluster genes, cell fates and cell migrations. Trends Genet 10, 159-164. |
|  |  | Stroeher, V.L., Kennedy, B.P., Millen, K.J., Schroeder, D.F., Hawkins, M.G., Goszczynski, B. and McGhee, J.D. DNA-protein interactions in the Caenorhabditis elegans embryo: oocyte and embryonic factors that bind to the promoter of the gut-specific ges-1 gene. Dev Biol 163, 367-380. |
|  |  | Zarkower, D., De Bono, M., Aronoff, R. and Hodgkin, J. Regulatory rearrangements and smg-sensitive alleles of the C. elegans sex-determining gene tra-1. Dev Genet 15, 240-250. |
|  |  | Waterston, R., Ainscough, R., Anderson, K., Berks, M., Blair, D., Connell, M., Cooper, J., Coulson, A., Craxton, M., Dear, S. et al. The genome of the nematode Caenorhabditis elegans. Cold Spring Harb Symp Quant Biol 58, 367-376. |
|  |  | Garriga, G. and Stern, M.J. Hams and Egls: genetic analysis of cell migration in Caenorhabditis elegans. Curr Opin Genet Dev 4, 575-580. |
|  |  | Culotti, J.G. Axon guidance mechanisms in Caenorhabditis elegans. Curr Opin Genet Dev 4, 587-595. |
|  |  | Stenico, M., Lloyd, A.T. and Sharp, P.M. Codon usage in Caenorhabditis elegans: delineation of translational selection and mutational biases. Nucleic Acids Res 22, 2437-2446. |
|  |  | Chow, K.L. and Emmons, S.W. HOM-C/Hox genes and four interacting loci determine the morphogenetic properties of single cells in the nematode male tail. Development 120, 2579-2592. |
|  |  | Sommer, R.J., Carta, L.K. and Sternberg, P.W. The evolution of cell lineage in nematodes. Dev Suppl, 85-95. |
|  |  | Duggan, A. and Chalfie, M. Control of neuronal development in Caenorhabditis elegans. Curr Opin Neurobiol 5, 6-9. |
|  |  | Walthall, W.W. Repeating patterns of motoneurons in nematodes: the origin of segmentation? Exs 72, 61-75. |
|  |  | Hunter, C.P. and Kenyon, C. Specification of anteroposterior cell fates in Caenorhabditis elegans by Drosophila Hox proteins. Nature 377, 229-232. |
|  |  | Sengupta, P. and Bargmann, C.I. Cell fate specification and differentiation in the nervous system of Caenorhabditis elegans. Dev Genet 18, 73-80. |
|  |  | Salser, S.J. and Kenyon, C. A C. elegans Hox gene switches on, off, on and off again to regulate proliferation, differentiation and morphogenesis. Development 122, 1651-1661. |
|  |  | Ahringer, J. Posterior patterning by the Caenorhabditis elegans even-skipped homolog vab-7. Genes Dev 10, 1120-1130. |
|  |  | Baumeister, R., Liu, Y. and Ruvkun, G. Lineage-specific regulators couple cell lineage asymmetry to the transcription of the Caenorhabditis elegans POU gene unc-86 during neurogenesis. Genes Dev 10, 1395-1410. |
|  |  | Cowing, D. and Kenyon, C. Correct Hox gene expression established independently of position in Caenorhabditis elegans. Nature 382, 353-356. |
|  |  | Emmons, S.W. Simple worms, complex genes. Nature 382, 301-302. |
|  |  | Harris, J., Honigberg, L., Robinson, N. and Kenyon, C. Neuronal cell migration in C. elegans: regulation of Hox gene expression and cell position. Development 122, 3117-3131. |
|  |  | Clandinin, T.R., Katz, W.S. and Sternberg, P.W. Caenorhabditis elegans HOM-C genes regulate the response of vulval precursor cells to inductive signal. Dev Biol 182, 150-161. |
|  |  | Schnabel, R. and Schnabel, H. Hox genes misled by local environments. Nature 385, 588-589. |
|  |  | Wrischnik, L.A. and Kenyon, C.J. The role of lin-22, a hairy/enhancer of split homolog, in patterning the peripheral nervous system of C. elegans. Development 124, 2875-2888. |
|  |  | Sternberg, P.W. and Felix, M.A. Evolution of cell lineage. Curr Opin Genet Dev 7, 543-550. |
|  |  | Wittmann, C., Bossinger, O., Goldstein, B., Fleischmann, M., Kohler, R., Brunschwig, K., Tobler, H. and Muller, F. The expression of the C. elegans labial-like Hox gene ceh-13 during early embryogenesis relies on cell fate and on anteroposterior cell polarity. Development 124, 4193-4200. |
|  |  | Maloof, J.N. and Kenyon, C. The Hox gene lin-39 is required during C. elegans vulval induction to select the outcome of Ras signaling. Development 125, 181-190. |
|  |  | Kenyon, C.J., Austin, J., Costa, M., Cowing, D.W., Harris, J.M., Honigberg, L., Hunter, C.P., Maloof, J.N., Muller-Immergluck, M.M., Salser, S.J. et al. The dance of the Hox genes: patterning the anteroposterior body axis of Caenorhabditis elegans. Cold Spring Harb Symp Quant Biol 62, 293-305. |
|  |  | Jungblut, B. and Sommer, R.J. The Pristionchus pacificus mab-5 gene is involved in the regulation of ventral epidermal cell fates. Curr Biol 8, 775-778. |
|  |  | Harfe, B.D., Vaz Gomes, A., Kenyon, C., Liu, J., Krause, M. and Fire, A. Analysis of a Caenorhabditis elegans Twist homolog identifies conserved and divergent aspects of mesodermal patterning. Genes Dev 12, 2623-2635. |
|  |  | Eisenmann, D.M., Maloof, J.N., Simske, J.S., Kenyon, C. and Kim, S.K. The beta-catenin homolog BAR-1 and LET-60 Ras coordinately regulate the Hox gene lin-39 during Caenorhabditis elegans vulval development. Development 125, 3667-3680. |
|  |  | Ruvkun, G. and Hobert, O. The taxonomy of developmental control in Caenorhabditis elegans. Science 282, 2033-2041. |
|  |  | Schroeder, D.F. and McGhee, J.D. Anterior-posterior patterning within the Caenorhabditis elegans endoderm. Development 125, 4877-4887. |
|  |  | Maloof, J.N., Whangbo, J., Harris, J.M., Jongeward, G.D. and Kenyon, C. A Wnt signaling pathway controls hox gene expression and neuroblast migration in C. elegans. Development 126, 37-49. |
|  |  | Kagoshima, H., Cassata, G. and Bürglin, T.R. A Caenorhabditis elegans homeobox gene expressed in the male tail, a link between pattern formation and sexual dimorphism? Dev Genes Evol 209, 59-62. |
|  |  | Ferreira, H.B., Zhang, Y., Zhao, C. and Emmons, S.W. Patterning of Caenorhabditis elegans posterior structures by the Abdominal-B homolog, egl-5. Dev Biol 207, 215-228. |
|  |  | Hunter, C.P., Harris, J.M., Maloof, J.N. and Kenyon, C. Hox gene expression in a single Caenorhabditis elegans cell is regulated by a caudal homolog and intercellular signals that inhibit wnt signaling. Development 126, 805-814. |
|  |  | Herman, M.A., Ch'ng, Q., Hettenbach, S.M., Ratliff, T.M., Kenyon, C. and Herman, R.K. EGL-27 is similar to a metastasis-associated factor and controls cell polarity and cell migration in C. elegans. Development 126, 1055-1064. |
|  |  | Brunschwig, K., Wittmann, C., Schnabel, R., Bürglin, T.R., Tobler, H. and Müller, F. Anterior organization of the Caenorhabditis elegans embryo by the labial-like Hox gene ceh-13. Development 126, 1537-1546. |
|  |  | Solari, F., Bateman, A. and Ahringer, J. The Caenorhabditis elegans genes egl-27 and egr-1 are similar to MTA1, a member of a chromatin regulatory complex, and are redundantly required for embryonic patterning. Development 126, 2483-2494. |
|  |  | Sym, M., Robinson, N. and Kenyon, C. MIG-13 positions migrating cells along the anteroposterior body axis of C. elegans. Cell 98, 25-36. |
|  |  | Ch'ng, Q. and Kenyon, C. egl-27 generates anteroposterior patterns of cell fusion in C. elegans by regulating Hox gene expression and Hox protein function. Development 126, 3303-3312. |
|  |  | Branda, C.S. and Stern, M.J. Cell migration and axon growth cone guidance in Caenorhabditis elegans. Curr Opin Genet Dev 9, 479-484. |
|  |  | Chow, K.L. and Chan, K.W. Stress-induced phenocopy of C. elegans defines functional steps of sensory organ differentiation. Dev Growth Differ 41, 629-637. |
|  |  | Yochem, J., Sundaram, M. and Bucher, E.A. Mosaic analysis in Caenorhabditis elegans. Methods Mol Biol 135, 447-462. |
|  |  | Whangbo, J. and Kenyon, C. A Wnt signaling system that specifies two patterns of cell migration in C. elegans. Mol Cell 4, 851-858. |
|  |  | Hsieh, J., Liu, J., Kostas, S.A., Chang, C., Sternberg, P.W. and Fire, A. The RING finger/B-box factor TAM-1 and a retinoblastoma-like protein LIN-35 modulate context-dependent gene silencing in Caenorhabditis elegans. Genes Dev 13, 2958-2970. |
|  |  | Chamberlin, H.M. and Thomas, J.H. The bromodomain protein LIN-49 and trithorax-related protein LIN-59 affect development and gene expression in Caenorhabditis elegans. Development 127, 713-723. |
|  |  | Van Auken, K., Weaver, D.C., Edgar, L.G. and Wood, W.B. Caenorhabditis elegans embryonic axial patterning requires two recently discovered posterior-group Hox genes. Proc Natl Acad Sci U S A 97, 4499-4503. |
|  |  | Shemer, G. and Podbilewicz, B. Fusomorphogenesis: cell fusion in organ formation. Dev Dyn 218, 30-51. |
|  |  | Corsi, A.K., Kostas, S.A., Fire, A. and Krause, M. Caenorhabditis elegans twist plays an essential role in non-striated muscle development. Development 127, 2041-2051. |
|  |  | Korswagen, H.C., Herman, M.A. and Clevers, H.C. Distinct beta-catenins mediate adhesion and signalling functions in C. elegans. Nature 406, 527-532. |
|  |  | Jungblut, B. and Sommer, R.J. Novel cell-cell interactions during vulva development in Pristionchus pacificus. Development 127, 3295-3303. |
|  |  | Grant, K., Hanna-Rose, W. and Han, M. sem-4 promotes vulval cell-fate determination in Caenorhabditis elegans through regulation of lin-39 Hox. Dev Biol 224, 496-506. |
|  |  | Zhang, H. and Emmons, S.W. A C. elegans mediator protein confers regulatory selectivity on lineage-specific expression of a transcription factor gene. Genes Dev 14, 2161-2172. |
|  |  | Hermann, G.J., Leung, B. and Priess, J.R. Left-right asymmetry in C. elegans intestine organogenesis involves a LIN-12/Notch signaling pathway. Development 127, 3429-3440. |
|  |  | Yi, W., Ross, J.M. and Zarkower, D. Mab-3 is a direct tra-1 target gene regulating diverse aspects of C. elegans male sexual development and behavior. Development 127, 4469-4480. |
|  |  | Honigberg, L. and Kenyon, C. Establishment of left/right asymmetry in neuroblast migration by UNC-40/DCC, UNC-73/Trio and DPY-19 proteins in C. elegans. Development 127, 4655-4668. |
|  |  | Adoutte, A. Evolutionary biology. Small but mighty timekeepers. Nature 408, 37-38. |
|  |  | Liu, J. and Fire, A. Overlapping roles of two Hox genes and the exd ortholog ceh-20 in diversification of the C. elegans postembryonic mesoderm. Development 127, 5179-5190. |
|  |  | Portman, D.S. and Emmons, S.W. The basic helix-loop-helix transcription factors LIN-32 and HLH-2 function together in multiple steps of a C. elegans neuronal sublineage. Development 127, 5415-5426. |
|  |  | Herman, M. C. elegans POP-1/TCF functions in a canonical Wnt pathway that controls cell migration and in a noncanonical Wnt pathway that controls cell polarity. Development 128, 581-590. |
|  |  | Zhang, H. and Emmons, S.W. The novel C. elegans gene sop-3 modulates Wnt signaling to regulate Hox gene expression. Development 128, 767-777. |
|  |  | Alper, S. and Kenyon, C. REF-1, a protein with two bHLH domains, alters the pattern of cell fusion in C. elegans by regulating Hox protein activity. Development 128, 1793-1804. |
|  |  | Kwon, J.Y. and Lee, J. Biological significance of a universally conserved transcription mediator in metazoan developmental signaling pathways. Development 128, 3095-3104. |
|  |  | Jungblut, B., Pires-daSilva, A. and Sommer, R.J. Formation of the egg-laying system in Pristionchus pacificus requires complex interactions between gonadal, mesodermal and epidermal tissues and does not rely on single cell inductions. Development 128, 3395-3404. |
|  |  | Lambie, E.J. Cell proliferation and growth in C. elegans. Bioessays 24, 38-53. |
|  |  | Kostas, S.A. and Fire, A. The T-box factor MLS-1 acts as a molecular switch during specification of nonstriated muscle in C. elegans. Genes Dev 16, 257-269. |
|  |  | Cameron, S., Clark, S.G., McDermott, J.B., Aamodt, E. and Horvitz, H.R. PAG-3, a Zn-finger transcription factor, determines neuroblast fate in C. elegans. Development 129, 1763-1774. |
|  |  | Korswagen, H.C., Coudreuse, D.Y., Betist, M.C., van de Water, S., Zivkovic, D. and Clevers, H.C. The Axin-like protein PRY-1 is a negative regulator of a canonical Wnt pathway in C. elegans. Genes Dev 16, 1291-1302. |
|  |  | Alper, S. and Kenyon, C. The zinc finger protein REF-2 functions with the Hox genes to inhibit cell fusion in the ventral epidermis of C. elegans. Development 129, 3335-3348. |
|  |  | Hobert, O., Johnston, R.J., Jr. and Chang, S. Left-right asymmetry in the nervous system: the Caenorhabditis elegans model. Nat Rev Neurosci 3, 629-640. |
|  |  | Korswagen, H.C. Canonical and non-canonical Wnt signaling pathways in Caenorhabditis elegans: variations on a common signaling theme. Bioessays 24, 801-810. |
|  |  | Herman, M.A. Control of cell polarity by noncanonical Wnt signaling in C. elegans. Semin Cell Dev Biol 13, 233-241. |
|  |  | Aboobaker, A.A. and Blaxter, M.L. Hox Gene Loss during Dynamic Evolution of the Nematode Cluster. Curr Biol 13, 37-40. |
|  |  | Gutierrez, A., Knoch, L., Witte, H. and Sommer, R.J. Functional specificity of the nematode Hox gene mab-5. Development 130, 983-993. |
|  |  | Hahn, A.C. and Emmons, S.W. The roles of an ephrin and a semaphorin in patterning cell-cell contacts in C. elegans sensory organ development. Dev Biol 256, 379-388. |
|  |  | Pires-daSilva, A. and Sommer, R.J. Finally, worm polycomb-like genes meet Hox regulation. Dev Cell 4, 770-772. |
|  |  | Ross, J.M. and Zarkower, D. Polycomb group regulation of Hox gene expression in C. elegans. Dev Cell 4, 891-901. |
|  |  | Zhang, H., Azevedo, R.B., Lints, R., Doyle, C., Teng, Y., Haber, D. and Emmons, S.W. Global regulation of Hox gene expression in C. elegans by a SAM domain protein. Dev Cell 4, 903-915. |
|  |  | Venkatesan, K., McManus, H.R., Mello, C.C., Smith, T.F. and Hansen, U. Functional conservation between members of an ancient duplicated transcription factor family, LSF/Grainyhead. Nucleic Acids Res 31, 4304-4316. |
|  |  | Chang, S., Johnston, R.J., Jr. and Hobert, O. A transcriptional regulatory cascade that controls left/right asymmetry in chemosensory neurons of C. elegans. Genes Dev 17, 2123-2137. |
|  |  | Toker, A.S., Teng, Y., Ferreira, H.B., Emmons, S.W. and Chalfie, M. The Caenorhabditis elegans spalt-like gene sem-4 restricts touch cell fate by repressing the selector Hox gene egl-5 and the effector gene mec-3. Development 130, 3831-3840. |
|  |  | Ch'ng, Q., Williams, L., Lie, Y.S., Sym, M., Whangbo, J. and Kenyon, C. Identification of genes that regulate a left-right asymmetric neuronal migration in Caenorhabditis elegans. Genetics 164, 1355-1367. |
|  |  | Aboobaker, A. and Blaxter, M. Hox gene evolution in nematodes: novelty conserved. Curr Opin Genet Dev 13, 593-598. |
|  |  | Herman, M.A. and Wu, M. Noncanonical Wnt signaling pathways in C. elegans converge on POP-1/TCF and control cell polarity. Front Biosci 9, 1530-1539. |
|  |  | Lints, R., Jia, L., Kim, K., Li, C. and Emmons, S.W. Axial patterning of C. elegans male sensilla identities by selector genes. Dev Biol 269, 137-151. |
|  |  | Botas, J. Control of morphogenesis and differentiation by HOM/Hox genes. Curr Opin Cell Biol 5, 1015-1022. |
|  |  | Nicholas, H.R. and Hodgkin, J. The ERK MAP kinase cascade mediates tail swelling and a protective response to rectal infection in C. elegans. Curr Biol 14, 1256-1261. |
|  |  | Silhankova, M., Jindra, M. and Asahina, M. Nuclear receptor NHR-25 is required for cell-shape dynamics during epidermal differentiation in Caenorhabditis elegans. J Cell Sci 118, 223-232. |
|  |  | Forrester, W.C., Kim, C. and Garriga, G. The Caenorhabditis elegans Ror RTK CAM-1 inhibits EGL-20/Wnt signaling in cell migration. Genetics 168, 1951-1962. |
|  |  | Ross, J.M., Kalis, A.K., Murphy, M.W. and Zarkower, D. The DM domain protein MAB-3 promotes sex-specific neurogenesis in C. elegans by regulating bHLH proteins. Dev Cell 8, 881-892. |
|  |  | Felix, M.A. An inversion in the wiring of an intercellular signal: evolution of Wnt signaling in the nematode vulva. Bioessays 27, 765-769. |
|  |  | Gilleard, J.S. The use of Caenorhabditis elegans in parasitic nematode research. Parasitology 128 Suppl 1, S49-70. |
|  |  | Jiang, Y., Horner, V. and Liu, J. The HMX homeodomain protein MLS-2 regulates cleavage orientation, cell proliferation and cell fate specification in the C. elegans postembryonic mesoderm. Development 132, 4119-4130. |
|  |  | Tiensuu, T., Larsen, M.K., Vernersson, E. and Tuck, S. lin-1 has both positive and negative functions in specifying multiple cell fates induced by Ras/MAP kinase signaling in C. elegans. Dev Biol 286, 338-351. |
|  |  | Shakir, M.A., Gill, J.S. and Lundquist, E.A. Interactions of UNC-34 Enabled with Rac GTPases and the NIK kinase MIG-15 in Caenorhabditis elegans axon pathfinding and neuronal migration. Genetics 172, 893-913. |
|  |  | Liu, H., Strauss, T.J., Potts, M.B. and Cameron, S. Direct regulation of egl-1 and of programmed cell death by the Hox protein MAB-5 and by CEH-20, a C. elegans homolog of Pbx1. Development 133, 641-650. |
|  |  | Dixon, S.J., Alexander, M., Fernandes, R., Ricker, N. and Roy, P.J. FGF negatively regulates muscle membrane extension in Caenorhabditis elegans. Development 133, 1263-1275. |
|  |  | Zhang, T., Sun, Y., Tian, E., Deng, H., Zhang, Y., Luo, X., Cai, Q., Wang, H., Chai, J. and Zhang, H. RNA-binding proteins SOP-2 and SOR-1 form a novel PcG-like complex in C. elegans. Development 133, 1023-1033. |
|  |  | Podbilewicz, B. Cell fusion. WormBook, 1-32. |
|  |  | Herman, M.A. Hermaphrodite cell-fate specification. WormBook, 1-16. |
|  |  | Emmons, S.W. Male development. WormBook, 1-22. |
|  |  | Hobert, O. Specification of the nervous system. WormBook, 1-19. |
|  |  | Okkema, P.G. and Krause, M. Transcriptional regulation. WormBook, 1-40. |
|  |  | Sternberg, P.W. Vulval development. WormBook, 1-28. |
|  |  | Eisenmann, D.M. Wnt signaling. WormBook, 1-17. |
|  |  | Coudreuse, D.Y., Roel, G., Betist, M.C., Destree, O. and Korswagen, H.C. Wnt gradient formation requires retromer function in Wnt-producing cells. Science 312, 921-924. |
|  |  | Schlager, B., Roseler, W., Zheng, M., Gutierrez, A. and Sommer, R.J. HAIRY-like transcription factors and the evolution of the nematode vulva equivalence group. Curr Biol 16, 1386-1394. |
|  |  | Takacs-Vellai, K., Vellai, T., Chen, E.B., Zhang, Y., Guerry, F., Stern, M.J. and Muller, F. Transcriptional control of Notch signaling by a HOX and a PBX/EXD protein during vulval development in C. elegans. Dev Biol 302, 661-669. |
|  |  | Amin, N.M., Hu, K., Pruyne, D., Terzic, D., Bretscher, A. and Liu, J. A Zn-finger/FH2-domain containing protein, FOZI-1, acts redundantly with CeMyoD to specify striated body wall muscle fates in the Caenorhabditis elegans postembryonic mesoderm. Development 134, 19-29. |
|  |  | Oosterveen, T., Coudreuse, D.Y., Yang, P.T., Fraser, E., Bergsma, J., Dale, T.C. and Korswagen, H.C. Two functionally distinct Axin-like proteins regulate canonical Wnt signaling in C. elegans. Dev Biol 308, 438-448. |
|  |  | Pan, C.L., Baum, P.D., Gu, M., Jorgensen, E.M., Clark, S.G. and Garriga, G. C. elegans AP-2 and retromer control Wnt signaling by regulating mig-14/Wntless. Dev Cell 14, 132-139. |
|  |  | Cai, Q., Sun, Y., Huang, X., Guo, C., Zhang, Y., Zhu, Z. and Zhang, H. The Caenorhabditis elegans PcG-like gene sop-2 regulates the temporal and sexual specificities of cell fates. Genetics 178, 1445-1456. |
|  |  | Jiang, Y., Shi, H., Amin, N.M., Sultan, I. and Liu, J. Mesodermal expression of the C. elegans HMX homolog mls-2 requires the PBC homolog CEH-20. Mech Dev 125, 451-461. |
|  |  | Schmitz, C., Wacker, I. and Hutter, H. The Fat-like cadherin CDH-4 controls axon fasciculation, cell migration and hypodermis and pharynx development in Caenorhabditis elegans. Dev Biol 316, 249-259. |
|  |  | Zinovyeva, A.Y., Yamamoto, Y., Sawa, H. and Forrester, W.C. Complex network of Wnt signaling regulates neuronal migrations during Caenorhabditis elegans development. Genetics 179, 1357-1371. |
|  |  | Shan, G. and Walthall, W.W. Copulation in C. elegans males requires a nuclear hormone receptor. Dev Biol 322, 11-20. |
|  |  | Irazoqui, J.E., Ng, A., Xavier, R.J. and Ausubel, F.M. Role for beta-catenin and HOX transcription factors in Caenorhabditis elegans and mammalian host epithelial-pathogen interactions. Proc Natl Acad Sci U S A 105, 17469-17474. |
|  |  | Van Hoffelen, S. and Herman, M.A. Analysis of Wnt signaling during Caenorhabditis elegans postembryonic development. Methods Mol Biol 469, 87-102. |
|  |  | Amin, N.M., Lim, S.E., Shi, H., Chan, T.L. and Liu, J. A conserved Six-Eya cassette acts downstream of Wnt signaling to direct non-myogenic versus myogenic fates in the C. elegans postembryonic mesoderm. Dev Biol 331, 350-360. |
|  |  | Li, X., Kulkarni, R.P., Hill, R.J. and Chamberlin, H.M. HOM-C genes, Wnt signaling and axial patterning in the C. elegans posterior ventral epidermis. Dev Biol 332, 156-165. |
|  |  | Jovelin, R. Rapid sequence evolution of transcription factors controlling neuron differentiation in Caenorhabditis. Mol Biol Evol 26, 2373-2386. |
|  |  | Jiang, Y., Shi, H. and Liu, J. Two Hox cofactors, the Meis/Hth homolog UNC-62 and the Pbx/Exd homolog CEH-20, function together during C. elegans postembryonic mesodermal development. Dev Biol 334, 535-546. |
|  |  | Yu, H., Seah, A. and Sternberg, P.W. Re-programming of C. elegans male epidermal precursor fates by Wnt, Hox, and LIN-12/Notch activities. Dev Biol 345, 1-11. |
|  |  | Hueber, S.D., Weiller, G.F., Djordjevic, M.A. and Frickey, T. Improving Hox protein classification across the major model organisms. PLoS One 5, e10820. |
|  |  | Tihanyi, B., Vellai, T., Regos, A., Ari, E., Muller, F. and Takacs-Vellai, K. The C. elegans Hox gene ceh-13 regulates cell migration and fusion in a non-colinear way. Implications for the early evolution of Hox clusters. BMC Dev Biol 10, 78. |
| mec-3 | F01D4.6 | Chalfie, M. and Sulston, J. Developmental genetics of the mechanosensory neurons of Caenorhabditis elegans. Dev Biol 82, 358-370. |
|  |  | Hodgkin, J. Male Phenotypes and Mating Efficiency in CAENORHABDITIS ELEGANS. Genetics 103, 43-64. |
|  |  | Trent, C., Tsuing, N. and Horvitz, H.R. Egg-laying defective mutants of the nematode Caenorhabditis elegans. Genetics 104, 619-647. |
|  |  | Kenyon, C. The nematode Caenorhabditis elegans. Science 240, 1448-1453. |
|  |  | Way, J.C. and Chalfie, M. mec-3, a homeobox-containing gene that specifies differentiation of the touch receptor neurons in C. elegans. Cell 54, 5-16. |
|  |  | Finney, M., Ruvkun, G. and Horvitz, H.R. The C. elegans cell lineage and differentiation gene unc-86 encodes a protein with a homeodomain and extended similarity to transcription factors. Cell 55, 757-769. |
|  |  | Chalfie, M. and Au, M. Genetic control of differentiation of the Caenorhabditis elegans touch receptor neurons. Science 243, 1027-1033. |
|  |  | Way, J.C. and Chalfie, M. The mec-3 gene of Caenorhabditis elegans requires its own product for maintained expression and is expressed in three neuronal cell types. Genes Dev 3, 1823-1833. |
|  |  | Freyd, G., Kim, S.K. and Horvitz, H.R. Novel cysteine-rich motif and homeodomain in the product of the Caenorhabditis elegans cell lineage gene lin-11. Nature 344, 876-879. |
|  |  | Sternberg, P.W. Genetic control of cell type and pattern formation in Caenorhabditis elegans. Adv Genet 27, 63-116. |
|  |  | Wolinsky, E. and Way, J. The behavioral genetics of Caenorhabditis elegans. Behav Genet 20, 169-189. |
|  |  | Li, P.M., Reichert, J., Freyd, G., Horvitz, H.R. and Walsh, C.T. The LIM region of a presumptive Caenorhabditis elegans transcription factor is an iron-sulfur- and zinc-containing metallodomain. Proc Natl Acad Sci U S A 88, 9210-9213. |
|  |  | Hodgkin, J. The interactive worm. Eighth international C. elegans Meeting, Madison, WI, USA, June 1-5, 1991. New Biol 3, 951-954. |
|  |  | Way, J.C., Wang, L., Run, J.Q. and Wang, A. The mec-3 gene contains cis-acting elements mediating positive and negative regulation in cells produced by asymmetric cell division in Caenorhabditis elegans. Genes Dev 5, 2199-2211. |
|  |  | White, J.G., Southgate, E. and Thomson, J.N. Mutations in the Caenorhabditis elegans unc-4 gene alter the synaptic input to ventral cord motor neurons. Nature 355, 838-841. |
|  |  | Wadsworth, W.G. and Hedgecock, E.M. Guidance of neuroblast migrations and axonal projections in Caenorhabditis elegans. Curr Opin Neurobiol 2, 36-41. |
|  |  | Clark, D.V. and Baillie, D.L. Genetic analysis and complementation by germ-line transformation of lethal mutations in the unc-22 IV region of Caenorhabditis elegans. Mol Gen Genet 232, 97-105. |
|  |  | Naito, M., Kohara, Y. and Kurosawa, Y. Identification of a homeobox-containing gene located between lin-45 and unc-24 on chromosome IV in the nematode Caenorhabditis elegans. Nucleic Acids Res 20, 2967-2969. |
|  |  | Hamelin, M., Scott, I.M., Way, J.C. and Culotti, J.G. The mec-7 beta-tubulin gene of Caenorhabditis elegans is expressed primarily in the touch receptor neurons. Embo J 11, 2885-2893. |
|  |  | Xue, D., Finney, M., Ruvkun, G. and Chalfie, M. Regulation of the mec-3 gene by the C.elegans homeoproteins UNC-86 and MEC-3. Embo J 11, 4969-4979. |
|  |  | Way, J.C., Run, J.Q. and Wang, A.Y. Regulation of anterior cell-specific mec-3 expression during asymmetric cell division in C. elegans. Dev Dyn 194, 289-302. |
|  |  | Kennedy, B.P., Aamodt, E.J., Allen, F.L., Chung, M.A., Heschl, M.F. and McGhee, J.D. The gut esterase gene (ges-1) from the nematodes Caenorhabditis elegans and Caenorhabditis briggsae. J Mol Biol 229, 890-908. |
|  |  | Bargmann, C.I. Genetic and cellular analysis of behavior in C. elegans. Annu Rev Neurosci 16, 47-71. |
|  |  | Chalfie, M. Homeobox genes in Caenorhabditis elegans. Curr Opin Genet Dev 3, 275-277. |
|  |  | Shackleford, G.M., Shivakumar, S., Shiue, L., Mason, J., Kenyon, C. and Varmus, H.E. Two wnt genes in Caenorhabditis elegans. Oncogene 8, 1857-1864. |
|  |  | Stringham, E.G. and Candido, E.P. Targeted single-cell induction of gene products in Caenorhabditis elegans: a new tool for developmental studies. J Exp Zool 266, 227-233. |
|  |  | Xue, D., Tu, Y. and Chalfie, M. Cooperative interactions between the Caenorhabditis elegans homeoproteins UNC-86 and MEC-3. Science 261, 1324-1328. |
|  |  | Chalfie, M. Touch receptor development and function in Caenorhabditis elegans. J Neurobiol 24, 1433-1441. |
|  |  | Bürglin, T.R. and Ruvkun, G. The Caenorhabditis elegans homeobox gene cluster. Curr Opin Genet Dev 3, 615-620. |
|  |  | Mitani, S., Du, H., Hall, D.H., Driscoll, M. and Chalfie, M. Combinatorial control of touch receptor neuron expression in Caenorhabditis elegans. Development 119, 773-783. |
|  |  | Huang, M. and Chalfie, M. Gene interactions affecting mechanosensory transduction in Caenorhabditis elegans. Nature 367, 467-470. |
|  |  | Chalfie, M., Tu, Y., Euskirchen, G., Ward, W.W. and Prasher, D.C. Green fluorescent protein as a marker for gene expression. Science 263, 802-805. |
|  |  | Culotti, J.G. Axon guidance mechanisms in Caenorhabditis elegans. Curr Opin Genet Dev 4, 587-595. |
|  |  | Stenico, M., Lloyd, A.T. and Sharp, P.M. Codon usage in Caenorhabditis elegans: delineation of translational selection and mutational biases. Nucleic Acids Res 22, 2437-2446. |
|  |  | Way, J.C., Wang, L., Run, J.Q. and Hung, M.S. Cell polarity and the mechanism of asymmetric cell division. Bioessays 16, 925-931. |
|  |  | Hodgkin, J. Epigenetics and the maintenance of gene activity states in Caenorhabditis elegans. Dev Genet 15, 471-477. |
|  |  | Duggan, A. and Chalfie, M. Control of neuronal development in Caenorhabditis elegans. Curr Opin Neurobiol 5, 6-9. |
|  |  | Varkey, J.P., Muhlrad, P.J., Minniti, A.N., Do, B. and Ward, S. The Caenorhabditis elegans spe-26 gene is necessary to form spermatids and encodes a protein similar to the actin-associated proteins kelch and scruin. Genes Dev 9, 1074-1086. |
|  |  | Lichtsteiner, S. and Tjian, R. Synergistic activation of transcription by UNC-86 and MEC-3 in Caenorhabditis elegans embryo extracts. Embo J 14, 3937-3945. |
|  |  | Huang, M., Gu, G., Ferguson, E.L. and Chalfie, M. A stomatin-like protein necessary for mechanosensation in C. elegans. Nature 378, 292-295. |
|  |  | Jia, Y., Xie, G. and Aamodt, E. pag-3, a Caenorhabditis elegans gene involved in touch neuron gene expression and coordinated movement. Genetics 142, 141-147. |
|  |  | Chalfie, M. The differentiation and function of the touch receptor neurons of Caenorhabditis elegans. Prog Brain Res 105, 179-182. |
|  |  | Du, H., Gu, G., William, C.M. and Chalfie, M. Extracellular proteins needed for C. elegans mechanosensation. Neuron 16, 183-194. |
|  |  | Herman, R.K. Touch sensation in Caenorhabditis elegans. Bioessays 18, 199-206. |
|  |  | Run, J.Q., Steven, R., Hung, M.S., van Weeghel, R., Culotti, J.G. and Way, J.C. Suppressors of the unc-73 gene of Caenorhabditis elegans. Genetics 143, 225-236. |
|  |  | Wang, L. and Way, J.C. Activation of the mec-3 promoter in two classes of stereotyped lineages in Caenorhabditis elegans. Mech Dev 56, 165-181. |
|  |  | Wang, L. and Way, J.C. Promoter sequences for the establishment of mec-3 expression in the nematode Caenorhabditis elegans. Mech Dev 56, 183-196. |
|  |  | Basson, M. and Horvitz, H.R. The Caenorhabditis elegans gene sem-4 controls neuronal and mesodermal cell development and encodes a zinc finger protein. Genes Dev 10, 1953-1965. |
|  |  | Garcia-Anoveros, J. and Corey, D.P. The molecules of mechanosensation. Annu Rev Neurosci 20, 567-594. |
|  |  | Tavernarakis, N. and Driscoll, M. Molecular modeling of mechanotransduction in the nematode Caenorhabditis elegans. Annu Rev Physiol 59, 659-689. |
|  |  | Sze, J.Y., Liu, Y. and Ruvkun, G. VP16-activation of the C. elegans neural specification transcription factor UNC-86 suppresses mutations in downstream genes and causes defects in neural migration and axon outgrowth. Development 124, 1159-1168. |
|  |  | Jia, Y., Xie, G., McDermott, J.B. and Aamodt, E. The C. elegans gene pag-3 is homologous to the zinc finger proto-oncogene gfi-1. Development 124, 2063-2073. |
|  |  | Wolf, F.W., Hung, M.S., Wightman, B., Way, J. and Garriga, G. vab-8 is a key regulator of posteriorly directed migrations in C. elegans and encodes a novel protein with kinesin motor similarity. Neuron 20, 655-666. |
|  |  | Rongo, C., Whitfield, C.W., Rodal, A., Kim, S.K. and Kaplan, J.M. LIN-10 is a shared component of the polarized protein localization pathways in neurons and epithelia. Cell 94, 751-759. |
|  |  | Duggan, A., Ma, C. and Chalfie, M. Regulation of touch receptor differentiation by the Caenorhabditis elegans mec-3 and unc-86 genes. Development 125, 4107-4119. |
|  |  | Chalfie, M. and Jorgensen, E.M. C. elegans neuroscience: genetics to genome. Trends Genet 14, 506-512. |
|  |  | Hobert, O., Tessmar, K. and Ruvkun, G. The Caenorhabditis elegans lim-6 LIM homeobox gene regulates neurite outgrowth and function of particular GABAergic neurons. Development 126, 1547-1562. |
|  |  | Sagasti, A., Hobert, O., Troemel, E.R., Ruvkun, G. and Bargmann, C.I. Alternative olfactory neuron fates are specified by the LIM homeobox gene lim-4. Genes Dev 13, 1794-1806. |
|  |  | Kim, S., Ren, X.C., Fox, E. and Wadsworth, W.G. SDQR migrations in Caenorhabditis elegans are controlled by multiple guidance cues and changing responses to netrin UNC-6. Development 126, 3881-3890. |
|  |  | Rockelein, I., Rohrig, S., Donhauser, R., Eimer, S. and Baumeister, R. Identification of amino acid residues in the Caenorhabditis elegans POU protein UNC-86 that mediate UNC-86-MEC-3-DNA ternary complex formation. Mol Cell Biol 20, 4806-4813. |
|  |  | Rohrig, S., Rockelein, I., Donhauser, R. and Baumeister, R. Protein interaction surface of the POU transcription factor UNC-86 selectively used in touch neurons. Embo J 19, 3694-3703. |
|  |  | Cassata, G., Röhrig, S., Kuhn, F., Hauri, H.P., Baumeister, R. and Bürglin, T.R. The Caenorhabditis elegans Ldb/NLI/Clim orthologue ldb-1 is required for neuronal function. Dev Biol 226, 45-56. |
|  |  | Zhang, Y., Ma, C., Delohery, T., Nasipak, B., Foat, B.C., Bounoutas, A., Bussemaker, H.J., Kim, S.K. and Chalfie, M. Identification of genes expressed in C. elegans touch receptor neurons. Nature 418, 331-335. |
|  |  | Toms, N., Cooper, J., Patchen, B. and Aamodt, E. High copy arrays containing a sequence upstream of mec-3 alter cell migration and axonal morphology in C. elegans. BMC Dev Biol 1, 2. |
|  |  | de Bono, M., Tobin, D.M., Davis, M.W., Avery, L. and Bargmann, C.I. Social feeding in Caenorhabditis elegans is induced by neurons that detect aversive stimuli. Nature 419, 899-903. |
|  |  | Zhang, Y. and Chalfie, M. MTD-1, a touch-cell-specific membrane protein with a subtle effect on touch sensitivity. Mech Dev 119, 3-7. |
|  |  | Reinke, V. Functional exploration of the C. elegans genome using DNA microarrays. Nat Genet 32 Suppl, 541-546. |
|  |  | Grant, B.D. and Wilkinson, H.A. Functional genomic maps in Caenorhabditis elegans. Curr Opin Cell Biol 15, 206-212. |
|  |  | Goodman, M.B. and Schwarz, E.M. Transducing touch in Caenorhabditis elegans. Annu Rev Physiol 65, 429-452. |
|  |  | Zhao, B., Khare, P., Feldman, L. and Dent, J.A. Reversal frequency in Caenorhabditis elegans represents an integrated response to the state of the animal and its environment. J Neurosci 23, 5319-5328. |
|  |  | Cutter, A.D. and Payseur, B.A. Rates of deleterious mutation and the evolution of sex in Caenorhabditis. J Evol Biol 16, 812-822. |
|  |  | Toker, A.S., Teng, Y., Ferreira, H.B., Emmons, S.W. and Chalfie, M. The Caenorhabditis elegans spalt-like gene sem-4 restricts touch cell fate by repressing the selector Hox gene egl-5 and the effector gene mec-3. Development 130, 3831-3840. |
|  |  | Clark, S.G. and Chiu, C. C. elegans ZAG-1, a Zn-finger-homeodomain protein, regulates axonal development and neuronal differentiation. Development 130, 3781-3794. |
|  |  | Hwang, S.B. and Lee, J. Neuron cell type-specific SNAP-25 expression driven by multiple regulatory elements in the nematode Caenorhabditis elegans. J Mol Biol 333, 237-247. |
|  |  | Tsalik, E.L., Niacaris, T., Wenick, A.S., Pau, K., Avery, L. and Hobert, O. LIM homeobox gene-dependent expression of biogenic amine receptors in restricted regions of the C. elegans nervous system. Dev Biol 263, 81-102. |
|  |  | Segalat, L. and Neri, C. [C. elegans as a model for human inherited degenerative diseases]. Med Sci (Paris) 19, 1218-1225. |
|  |  | Kim, K. and Li, C. Expression and regulation of an FMRFamide-related neuropeptide gene family in Caenorhabditis elegans. J Comp Neurol 475, 540-550. |
|  |  | Shtonda, B.B. and Avery, L. Dietary choice behavior in Caenorhabditis elegans. J Exp Biol 209, 89-102. |
|  |  | Li, W., Feng, Z., Sternberg, P.W. and Xu, X.Z. A C. elegans stretch receptor neuron revealed by a mechanosensitive TRP channel homologue. Nature 440, 684-687. |
|  |  | Goodman, M.B. Mechanosensation. WormBook, 1-14. |
|  |  | Hobert, O. Specification of the nervous system. WormBook, 1-19. |
|  |  | Okkema, P.G. and Krause, M. Transcriptional regulation. WormBook, 1-40. |
|  |  | Van Buskirk, C. and Sternberg, P.W. Epidermal growth factor signaling induces behavioral quiescence in Caenorhabditis elegans. Nat Neurosci 10, 1300-1307. |
|  |  | Donohoe, D.R., Weeks, K., Aamodt, E.J. and Dwyer, D.S. Antipsychotic drugs alter neuronal development including ALM neuroblast migration and PLM axonal outgrowth in Caenorhabditis elegans. Int J Dev Neurosci 26, 371-380. |
|  |  | Ghosh, R. and Emmons, S.W. Episodic swimming behavior in the nematode C. elegans. J Exp Biol 211, 3703-3711. |
|  |  | Jovelin, R. Rapid sequence evolution of transcription factors controlling neuron differentiation in Caenorhabditis. Mol Biol Evol 26, 2373-2386. |
|  |  | Smith, C.J., Watson, J.D., Spencer, W.C., O'Brien, T., Cha, B., Albeg, A., Treinin, M. and Miller, D.M., 3rd Time-lapse imaging and cell-specific expression profiling reveal dynamic branching and molecular determinants of a multi-dendritic nociceptor in C. elegans. Dev Biol 345, 18-33. |
| mls-2 | C39E6.4 | Jiang, Y., Horner, V. and Liu, J. The HMX homeodomain protein MLS-2 regulates cleavage orientation, cell proliferation and cell fate specification in the C. elegans postembryonic mesoderm. Development 132, 4119-4130. |
|  |  | Okkema, P.G. and Krause, M. Transcriptional regulation. WormBook, 1-40. |
|  |  | Amin, N.M., Hu, K., Pruyne, D., Terzic, D., Bretscher, A. and Liu, J. A Zn-finger/FH2-domain containing protein, FOZI-1, acts redundantly with CeMyoD to specify striated body wall muscle fates in the Caenorhabditis elegans postembryonic mesoderm. Development 134, 19-29. |
|  |  | Jiang, Y., Shi, H., Amin, N.M., Sultan, I. and Liu, J. Mesodermal expression of the C. elegans HMX homolog mls-2 requires the PBC homolog CEH-20. Mech Dev 125, 451-461. |
|  |  | Yoshimura, S., Murray, J.I., Lu, Y., Waterston, R.H. and Shaham, S. mls-2 and vab-3 Control glia development, hlh-17/Olig expression and glia-dependent neurite extension in C. elegans. Development 135, 2263-2275. |
|  |  | Kim, K., Kim, R. and Sengupta, P. The HMX/NKX homeodomain protein MLS-2 specifies the identity of the AWC sensory neuron type via regulation of the ceh-36 Otx gene in C. elegans. Development 137, 963-974. |
| nob-1 | Y75B8A.2 | Fukushige, T., Hawkins, M.G. and McGhee, J.D. The GATA-factor elt-2 is essential for formation of the Caenorhabditis elegans intestine. Dev Biol 198, 286-302. |
|  |  | Van Auken, K., Weaver, D.C., Edgar, L.G. and Wood, W.B. Caenorhabditis elegans embryonic axial patterning requires two recently discovered posterior-group Hox genes. Proc Natl Acad Sci U S A 97, 4499-4503. |
|  |  | Van Auken, K., Weaver, D., Robertson, B., Sundaram, M., Saldi, T., Edgar, L., Elling, U., Lee, M., Boese, Q. and Wood, W.B. Roles of the Homothorax/Meis/Prep homolog UNC-62 and the Exd/Pbx homologs CEH-20 and CEH-40 in C. elegans embryogenesis. Development 129, 5255-5268. |
|  |  | Aboobaker, A.A. and Blaxter, M.L. Hox Gene Loss during Dynamic Evolution of the Nematode Cluster. Curr Biol 13, 37-40. |
|  |  | Aboobaker, A. and Blaxter, M. Hox gene evolution in nematodes: novelty conserved. Curr Opin Genet Dev 13, 593-598. |
|  |  | Landmann, F., Quintin, S. and Labouesse, M. Multiple regulatory elements with spatially and temporally distinct activities control the expression of the epithelial differentiation gene lin-26 in C. elegans. Dev Biol 265, 478-490. |
|  |  | Chen, Z., Eastburn, D.J. and Han, M. The Caenorhabditis elegans nuclear receptor gene nhr-25 regulates epidermal cell development. Mol Cell Biol 24, 7345-7358. |
|  |  | Baugh, L.R., Hill, A.A., Claggett, J.M., Hill-Harfe, K., Wen, J.C., Slonim, D.K., Brown, E.L. and Hunter, C.P. The homeodomain protein PAL-1 specifies a lineage-specific regulatory network in the C. elegans embryo. Development 132, 1843-1854. |
|  |  | Yanagi, K., Mizuno, T., Tsuyama, T., Tada, S., Iida, Y., Sugimoto, A., Eki, T., Enomoto, T. and Hanaoka, F. Caenorhabditis elegans geminin homologue participates in cell cycle regulation and germ line development. J Biol Chem 280, 19689-19694. |
|  |  | Baugh, L.R., Wen, J.C., Hill, A.A., Slonim, D.K., Brown, E.L. and Hunter, C.P. Synthetic lethal analysis of Caenorhabditis elegans posterior embryonic patterning genes identifies conserved genetic interactions. Genome Biol 6, R45. |
|  |  | Herman, M.A. Hermaphrodite cell-fate specification. WormBook, 1-16. |
|  |  | Antebi, A. Nuclear hormone receptors in C. elegans. WormBook, 1-13. |
|  |  | Okkema, P.G. and Krause, M. Transcriptional regulation. WormBook, 1-40. |
|  |  | Arata, Y., Kouike, H., Zhang, Y., Herman, M.A., Okano, H. and Sawa, H. Wnt signaling and a Hox protein cooperatively regulate psa-3/Meis to determine daughter cell fate after asymmetric cell division in C. elegans. Dev Cell 11, 105-115. |
|  |  | Meyer, J.N., Boyd, W.A., Azzam, G.A., Haugen, A.C., Freedman, J.H. and Van Houten, B. Decline of nucleotide excision repair capacity in aging Caenorhabditis elegans. Genome Biol 8, R70. |
|  |  | Yanai, I., Baugh, L.R., Smith, J.J., Roehrig, C., Shen-Orr, S.S., Claggett, J.M., Hill, A.A., Slonim, D.K. and Hunter, C.P. Pairing of competitive and topologically distinct regulatory modules enhances patterned gene expression. Mol Syst Biol 4, 163. |
|  |  | Jiang, Y., Shi, H., Amin, N.M., Sultan, I. and Liu, J. Mesodermal expression of the C. elegans HMX homolog mls-2 requires the PBC homolog CEH-20. Mech Dev 125, 451-461. |
|  |  | Hueber, S.D., Weiller, G.F., Djordjevic, M.A. and Frickey, T. Improving Hox protein classification across the major model organisms. PLoS One 5, e10820. |
|  |  | Zhao, Z., Boyle, T.J., Liu, Z., Murray, J.I., Wood, W.B. and Waterston, R.H. A negative regulatory loop between microRNA and Hox gene controls posterior identities in Caenorhabditis elegans. PLoS Genet 6. |
| nsy-7 | C18F3.4 | Lesch, B.J., Gehrke, A.R., Bulyk, M.L. and Bargmann, C.I. Transcriptional regulation and stabilization of left-right neuronal identity in C. elegans. Genes Dev 23, 345-358. |
| pal-1 | C38D4.6 | Bürglin, T.R., Finney, M., Coulson, A. and Ruvkun, G. Caenorhabditis elegans has scores of homoeobox-containing genes. Nature 341, 239-243. |
|  |  | Waring, D.A. and Kenyon, C. Selective silencing of cell communication influences anteroposterior pattern formation in C. elegans. Cell 60, 123-131. |
|  |  | Waring, D.A. and Kenyon, C. Regulation of cellular responsiveness to inductive signals in the developing C. elegans nervous system. Nature 350, 712-715. |
|  |  | Emmons, S.W. From cell fates to morphology: developmental genetics of the Caenorhabditis elegans male tail. Bioessays 14, 309-316. |
|  |  | Naito, M., Kohara, Y. and Kurosawa, Y. Identification of a homeobox-containing gene located between lin-45 and unc-24 on chromosome IV in the nematode Caenorhabditis elegans. Nucleic Acids Res 20, 2967-2969. |
|  |  | Waring, D.A., Wrischnik, L. and Kenyon, C. Cell signals allow the expression of a pre-existent neural pattern in C. elegans. Development 116, 457-466. |
|  |  | Katz, W.S. and Sternberg, P.W. A plethora of intercellular signals during Caenorhabditis elegans development. Curr Opin Cell Biol 4, 939-947. |
|  |  | Chalfie, M. Homeobox genes in Caenorhabditis elegans. Curr Opin Genet Dev 3, 275-277. |
|  |  | Katsura, I. In search of new mutants in cell-signaling systems of the nematode Caenorhabditis elegans. Review. Genetica 88, 137-146. |
|  |  | Sternberg, P.W. Intercellular signaling and signal transduction in C. elegans. Annu Rev Genet 27, 497-521. |
|  |  | Yandell, M.D., Edgar, L.G. and Wood, W.B. Trimethylpsoralen induces small deletion mutations in Caenorhabditis elegans. Proc Natl Acad Sci U S A 91, 1381-1385. |
|  |  | Austin, J. and Kenyon, C. Cell contact regulates neuroblast formation in the Caenorhabditis elegans lateral epidermis. Development 120, 313-323. |
|  |  | Salser, S.J. and Kenyon, C. Patterning C. elegans: homeotic cluster genes, cell fates and cell migrations. Trends Genet 10, 159-164. |
|  |  | Stenico, M., Lloyd, A.T. and Sharp, P.M. Codon usage in Caenorhabditis elegans: delineation of translational selection and mutational biases. Nucleic Acids Res 22, 2437-2446. |
|  |  | Salser, S.J. and Kenyon, C. A C. elegans Hox gene switches on, off, on and off again to regulate proliferation, differentiation and morphogenesis. Development 122, 1651-1661. |
|  |  | Draper, B.W., Mello, C.C., Bowerman, B., Hardin, J. and Priess, J.R. MEX-3 is a KH domain protein that regulates blastomere identity in early C. elegans embryos. Cell 87, 205-216. |
|  |  | Hunter, C.P. and Kenyon, C. Spatial and temporal controls target pal-1 blastomere-specification activity to a single blastomere lineage in C. elegans embryos. Cell 87, 217-226. |
|  |  | Seydoux, G. Mechanisms of translational control in early development. Curr Opin Genet Dev 6, 555-561. |
|  |  | Guedes, S. and Priess, J.R. The C. elegans MEX-1 protein is present in germline blastomeres and is a P granule component. Development 124, 731-739. |
|  |  | Wrischnik, L.A. and Kenyon, C.J. The role of lin-22, a hairy/enhancer of split homolog, in patterning the peripheral nervous system of C. elegans. Development 124, 2875-2888. |
|  |  | Bowerman, B., Ingram, M.K. and Hunter, C.P. The maternal par genes and the segregation of cell fate specification activities in early Caenorhabditis elegans embryos. Development 124, 3815-3826. |
|  |  | Ahringer, J. Maternal control of a zygotic patterning gene in Caenorhabditis elegans. Development 124, 3865-3869. |
|  |  | Shi, Y. and Mello, C. A CBP/p300 homolog specifies multiple differentiation pathways in Caenorhabditis elegans. Genes Dev 12, 943-955. |
|  |  | Bowerman, B. Maternal control of pattern formation in early Caenorhabditis elegans embryos. Curr Top Dev Biol 39, 73-117. |
|  |  | Kenyon, C.J., Austin, J., Costa, M., Cowing, D.W., Harris, J.M., Honigberg, L., Hunter, C.P., Maloof, J.N., Muller-Immergluck, M.M., Salser, S.J. et al. The dance of the Hox genes: patterning the anteroposterior body axis of Caenorhabditis elegans. Cold Spring Harb Symp Quant Biol 62, 293-305. |
|  |  | Newman-Smith, E.D. and Rothman, J.H. The maternal-to-zygotic transition in embryonic patterning of Caenorhabditis elegans. Curr Opin Genet Dev 8, 472-480. |
|  |  | Lu, B., Jan, L.Y. and Jan, Y.N. Asymmetric cell division: lessons from flies and worms. Curr Opin Genet Dev 8, 392-399. |
|  |  | Hunter, C.P., Harris, J.M., Maloof, J.N. and Kenyon, C. Hox gene expression in a single Caenorhabditis elegans cell is regulated by a caudal homolog and intercellular signals that inhibit wnt signaling. Development 126, 805-814. |
|  |  | Van Auken, K., Weaver, D.C., Edgar, L.G. and Wood, W.B. Caenorhabditis elegans embryonic axial patterning requires two recently discovered posterior-group Hox genes. Proc Natl Acad Sci U S A 97, 4499-4503. |
|  |  | Schubert, C.M., Lin, R., de Vries, C.J., Plasterk, R.H. and Priess, J.R. MEX-5 and MEX-6 function to establish soma/germline asymmetry in early C. elegans embryos. Mol Cell 5, 671-682. |
|  |  | Kemphues, K. PARsing embryonic polarity. Cell 101, 345-348. |
|  |  | Corsi, A.K., Kostas, S.A., Fire, A. and Krause, M. Caenorhabditis elegans twist plays an essential role in non-striated muscle development. Development 127, 2041-2051. |
|  |  | Zhang, H. and Emmons, S.W. A C. elegans mediator protein confers regulatory selectivity on lineage-specific expression of a transcription factor gene. Genes Dev 14, 2161-2172. |
|  |  | Edgar, L.G., Carr, S., Wang, H. and Wood, W.B. Zygotic expression of the caudal homolog pal-1 is required for posterior patterning in Caenorhabditis elegans embryogenesis. Dev Biol 229, 71-88. |
|  |  | Kamath, R.S., Martinez-Campos, M., Zipperlen, P., Fraser, A.G. and Ahringer, J. Effectiveness of specific RNA-mediated interference through ingested double-stranded RNA in Caenorhabditis elegans. Genome Biol 2, RESEARCH0002. |
|  |  | Seydoux, G. and Schedl, T. The germline in C. elegans: origins, proliferation, and silencing. Int Rev Cytol 203, 139-185. |
|  |  | Kwon, J.Y. and Lee, J. Biological significance of a universally conserved transcription mediator in metazoan developmental signaling pathways. Development 128, 3095-3104. |
|  |  | Gomes, J.E., Encalada, S.E., Swan, K.A., Shelton, C.A., Carter, J.C. and Bowerman, B. The maternal gene spn-4 encodes a predicted RRM protein required for mitotic spindle orientation and cell fate patterning in early C. elegans embryos. Development 128, 4301-4314. |
|  |  | Huang, N.N., Mootz, D.E., Walhout, A.J., Vidal, M. and Hunter, C.P. MEX-3 interacting proteins link cell polarity to asymmetric gene expression in Caenorhabditis elegans. Development 129, 747-759. |
|  |  | Maduro, M.F. and Rothman, J.H. Making worm guts: the gene regulatory network of the Caenorhabditis elegans endoderm. Dev Biol 246, 68-85. |
|  |  | Labbe, J.C. and Goldstein, B. Embryonic development: a new SPN on cell fate specification. Curr Biol 12, R396-398. |
|  |  | Korswagen, H.C. Canonical and non-canonical Wnt signaling pathways in Caenorhabditis elegans: variations on a common signaling theme. Bioessays 24, 801-810. |
|  |  | Van Auken, K., Weaver, D., Robertson, B., Sundaram, M., Saldi, T., Edgar, L., Elling, U., Lee, M., Boese, Q. and Wood, W.B. Roles of the Homothorax/Meis/Prep homolog UNC-62 and the Exd/Pbx homologs CEH-20 and CEH-40 in C. elegans embryogenesis. Development 129, 5255-5268. |
|  |  | Ogura, K., Kishimoto, N., Mitani, S., Gengyo-Ando, K. and Kohara, Y. Translational control of maternal glp-1 mRNA by POS-1 and its interacting protein SPN-4 in Caenorhabditis elegans. Development 130, 2495-2503. |
|  |  | Lee, K.Z., Eizinger, A., Nandakumar, R., Schuster, S.C. and Sommer, R.J. Limited microsynteny between the genomes of Pristionchus pacificus and Caenorhabditis elegans. Nucleic Acids Res 31, 2553-2560. |
|  |  | Pires-daSilva, A. and Sommer, R.J. Finally, worm polycomb-like genes meet Hox regulation. Dev Cell 4, 770-772. |
|  |  | Ross, J.M. and Zarkower, D. Polycomb group regulation of Hox gene expression in C. elegans. Dev Cell 4, 891-901. |
|  |  | Kuersten, S. and Goodwin, E.B. The power of the 3' UTR: translational control and development. Nat Rev Genet 4, 626-637. |
|  |  | Aboobaker, A. and Blaxter, M. Hox gene evolution in nematodes: novelty conserved. Curr Opin Genet Dev 13, 593-598. |
|  |  | Walker, A.K., Shi, Y. and Blackwell, T.K. An extensive requirement for transcription factor IID-specific TAF-1 in Caenorhabditis elegans embryonic transcription. J Biol Chem 279, 15339-15347. |
|  |  | Couillault, C., Pujol, N., Reboul, J., Sabatier, L., Guichou, J.F., Kohara, Y. and Ewbank, J.J. TLR-independent control of innate immunity in Caenorhabditis elegans by the TIR domain adaptor protein TIR-1, an ortholog of human SARM. Nat Immunol 5, 488-494. |
|  |  | Mootz, D., Ho, D.M. and Hunter, C.P. The STAR/Maxi-KH domain protein GLD-1 mediates a developmental switch in the translational control of C. elegans PAL-1. Development 131, 3263-3272. |
|  |  | Pocock, R., Ahringer, J., Mitsch, M., Maxwell, S. and Woollard, A. A regulatory network of T-box genes and the even-skipped homologue vab-7 controls patterning and morphogenesis in C. elegans. Development 131, 2373-2385. |
|  |  | Wang, J.C., Walker, A., Blackwell, T.K. and Yamamoto, K.R. The Caenorhabditis elegans ortholog of TRAP240, CeTRAP240/let-19, selectively modulates gene expression and is essential for embryogenesis. J Biol Chem 279, 29270-29277. |
|  |  | Baugh, L.R., Hill, A.A., Claggett, J.M., Hill-Harfe, K., Wen, J.C., Slonim, D.K., Brown, E.L. and Hunter, C.P. The homeodomain protein PAL-1 specifies a lineage-specific regulatory network in the C. elegans embryo. Development 132, 1843-1854. |
|  |  | Fukushige, T. and Krause, M. The myogenic potency of HLH-1 reveals wide-spread developmental plasticity in early C. elegans embryos. Development 132, 1795-1805. |
|  |  | Yoda, A., Kouike, H., Okano, H. and Sawa, H. Components of the transcriptional Mediator complex are required for asymmetric cell division in C. elegans. Development 132, 1885-1893. |
|  |  | Konwerski, J., Senchuk, M., Petty, E., Lahaie, D. and Schisa, J.A. Cloning and expression analysis of pos-1 in the nematodes Caenorhabditis briggsae and Caenorhabditis remanei. Dev Dyn 233, 1006-1012. |
|  |  | Baugh, L.R., Wen, J.C., Hill, A.A., Slonim, D.K., Brown, E.L. and Hunter, C.P. Synthetic lethal analysis of Caenorhabditis elegans posterior embryonic patterning genes identifies conserved genetic interactions. Genome Biol 6, R45. |
|  |  | Ross, J.M., Kalis, A.K., Murphy, M.W. and Zarkower, D. The DM domain protein MAB-3 promotes sex-specific neurogenesis in C. elegans by regulating bHLH proteins. Dev Cell 8, 881-892. |
|  |  | Maduro, M.F., Kasmir, J.J., Zhu, J. and Rothman, J.H. The Wnt effector POP-1 and the PAL-1/Caudal homeoprotein collaborate with SKN-1 to activate C. elegans endoderm development. Dev Biol 285, 510-523. |
|  |  | Ciosk, R., DePalma, M. and Priess, J.R. Translational regulators maintain totipotency in the Caenorhabditis elegans germline. Science 311, 851-853. |
|  |  | Lee, M.H. and Schedl, T. RNA-binding proteins. WormBook, 1-13. |
|  |  | Gonczy, P. and Rose, L.S. Asymmetric cell division and axis formation in the embryo. WormBook, 1-20. |
|  |  | Chisholm, A.D. and Hardin, J. Epidermal morphogenesis. WormBook, 1-22. |
|  |  | Hodgkin, J. Genetic suppression. WormBook, 1-13. |
|  |  | Emmons, S.W. Male development. WormBook, 1-22. |
|  |  | Rhoads, R.E., Dinkova, T.D. and Korneeva, N.L. Mechanism and regulation of translation in C. elegans. WormBook, 1-18. |
|  |  | Priess, J.R. Notch signaling in the C. elegans embryo. WormBook, 1-16. |
|  |  | Okkema, P.G. and Krause, M. Transcriptional regulation. WormBook, 1-40. |
|  |  | Evans, T.C. and Hunter, C.P. Translational control of maternal RNAs. WormBook, 1-11. |
|  |  | Eisenmann, D.M. Wnt signaling. WormBook, 1-17. |
|  |  | Popovici, C., Berda, Y., Conchonaud, F., Harbis, A., Birnbaum, D. and Roubin, R. Direct and heterologous approaches to identify the LET-756/FGF interactome. BMC Genomics 7, 105. |
|  |  | Broitman-Maduro, G., Lin, K.T., Hung, W.W. and Maduro, M.F. Specification of the C. elegans MS blastomere by the T-box factor TBX-35. Development 133, 3097-3106. |
|  |  | Fukushige, T., Brodigan, T.M., Schriefer, L.A., Waterston, R.H. and Krause, M. Defining the transcriptional redundancy of early bodywall muscle development in C. elegans: evidence for a unified theory of animal muscle development. Genes Dev 20, 3395-3406. |
|  |  | Sommer, R.J. Pristionchus pacificus. WormBook, 1-8. |
|  |  | Vasudevan, S., Starostina, N.G. and Kipreos, E.T. The Caenorhabditis elegans cell-cycle regulator ZYG-11 defines a conserved family of CUL-2 complex components. EMBO Rep 8, 279-286. |
|  |  | Maurer, C.W., Chiorazzi, M. and Shaham, S. Timing of the onset of a developmental cell death is controlled by transcriptional induction of the C. elegans ced-3 caspase-encoding gene. Development 134, 1357-1368. |
|  |  | Witten, T.M. and Bonchev, D. Predicting aging/longevity-related genes in the nematode Caenorhabditis elegans. Chem Biodivers 4, 2639-2655. |
|  |  | Yanai, I., Baugh, L.R., Smith, J.J., Roehrig, C., Shen-Orr, S.S., Claggett, J.M., Hill, A.A., Slonim, D.K. and Hunter, C.P. Pairing of competitive and topologically distinct regulatory modules enhances patterned gene expression. Mol Syst Biol 4, 163. |
|  |  | Merritt, C., Rasoloson, D., Ko, D. and Seydoux, G. 3' UTRs are the primary regulators of gene expression in the C. elegans germline. Curr Biol 18, 1476-1482. |
|  |  | Farley, B.M., Pagano, J.M. and Ryder, S.P. RNA target specificity of the embryonic cell fate determinant POS-1. Rna 14, 2685-2697. |
|  |  | Lei, H., Liu, J., Fukushige, T., Fire, A. and Krause, M. Caudal-like PAL-1 directly activates the bodywall muscle module regulator hlh-1 in C. elegans to initiate the embryonic muscle gene regulatory network. Development 136, 1241-1249. |
|  |  | Henderson, M.A., Cronland, E., Dunkelbarger, S., Contreras, V., Strome, S. and Keiper, B.D. A germline-specific isoform of eIF4E (IFE-1) is required for efficient translation of stored mRNAs and maturation of both oocytes and sperm. J Cell Sci 122, 1529-1539. |
|  |  | Broitman-Maduro, G., Owraghi, M., Hung, W.W., Kuntz, S., Sternberg, P.W. and Maduro, M.F. The NK-2 class homeodomain factor CEH-51 and the T-box factor TBX-35 have overlapping function in C. elegans mesoderm development. Development 136, 2735-2746. |
|  |  | Pagano, J.M., Farley, B.M., Essien, K.I. and Ryder, S.P. RNA recognition by the embryonic cell fate determinant and germline totipotency factor MEX-3. Proc Natl Acad Sci U S A 106, 20252-20257. |
| pax-3 | F27E5.2 | Hobert, O. and Ruvkun, G. Pax genes in Caenorhabditis elegans: a new twist. Trends Genet 15, 214-216. |
|  |  | Furuya, M., Qadota, H., Chisholm, A.D. and Sugimoto, A. The C. elegans eyes absent ortholog EYA-1 is required for tissue differentiation and plays partially redundant roles with PAX-6. Dev Biol 286, 452-463. |
|  |  | Okkema, P.G. and Krause, M. Transcriptional regulation. WormBook, 1-40. |
|  |  | Meyer, J.N., Boyd, W.A., Azzam, G.A., Haugen, A.C., Freedman, J.H. and Van Houten, B. Decline of nucleotide excision repair capacity in aging Caenorhabditis elegans. Genome Biol 8, R70. |
|  |  | Yi, B. and Sommer, R.J. The pax-3 gene is involved in vulva formation in Pristionchus pacificus and is a target of the Hox gene lin-39. Development 134, 3111-3119. |
|  |  | Amin, N.M., Lim, S.E., Shi, H., Chan, T.L. and Liu, J. A conserved Six-Eya cassette acts downstream of Wnt signaling to direct non-myogenic versus myogenic fates in the C. elegans postembryonic mesoderm. Dev Biol 331, 350-360. |
|  |  | Zahreddine, H., Zhang, H., Diogon, M., Nagamatsu, Y. and Labouesse, M. CRT-1/calreticulin and the E3 ligase EEL-1/HUWE1 control hemidesmosome maturation in C. elegans development. Curr Biol 20, 322-327. |
|  |  | Yi, B., Bumbarger, D. and Sommer, R.J. Genetic evidence for pax-3 function in myogenesis in the nematode Pristionchus pacificus. Evol Dev 11, 669-679. |
| pha-2 | M6.3 | Avery, L. The genetics of feeding in Caenorhabditis elegans. Genetics 133, 897-917. |
|  |  | Morck, C., Axang, C. and Pilon, M. A genetic analysis of axon guidance in the C elegans pharynx. Dev Biol 260, 158-175. |
|  |  | Morck, C., Rauthan, M., Wagberg, F. and Pilon, M. pha-2 encodes the C. elegans ortholog of the homeodomain protein HEX and is required for the formation of the pharyngeal isthmus. Dev Biol 272, 403-418. |
|  |  | Pilon, M. and Morck, C. Development of Caenorhabditis elegans pharynx, with emphasis on its nervous system. Acta Pharmacol Sin 26, 396-404. |
|  |  | Okkema, P.G. and Krause, M. Transcriptional regulation. WormBook, 1-40. |
|  |  | Morck, C., Axang, C., Goksor, M. and Pilon, M. Misexpression of acetylcholinesterases in the C. elegans pha-2 mutant accompanies ultrastructural defects in pharyngeal muscle cells. Dev Biol 297, 446-460. |
|  |  | Morck, C. and Pilon, M. C. elegans feeding defective mutants have shorter body lengths and increased autophagy. BMC Dev Biol 6, 39. |
|  |  | Mango, S.E. The C. elegans pharynx: a model for organogenesis. WormBook, 1-26. |
|  |  | Axang, C., Rauthan, M., Hall, D.H. and Pilon, M. The twisted pharynx phenotype in C. elegans. BMC Dev Biol 7, 61. |
|  |  | Axang, C., Rauthan, M., Hall, D.H. and Pilon, M. Developmental genetics of the C. elegans pharyngeal neurons NSML and NSMR. BMC Dev Biol 8, 38. |
| php-3 | Y75B8A.1 | Van Auken, K., Weaver, D.C., Edgar, L.G. and Wood, W.B. Caenorhabditis elegans embryonic axial patterning requires two recently discovered posterior-group Hox genes. Proc Natl Acad Sci U S A 97, 4499-4503. |
|  |  | Van Auken, K., Weaver, D., Robertson, B., Sundaram, M., Saldi, T., Edgar, L., Elling, U., Lee, M., Boese, Q. and Wood, W.B. Roles of the Homothorax/Meis/Prep homolog UNC-62 and the Exd/Pbx homologs CEH-20 and CEH-40 in C. elegans embryogenesis. Development 129, 5255-5268. |
|  |  | Aboobaker, A.A. and Blaxter, M.L. Hox Gene Loss during Dynamic Evolution of the Nematode Cluster. Curr Biol 13, 37-40. |
|  |  | Aboobaker, A. and Blaxter, M. Hox gene evolution in nematodes: novelty conserved. Curr Opin Genet Dev 13, 593-598. |
|  |  | Herman, M.A. Hermaphrodite cell-fate specification. WormBook, 1-16. |
|  |  | Fitch, D.H. Introduction to nematode evolution and ecology. WormBook, 1-8. |
|  |  | Okkema, P.G. and Krause, M. Transcriptional regulation. WormBook, 1-40. |
|  |  | Arata, Y., Kouike, H., Zhang, Y., Herman, M.A., Okano, H. and Sawa, H. Wnt signaling and a Hox protein cooperatively regulate psa-3/Meis to determine daughter cell fate after asymmetric cell division in C. elegans. Dev Cell 11, 105-115. |
|  |  | Jiang, Y., Shi, H., Amin, N.M., Sultan, I. and Liu, J. Mesodermal expression of the C. elegans HMX homolog mls-2 requires the PBC homolog CEH-20. Mech Dev 125, 451-461. |
|  |  | Hueber, S.D., Weiller, G.F., Djordjevic, M.A. and Frickey, T. Improving Hox protein classification across the major model organisms. PLoS One 5, e10820. |
| tab-1 | F31E8.3 | Wolinsky, E. and Way, J. The behavioral genetics of Caenorhabditis elegans. Behav Genet 20, 169-189. |
|  |  | Nelson, C.E., Hersh, B.M. and Carroll, S.B. The regulatory content of intergenic DNA shapes genome architecture. Genome Biol 5, R25. |
|  |  | Okkema, P.G. and Krause, M. Transcriptional regulation. WormBook, 1-40. |
|  |  | Broitman-Maduro, G., Owraghi, M., Hung, W.W., Kuntz, S., Sternberg, P.W. and Maduro, M.F. The NK-2 class homeodomain factor CEH-51 and the T-box factor TBX-35 have overlapping function in C. elegans mesoderm development. Development 136, 2735-2746. |
| ttx-1 | Y113G7A.6 | Perkins, L.A., Hedgecock, E.M., Thomson, J.N. and Culotti, J.G. Mutant sensory cilia in the nematode Caenorhabditis elegans. Dev Biol 117, 456-487. |
|  |  | Bargmann, C.I. Genetic and cellular analysis of behavior in C. elegans. Annu Rev Neurosci 16, 47-71. |
|  |  | Mori, I. and Ohshima, Y. Neural regulation of thermotaxis in Caenorhabditis elegans. Nature 376, 344-348. |
|  |  | Mori, I. and Ohshima, Y. Molecular neurogenetics of chemotaxis and thermotaxis in the nematode Caenorhabditis elegans. Bioessays 19, 1055-1064. |
|  |  | Apfeld, J. and Kenyon, C. Regulation of lifespan by sensory perception in Caenorhabditis elegans. Nature 402, 804-809. |
|  |  | Ailion, M. and Thomas, J.H. Dauer formation induced by high temperatures in Caenorhabditis elegans. Genetics 156, 1047-1067. |
|  |  | Satterlee, J.S., Sasakura, H., Kuhara, A., Berkeley, M., Mori, I. and Sengupta, P. Specification of thermosensory neuron fate in C. elegans requires ttx-1, a homolog of otd/Otx. Neuron 31, 943-956. |
|  |  | Jorgensen, E.M. and Mango, S.E. The art and design of genetic screens: caenorhabditis elegans. Nat Rev Genet 3, 356-369. |
|  |  | Ryu, W.S. and Samuel, A.D. Thermotaxis in Caenorhabditis elegans analyzed by measuring responses to defined Thermal stimuli. J Neurosci 22, 5727-5733. |
|  |  | Zariwala, H.A., Miller, A.C., Faumont, S. and Lockery, S.R. Step response analysis of thermotaxis in Caenorhabditis elegans. J Neurosci 23, 4369-4377. |
|  |  | Ailion, M. and Thomas, J.H. Isolation and characterization of high-temperature-induced Dauer formation mutants in Caenorhabditis elegans. Genetics 165, 127-144. |
|  |  | Lanjuin, A., VanHoven, M.K., Bargmann, C.I., Thompson, J.K. and Sengupta, P. Otx/otd homeobox genes specify distinct sensory neuron identities in C. elegans. Dev Cell 5, 621-633. |
|  |  | Satterlee, J.S., Ryu, W.S. and Sengupta, P. The CMK-1 CaMKI and the TAX-4 Cyclic nucleotide-gated channel regulate thermosensory neuron gene expression and function in C. elegans. Curr Biol 14, 62-68. |
|  |  | Melkman, T. and Sengupta, P. The worm's sense of smell. Development of functional diversity in the chemosensory system of Caenorhabditis elegans. Dev Biol 265, 302-319. |
|  |  | Wakabayashi, T., Kitagawa, I. and Shingai, R. Neurons regulating the duration of forward locomotion in Caenorhabditis elegans. Neurosci Res 50, 103-111. |
|  |  | Colosimo, M.E., Brown, A., Mukhopadhyay, S., Gabel, C., Lanjuin, A.E., Samuel, A.D. and Sengupta, P. Identification of thermosensory and olfactory neuron-specific genes via expression profiling of single neuron types. Curr Biol 14, 2245-2251. |
|  |  | Shtonda, B.B. and Avery, L. Dietary choice behavior in Caenorhabditis elegans. J Exp Biol 209, 89-102. |
|  |  | Ito, H., Inada, H. and Mori, I. Quantitative analysis of thermotaxis in the nematode Caenorhabditis elegans. J Neurosci Methods 154, 45-52. |
|  |  | Hobert, O. Specification of the nervous system. WormBook, 1-19. |
|  |  | Savage-Dunn, C. TGF-beta signaling. WormBook, 1-12. |
|  |  | Okkema, P.G. and Krause, M. Transcriptional regulation. WormBook, 1-40. |
|  |  | Bargmann, C.I. Chemosensation in C. elegans. WormBook, 1-29. |
|  |  | Gabel, C.V., Gabel, H., Pavlichin, D., Kao, A., Clark, D.A. and Samuel, A.D. Neural circuits mediate electrosensory behavior in Caenorhabditis elegans. J Neurosci 27, 7586-7596. |
|  |  | Adachi, R., Wakabayashi, T., Oda, N. and Shingai, R. Modulation of Caenorhabditis elegans chemotaxis by cultivation and assay temperatures. Neurosci Res 60, 300-306. |
|  |  | Prahlad, V., Cornelius, T. and Morimoto, R.I. Regulation of the cellular heat shock response in Caenorhabditis elegans by thermosensory neurons. Science 320, 811-814. |
|  |  | Hallem, E.A. and Sternberg, P.W. Acute carbon dioxide avoidance in Caenorhabditis elegans. Proc Natl Acad Sci U S A 105, 8038-8043. |
|  |  | Bacaj, T., Tevlin, M., Lu, Y. and Shaham, S. Glia are essential for sensory organ function in C. elegans. Science 322, 744-747. |
|  |  | Jovelin, R. Rapid sequence evolution of transcription factors controlling neuron differentiation in Caenorhabditis. Mol Biol Evol 26, 2373-2386. |
|  |  | Ben Arous, J., Laffont, S. and Chatenay, D. Molecular and sensory basis of a food related two-state behavior in C. elegans. PLoS One 4, e7584. |
|  |  | Li, Y., Ye, H., Du, M., Zhang, Y., Ye, B., Pu, Y. and Wang, D. Induction of chemotaxis to sodium chloride and diacetyl and thermotaxis defects by microcystin-LR exposure in nematode Caenorhabditis elegans. J Environ Sci (China) 21, 971-979. |
|  |  | Jurado, P., Kodama, E., Tanizawa, Y. and Mori, I. Distinct thermal migration behaviors in response to different thermal gradients in Caenorhabditis elegans. Genes Brain Behav 9, 120-127. |
| ttx-3 | C40H5.5 | Mori, I. and Ohshima, Y. Neural regulation of thermotaxis in Caenorhabditis elegans. Nature 376, 344-348. |
|  |  | Hobert, O., Mori, I., Yamashita, Y., Honda, H., Ohshima, Y., Liu, Y. and Ruvkun, G. Regulation of interneuron function in the C. elegans thermoregulatory pathway by the ttx-3 LIM homeobox gene. Neuron 19, 345-357. |
|  |  | Mori, I. and Ohshima, Y. Molecular neurogenetics of chemotaxis and thermotaxis in the nematode Caenorhabditis elegans. Bioessays 19, 1055-1064. |
|  |  | Shibatohge, M., Kariya, K., Liao, Y., Hu, C.D., Watari, Y., Goshima, M., Shima, F. and Kataoka, T. Identification of PLC210, a Caenorhabditis elegans phospholipase C, as a putative effector of Ras. J Biol Chem 273, 6218-6222. |
|  |  | Hobert, O., D'Alberti, T., Liu, Y. and Ruvkun, G. Control of neural development and function in a thermoregulatory network by the LIM homeobox gene lin-11. J Neurosci 18, 2084-2096. |
|  |  | Hobert, O., Tessmar, K. and Ruvkun, G. The Caenorhabditis elegans lim-6 LIM homeobox gene regulates neurite outgrowth and function of particular GABAergic neurons. Development 126, 1547-1562. |
|  |  | Troemel, E.R., Sagasti, A. and Bargmann, C.I. Lateral signaling mediated by axon contact and calcium entry regulates asymmetric odorant receptor expression in C. elegans. Cell 99, 387-398. |
|  |  | Davidson, E.H. and Ruvkun, G. Themes from a NASA workshop on gene regulatory processes in development and evolution. J Exp Zool 285, 104-115. |
|  |  | Mori, I. Genetics of chemotaxis and thermotaxis in the nematode Caenorhabditis elegans. Annu Rev Genet 33, 399-422. |
|  |  | Cassata, G., Kagoshima, H., Andachi, Y., Kohara, Y., Dürrenberger, M.B., Hall, D.H. and Bürglin, T.R. The LIM homeobox gene ceh-14 confers thermosensory function to the AFD neurons in Caenorhabditis elegans. Neuron 25, 587-597. |
|  |  | Wittenburg, N., Eimer, S., Lakowski, B., Rohrig, S., Rudolph, C. and Baumeister, R. Presenilin is required for proper morphology and function of neurons in C. elegans. Nature 406, 306-309. |
|  |  | Cassata, G., Kuhn, F., Witmer, A., Kirchhofer, R. and Bürglin, T.R. A steep thermal gradient thermotaxis assay for the nematode Caenorhabditis elegans. Genesis 27, 141-144. |
|  |  | Cassata, G., Röhrig, S., Kuhn, F., Hauri, H.P., Baumeister, R. and Bürglin, T.R. The Caenorhabditis elegans Ldb/NLI/Clim orthologue ldb-1 is required for neuronal function. Dev Biol 226, 45-56. |
|  |  | Sarafi-Reinach, T.R. and Sengupta, P. The forkhead domain gene unc-130 generates chemosensory neuron diversity in C. elegans. Genes Dev 14, 2472-2485. |
|  |  | Altun-Gultekin, Z., Andachi, Y., Tsalik, E.L., Pilgrim, D., Kohara, Y. and Hobert, O. A regulatory cascade of three homeobox genes, ceh-10, ttx-3 and ceh-23, controls cell fate specification of a defined interneuron class in C. elegans. Development 128, 1951-1969. |
|  |  | Sarafi-Reinach, T.R., Melkman, T., Hobert, O. and Sengupta, P. The lin-11 LIM homeobox gene specifies olfactory and chemosensory neuron fates in C. elegans. Development 128, 3269-3281. |
|  |  | Kuhara, A., Inada, H., Katsura, I. and Mori, I. Negative regulation and gain control of sensory neurons by the C. elegans calcineurin TAX-6. Neuron 33, 751-763. |
|  |  | Bulow, H.E., Berry, K.L., Topper, L.H., Peles, E. and Hobert, O. Heparan sulfate proteoglycan-dependent induction of axon branching and axon misrouting by the Kallmann syndrome gene kal-1. Proc Natl Acad Sci U S A 99, 6346-6351. |
|  |  | MacColl, G., Bouloux, P. and Quinton, R. Kallmann syndrome: adhesion, afferents, and anosmia. Neuron 34, 675-678. |
|  |  | Ishihara, T., Iino, Y., Mohri, A., Mori, I., Gengyo-Ando, K., Mitani, S. and Katsura, I. HEN-1, a secretory protein with an LDL receptor motif, regulates sensory integration and learning in Caenorhabditis elegans. Cell 109, 639-649. |
|  |  | Ryu, W.S. and Samuel, A.D. Thermotaxis in Caenorhabditis elegans analyzed by measuring responses to defined Thermal stimuli. J Neurosci 22, 5727-5733. |
|  |  | Eimer, S., Donhauser, R. and Baumeister, R. The Caenorhabditis elegans presenilin sel-12 is required for mesodermal patterning and muscle function. Dev Biol 251, 178-192. |
|  |  | Zariwala, H.A., Miller, A.C., Faumont, S. and Lockery, S.R. Step response analysis of thermotaxis in Caenorhabditis elegans. J Neurosci 23, 4369-4377. |
|  |  | Yamada, Y. and Ohshima, Y. Distribution and movement of Caenorhabditis elegans on a thermal gradient. J Exp Biol 206, 2581-2593. |
|  |  | Tsalik, E.L., Niacaris, T., Wenick, A.S., Pau, K., Avery, L. and Hobert, O. LIM homeobox gene-dependent expression of biogenic amine receptors in restricted regions of the C. elegans nervous system. Dev Biol 263, 81-102. |
|  |  | Satterlee, J.S., Ryu, W.S. and Sengupta, P. The CMK-1 CaMKI and the TAX-4 Cyclic nucleotide-gated channel regulate thermosensory neuron gene expression and function in C. elegans. Curr Biol 14, 62-68. |
|  |  | Wenick, A.S. and Hobert, O. Genomic cis-regulatory architecture and trans-acting regulators of a single interneuron-specific gene battery in C. elegans. Dev Cell 6, 757-770. |
|  |  | Wakabayashi, T., Kitagawa, I. and Shingai, R. Neurons regulating the duration of forward locomotion in Caenorhabditis elegans. Neurosci Res 50, 103-111. |
|  |  | Gray, J.M., Hill, J.J. and Bargmann, C.I. A circuit for navigation in Caenorhabditis elegans. Proc Natl Acad Sci U S A 102, 3184-3191. |
|  |  | Okochi, Y., Kimura, K.D., Ohta, A. and Mori, I. Diverse regulation of sensory signaling by C. elegans nPKC-epsilon/eta TTX-4. Embo J 24, 2127-2137. |
|  |  | Hirotsu, T. and Iino, Y. Neural circuit-dependent odor adaptation in C. elegans is regulated by the Ras-MAPK pathway. Genes Cells 10, 517-530. |
|  |  | Remy, J.J. and Hobert, O. An interneuronal chemoreceptor required for olfactory imprinting in C. elegans. Science 309, 787-790. |
|  |  | Shtonda, B.B. and Avery, L. Dietary choice behavior in Caenorhabditis elegans. J Exp Biol 209, 89-102. |
|  |  | Ito, H., Inada, H. and Mori, I. Quantitative analysis of thermotaxis in the nematode Caenorhabditis elegans. J Neurosci Methods 154, 45-52. |
|  |  | Suzuki, Y. and Han, M. Genetic redundancy masks diverse functions of the tumor suppressor gene PTEN during C. elegans development. Genes Dev 20, 423-428. |
|  |  | Faumont, S., Boulin, T., Hobert, O. and Lockery, S.R. Developmental regulation of whole cell capacitance and membrane current in identified interneurons in C. elegans. J Neurophysiol 95, 3665-3673. |
|  |  | Chung, S.H., Clark, D.A., Gabel, C.V., Mazur, E. and Samuel, A.D. The role of the AFD neuron in C. elegans thermotaxis analyzed using femtosecond laser ablation. BMC Neurosci 7, 30. |
|  |  | Hobert, O. Specification of the nervous system. WormBook, 1-19. |
|  |  | Okkema, P.G. and Krause, M. Transcriptional regulation. WormBook, 1-40. |
|  |  | Boulin, T., Etchberger, J.F. and Hobert, O. Reporter gene fusions. WormBook, 1-23. |
|  |  | Hoshi, K. and Shingai, R. Computer-driven automatic identification of locomotion states in Caenorhabditis elegans. J Neurosci Methods 157, 355-363. |
|  |  | Clark, D.A., Biron, D., Sengupta, P. and Samuel, A.D. The AFD sensory neurons encode multiple functions underlying thermotactic behavior in Caenorhabditis elegans. J Neurosci 26, 7444-7451. |
|  |  | Luo, L., Clark, D.A., Biron, D., Mahadevan, L. and Samuel, A.D. Sensorimotor control during isothermal tracking in Caenorhabditis elegans. J Exp Biol 209, 4652-4662. |
|  |  | Clark, D.A., Gabel, C.V., Lee, T.M. and Samuel, A.D. Short-term adaptation and temporal processing in the cryophilic response of Caenorhabditis elegans. J Neurophysiol 97, 1903-1910. |
|  |  | Bargmann, C.I. Chemosensation in C. elegans. WormBook, 1-29. |
|  |  | Duerr, J.S. Immunohistochemistry. WormBook, 1-61. |
|  |  | Torayama, I., Ishihara, T. and Katsura, I. Caenorhabditis elegans integrates the signals of butanone and food to enhance chemotaxis to butanone. J Neurosci 27, 741-750. |
|  |  | Gabel, C.V., Gabel, H., Pavlichin, D., Kao, A., Clark, D.A. and Samuel, A.D. Neural circuits mediate electrosensory behavior in Caenorhabditis elegans. J Neurosci 27, 7586-7596. |
|  |  | Tan, P.L., Barr, T., Inglis, P.N., Mitsuma, N., Huang, S.M., Garcia-Gonzalez, M.A., Bradley, B.A., Coforio, S., Albrecht, P.J., Watnick, T. et al. Loss of Bardet Biedl syndrome proteins causes defects in peripheral sensory innervation and function. Proc Natl Acad Sci U S A 104, 17524-17529. |
|  |  | Chi, C.A., Clark, D.A., Lee, S., Biron, D., Luo, L., Gabel, C.V., Brown, J., Sengupta, P. and Samuel, A.D. Temperature and food mediate long-term thermotactic behavioral plasticity by association-independent mechanisms in C. elegans. J Exp Biol 210, 4043-4052. |
|  |  | Kuhara, A., Okumura, M., Kimata, T., Tanizawa, Y., Takano, R., Kimura, K.D., Inada, H., Matsumoto, K. and Mori, I. Temperature sensing by an olfactory neuron in a circuit controlling behavior of C. elegans. Science 320, 803-807. |
|  |  | Prahlad, V., Cornelius, T. and Morimoto, R.I. Regulation of the cellular heat shock response in Caenorhabditis elegans by thermosensory neurons. Science 320, 811-814. |
|  |  | Reiner, D.J., Ailion, M., Thomas, J.H. and Meyer, B.J. C. elegans anaplastic lymphoma kinase ortholog SCD-2 controls dauer formation by modulating TGF-beta signaling. Curr Biol 18, 1101-1109. |
|  |  | Kang, C. and Avery, L. Systemic regulation of starvation response in Caenorhabditis elegans. Genes Dev 23, 12-17. |
|  |  | Bertrand, V. and Hobert, O. Linking asymmetric cell division to the terminal differentiation program of postmitotic neurons in C. elegans. Dev Cell 16, 563-575. |
|  |  | Iino, Y. and Yoshida, K. Parallel use of two behavioral mechanisms for chemotaxis in Caenorhabditis elegans. J Neurosci 29, 5370-5380. |
|  |  | Jovelin, R. Rapid sequence evolution of transcription factors controlling neuron differentiation in Caenorhabditis. Mol Biol Evol 26, 2373-2386. |
|  |  | Ben Arous, J., Laffont, S. and Chatenay, D. Molecular and sensory basis of a food related two-state behavior in C. elegans. PLoS One 4, e7584. |
|  |  | Li, Y., Ye, H., Du, M., Zhang, Y., Ye, B., Pu, Y. and Wang, D. Induction of chemotaxis to sodium chloride and diacetyl and thermotaxis defects by microcystin-LR exposure in nematode Caenorhabditis elegans. J Environ Sci (China) 21, 971-979. |
|  |  | Jurado, P., Kodama, E., Tanizawa, Y. and Mori, I. Distinct thermal migration behaviors in response to different thermal gradients in Caenorhabditis elegans. Genes Brain Behav 9, 120-127. |
| unc-4 | F26C11.2 | Brenner, S. The genetics of Caenorhabditis elegans. Genetics 77, 71-94. |
|  |  | Herman, R.K. Crossover suppressors and balanced recessive lethals in Caenorhabditis elegans. Genetics 88, 49-65. |
|  |  | Hodgkin, J.A. and Brenner, S. Mutations causing transformation of sexual phenotype in the nematode Caenorhabditis elegans. Genetics 86, 275-287. |
|  |  | Waterston, R.H., Thomson, J.N. and Brenner, S. Mutants with altered muscle structure of Caenorhabditis elegans. Dev Biol 77, 271-302. |
|  |  | Hodgkin, J. Male Phenotypes and Mating Efficiency in CAENORHABDITIS ELEGANS. Genetics 103, 43-64. |
|  |  | Sigurdson, D.C., Spanier, G.J. and Herman, R.K. Caenorhabditis elegans deficiency mapping. Genetics 108, 331-345. |
|  |  | Huang, X.Y., Barrios, L.A., Vonkhorporn, P., Honda, S., Albertson, D.G. and Hecht, R.M. Genomic organization of the glyceraldehyde-3-phosphate dehydrogenase gene family of Caenorhabditis elegans. J Mol Biol 206, 411-424. |
|  |  | Bürglin, T.R., Finney, M., Coulson, A. and Ruvkun, G. Caenorhabditis elegans has scores of homoeobox-containing genes. Nature 341, 239-243. |
|  |  | Stewart, H.I., Rosenbluth, R.E. and Baillie, D.L. Most ultraviolet irradiation induced mutations in the nematode Caenorhabditis elegans are chromosomal rearrangements. Mutat Res 249, 37-54. |
|  |  | Beh, C.T., Ferrari, D.C., Chung, M.A. and McGhee, J.D. An acid phosphatase as a biochemical marker for intestinal development in the nematode Caenorhabditis elegans. Dev Biol 147, 133-143. |
|  |  | Hodgkin, J. The interactive worm. Eighth international C. elegans Meeting, Madison, WI, USA, June 1-5, 1991. New Biol 3, 951-954. |
|  |  | White, J.G., Southgate, E. and Thomson, J.N. Mutations in the Caenorhabditis elegans unc-4 gene alter the synaptic input to ventral cord motor neurons. Nature 355, 838-841. |
|  |  | Miller, D.M., Shen, M.M., Shamu, C.E., Bürglin, T.R., Ruvkun, G., Dubois, M.L., Ghee, M. and Wilson, L. C. elegans unc-4 gene encodes a homeodomain protein that determines the pattern of synaptic input to specific motor neurons. Nature 355, 841-845. |
|  |  | Williams, B.D., Schrank, B., Huynh, C., Shownkeen, R. and Waterston, R.H. A genetic mapping system in Caenorhabditis elegans based on polymorphic sequence-tagged sites. Genetics 131, 609-624. |
|  |  | Hill, R.J. and Sternberg, P.W. The gene lin-3 encodes an inductive signal for vulval development in C. elegans. Nature 358, 470-476. |
|  |  | Johnson, T.E. and Lithgow, G.J. The search for the genetic basis of aging: the identification of gerontogenes in the nematode Caenorhabditis elegans. J Am Geriatr Soc 40, 936-945. |
|  |  | Aroian, R.V., Levy, A.D., Koga, M., Ohshima, Y., Kramer, J.M. and Sternberg, P.W. Splicing in Caenorhabditis elegans does not require an AG at the 3' splice acceptor site. Mol Cell Biol 13, 626-637. |
|  |  | Katz, W.S. and Sternberg, P.W. A plethora of intercellular signals during Caenorhabditis elegans development. Curr Opin Cell Biol 4, 939-947. |
|  |  | Avery, L. The genetics of feeding in Caenorhabditis elegans. Genetics 133, 897-917. |
|  |  | Albertson, D.G. Mapping chromosome rearrangement breakpoints to the physical map of Caenorhabditis elegans by fluorescent in situ hybridization. Genetics 134, 211-219. |
|  |  | Chalfie, M. Homeobox genes in Caenorhabditis elegans. Curr Opin Genet Dev 3, 275-277. |
|  |  | Seydoux, G., Savage, C. and Greenwald, I. Isolation and characterization of mutations causing abnormal eversion of the vulva in Caenorhabditis elegans. Dev Biol 157, 423-436. |
|  |  | Miller, D.M., 3rd, Niemeyer, C.J. and Chitkara, P. Dominant unc-37 mutations suppress the movement defect of a homeodomain mutation in unc-4, a neural specificity gene in Caenorhabditis elegans. Genetics 135, 741-753. |
|  |  | Waddle, J.A., Cooper, J.A. and Waterston, R.H. The alpha and beta subunits of nematode actin capping protein function in yeast. Mol Biol Cell 4, 907-917. |
|  |  | Walthall, W.W., Li, L., Plunkett, J.A. and Hsu, C.Y. Changing synaptic specificities in the nervous system of Caenorhabditis elegans: differentiation of the DD motoneurons. J Neurobiol 24, 1589-1599. |
|  |  | Stenico, M., Lloyd, A.T. and Sharp, P.M. Codon usage in Caenorhabditis elegans: delineation of translational selection and mutational biases. Nucleic Acids Res 22, 2437-2446. |
|  |  | Duggan, A. and Chalfie, M. Control of neuronal development in Caenorhabditis elegans. Curr Opin Neurobiol 5, 6-9. |
|  |  | Walthall, W.W. Repeating patterns of motoneurons in nematodes: the origin of segmentation? Exs 72, 61-75. |
|  |  | Miller, D.M., 3rd and Niemeyer, C.J. Expression of the unc-4 homeoprotein in Caenorhabditis elegans motor neurons specifies presynaptic input. Development 121, 2877-2886. |
|  |  | Pflugrad, A., Meir, J.Y., Barnes, T.M. and Miller, D.M., 3rd The Groucho-like transcription factor UNC-37 functions with the neural specificity gene unc-4 to govern motor neuron identity in C. elegans. Development 124, 1699-1709. |
|  |  | Harbinder, S., Tavernarakis, N., Herndon, L.A., Kinnell, M., Xu, S.Q., Fire, A. and Driscoll, M. Genetically targeted cell disruption in Caenorhabditis elegans. Proc Natl Acad Sci U S A 94, 13128-13133. |
|  |  | Prasad, B.C., Ye, B., Zackhary, R., Schrader, K., Seydoux, G. and Reed, R.R. unc-3, a gene required for axonal guidance in Caenorhabditis elegans, encodes a member of the O/E family of transcription factors. Development 125, 1561-1568. |
|  |  | Ruvkun, G. and Hobert, O. The taxonomy of developmental control in Caenorhabditis elegans. Science 282, 2033-2041. |
|  |  | Chalfie, M. and Jorgensen, E.M. C. elegans neuroscience: genetics to genome. Trends Genet 14, 506-512. |
|  |  | Nonet, M.L. Visualization of synaptic specializations in live C. elegans with synaptic vesicle protein-GFP fusions. J Neurosci Methods 89, 33-40. |
|  |  | Winnier, A.R., Meir, J.Y., Ross, J.M., Tavernarakis, N., Driscoll, M., Ishihara, T., Katsura, I. and Miller, D.M., 3rd UNC-4/UNC-37-dependent repression of motor neuron-specific genes controls synaptic choice in Caenorhabditis elegans. Genes Dev 13, 2774-2786. |
|  |  | Zhao, H. and Nonet, M.L. A retrograde signal is involved in activity-dependent remodeling at a C. elegans neuromuscular junction. Development 127, 1253-1266. |
|  |  | Gems, D. and Riddle, D.L. Genetic, behavioral and environmental determinants of male longevity in Caenorhabditis elegans. Genetics 154, 1597-1610. |
|  |  | Hekimi, S. Crossroads of aging in the nematode Caenorhabditis elegans. Results Probl Cell Differ 29, 81-112. |
|  |  | Pujol, N., Torregrossa, P., Ewbank, J.J. and Brunet, J.F. The homeodomain protein CePHOX2/CEH-17 controls antero-posterior axonal growth in C. elegans. Development 127, 3361-3371. |
|  |  | Hallam, S., Singer, E., Waring, D. and Jin, Y. The C. elegans NeuroD homolog cnd-1 functions in multiple aspects of motor neuron fate specification. Development 127, 4239-4252. |
|  |  | Waggoner, L.E., Dickinson, K.A., Poole, D.S., Tabuse, Y., Miwa, J. and Schafer, W.R. Long-term nicotine adaptation in Caenorhabditis elegans involves PKC-dependent changes in nicotinic receptor abundance. J Neurosci 20, 8802-8811. |
|  |  | Lickteig, K.M., Duerr, J.S., Frisby, D.L., Hall, D.H., Rand, J.B. and Miller, D.M., 3rd Regulation of neurotransmitter vesicles by the homeodomain protein UNC-4 and its transcriptional corepressor UNC-37/groucho in Caenorhabditis elegans cholinergic motor neurons. J Neurosci 21, 2001-2014. |
|  |  | Fares, H. and Greenwald, I. Genetic analysis of endocytosis in Caenorhabditis elegans: coelomocyte uptake defective mutants. Genetics 159, 133-145. |
|  |  | Nass, R., Hall, D.H., Miller, D.M., 3rd and Blakely, R.D. Neurotoxin-induced degeneration of dopamine neurons in Caenorhabditis elegans. Proc Natl Acad Sci U S A 99, 3264-3269. |
|  |  | Jorgensen, E.M. and Mango, S.E. The art and design of genetic screens: caenorhabditis elegans. Nat Rev Genet 3, 356-369. |
|  |  | Simmer, F., Tijsterman, M., Parrish, S., Koushika, S.P., Nonet, M.L., Fire, A., Ahringer, J. and Plasterk, R.H. Loss of the putative RNA-directed RNA polymerase RRF-3 makes C. elegans hypersensitive to RNAi. Curr Biol 12, 1317-1319. |
|  |  | Thor, S. and Thomas, J.B. Motor neuron specification in worms, flies and mice: conserved and 'lost' mechanisms. Curr Opin Genet Dev 12, 558-564. |
|  |  | Strange, K. From genes to integrative physiology: ion channel and transporter biology in Caenorhabditis elegans. Physiol Rev 83, 377-415. |
|  |  | Bany, I.A., Dong, M.Q. and Koelle, M.R. Genetic and cellular basis for acetylcholine inhibition of Caenorhabditis elegans egg-laying behavior. J Neurosci 23, 8060-8069. |
|  |  | Ailion, M. and Thomas, J.H. Isolation and characterization of high-temperature-induced Dauer formation mutants in Caenorhabditis elegans. Genetics 165, 127-144. |
|  |  | Wacker, I., Schwarz, V., Hedgecock, E.M. and Hutter, H. zag-1, a Zn-finger homeodomain transcription factor controlling neuronal differentiation and axon outgrowth in C. elegans. Development 130, 3795-3805. |
|  |  | Moghal, N., Garcia, L.R., Khan, L.A., Iwasaki, K. and Sternberg, P.W. Modulation of EGF receptor-mediated vulva development by the heterotrimeric G-protein Galphaq and excitable cells in C. elegans. Development 130, 4553-4566. |
|  |  | Yin, X., Gower, N.J., Baylis, H.A. and Strange, K. Inositol 1,4,5-trisphosphate signaling regulates rhythmic contractile activity of myoepithelial sheath cells in Caenorhabditis elegans. Mol Biol Cell 15, 3938-3949. |
|  |  | Williams, S.N., Locke, C.J., Braden, A.L., Caldwell, K.A. and Caldwell, G.A. Epileptic-like convulsions associated with LIS-1 in the cytoskeletal control of neurotransmitter signaling in Caenorhabditis elegans. Hum Mol Genet 13, 2043-2059. |
|  |  | Fox, R.M., Von Stetina, S.E., Barlow, S.J., Shaffer, C., Olszewski, K.L., Moore, J.H., Dupuy, D., Vidal, M. and Miller, D.M., 3rd A gene expression fingerprint of C. elegans embryonic motor neurons. BMC Genomics 6, 42. |
|  |  | Johnson, N.M., Behm, C.A. and Trowell, S.C. Heritable and inducible gene knockdown in C. elegans using Wormgate and the ORFeome. Gene 359, 26-34. |
|  |  | Chu, K.W., Chan, S.K. and Chow, K.L. Improvement of heavy metal stress and toxicity assays by coupling a transgenic reporter in a mutant nematode strain. Aquat Toxicol 74, 320-332. |
|  |  | Hobert, O. Specification of the nervous system. WormBook, 1-19. |
|  |  | Jin, Y. Synaptogenesis. WormBook, 1-11. |
|  |  | Okkema, P.G. and Krause, M. Transcriptional regulation. WormBook, 1-40. |
|  |  | Boulin, T., Etchberger, J.F. and Hobert, O. Reporter gene fusions. WormBook, 1-23. |
|  |  | Gengyo-Ando, K., Yoshina, S., Inoue, H. and Mitani, S. An efficient transgenic system by TA cloning vectors and RNAi for C. elegans. Biochem Biophys Res Commun 349, 1345-1350. |
|  |  | Edgley, M.L., Baillie, D.L., Riddle, D.L. and Rose, A.M. Genetic balancers. WormBook, 1-32. |
|  |  | Felix, M.A. Oscheius tipulae. WormBook, 1-8. |
|  |  | Von Stetina, S.E., Fox, R.M., Watkins, K.L., Starich, T.A., Shaw, J.E. and Miller, D.M., 3rd UNC-4 represses CEH-12/HB9 to specify synaptic inputs to VA motor neurons in C. elegans. Genes Dev 21, 332-346. |
|  |  | Van Buskirk, C. and Sternberg, P.W. Epidermal growth factor signaling induces behavioral quiescence in Caenorhabditis elegans. Nat Neurosci 10, 1300-1307. |
|  |  | Feinberg, E.H., Vanhoven, M.K., Bendesky, A., Wang, G., Fetter, R.D., Shen, K. and Bargmann, C.I. GFP Reconstitution Across Synaptic Partners (GRASP) defines cell contacts and synapses in living nervous systems. Neuron 57, 353-363. |
|  |  | Jovelin, R. Rapid sequence evolution of transcription factors controlling neuron differentiation in Caenorhabditis. Mol Biol Evol 26, 2373-2386. |
| unc-30 | B0564.10 | Brenner, S. The genetics of Caenorhabditis elegans. Genetics 77, 71-94. |
|  |  | Lewis, J.A., Wu, C.H., Levine, J.H. and Berg, H. Levamisole-resistant mutants of the nematode Caenorhabditis elegans appear to lack pharmacological acetylcholine receptors. Neuroscience 5, 967-989. |
|  |  | Rogalski, T.M., Moerman, D.G. and Baillie, D.L. Essential genes and deficiencies in the unc-22 IV region of Caenorhabditis elegans. Genetics 102, 725-736. |
|  |  | Hodgkin, J. Male Phenotypes and Mating Efficiency in CAENORHABDITIS ELEGANS. Genetics 103, 43-64. |
|  |  | Hedgecock, E.M., Culotti, J.G., Hall, D.H. and Stern, B.D. Genetics of cell and axon migrations in Caenorhabditis elegans. Development 100, 365-382. |
|  |  | Herman, R.K. Mosaic analysis in the nematode Caenorhabditis elegans. J Neurogenet 5, 1-24. |
|  |  | Yuan, J.Y. and Horvitz, H.R. The Caenorhabditis elegans genes ced-3 and ced-4 act cell autonomously to cause programmed cell death. Dev Biol 138, 33-41. |
|  |  | Wolinsky, E. and Way, J. The behavioral genetics of Caenorhabditis elegans. Behav Genet 20, 169-189. |
|  |  | Siddiqui, S.S. Mutations affecting axonal growth and guidance of motor neurons and mechanosensory neurons in the nematode Caenorhabditis elegans. Neurosci Res Suppl 13, S171-190. |
|  |  | Avery, L. The genetics of feeding in Caenorhabditis elegans. Genetics 133, 897-917. |
|  |  | Miller, L.M., Gallegos, M.E., Morisseau, B.A. and Kim, S.K. lin-31, a Caenorhabditis elegans HNF-3/fork head transcription factor homolog, specifies three alternative cell fates in vulval development. Genes Dev 7, 933-947. |
|  |  | McIntire, S.L., Jorgensen, E. and Horvitz, H.R. Genes required for GABA function in Caenorhabditis elegans. Nature 364, 334-337. |
|  |  | Herman, R.K. Neurobiology. Genes make worms behave. Nature 364, 282-283. |
|  |  | Walthall, W.W., Li, L., Plunkett, J.A. and Hsu, C.Y. Changing synaptic specificities in the nervous system of Caenorhabditis elegans: differentiation of the DD motoneurons. J Neurobiol 24, 1589-1599. |
|  |  | Jin, Y., Hoskins, R. and Horvitz, H.R. Control of type-D GABAergic neuron differentiation by C. elegans UNC-30 homeodomain protein. Nature 372, 780-783. |
|  |  | Walthall, W.W. and Plunkett, J.A. Genetic transformation of the synaptic pattern of a motoneuron class in Caenorhabditis elegans. J Neurosci 15, 1035-1043. |
|  |  | Reiner, D.J. and Thomas, J.H. Reversal of a muscle response to GABA during C. elegans male development. J Neurosci 15, 6094-6102. |
|  |  | Shaham, S. and Horvitz, H.R. Developing Caenorhabditis elegans neurons may contain both cell-death protective and killer activities. Genes Dev 10, 578-591. |
|  |  | Wightman, B., Baran, R. and Garriga, G. Genes that guide growth cones along the C. elegans ventral nerve cord. Development 124, 2571-2580. |
|  |  | Chalfie, M. and Jorgensen, E.M. C. elegans neuroscience: genetics to genome. Trends Genet 14, 506-512. |
|  |  | Zhou, H.M. and Walthall, W.W. UNC-55, an orphan nuclear hormone receptor, orchestrates synaptic specificity among two classes of motor neurons in Caenorhabditis elegans. J Neurosci 18, 10438-10444. |
|  |  | Hobert, O., Tessmar, K. and Ruvkun, G. The Caenorhabditis elegans lim-6 LIM homeobox gene regulates neurite outgrowth and function of particular GABAergic neurons. Development 126, 1547-1562. |
|  |  | Eastman, C., Horvitz, H.R. and Jin, Y. Coordinated transcriptional regulation of the unc-25 glutamic acid decarboxylase and the unc-47 GABA vesicular transporter by the Caenorhabditis elegans UNC-30 homeodomain protein. J Neurosci 19, 6225-6234. |
|  |  | Yochem, J., Sundaram, M. and Bucher, E.A. Mosaic analysis in Caenorhabditis elegans. Methods Mol Biol 135, 447-462. |
|  |  | Hekimi, S. Crossroads of aging in the nematode Caenorhabditis elegans. Results Probl Cell Differ 29, 81-112. |
|  |  | Brownlee, D., Holden-Dye, L. and Walker, R. The range and biological activity of FMRFamide-related peptides and classical neurotransmitters in nematodes. Adv Parasitol 45, 109-180. |
|  |  | Branda, C.S. and Stern, M.J. Mechanisms controlling sex myoblast migration in Caenorhabditis elegans hermaphrodites. Dev Biol 226, 137-151. |
|  |  | Sarafi-Reinach, T.R. and Sengupta, P. The forkhead domain gene unc-130 generates chemosensory neuron diversity in C. elegans. Genes Dev 14, 2472-2485. |
|  |  | Hallam, S., Singer, E., Waring, D. and Jin, Y. The C. elegans NeuroD homolog cnd-1 functions in multiple aspects of motor neuron fate specification. Development 127, 4239-4252. |
|  |  | Rand, J.B., Duerr, J.S. and Frisby, D.L. Neurogenetics of vesicular transporters in C. elegans. Faseb J 14, 2414-2422. |
|  |  | Westmoreland, J.J., McEwen, J., Moore, B.A., Jin, Y. and Condie, B.G. Conserved function of Caenorhabditis elegans UNC-30 and mouse Pitx2 in controlling GABAergic neuron differentiation. J Neurosci 21, 6810-6819. |
|  |  | Fares, H. and Greenwald, I. Genetic analysis of endocytosis in Caenorhabditis elegans: coelomocyte uptake defective mutants. Genetics 159, 133-145. |
|  |  | Jorgensen, E.M. and Mango, S.E. The art and design of genetic screens: caenorhabditis elegans. Nat Rev Genet 3, 356-369. |
|  |  | Simmer, F., Tijsterman, M., Parrish, S., Koushika, S.P., Nonet, M.L., Fire, A., Ahringer, J. and Plasterk, R.H. Loss of the putative RNA-directed RNA polymerase RRF-3 makes C. elegans hypersensitive to RNAi. Curr Biol 12, 1317-1319. |
|  |  | Thor, S. and Thomas, J.B. Motor neuron specification in worms, flies and mice: conserved and 'lost' mechanisms. Curr Opin Genet Dev 12, 558-564. |
|  |  | de Bono, M., Tobin, D.M., Davis, M.W., Avery, L. and Bargmann, C.I. Social feeding in Caenorhabditis elegans is induced by neurons that detect aversive stimuli. Nature 419, 899-903. |
|  |  | Hallam, S.J., Goncharov, A., McEwen, J., Baran, R. and Jin, Y. SYD-1, a presynaptic protein with PDZ, C2 and rhoGAP-like domains, specifies axon identity in C. elegans. Nat Neurosci 5, 1137-1146. |
|  |  | Lakso, M., Vartiainen, S., Moilanen, A.M., Sirvio, J., Thomas, J.H., Nass, R., Blakely, R.D. and Wong, G. Dopaminergic neuronal loss and motor deficits in Caenorhabditis elegans overexpressing human alpha-synuclein. J Neurochem 86, 165-172. |
|  |  | Ruvinsky, I. and Ruvkun, G. Functional tests of enhancer conservation between distantly related species. Development 130, 5133-5142. |
|  |  | Hutter, H. Extracellular cues and pioneers act together to guide axons in the ventral cord of C. elegans. Development 130, 5307-5318. |
|  |  | Mehta, N., Loria, P.M. and Hobert, O. A genetic screen for neurite outgrowth mutants in Caenorhabditis elegans reveals a new function for the F-box ubiquitin ligase component LIN-23. Genetics 166, 1253-1267. |
|  |  | Schuske, K., Beg, A.A. and Jorgensen, E.M. The GABA nervous system in C. elegans. Trends Neurosci 27, 407-414. |
|  |  | Cinar, H., Keles, S. and Jin, Y. Expression profiling of GABAergic motor neurons in Caenorhabditis elegans. Curr Biol 15, 340-346. |
|  |  | Melkman, T. and Sengupta, P. Regulation of chemosensory and GABAergic motor neuron development by the C. elegans Aristaless/Arx homolog alr-1. Development 132, 1935-1949. |
|  |  | Shan, G., Kim, K., Li, C. and Walthall, W.W. Convergent genetic programs regulate similarities and differences between related motor neuron classes in Caenorhabditis elegans. Dev Biol 280, 494-503. |
|  |  | Hobert, O. Specification of the nervous system. WormBook, 1-19. |
|  |  | Okkema, P.G. and Krause, M. Transcriptional regulation. WormBook, 1-40. |
|  |  | Hung, W., Hwang, C., Po, M.D. and Zhen, M. Neuronal polarity is regulated by a direct interaction between a scaffolding protein, Neurabin, and a presynaptic SAD-1 kinase in Caenorhabditis elegans. Development 134, 237-249. |
|  |  | Edgley, M.L., Baillie, D.L., Riddle, D.L. and Rose, A.M. Genetic balancers. WormBook, 1-32. |
|  |  | Garcia, S.M., Casanueva, M.O., Silva, M.C., Amaral, M.D. and Morimoto, R.I. Neuronal signaling modulates protein homeostasis in Caenorhabditis elegans post-synaptic muscle cells. Genes Dev 21, 3006-3016. |
|  |  | Axang, C., Rauthan, M., Hall, D.H. and Pilon, M. Developmental genetics of the C. elegans pharyngeal neurons NSML and NSMR. BMC Dev Biol 8, 38. |
|  |  | Ghosh, R. and Emmons, S.W. Episodic swimming behavior in the nematode C. elegans. J Exp Biol 211, 3703-3711. |
|  |  | Ziel, J.W., Hagedorn, E.J., Audhya, A. and Sherwood, D.R. UNC-6 (netrin) orients the invasive membrane of the anchor cell in C. elegans. Nat Cell Biol 11, 183-189. |
|  |  | Jovelin, R. Rapid sequence evolution of transcription factors controlling neuron differentiation in Caenorhabditis. Mol Biol Evol 26, 2373-2386. |
|  |  | Locke, C.J., Kautu, B.B., Berry, K.P., Lee, S.K., Caldwell, K.A. and Caldwell, G.A. Pharmacogenetic analysis reveals a post-developmental role for Rac GTPases in Caenorhabditis elegans GABAergic neurotransmission. Genetics 183, 1357-1372. |
|  |  | Wang, J., Chen, P.J., Wang, G.J. and Keller, L. Chromosome size differences may affect meiosis and genome size. Science 329, 293. |
| unc-39 | F56A12.1 | Brenner, S. The genetics of Caenorhabditis elegans. Genetics 77, 71-94. |
|  |  | Hodgkin, J. Male Phenotypes and Mating Efficiency in CAENORHABDITIS ELEGANS. Genetics 103, 43-64. |
|  |  | Hedgecock, E.M., Culotti, J.G., Hall, D.H. and Stern, B.D. Genetics of cell and axon migrations in Caenorhabditis elegans. Development 100, 365-382. |
|  |  | Manser, J. and Wood, W.B. Mutations affecting embryonic cell migrations in Caenorhabditis elegans. Dev Genet 11, 49-64. |
|  |  | Aamodt, E.J., Chung, M.A. and McGhee, J.D. Spatial control of gut-specific gene expression during Caenorhabditis elegans development. Science 252, 579-582. |
|  |  | Wadsworth, W.G. and Hedgecock, E.M. Guidance of neuroblast migrations and axonal projections in Caenorhabditis elegans. Curr Opin Neurobiol 2, 36-41. |
|  |  | Dozier, C., Kagoshima, H., Niklaus, G., Cassata, G. and Bürglin, T.R. The Caenorhabditis elegans Six/sine oculis class homeobox gene ceh-32 is required for head morphogenesis. Dev Biol 236, 289-303. |
|  |  | Fares, H. and Greenwald, I. Genetic analysis of endocytosis in Caenorhabditis elegans: coelomocyte uptake defective mutants. Genetics 159, 133-145. |
|  |  | Yanowitz, J.L., Shakir, M.A., Hedgecock, E., Hutter, H., Fire, A.Z. and Lundquist, E.A. UNC-39, the C. elegans homolog of the human myotonic dystrophy-associated homeodomain protein Six5, regulates cell motility and differentiation. Dev Biol 272, 389-402. |
|  |  | Herman, M.A. Hermaphrodite cell-fate specification. WormBook, 1-16. |
|  |  | Okkema, P.G. and Krause, M. Transcriptional regulation. WormBook, 1-40. |
|  |  | Mango, S.E. The C. elegans pharynx: a model for organogenesis. WormBook, 1-26. |
|  |  | Watari-Goshima, N., Ogura, K., Wolf, F.W., Goshima, Y. and Garriga, G. C. elegans VAB-8 and UNC-73 regulate the SAX-3 receptor to direct cell and growth-cone migrations. Nat Neurosci 10, 169-176. |
|  |  | Reece-Hoyes, J.S., Shingles, J., Dupuy, D., Grove, C.A., Walhout, A.J., Vidal, M. and Hope, I.A. Insight into transcription factor gene duplication from Caenorhabditis elegans Promoterome-driven expression patterns. BMC Genomics 8, 27. |
|  |  | Ziel, J.W., Hagedorn, E.J., Audhya, A. and Sherwood, D.R. UNC-6 (netrin) orients the invasive membrane of the anchor cell in C. elegans. Nat Cell Biol 11, 183-189. |
|  |  | Amin, N.M., Lim, S.E., Shi, H., Chan, T.L. and Liu, J. A conserved Six-Eya cassette acts downstream of Wnt signaling to direct non-myogenic versus myogenic fates in the C. elegans postembryonic mesoderm. Dev Biol 331, 350-360. |
| unc-42 | F58E6.10 | Brenner, S. The genetics of Caenorhabditis elegans. Genetics 77, 71-94. |
|  |  | Herman, R.K. Crossover suppressors and balanced recessive lethals in Caenorhabditis elegans. Genetics 88, 49-65. |
|  |  | Riddle, D.L. and Brenner, S. Indirect suppression in Caenorhabditis elegans. Genetics 89, 299-314. |
|  |  | Rose, A.M. and Baillie, D.L. A mutation in Caenorhabditis elegans that increases recombination frequency more than threefold. Nature 281, 599-600. |
|  |  | Waterston, R.H., Thomson, J.N. and Brenner, S. Mutants with altered muscle structure of Caenorhabditis elegans. Dev Biol 77, 271-302. |
|  |  | Rosenbluth, R.E. and Baillie, D.L. The genetic analysis of a reciprocal translocation, eT1(III; V), in Caenorhabditis elegans. Genetics 99, 415-428. |
|  |  | Hodgkin, J. Male Phenotypes and Mating Efficiency in CAENORHABDITIS ELEGANS. Genetics 103, 43-64. |
|  |  | Rosenbluth, R.E., Cuddeford, C. and Baillie, D.L. Mutagenesis in Caenorhabditis elegans. II. A spectrum of mutational events induced with 1500 r of gamma-radiation. Genetics 109, 493-511. |
|  |  | Stewart, H.I., Rosenbluth, R.E. and Baillie, D.L. Most ultraviolet irradiation induced mutations in the nematode Caenorhabditis elegans are chromosomal rearrangements. Mutat Res 249, 37-54. |
|  |  | Zetka, M.C. and Rose, A.M. The meiotic behavior of an inversion in Caenorhabditis elegans. Genetics 131, 321-332. |
|  |  | Perry, M.D., Li, W., Trent, C., Robertson, B., Fire, A., Hageman, J.M. and Wood, W.B. Molecular characterization of the her-1 gene suggests a direct role in cell signaling during Caenorhabditis elegans sex determination. Genes Dev 7, 216-228. |
|  |  | Seydoux, G., Savage, C. and Greenwald, I. Isolation and characterization of mutations causing abnormal eversion of the vulva in Caenorhabditis elegans. Dev Biol 157, 423-436. |
|  |  | McKim, K.S., Peters, K. and Rose, A.M. Two types of sites required for meiotic chromosome pairing in Caenorhabditis elegans. Genetics 134, 749-768. |
|  |  | Thomas, J.H., Birnby, D.A. and Vowels, J.J. Evidence for parallel processing of sensory information controlling dauer formation in Caenorhabditis elegans. Genetics 134, 1105-1117. |
|  |  | Macrae, M., Plasterk, R.H. and Coffino, P. The ornithine decarboxylase gene of Caenorhabditis elegans: cloning, mapping and mutagenesis. Genetics 140, 517-525. |
|  |  | Wightman, B., Baran, R. and Garriga, G. Genes that guide growth cones along the C. elegans ventral nerve cord. Development 124, 2571-2580. |
|  |  | Baran, R., Aronoff, R. and Garriga, G. The C. elegans homeodomain gene unc-42 regulates chemosensory and glutamate receptor expression. Development 126, 2241-2251. |
|  |  | Much, J.W., Slade, D.J., Klampert, K., Garriga, G. and Wightman, B. The fax-1 nuclear hormone receptor regulates axon pathfinding and neurotransmitter expression. Development 127, 703-712. |
|  |  | Pujol, N., Torregrossa, P., Ewbank, J.J. and Brunet, J.F. The homeodomain protein CePHOX2/CEH-17 controls antero-posterior axonal growth in C. elegans. Development 127, 3361-3371. |
|  |  | Brockie, P.J., Madsen, D.M., Zheng, Y., Mellem, J. and Maricq, A.V. Differential expression of glutamate receptor subunits in the nervous system of Caenorhabditis elegans and their regulation by the homeodomain protein UNC-42. J Neurosci 21, 1510-1522. |
|  |  | Fares, H. and Greenwald, I. Genetic analysis of endocytosis in Caenorhabditis elegans: coelomocyte uptake defective mutants. Genetics 159, 133-145. |
|  |  | Sarafi-Reinach, T.R., Melkman, T., Hobert, O. and Sengupta, P. The lin-11 LIM homeobox gene specifies olfactory and chemosensory neuron fates in C. elegans. Development 128, 3269-3281. |
|  |  | Bany, I.A., Dong, M.Q. and Koelle, M.R. Genetic and cellular basis for acetylcholine inhibition of Caenorhabditis elegans egg-laying behavior. J Neurosci 23, 8060-8069. |
|  |  | Chang, S., Johnston, R.J., Jr. and Hobert, O. A transcriptional regulatory cascade that controls left/right asymmetry in chemosensory neurons of C. elegans. Genes Dev 17, 2123-2137. |
|  |  | Ailion, M. and Thomas, J.H. Isolation and characterization of high-temperature-induced Dauer formation mutants in Caenorhabditis elegans. Genetics 165, 127-144. |
|  |  | Melkman, T. and Sengupta, P. The worm's sense of smell. Development of functional diversity in the chemosensory system of Caenorhabditis elegans. Dev Biol 265, 302-319. |
|  |  | Lanjuin, A. and Sengupta, P. Specification of chemosensory neuron subtype identities in Caenorhabditis elegans. Curr Opin Neurobiol 14, 22-30. |
|  |  | Gravato-Nobre, M.J., Nicholas, H.R., Nijland, R., O'Rourke, D., Whittington, D.E., Yook, K.J. and Hodgkin, J. Multiple genes affect sensitivity of Caenorhabditis elegans to the bacterial pathogen Microbacterium nematophilum. Genetics 171, 1033-1045. |
|  |  | Wightman, B., Ebert, B., Carmean, N., Weber, K. and Clever, S. The C. elegans nuclear receptor gene fax-1 and homeobox gene unc-42 coordinate interneuron identity by regulating the expression of glutamate receptor subunits and other neuron-specific genes. Dev Biol 287, 74-85. |
|  |  | Antebi, A. Nuclear hormone receptors in C. elegans. WormBook, 1-13. |
|  |  | Hobert, O. Specification of the nervous system. WormBook, 1-19. |
|  |  | Okkema, P.G. and Krause, M. Transcriptional regulation. WormBook, 1-40. |
|  |  | Edgley, M.L., Baillie, D.L., Riddle, D.L. and Rose, A.M. Genetic balancers. WormBook, 1-32. |
|  |  | Jovelin, R. Rapid sequence evolution of transcription factors controlling neuron differentiation in Caenorhabditis. Mol Biol Evol 26, 2373-2386. |
| unc-62 | T28F12.2 | Brenner, S. The genetics of Caenorhabditis elegans. Genetics 77, 71-94. |
|  |  | Hodgkin, J. Male Phenotypes and Mating Efficiency in CAENORHABDITIS ELEGANS. Genetics 103, 43-64. |
|  |  | Rosenbluth, R.E., Cuddeford, C. and Baillie, D.L. Mutagenesis in Caenorhabditis elegans. II. A spectrum of mutational events induced with 1500 r of gamma-radiation. Genetics 109, 493-511. |
|  |  | Hedgecock, E.M., Culotti, J.G., Hall, D.H. and Stern, B.D. Genetics of cell and axon migrations in Caenorhabditis elegans. Development 100, 365-382. |
|  |  | Wolinsky, E. and Way, J. The behavioral genetics of Caenorhabditis elegans. Behav Genet 20, 169-189. |
|  |  | Siddiqui, S.S. Mutations affecting axonal growth and guidance of motor neurons and mechanosensory neurons in the nematode Caenorhabditis elegans. Neurosci Res Suppl 13, S171-190. |
|  |  | Johnsen, R.C. and Baillie, D.L. Genetic analysis of a major segment [LGV(left)] of the genome of Caenorhabditis elegans. Genetics 129, 735-752. |
|  |  | McKim, K.S., Matheson, C., Marra, M.A., Wakarchuk, M.F. and Baillie, D.L. The Caenorhabditis elegans unc-60 gene encodes proteins homologous to a family of actin-binding proteins. Mol Gen Genet 242, 346-357. |
|  |  | Bürglin, T.R. Analysis of TALE superclass homeobox genes (MEIS, PBC, KNOX, Iroquois, TGIF) reveals a novel domain conserved between plants and animals. Nucleic Acids Res 25, 4173-4180. |
|  |  | Chamberlin, H.M. and Thomas, J.H. The bromodomain protein LIN-49 and trithorax-related protein LIN-59 affect development and gene expression in Caenorhabditis elegans. Development 127, 713-723. |
|  |  | Branda, C.S. and Stern, M.J. Mechanisms controlling sex myoblast migration in Caenorhabditis elegans hermaphrodites. Dev Biol 226, 137-151. |
|  |  | Van Auken, K., Weaver, D., Robertson, B., Sundaram, M., Saldi, T., Edgar, L., Elling, U., Lee, M., Boese, Q. and Wood, W.B. Roles of the Homothorax/Meis/Prep homolog UNC-62 and the Exd/Pbx homologs CEH-20 and CEH-40 in C. elegans embryogenesis. Development 129, 5255-5268. |
|  |  | Morck, C., Axang, C. and Pilon, M. A genetic analysis of axon guidance in the C elegans pharynx. Dev Biol 260, 158-175. |
|  |  | Chang, S., Johnston, R.J., Jr. and Hobert, O. A transcriptional regulatory cascade that controls left/right asymmetry in chemosensory neurons of C. elegans. Genes Dev 17, 2123-2137. |
|  |  | Ryder, S.P., Frater, L.A., Abramovitz, D.L., Goodwin, E.B. and Williamson, J.R. RNA target specificity of the STAR/GSG domain post-transcriptional regulatory protein GLD-1. Nat Struct Mol Biol 11, 20-28. |
|  |  | Nelson, C.E., Hersh, B.M. and Carroll, S.B. The regulatory content of intergenic DNA shapes genome architecture. Genome Biol 5, R25. |
|  |  | Yang, L., Sym, M. and Kenyon, C. The roles of two C. elegans HOX co-factor orthologs in cell migration and vulva development. Development 132, 1413-1428. |
|  |  | Baugh, L.R., Wen, J.C., Hill, A.A., Slonim, D.K., Brown, E.L. and Hunter, C.P. Synthetic lethal analysis of Caenorhabditis elegans posterior embryonic patterning genes identifies conserved genetic interactions. Genome Biol 6, R45. |
|  |  | Chisholm, A.D. and Hardin, J. Epidermal morphogenesis. WormBook, 1-22. |
|  |  | Okkema, P.G. and Krause, M. Transcriptional regulation. WormBook, 1-40. |
|  |  | Sternberg, P.W. Vulval development. WormBook, 1-28. |
|  |  | McGhee, J.D., Sleumer, M.C., Bilenky, M., Wong, K., McKay, S.J., Goszczynski, B., Tian, H., Krich, N.D., Khattra, J., Holt, R.A. et al. The ELT-2 GATA-factor and the global regulation of transcription in the C. elegans intestine. Dev Biol 302, 627-645. |
|  |  | Curran, S.P. and Ruvkun, G. Lifespan regulation by evolutionarily conserved genes essential for viability. PLoS Genet 3, e56. |
|  |  | Mukherjee, K. and Bürglin, T.R. Comprehensive analysis of animal TALE homeobox genes: new conserved motifs and cases of accelerated evolution. J Mol Evol 65, 137-153. |
|  |  | Potts, M.B., Wang, D.P. and Cameron, S. Trithorax, Hox, and TALE-class homeodomain proteins ensure cell survival through repression of the BH3-only gene egl-1. Dev Biol 329, 374-385. |
|  |  | Jiang, Y., Shi, H. and Liu, J. Two Hox cofactors, the Meis/Hth homolog UNC-62 and the Pbx/Exd homolog CEH-20, function together during C. elegans postembryonic mesodermal development. Dev Biol 334, 535-546. |
|  |  | Zhang, Y. and Maduzia, L.L. Mutations in Caenorhabditis elegans eIF2beta permit translation initiation from non-AUG start codons. Genetics 185, 141-152. |
| unc-86 | C30A5.7 | Hodgkin, J., Horvitz, H.R. and Brenner, S. Nondisjunction Mutants of the Nematode CAENORHABDITIS ELEGANS. Genetics 91, 67-94. |
|  |  | Horvitz, H.R. and Sulston, J.E. Isolation and genetic characterization of cell-lineage mutants of the nematode Caenorhabditis elegans. Genetics 96, 435-454. |
|  |  | Sulston, J.E. and Horvitz, H.R. Abnormal cell lineages in mutants of the nematode Caenorhabditis elegans. Dev Biol 82, 41-55. |
|  |  | Chalfie, M. and Sulston, J. Developmental genetics of the mechanosensory neurons of Caenorhabditis elegans. Dev Biol 82, 358-370. |
|  |  | Chalfie, M., Horvitz, H.R. and Sulston, J.E. Mutations that lead to reiterations in the cell lineages of C. elegans. Cell 24, 59-69. |
|  |  | Horvitz, H.R., Chalfie, M., Trent, C., Sulston, J.E. and Evans, P.D. Serotonin and octopamine in the nematode Caenorhabditis elegans. Science 216, 1012-1014. |
|  |  | Chalfie, M. Microtubule structure in Caenorhabditis elegans neurons. Cold Spring Harb Symp Quant Biol 46 Pt 1, 255-261. |
|  |  | Hartman, P.S. and Herman, R.K. Radiation-sensitive mutants of Caenorhabditis elegans. Genetics 102, 159-178. |
|  |  | Hodgkin, J. Male Phenotypes and Mating Efficiency in CAENORHABDITIS ELEGANS. Genetics 103, 43-64. |
|  |  | Trent, C., Tsuing, N. and Horvitz, H.R. Egg-laying defective mutants of the nematode Caenorhabditis elegans. Genetics 104, 619-647. |
|  |  | Sulston, J.E. Neuronal cell lineages in the nematode Caenorhabditis elegans. Cold Spring Harb Symp Quant Biol 48 Pt 2, 443-452. |
|  |  | Horvitz, H.R., Sternberg, P.W., Greenwald, I.S., Fixsen, W. and Ellis, H.M. Mutations that affect neural cell lineages and cell fates during the development of the nematode Caenorhabditis elegans. Cold Spring Harb Symp Quant Biol 48 Pt 2, 453-463. |
|  |  | Walthall, W.W. and Chalfie, M. Cell-cell interactions in the guidance of late-developing neurons in Caenorhabditis elegans. Science 239, 643-645. |
|  |  | Kenyon, C. The nematode Caenorhabditis elegans. Science 240, 1448-1453. |
|  |  | Desai, C., Garriga, G., McIntire, S.L. and Horvitz, H.R. A genetic pathway for the development of the Caenorhabditis elegans HSN motor neurons. Nature 336, 638-646. |
|  |  | Finney, M., Ruvkun, G. and Horvitz, H.R. The C. elegans cell lineage and differentiation gene unc-86 encodes a protein with a homeodomain and extended similarity to transcription factors. Cell 55, 757-769. |
|  |  | Herr, W., Sturm, R.A., Clerc, R.G., Corcoran, L.M., Baltimore, D., Sharp, P.A., Ingraham, H.A., Rosenfeld, M.G., Finney, M., Ruvkun, G. et al. The POU domain: a large conserved region in the mammalian pit-1, oct-1, oct-2, and Caenorhabditis elegans unc-86 gene products. Genes Dev 2, 1513-1516. |
|  |  | Desai, C. and Horvitz, H.R. Caenorhabditis elegans mutants defective in the functioning of the motor neurons responsible for egg laying. Genetics 121, 703-721. |
|  |  | Bürglin, T.R., Finney, M., Coulson, A. and Ruvkun, G. Caenorhabditis elegans has scores of homoeobox-containing genes. Nature 341, 239-243. |
|  |  | Way, J.C. and Chalfie, M. The mec-3 gene of Caenorhabditis elegans requires its own product for maintained expression and is expressed in three neuronal cell types. Genes Dev 3, 1823-1833. |
|  |  | Sternberg, P.W. Genetic control of cell type and pattern formation in Caenorhabditis elegans. Adv Genet 27, 63-116. |
|  |  | Wolinsky, E. and Way, J. The behavioral genetics of Caenorhabditis elegans. Behav Genet 20, 169-189. |
|  |  | Chiba, C.M. and Rankin, C.H. A developmental analysis of spontaneous and reflexive reversals in the nematode Caenorhabditis elegans. J Neurobiol 21, 543-554. |
|  |  | Horvitz, H.R. Genetic control of Caenorhabditis elegans cell lineage. Harvey Lect 84, 65-77. |
|  |  | Finney, M. and Ruvkun, G. The unc-86 gene product couples cell lineage and cell identity in C. elegans. Cell 63, 895-905. |
|  |  | Yuan, J.Y., Finney, M., Tsung, N. and Horvitz, H.R. Tc4, a Caenorhabditis elegans transposable element with an unusual fold-back structure. Proc Natl Acad Sci U S A 88, 3334-3338. |
|  |  | Siddiqui, S.S. and Culotti, J.G. Examination of neurons in wild type and mutants of Caenorhabditis elegans using antibodies to horseradish peroxidase. J Neurogenet 21, 271-289. |
|  |  | Hodgkin, J. The interactive worm. Eighth international C. elegans Meeting, Madison, WI, USA, June 1-5, 1991. New Biol 3, 951-954. |
|  |  | Way, J.C., Wang, L., Run, J.Q. and Wang, A. The mec-3 gene contains cis-acting elements mediating positive and negative regulation in cells produced by asymmetric cell division in Caenorhabditis elegans. Genes Dev 5, 2199-2211. |
|  |  | Wadsworth, W.G. and Hedgecock, E.M. Guidance of neuroblast migrations and axonal projections in Caenorhabditis elegans. Curr Opin Neurobiol 2, 36-41. |
|  |  | Hamelin, M., Scott, I.M., Way, J.C. and Culotti, J.G. The mec-7 beta-tubulin gene of Caenorhabditis elegans is expressed primarily in the touch receptor neurons. Embo J 11, 2885-2893. |
|  |  | Bird, D.M. Sequence comparison of the Caenorhabditis elegans dpy-13 and col-34 genes, and their deduced collagen products. Gene 120, 261-266. |
|  |  | Li, W., Herman, R.K. and Shaw, J.E. Analysis of the Caenorhabditis elegans axonal guidance and outgrowth gene unc-33. Genetics 132, 675-689. |
|  |  | Xue, D., Finney, M., Ruvkun, G. and Chalfie, M. Regulation of the mec-3 gene by the C.elegans homeoproteins UNC-86 and MEC-3. Embo J 11, 4969-4979. |
|  |  | Way, J.C., Run, J.Q. and Wang, A.Y. Regulation of anterior cell-specific mec-3 expression during asymmetric cell division in C. elegans. Dev Dyn 194, 289-302. |
|  |  | Bargmann, C.I. Genetic and cellular analysis of behavior in C. elegans. Annu Rev Neurosci 16, 47-71. |
|  |  | Bargmann, C.I. Death from natural and unnatural causes. Curr Biol 1, 388-390. |
|  |  | Chalfie, M. Homeobox genes in Caenorhabditis elegans. Curr Opin Genet Dev 3, 275-277. |
|  |  | L'Hernault, S.W., Benian, G.M. and Emmons, R.B. Genetic and molecular characterization of the Caenorhabditis elegans spermatogenesis-defective gene spe-17. Genetics 134, 769-780. |
|  |  | Xue, D., Tu, Y. and Chalfie, M. Cooperative interactions between the Caenorhabditis elegans homeoproteins UNC-86 and MEC-3. Science 261, 1324-1328. |
|  |  | Chalfie, M. Touch receptor development and function in Caenorhabditis elegans. J Neurobiol 24, 1433-1441. |
|  |  | Bürglin, T.R. and Ruvkun, G. The Caenorhabditis elegans homeobox gene cluster. Curr Opin Genet Dev 3, 615-620. |
|  |  | Mitani, S., Du, H., Hall, D.H., Driscoll, M. and Chalfie, M. Combinatorial control of touch receptor neuron expression in Caenorhabditis elegans. Development 119, 773-783. |
|  |  | Wilson, R., Ainscough, R., Anderson, K., Baynes, C., Berks, M., Bonfield, J., Burton, J., Connell, M., Copsey, T., Cooper, J. et al. 2.2 Mb of contiguous nucleotide sequence from chromosome III of C. elegans. Nature 368, 32-38. |
|  |  | Collins, J.J. and Anderson, P. The Tc5 family of transposable elements in Caenorhabditis elegans. Genetics 137, 771-781. |
|  |  | Stenico, M., Lloyd, A.T. and Sharp, P.M. Codon usage in Caenorhabditis elegans: delineation of translational selection and mutational biases. Nucleic Acids Res 22, 2437-2446. |
|  |  | Sommer, R.J., Carta, L.K. and Sternberg, P.W. The evolution of cell lineage in nematodes. Dev Suppl, 85-95. |
|  |  | Duggan, A. and Chalfie, M. Control of neuronal development in Caenorhabditis elegans. Curr Opin Neurobiol 5, 6-9. |
|  |  | Mori, I. and Ohshima, Y. Neural regulation of thermotaxis in Caenorhabditis elegans. Nature 376, 344-348. |
|  |  | Lichtsteiner, S. and Tjian, R. Synergistic activation of transcription by UNC-86 and MEC-3 in Caenorhabditis elegans embryo extracts. Embo J 14, 3937-3945. |
|  |  | Huang, M., Gu, G., Ferguson, E.L. and Chalfie, M. A stomatin-like protein necessary for mechanosensation in C. elegans. Nature 378, 292-295. |
|  |  | Jia, Y., Xie, G. and Aamodt, E. pag-3, a Caenorhabditis elegans gene involved in touch neuron gene expression and coordinated movement. Genetics 142, 141-147. |
|  |  | Chalfie, M. The differentiation and function of the touch receptor neurons of Caenorhabditis elegans. Prog Brain Res 105, 179-182. |
|  |  | Herman, R.K. Touch sensation in Caenorhabditis elegans. Bioessays 18, 199-206. |
|  |  | Miura, M. and Yuan, J. Mechanisms of programmed cell death in Caenorhabditis elegans and vertebrates. Curr Top Dev Biol 32, 139-174. |
|  |  | Baumeister, R., Liu, Y. and Ruvkun, G. Lineage-specific regulators couple cell lineage asymmetry to the transcription of the Caenorhabditis elegans POU gene unc-86 during neurogenesis. Genes Dev 10, 1395-1410. |
|  |  | Wang, L. and Way, J.C. Activation of the mec-3 promoter in two classes of stereotyped lineages in Caenorhabditis elegans. Mech Dev 56, 165-181. |
|  |  | Wang, L. and Way, J.C. Promoter sequences for the establishment of mec-3 expression in the nematode Caenorhabditis elegans. Mech Dev 56, 183-196. |
|  |  | Guenther, C. and Garriga, G. Asymmetric distribution of the C. elegans HAM-1 protein in neuroblasts enables daughter cells to adopt distinct fates. Development 122, 3509-3518. |
|  |  | Garcia-Anoveros, J. and Corey, D.P. The molecules of mechanosensation. Annu Rev Neurosci 20, 567-594. |
|  |  | Tavernarakis, N. and Driscoll, M. Molecular modeling of mechanotransduction in the nematode Caenorhabditis elegans. Annu Rev Physiol 59, 659-689. |
|  |  | Sze, J.Y., Liu, Y. and Ruvkun, G. VP16-activation of the C. elegans neural specification transcription factor UNC-86 suppresses mutations in downstream genes and causes defects in neural migration and axon outgrowth. Development 124, 1159-1168. |
|  |  | Hobert, O., Mori, I., Yamashita, Y., Honda, H., Ohshima, Y., Liu, Y. and Ruvkun, G. Regulation of interneuron function in the C. elegans thermoregulatory pathway by the ttx-3 LIM homeobox gene. Neuron 19, 345-357. |
|  |  | Shibatohge, M., Kariya, K., Liao, Y., Hu, C.D., Watari, Y., Goshima, M., Shima, F. and Kataoka, T. Identification of PLC210, a Caenorhabditis elegans phospholipase C, as a putative effector of Ras. J Biol Chem 273, 6218-6222. |
|  |  | Duggan, A., Ma, C. and Chalfie, M. Regulation of touch receptor differentiation by the Caenorhabditis elegans mec-3 and unc-86 genes. Development 125, 4107-4119. |
|  |  | Chalfie, M. and Jorgensen, E.M. C. elegans neuroscience: genetics to genome. Trends Genet 14, 506-512. |
|  |  | Hawkins, N. and Garriga, G. Asymmetric cell division: from A to Z. Genes Dev 12, 3625-3638. |
|  |  | Baum, P.D., Guenther, C., Frank, C.A., Pham, B.V. and Garriga, G. The Caenorhabditis elegans gene ham-2 links Hox patterning to migration of the HSN motor neuron. Genes Dev 13, 472-483. |
|  |  | Troemel, E.R., Sagasti, A. and Bargmann, C.I. Lateral signaling mediated by axon contact and calcium entry regulates asymmetric odorant receptor expression in C. elegans. Cell 99, 387-398. |
|  |  | Mori, I. Genetics of chemotaxis and thermotaxis in the nematode Caenorhabditis elegans. Annu Rev Genet 33, 399-422. |
|  |  | Rockelein, I., Rohrig, S., Donhauser, R., Eimer, S. and Baumeister, R. Identification of amino acid residues in the Caenorhabditis elegans POU protein UNC-86 that mediate UNC-86-MEC-3-DNA ternary complex formation. Mol Cell Biol 20, 4806-4813. |
|  |  | Rohrig, S., Rockelein, I., Donhauser, R. and Baumeister, R. Protein interaction surface of the POU transcription factor UNC-86 selectively used in touch neurons. Embo J 19, 3694-3703. |
|  |  | Bürglin, T.R. and Ruvkun, G. Regulation of ectodermal and excretory function by the C. elegans POU homeobox gene ceh-6. Development 128, 779-790. |
|  |  | Sarafi-Reinach, T.R., Melkman, T., Hobert, O. and Sengupta, P. The lin-11 LIM homeobox gene specifies olfactory and chemosensory neuron fates in C. elegans. Development 128, 3269-3281. |
|  |  | Shaham, S. and Bargmann, C.I. Control of neuronal subtype identity by the C. elegans ARID protein CFI-1. Genes Dev 16, 972-983. |
|  |  | Sze, J.Y., Zhang, S., Li, J. and Ruvkun, G. The C. elegans POU-domain transcription factor UNC-86 regulates the tph-1 tryptophan hydroxylase gene and neurite outgrowth in specific serotonergic neurons. Development 129, 3901-3911. |
|  |  | Simmer, F., Tijsterman, M., Parrish, S., Koushika, S.P., Nonet, M.L., Fire, A., Ahringer, J. and Plasterk, R.H. Loss of the putative RNA-directed RNA polymerase RRF-3 makes C. elegans hypersensitive to RNAi. Curr Biol 12, 1317-1319. |
|  |  | Reinke, V. Functional exploration of the C. elegans genome using DNA microarrays. Nat Genet 32 Suppl, 541-546. |
|  |  | Zariwala, H.A., Miller, A.C., Faumont, S. and Lockery, S.R. Step response analysis of thermotaxis in Caenorhabditis elegans. J Neurosci 23, 4369-4377. |
|  |  | Sze, J.Y. and Ruvkun, G. Activity of the Caenorhabditis elegans UNC-86 POU transcription factor modulates olfactory sensitivity. Proc Natl Acad Sci U S A 100, 9560-9565. |
|  |  | Ailion, M. and Thomas, J.H. Isolation and characterization of high-temperature-induced Dauer formation mutants in Caenorhabditis elegans. Genetics 165, 127-144. |
|  |  | Toker, A.S., Teng, Y., Ferreira, H.B., Emmons, S.W. and Chalfie, M. The Caenorhabditis elegans spalt-like gene sem-4 restricts touch cell fate by repressing the selector Hox gene egl-5 and the effector gene mec-3. Development 130, 3831-3840. |
|  |  | Clark, S.G. and Chiu, C. C. elegans ZAG-1, a Zn-finger-homeodomain protein, regulates axonal development and neuronal differentiation. Development 130, 3781-3794. |
|  |  | Hwang, S.B. and Lee, J. Neuron cell type-specific SNAP-25 expression driven by multiple regulatory elements in the nematode Caenorhabditis elegans. J Mol Biol 333, 237-247. |
|  |  | Frank, C.A., Baum, P.D. and Garriga, G. HLH-14 is a C. elegans achaete-scute protein that promotes neurogenesis through asymmetric cell division. Development 130, 6507-6518. |
|  |  | Qin, H. and Powell-Coffman, J.A. The Caenorhabditis elegans aryl hydrocarbon receptor, AHR-1, regulates neuronal development. Dev Biol 270, 64-75. |
|  |  | Kim, K. and Li, C. Expression and regulation of an FMRFamide-related neuropeptide gene family in Caenorhabditis elegans. J Comp Neurol 475, 540-550. |
|  |  | Nelson, C.E., Hersh, B.M. and Carroll, S.B. The regulatory content of intergenic DNA shapes genome architecture. Genome Biol 5, R25. |
|  |  | Kage, E., Hayashi, Y., Takeuchi, H., Hirotsu, T., Kunitomo, H., Inoue, T., Arai, H., Iino, Y. and Kubo, T. MBR-1, a novel helix-turn-helix transcription factor, is required for pruning excessive neurites in Caenorhabditis elegans. Curr Biol 15, 1554-1559. |
|  |  | Wadsworth, W.G. Axon pruning: C. elegans makes the cut. Curr Biol 15, R796-798. |
|  |  | Priess, J.R. Notch signaling in the C. elegans embryo. WormBook, 1-16. |
|  |  | Conradt, B. and Xue, D. Programmed cell death. WormBook, 1-13. |
|  |  | Hobert, O. Specification of the nervous system. WormBook, 1-19. |
|  |  | Jin, Y. Synaptogenesis. WormBook, 1-11. |
|  |  | Okkema, P.G. and Krause, M. Transcriptional regulation. WormBook, 1-40. |
|  |  | Bessereau, J.L. Transposons in C. elegans. WormBook, 1-13. |
|  |  | Liang, B., Moussaif, M., Kuan, C.J., Gargus, J.J. and Sze, J.Y. Serotonin targets the DAF-16/FOXO signaling pathway to modulate stress responses. Cell Metab 4, 429-440. |
|  |  | Edgley, M.L., Baillie, D.L., Riddle, D.L. and Rose, A.M. Genetic balancers. WormBook, 1-32. |
|  |  | Kobayashi, T., Gengyo-Ando, K., Ishihara, T., Katsura, I. and Mitani, S. IFT-81 and IFT-74 are required for intraflagellar transport in C. elegans. Genes Cells 12, 593-602. |
|  |  | Van Buskirk, C. and Sternberg, P.W. Epidermal growth factor signaling induces behavioral quiescence in Caenorhabditis elegans. Nat Neurosci 10, 1300-1307. |
|  |  | Peden, E., Kimberly, E., Gengyo-Ando, K., Mitani, S. and Xue, D. Control of sex-specific apoptosis in C. elegans by the BarH homeodomain protein CEH-30 and the transcriptional repressor UNC-37/Groucho. Genes Dev 21, 3195-3207. |
|  |  | Yanowitz, J.L. Genome integrity is regulated by the Caenorhabditis elegans Rad51D homolog rfs-1. Genetics 179, 249-262. |
|  |  | Luo, J., Shah, S., Riabowol, K. and Mains, P.E. The Caenorhabditis elegans ing-3 gene regulates ionizing radiation-induced germ-cell apoptosis in a p53-associated pathway. Genetics 181, 473-482. |
|  |  | Donohoe, D.R., Jarvis, R.A., Weeks, K., Aamodt, E.J. and Dwyer, D.S. Behavioral adaptation in C. elegans produced by antipsychotic drugs requires serotonin and is associated with calcium signaling and calcineurin inhibition. Neurosci Res 64, 280-289. |
|  |  | Jovelin, R. Rapid sequence evolution of transcription factors controlling neuron differentiation in Caenorhabditis. Mol Biol Evol 26, 2373-2386. |
|  |  | Tong, Y.G. and Bürglin, T.R. Conditions for dye-filling of sensory neurons in Caenorhabditis elegans. J Neurosci Methods 188, 58-61. |
|  |  | Nehme, R., Grote, P., Tomasi, T., Loser, S., Holzkamp, H., Schnabel, R. and Conradt, B. Transcriptional upregulation of both egl-1 BH3-only and ced-3 caspase is required for the death of the male-specific CEM neurons. Cell Death Differ 17, 1266-1276. |
| vab-3 | F14F3.1 | Lewis, J.A. and Hodgkin, J.A. Specific neuroanatomical changes in chemosensory mutants of the nematode Caenorhabditis elegans. J Comp Neurol 172, 489-510. |
|  |  | Hodgkin, J. Male Phenotypes and Mating Efficiency in CAENORHABDITIS ELEGANS. Genetics 103, 43-64. |
|  |  | Hedgecock, E.M., Culotti, J.G., Hall, D.H. and Stern, B.D. Genetics of cell and axon migrations in Caenorhabditis elegans. Development 100, 365-382. |
|  |  | Link, C.D., Ehrenfels, C.W. and Wood, W.B. Mutant expression of male copulatory bursa surface markers in Caenorhabditis elegans. Development 103, 485-495. |
|  |  | Baird, S.E., Fitch, D.H., Kassem, I.A. and Emmons, S.W. Pattern formation in the nematode epidermis: determination of the arrangement of peripheral sense organs in the C. elegans male tail. Development 113, 515-526. |
|  |  | Mitani, S., Du, H., Hall, D.H., Driscoll, M. and Chalfie, M. Combinatorial control of touch receptor neuron expression in Caenorhabditis elegans. Development 119, 773-783. |
|  |  | Chamberlin, H.M. and Sternberg, P.W. Mutations in the Caenorhabditis elegans gene vab-3 reveal distinct roles in fate specification and unequal cytokinesis in an asymmetric cell division. Dev Biol 170, 679-689. |
|  |  | Zhang, Y. and Emmons, S.W. Specification of sense-organ identity by a Caenorhabditis elegans Pax-6 homologue. Nature 377, 55-59. |
|  |  | Chisholm, A.D. and Horvitz, H.R. Patterning of the Caenorhabditis elegans head region by the Pax-6 family member vab-3. Nature 377, 52-55. |
|  |  | Baumeister, R., Liu, Y. and Ruvkun, G. Lineage-specific regulators couple cell lineage asymmetry to the transcription of the Caenorhabditis elegans POU gene unc-86 during neurogenesis. Genes Dev 10, 1395-1410. |
|  |  | Ahringer, J. Turn to the worm! Curr Opin Genet Dev 7, 410-415. |
|  |  | Kuwabara, P.E. Worming your way through the genome. Trends Genet 13, 455-460. |
|  |  | Zhang, Y., Ferreira, H.B., Greenstein, D., Chisholm, A. and Emmons, S.W. Regulated nuclear entry of the C. elegans Pax-6 transcription factor. Mech Dev 78, 179-187. |
|  |  | Nishiwaki, K. Mutations affecting symmetrical migration of distal tip cells in Caenorhabditis elegans. Genetics 152, 985-997. |
|  |  | Hobert, O. and Ruvkun, G. Pax genes in Caenorhabditis elegans: a new twist. Trends Genet 15, 214-216. |
|  |  | Troemel, E.R., Sagasti, A. and Bargmann, C.I. Lateral signaling mediated by axon contact and calcium entry regulates asymmetric odorant receptor expression in C. elegans. Cell 99, 387-398. |
|  |  | Chow, K.L. and Chan, K.W. Stress-induced phenocopy of C. elegans defines functional steps of sensory organ differentiation. Dev Growth Differ 41, 629-637. |
|  |  | Aboobaker, A.A. and Blaxter, M.L. Medical significance of Caenorhabditis elegans. Ann Med 32, 23-30. |
|  |  | Hubbard, E.J. and Greenstein, D. The Caenorhabditis elegans gonad: a test tube for cell and developmental biology. Dev Dyn 218, 2-22. |
|  |  | Chin-Sang, I.D. and Chisholm, A.D. Form of the worm: genetics of epidermal morphogenesis in C. elegans. Trends Genet 16, 544-551. |
|  |  | Jiang, M., Ryu, J., Kiraly, M., Duke, K., Reinke, V. and Kim, S.K. Genome-wide analysis of developmental and sex-regulated gene expression profiles in Caenorhabditis elegans. Proc Natl Acad Sci U S A 98, 218-223. |
|  |  | Dozier, C., Kagoshima, H., Niklaus, G., Cassata, G. and Bürglin, T.R. The Caenorhabditis elegans Six/sine oculis class homeobox gene ceh-32 is required for head morphogenesis. Dev Biol 236, 289-303. |
|  |  | Fares, H. and Greenwald, I. Genetic analysis of endocytosis in Caenorhabditis elegans: coelomocyte uptake defective mutants. Genetics 159, 133-145. |
|  |  | Hahn, A.C. and Emmons, S.W. The roles of an ephrin and a semaphorin in patterning cell-cell contacts in C. elegans sensory organ development. Dev Biol 256, 379-388. |
|  |  | Chang, S., Johnston, R.J., Jr. and Hobert, O. A transcriptional regulatory cascade that controls left/right asymmetry in chemosensory neurons of C. elegans. Genes Dev 17, 2123-2137. |
|  |  | Ruvinsky, I. and Ruvkun, G. Functional tests of enhancer conservation between distantly related species. Development 130, 5133-5142. |
|  |  | Lints, R., Jia, L., Kim, K., Li, C. and Emmons, S.W. Axial patterning of C. elegans male sensilla identities by selector genes. Dev Biol 269, 137-151. |
|  |  | Cinar, H.N. and Chisholm, A.D. Genetic analysis of the Caenorhabditis elegans pax-6 locus: roles of paired domain-containing and nonpaired domain-containing isoforms. Genetics 168, 1307-1322. |
|  |  | Bando, T., Ikeda, T. and Kagawa, H. The homeoproteins MAB-18 and CEH-14 insulate the dauer collagen gene col-43 from activation by the adjacent promoter of the Spermatheca gene sth-1 in Caenorhabditis elegans. J Mol Biol 348, 101-112. |
|  |  | Furuya, M., Qadota, H., Chisholm, A.D. and Sugimoto, A. The C. elegans eyes absent ortholog EYA-1 is required for tissue differentiation and plays partially redundant roles with PAX-6. Dev Biol 286, 452-463. |
|  |  | Emmons, S.W. Male development. WormBook, 1-22. |
|  |  | Hobert, O. Specification of the nervous system. WormBook, 1-19. |
|  |  | Okkema, P.G. and Krause, M. Transcriptional regulation. WormBook, 1-40. |
|  |  | Su, C.W., Tharin, S., Jin, Y., Wightman, B., Spector, M., Meili, D., Tsung, N., Rhiner, C., Bourikas, D., Stoeckli, E. et al. The short coiled-coil domain-containing protein UNC-69 cooperates with UNC-76 to regulate axonal outgrowth and normal presynaptic organization in Caenorhabditis elegans. J Biol 5, 9. |
|  |  | Mango, S.E. The C. elegans pharynx: a model for organogenesis. WormBook, 1-26. |
|  |  | Meighan, C.M. and Schwarzbauer, J.E. Control of C. elegans hermaphrodite gonad size and shape by vab-3/Pax6-mediated regulation of integrin receptors. Genes Dev 21, 1615-1620. |
|  |  | Lecroisey, C., Martin, E., Mariol, M.C., Granger, L., Schwab, Y., Labouesse, M., Segalat, L. and Gieseler, K. DYC-1, a protein functionally linked to dystrophin in Caenorhabditis elegans is associated with the dense body, where it interacts with the muscle LIM domain protein ZYX-1. Mol Biol Cell 19, 785-796. |
|  |  | Johnson, R.W. and Chamberlin, H.M. Positive and negative regulatory inputs restrict pax-6/vab-3 transcription to sensory organ precursors in Caenorhabditis elegans. Mech Dev 125, 486-497. |
|  |  | Yoshimura, S., Murray, J.I., Lu, Y., Waterston, R.H. and Shaham, S. mls-2 and vab-3 Control glia development, hlh-17/Olig expression and glia-dependent neurite extension in C. elegans. Development 135, 2263-2275. |
|  |  | Amin, N.M., Lim, S.E., Shi, H., Chan, T.L. and Liu, J. A conserved Six-Eya cassette acts downstream of Wnt signaling to direct non-myogenic versus myogenic fates in the C. elegans postembryonic mesoderm. Dev Biol 331, 350-360. |
|  |  | Jovelin, R. Rapid sequence evolution of transcription factors controlling neuron differentiation in Caenorhabditis. Mol Biol Evol 26, 2373-2386. |
|  |  | Wong, Y.F., Sheng, Q., Chung, J.W., Chan, J.K. and Chow, K.L. mab-31 and the TGF-beta pathway act in the ray lineage to pattern C. elegans male sensory rays. BMC Dev Biol 10, 82. |
| vab-7 | M142.4 | Hodgkin, J. More sex-determination mutants of Caenorhabditis elegans. Genetics 96, 649-664. |
|  |  | Hodgkin, J. Male Phenotypes and Mating Efficiency in CAENORHABDITIS ELEGANS. Genetics 103, 43-64. |
|  |  | Schnabel, H. and Schnabel, R. An Organ-Specific Differentiation Gene, pha-1, from Caenorhabditis elegans. Science 250, 686-688. |
|  |  | Schnabel, H., Bauer, G. and Schnabel, R. Suppressors of the organ-specific differentiation gene pha-1 of Caenorhabditis elegans. Genetics 129, 69-77. |
|  |  | Hodgkin, J. Molecular cloning and duplication of the nematode sex-determining gene tra-1. Genetics 133, 543-560. |
|  |  | Granato, M., Schnabel, H. and Schnabel, R. Genesis of an organ: molecular analysis of the pha-1 gene. Development 120, 3005-3017. |
|  |  | Ahringer, J. Posterior patterning by the Caenorhabditis elegans even-skipped homolog vab-7. Genes Dev 10, 1120-1130. |
|  |  | Hunter, C.P. and Kenyon, C. Spatial and temporal controls target pal-1 blastomere-specification activity to a single blastomere lineage in C. elegans embryos. Cell 87, 217-226. |
|  |  | Ahringer, J. Maternal control of a zygotic patterning gene in Caenorhabditis elegans. Development 124, 3865-3869. |
|  |  | Newman-Smith, E.D. and Rothman, J.H. The maternal-to-zygotic transition in embryonic patterning of Caenorhabditis elegans. Curr Opin Genet Dev 8, 472-480. |
|  |  | Ruvkun, G. and Hobert, O. The taxonomy of developmental control in Caenorhabditis elegans. Science 282, 2033-2041. |
|  |  | Chin-Sang, I.D. and Chisholm, A.D. Form of the worm: genetics of epidermal morphogenesis in C. elegans. Trends Genet 16, 544-551. |
|  |  | Jungblut, B. and Sommer, R.J. The nematode even-skipped homolog vab-7 regulates gonad and vulva position in Pristionchus pacificus. Development 128, 253-261. |
|  |  | Gomes, J.E., Encalada, S.E., Swan, K.A., Shelton, C.A., Carter, J.C. and Bowerman, B. The maternal gene spn-4 encodes a predicted RRM protein required for mitotic spindle orientation and cell fate patterning in early C. elegans embryos. Development 128, 4301-4314. |
|  |  | Esmaeili, B., Ross, J.M., Neades, C., Miller, D.M., 3rd and Ahringer, J. The C. elegans even-skipped homologue, vab-7, specifies DB motoneurone identity and axon trajectory. Development 129, 853-862. |
|  |  | Thor, S. and Thomas, J.B. Motor neuron specification in worms, flies and mice: conserved and 'lost' mechanisms. Curr Opin Genet Dev 12, 558-564. |
|  |  | Chang, S., Johnston, R.J., Jr. and Hobert, O. A transcriptional regulatory cascade that controls left/right asymmetry in chemosensory neurons of C. elegans. Genes Dev 17, 2123-2137. |
|  |  | Pocock, R., Ahringer, J., Mitsch, M., Maxwell, S. and Woollard, A. A regulatory network of T-box genes and the even-skipped homologue vab-7 controls patterning and morphogenesis in C. elegans. Development 131, 2373-2385. |
|  |  | Nelson, C.E., Hersh, B.M. and Carroll, S.B. The regulatory content of intergenic DNA shapes genome architecture. Genome Biol 5, R25. |
|  |  | Baugh, L.R., Hill, A.A., Claggett, J.M., Hill-Harfe, K., Wen, J.C., Slonim, D.K., Brown, E.L. and Hunter, C.P. The homeodomain protein PAL-1 specifies a lineage-specific regulatory network in the C. elegans embryo. Development 132, 1843-1854. |
|  |  | Baugh, L.R., Wen, J.C., Hill, A.A., Slonim, D.K., Brown, E.L. and Hunter, C.P. Synthetic lethal analysis of Caenorhabditis elegans posterior embryonic patterning genes identifies conserved genetic interactions. Genome Biol 6, R45. |
|  |  | Felix, M.A. An inversion in the wiring of an intercellular signal: evolution of Wnt signaling in the nematode vulva. Bioessays 27, 765-769. |
|  |  | Hobert, O. Specification of the nervous system. WormBook, 1-19. |
|  |  | Okkema, P.G. and Krause, M. Transcriptional regulation. WormBook, 1-40. |
|  |  | Edgley, M.L., Baillie, D.L., Riddle, D.L. and Rose, A.M. Genetic balancers. WormBook, 1-32. |
|  |  | Yanai, I., Baugh, L.R., Smith, J.J., Roehrig, C., Shen-Orr, S.S., Claggett, J.M., Hill, A.A., Slonim, D.K. and Hunter, C.P. Pairing of competitive and topologically distinct regulatory modules enhances patterned gene expression. Mol Syst Biol 4, 163. |
|  |  | Jovelin, R. Rapid sequence evolution of transcription factors controlling neuron differentiation in Caenorhabditis. Mol Biol Evol 26, 2373-2386. |
| vab-15 | R07B1.1 | Du, H. and Chalfie, M. Genes regulating touch cell development in Caenorhabditis elegans. Genetics 158, 197-207. |
|  |  | Nelson, C.E., Hersh, B.M. and Carroll, S.B. The regulatory content of intergenic DNA shapes genome architecture. Genome Biol 5, R25. |
|  |  | Hutter, H., Wacker, I., Schmid, C. and Hedgecock, E.M. Novel genes controlling ventral cord asymmetry and navigation of pioneer axons in C. elegans. Dev Biol 284, 260-272. |
|  |  | Okkema, P.G. and Krause, M. Transcriptional regulation. WormBook, 1-40. |
| zag-1 | F28F9.1 | Wacker, I., Schwarz, V., Hedgecock, E.M. and Hutter, H. zag-1, a Zn-finger homeodomain transcription factor controlling neuronal differentiation and axon outgrowth in C. elegans. Development 130, 3795-3805. |
|  |  | Clark, S.G. and Chiu, C. C. elegans ZAG-1, a Zn-finger-homeodomain protein, regulates axonal development and neuronal differentiation. Development 130, 3781-3794. |
|  |  | Hutter, H., Wacker, I., Schmid, C. and Hedgecock, E.M. Novel genes controlling ventral cord asymmetry and navigation of pioneer axons in C. elegans. Dev Biol 284, 260-272. |
|  |  | Schmid, C., Schwarz, V. and Hutter, H. AST-1, a novel ETS-box transcription factor, controls axon guidance and pharynx development in C. elegans. Dev Biol 293, 403-413. |
|  |  | Hobert, O. Specification of the nervous system. WormBook, 1-19. |
|  |  | Okkema, P.G. and Krause, M. Transcriptional regulation. WormBook, 1-40. |
|  |  | Axang, C., Rauthan, M., Hall, D.H. and Pilon, M. Developmental genetics of the C. elegans pharyngeal neurons NSML and NSMR. BMC Dev Biol 8, 38. |
|  |  | Jovelin, R. Rapid sequence evolution of transcription factors controlling neuron differentiation in Caenorhabditis. Mol Biol Evol 26, 2373-2386. |
| zfh-2 | ZC123.3 | Okkema, P.G. and Krause, M. Transcriptional regulation. WormBook, 1-40. |
|  |  | Hurd, D.D. A microcosm of the biomedical research experience for upper-level undergraduates. CBE Life Sci Educ 7, 210-219. |

| egl-38 | C04G2.7 | Trent, C., Tsuing, N. and Horvitz, H.R. Egg-laying defective mutants of the nematode Caenorhabditis elegans. Genetics *104*, 619-647. |
| --- | --- | --- |
|  |  | Chamberlin, H.M., Palmer, R.E., Newman, A.P., Sternberg, P.W., Baillie, D.L. and Thomas, J.H. The PAX gene egl-38 mediates developmental patterning in Caenorhabditis elegans. Development *124*, 3919-3928. |
|  |  | Hobert, O., Tessmar, K. and Ruvkun, G. The Caenorhabditis elegans lim-6 LIM homeobox gene regulates neurite outgrowth and function of particular GABAergic neurons. Development *126*, 1547-1562. |
|  |  | Hobert, O. and Ruvkun, G. Pax genes in Caenorhabditis elegans: a new twist. Trends Genet *15*, 214-216. |
|  |  | Chamberlin, H.M., Brown, K.B., Sternberg, P.W. and Thomas, J.H. Characterization of seven genes affecting Caenorhabditis elegans hindgut development. Genetics *153*, 731-742. |
|  |  | Hubbard, E.J. and Greenstein, D. The Caenorhabditis elegans gonad: a test tube for cell and developmental biology. Dev Dyn *218*, 2-22. |
|  |  | Johnson, A.D., Fitzsimmons, D., Hagman, J. and Chamberlin, H.M. EGL-38 Pax regulates the ovo-related gene lin-48 during Caenorhabditis elegans organ development. Development *128*, 2857-2865. |
|  |  | Lints, R. and Emmons, S.W. Regulation of sex-specific differentiation and mating behavior in C. elegans by a new member of the DM domain transcription factor family. Genes Dev *16*, 2390-2402. |
|  |  | Sewell, S.T., Zhang, G., Uttam, A. and Chamberlin, H.M. Developmental patterning in the Caenorhabditis elegans hindgut. Dev Biol *262*, 88-93. |
|  |  | Wang, X., Greenberg, J.F. and Chamberlin, H.M. Evolution of regulatory elements producing a conserved gene expression pattern in Caenorhabditis. Evol Dev *6*, 237-245. |
|  |  | Dutt, A., Canevascini, S., Froehli-Hoier, E. and Hajnal, A. EGF signal propagation during C. elegans vulval development mediated by ROM-1 rhomboid. PLoS Biol *2*, e334. |
|  |  | Inoue, T., Wang, M., Ririe, T.O., Fernandes, J.S. and Sternberg, P.W. Transcriptional network underlying Caenorhabditis elegans vulval development. Proc Natl Acad Sci U S A *102*, 4972-4977. |
|  |  | Zhang, G., Sleiman, S.F., Tseng, R.J., Rajakumar, V., Wang, X. and Chamberlin, H.M. Alteration of the DNA binding domain disrupts distinct functions of the C. elegans Pax protein EGL-38. Mech Dev *122*, 887-899. |
|  |  | Emmons, S.W. Male development. WormBook, 1-22. |
|  |  | Okkema, P.G. and Krause, M. Transcriptional regulation. WormBook, 1-40. |
|  |  | Sternberg, P.W. Vulval development. WormBook, 1-28. |
|  |  | Huang, L. and Hanna-Rose, W. EGF signaling overcomes a uterine cell death associated with temporal mis-coordination of organogenesis within the C. elegans egg-laying apparatus. Dev Biol *300*, 599-611. |
|  |  | Rajakumar, V. and Chamberlin, H.M. The Pax2/5/8 gene egl-38 coordinates organogenesis of the C. elegans egg-laying system. Dev Biol *301*, 240-253. |
|  |  | Park, D., Jia, H., Rajakumar, V. and Chamberlin, H.M. Pax2/5/8 proteins promote cell survival in C. elegans. Development *133*, 4193-4202. |
|  |  | Fernandes, J.S. and Sternberg, P.W. The tailless ortholog nhr-67 regulates patterning of gene expression and morphogenesis in the C. elegans vulva. PLoS Genet *3*, e69. |
|  |  | Tseng, R.J., Armstrong, K.R., Wang, X. and Chamberlin, H.M. The bromodomain protein LEX-1 acts with TAM-1 to modulate gene expression in C. elegans. Mol Genet Genomics *278*, 507-518. |
|  |  | Sun, H., Nelms, B.L., Sleiman, S.F., Chamberlin, H.M. and Hanna-Rose, W. Modulation of Caenorhabditis elegans transcription factor activity by HIM-8 and the related Zinc-Finger ZIM proteins. Genetics *177*, 1221-1226. |
|  |  | Jarriault, S., Schwab, Y. and Greenwald, I. A Caenorhabditis elegans model for epithelial-neuronal transdifferentiation. Proc Natl Acad Sci U S A *105*, 3790-3795. |
|  |  | Johnson, R.W. and Chamberlin, H.M. Positive and negative regulatory inputs restrict pax-6/vab-3 transcription to sensory organ precursors in Caenorhabditis elegans. Mech Dev *125*, 486-497. |
|  |  | Gravato-Nobre, M.J. and Hodgkin, J. The acyltransferase gene bus-1 exhibits conserved and specific expression in nematode rectal cells and reveals pathogen-induced cell swelling. Dev Dyn *237*, 3762-3776. |
|  |  | Ririe, T.O., Fernandes, J.S. and Sternberg, P.W. The Caenorhabditis elegans vulva: a post-embryonic gene regulatory network controlling organogenesis. Proc Natl Acad Sci U S A *105*, 20095-20099. |
|  |  | Reece-Hoyes, J.S., Deplancke, B., Barrasa, M.I., Hatzold, J., Smit, R.B., Arda, H.E., Pope, P.A., Gaudet, J., Conradt, B. and Walhout, A.J. The C. elegans Snail homolog CES-1 can activate gene expression in vivo and share targets with bHLH transcription factors. Nucleic Acids Res *37*, 3689-3698. |
|  |  | Amin, N.M., Lim, S.E., Shi, H., Chan, T.L. and Liu, J. A conserved Six-Eya cassette acts downstream of Wnt signaling to direct non-myogenic versus myogenic fates in the C. elegans postembryonic mesoderm. Dev Biol *331*, 350-360. |
| pax-1 | K07C11.1 | Hobert, O. and Ruvkun, G. Pax genes in Caenorhabditis elegans: a new twist. Trends Genet *15*, 214-216. |
|  |  | Nelson, C.E., Hersh, B.M. and Carroll, S.B. The regulatory content of intergenic DNA shapes genome architecture. Genome Biol *5*, R25. |
|  |  | Okkema, P.G. and Krause, M. Transcriptional regulation. WormBook, 1-40. |
|  |  | Updike, D.L. and Mango, S.E. Temporal regulation of foregut development by HTZ-1/H2A.Z and PHA-4/FoxA. PLoS Genet *2*, e161. |
|  |  | Amin, N.M., Lim, S.E., Shi, H., Chan, T.L. and Liu, J. A conserved Six-Eya cassette acts downstream of Wnt signaling to direct non-myogenic versus myogenic fates in the C. elegans postembryonic mesoderm. Dev Biol *331*, 350-360. |
|  |  | Raharjo, W.H., Logan, B.C., Wen, S., Kalb, J.M. and Gaudet, J. In vitro and in vivo characterization of Caenorhabditis elegans PHA-4/FoxA response elements. Dev Dyn *239*, 2219-2232. |
| pax-2 | K06B9.5 | Hobert, O. and Ruvkun, G. Pax genes in Caenorhabditis elegans: a new twist. Trends Genet *15*, 214-216. |
|  |  | Wang, X., Greenberg, J.F. and Chamberlin, H.M. Evolution of regulatory elements producing a conserved gene expression pattern in Caenorhabditis. Evol Dev *6*, 237-245. |
|  |  | Okkema, P.G. and Krause, M. Transcriptional regulation. WormBook, 1-40. |
|  |  | Park, D., Jia, H., Rajakumar, V. and Chamberlin, H.M. Pax2/5/8 proteins promote cell survival in C. elegans. Development *133*, 4193-4202. |
|  |  | Fernandes, J.S. and Sternberg, P.W. The tailless ortholog nhr-67 regulates patterning of gene expression and morphogenesis in the C. elegans vulva. PLoS Genet *3*, e69. |
|  |  | Amin, N.M., Lim, S.E., Shi, H., Chan, T.L. and Liu, J. A conserved Six-Eya cassette acts downstream of Wnt signaling to direct non-myogenic versus myogenic fates in the C. elegans postembryonic mesoderm. Dev Biol *331*, 350-360. |
| psa-3 | F39D8.2 | Arata, Y., Kouike, H., Zhang, Y., Herman, M.A., Okano, H. and Sawa, H. Wnt signaling and a Hox protein cooperatively regulate psa-3/Meis to determine daughter cell fate after asymmetric cell division in C. elegans. Dev Cell *11*, 105-115. |
|  |  | Mukherjee, K. and Bürglin, T.R. Comprehensive analysis of animal TALE homeobox genes: new conserved motifs and cases of accelerated evolution. J Mol Evol *65*, 137-153. |
| npax-1 | F21D12.5 | Hobert, O. and Ruvkun, G. Pax genes in Caenorhabditis elegans: a new twist. Trends Genet *15*, 214-216. |
| npax-2 | F48B9.5 | Hobert, O. and Ruvkun, G. Pax genes in Caenorhabditis elegans: a new twist. Trends Genet *15*, 214-216. |
| npax-3 | R13.2 | Hobert, O. and Ruvkun, G. Pax genes in Caenorhabditis elegans: a new twist. Trends Genet *15*, 214-216. |
| npax-4 | C09G9.7 | Hobert, O. and Ruvkun, G. Pax genes in Caenorhabditis elegans: a new twist. Trends Genet *15*, 214-216. |
| ocam-1 | T02B5.2 | Bürglin, T.R. and Cassata, G. Loss and gain of domains during evolution of cut superclass homeobox genes. Int J Dev Biol *46*, 115-123. |

Aamodt, E.J., Chung, M.A., and McGhee, J.D. (1991). Spatial control of gut-specific gene expression during Caenorhabditis elegans development. Science *252*, 579-582.

Aboobaker, A., and Blaxter, M. (2003). Hox gene evolution in nematodes: novelty conserved. Curr Opin Genet Dev *13*, 593-598.

Aboobaker, A.A., and Blaxter, M.L. (2000). Medical significance of Caenorhabditis elegans. Ann Med *32*, 23-30.

Aboobaker, A.A., and Blaxter, M.L. (2003). Hox Gene Loss during Dynamic Evolution of the Nematode Cluster. Curr Biol *13*, 37-40.

Adachi, R., Wakabayashi, T., Oda, N., and Shingai, R. (2008). Modulation of Caenorhabditis elegans chemotaxis by cultivation and assay temperatures. Neurosci Res *60*, 300-306.

Adoutte, A. (2000). Evolutionary biology. Small but mighty timekeepers. Nature *408*, 37-38.

Ahringer, J. (1996). Posterior patterning by the Caenorhabditis elegans even-skipped homolog vab-7. Genes Dev *10*, 1120-1130.

Ahringer, J. (1997). Maternal control of a zygotic patterning gene in Caenorhabditis elegans. Development *124*, 3865-3869.

Ahringer, J. (1997). Turn to the worm! Curr Opin Genet Dev *7*, 410-415.

Ailion, M., and Thomas, J.H. (2000). Dauer formation induced by high temperatures in Caenorhabditis elegans. Genetics *156*, 1047-1067.

Ailion, M., and Thomas, J.H. (2003). Isolation and characterization of high-temperature-induced Dauer formation mutants in Caenorhabditis elegans. Genetics *165*, 127-144.

Albertson, D.G. (1993). Mapping chromosome rearrangement breakpoints to the physical map of Caenorhabditis elegans by fluorescent in situ hybridization. Genetics *134*, 211-219.

Alper, S., and Kenyon, C. (2001). REF-1, a protein with two bHLH domains, alters the pattern of cell fusion in C. elegans by regulating Hox protein activity. Development *128*, 1793-1804.

Alper, S., and Kenyon, C. (2002). The zinc finger protein REF-2 functions with the Hox genes to inhibit cell fusion in the ventral epidermis of C. elegans. Development *129*, 3335-3348.

Altun-Gultekin, Z., Andachi, Y., Tsalik, E.L., Pilgrim, D., Kohara, Y., and Hobert, O. (2001). A regulatory cascade of three homeobox genes, ceh-10, ttx-3 and ceh-23, controls cell fate specification of a defined interneuron class in C. elegans. Development *128*, 1951-1969.

Amin, N.M., Hu, K., Pruyne, D., Terzic, D., Bretscher, A., and Liu, J. (2007). A Zn-finger/FH2-domain containing protein, FOZI-1, acts redundantly with CeMyoD to specify striated body wall muscle fates in the Caenorhabditis elegans postembryonic mesoderm. Development *134*, 19-29.

Amin, N.M., Lim, S.E., Shi, H., Chan, T.L., and Liu, J. (2009). A conserved Six-Eya cassette acts downstream of Wnt signaling to direct non-myogenic versus myogenic fates in the C. elegans postembryonic mesoderm. Dev Biol *331*, 350-360.

Amin, N.M., Shi, H., and Liu, J. (2010). The FoxF/FoxC factor LET-381 directly regulates both cell fate specification and cell differentiation in C. elegans mesoderm development. Development *137*, 1451-1460.

Antebi, A. (2006). Nuclear hormone receptors in C. elegans. WormBook, 1-13.

Ao, W., Gaudet, J., Kent, W.J., Muttumu, S., and Mango, S.E. (2004). Environmentally induced foregut remodeling by PHA-4/FoxA and DAF-12/NHR. Science *305*, 1743-1746.

Aono, S., Legouis, R., Hoose, W.A., and Kemphues, K.J. (2004). PAR-3 is required for epithelial cell polarity in the distal spermatheca of C. elegans. Development *131*, 2865-2874.

Apfeld, J., and Kenyon, C. (1999). Regulation of lifespan by sensory perception in Caenorhabditis elegans. Nature *402*, 804-809.

Arata, Y., Kouike, H., Zhang, Y., Herman, M.A., Okano, H., and Sawa, H. (2006). Wnt signaling and a Hox protein cooperatively regulate psa-3/Meis to determine daughter cell fate after asymmetric cell division in C. elegans. Dev Cell *11*, 105-115.

Armstrong, K.R., and Chamberlin, H.M. (2010). Coordinate regulation of gene expression in the C. elegans excretory cell by the POU domain protein CEH-6. Mol Genet Genomics *283*, 73-87.

Aroian, R.V., Levy, A.D., Koga, M., Ohshima, Y., Kramer, J.M., and Sternberg, P.W. (1993). Splicing in Caenorhabditis elegans does not require an AG at the 3' splice acceptor site. Mol Cell Biol *13*, 626-637.

Aspöck, G., and Bürglin, T.R. (2001). The Caenorhabditis elegans distal-less ortholog ceh-43 is required for development of the anterior hypodermis. Dev Dyn *222*, 403-409.

Aspöck, G., Ruvkun, G., and Bürglin, T.R. (2003). The Caenorhabditis elegans ems class homeobox gene ceh-2 is required for M3 pharynx motoneuron function. Development *130*, 3369-3378.

Aurelio, O., Boulin, T., and Hobert, O. (2003). Identification of spatial and temporal cues that regulate postembryonic expression of axon maintenance factors in the C. elegans ventral nerve cord. Development *130*, 599-610.

Austin, J., and Kenyon, C. (1994). Cell contact regulates neuroblast formation in the Caenorhabditis elegans lateral epidermis. Development *120*, 313-323.

Avery, L. (1993). The genetics of feeding in Caenorhabditis elegans. Genetics *133*, 897-917.

Axang, C., Rauthan, M., Hall, D.H., and Pilon, M. (2007). The twisted pharynx phenotype in C. elegans. BMC Dev Biol *7*, 61.

Axang, C., Rauthan, M., Hall, D.H., and Pilon, M. (2008). Developmental genetics of the C. elegans pharyngeal neurons NSML and NSMR. BMC Dev Biol *8*, 38.

Bacaj, T., Tevlin, M., Lu, Y., and Shaham, S. (2008). Glia are essential for sensory organ function in C. elegans. Science *322*, 744-747.

Baird, S.E., and Ellazar, S.A. (1999). TGFbeta-like signaling and spicule development in Caenorhabditis elegans. Dev Biol *212*, 93-100.

Baird, S.E., Fitch, D.H., Kassem, I.A., and Emmons, S.W. (1991). Pattern formation in the nematode epidermis: determination of the arrangement of peripheral sense organs in the C. elegans male tail. Development *113*, 515-526.

Bando, T., Ikeda, T., and Kagawa, H. (2005). The homeoproteins MAB-18 and CEH-14 insulate the dauer collagen gene col-43 from activation by the adjacent promoter of the Spermatheca gene sth-1 in Caenorhabditis elegans. J Mol Biol *348*, 101-112.

Bany, I.A., Dong, M.Q., and Koelle, M.R. (2003). Genetic and cellular basis for acetylcholine inhibition of Caenorhabditis elegans egg-laying behavior. J Neurosci *23*, 8060-8069.

Baran, R., Aronoff, R., and Garriga, G. (1999). The C. elegans homeodomain gene unc-42 regulates chemosensory and glutamate receptor expression. Development *126*, 2241-2251.

Barbazuk, W.B., Johnsen, R.C., and Baillie, D.L. (1994). The generation and genetic analysis of suppressors of lethal mutations in the Caenorhabditis elegans rol-3(V) gene. Genetics *136*, 129-143.

Bargmann, C.I. (1991). Death from natural and unnatural causes. Curr Biol *1*, 388-390.

Bargmann, C.I. (1993). Genetic and cellular analysis of behavior in C. elegans. Annu Rev Neurosci *16*, 47-71.

Bargmann, C.I. (2006). Chemosensation in C. elegans. WormBook, 1-29.

Barnes, T.M., Jin, Y., Horvitz, H.R., Ruvkun, G., and Hekimi, S. (1996). The Caenorhabditis elegans behavioral gene unc-24 encodes a novel bipartite protein similar to both erythrocyte band 7.2 (stomatin) and nonspecific lipid transfer protein. J Neurochem *67*, 46-57.

Basson, M., and Horvitz, H.R. (1996). The Caenorhabditis elegans gene sem-4 controls neuronal and mesodermal cell development and encodes a zinc finger protein. Genes Dev *10*, 1953-1965.

Baugh, L.R., Hill, A.A., Claggett, J.M., Hill-Harfe, K., Wen, J.C., Slonim, D.K., Brown, E.L., and Hunter, C.P. (2005). The homeodomain protein PAL-1 specifies a lineage-specific regulatory network in the C. elegans embryo. Development *132*, 1843-1854.

Baugh, L.R., Wen, J.C., Hill, A.A., Slonim, D.K., Brown, E.L., and Hunter, C.P. (2005). Synthetic lethal analysis of Caenorhabditis elegans posterior embryonic patterning genes identifies conserved genetic interactions. Genome Biol *6*, R45.

Baum, P.D., Guenther, C., Frank, C.A., Pham, B.V., and Garriga, G. (1999). The Caenorhabditis elegans gene ham-2 links Hox patterning to migration of the HSN motor neuron. Genes Dev *13*, 472-483.

Baumeister, R., Liu, Y., and Ruvkun, G. (1996). Lineage-specific regulators couple cell lineage asymmetry to the transcription of the Caenorhabditis elegans POU gene unc-86 during neurogenesis. Genes Dev *10*, 1395-1410.

Beh, C.T., Ferrari, D.C., Chung, M.A., and McGhee, J.D. (1991). An acid phosphatase as a biochemical marker for intestinal development in the nematode Caenorhabditis elegans. Dev Biol *147*, 133-143.

Belfiore, M., Pugnale, P., Saudan, Z., and Puoti, A. (2004). Roles of the C. elegans cyclophilin-like protein MOG-6 in MEP-1 binding and germline fates. Development *131*, 2935-2945.

Ben Arous, J., Laffont, S., and Chatenay, D. (2009). Molecular and sensory basis of a food related two-state behavior in C. elegans. PLoS One *4*, e7584.

Bender, A.M., Wells, O., and Fay, D.S. (2004). lin-35/Rb and xnp-1/ATR-X function redundantly to control somatic gonad development in C. elegans. Dev Biol *273*, 335-349.

Berger, M.F., Badis, G., Gehrke, A.R., Talukder, S., Philippakis, A.A., Pena-Castillo, L., Alleyne, T.M., Mnaimneh, S., Botvinnik, O.B., Chan, E.T.*, et al.* (2008). Variation in homeodomain DNA binding revealed by high-resolution analysis of sequence preferences. Cell *133*, 1266-1276.

Bertrand, V., and Hobert, O. (2009). Linking asymmetric cell division to the terminal differentiation program of postmitotic neurons in C. elegans. Dev Cell *16*, 563-575.

Bessereau, J.L. (2006). Transposons in C. elegans. WormBook, 1-13.

Bird, D.M. (1992). Sequence comparison of the Caenorhabditis elegans dpy-13 and col-34 genes, and their deduced collagen products. Gene *120*, 261-266.

Blelloch, R., Anna-Arriola, S.S., Gao, D., Li, Y., Hodgkin, J., and Kimble, J. (1999). The gon-1 gene is required for gonadal morphogenesis in Caenorhabditis elegans. Dev Biol *216*, 382-393.

Botas, J. (1993). Control of morphogenesis and differentiation by HOM/Hox genes. Curr Opin Cell Biol *5*, 1015-1022.

Boulin, T., Etchberger, J.F., and Hobert, O. (2006). Reporter gene fusions. WormBook, 1-23.

Boulin, T., Pocock, R., and Hobert, O. (2006). A novel Eph receptor-interacting IgSF protein provides C. elegans motoneurons with midline guidepost function. Curr Biol *16*, 1871-1883.

Bowerman, B. (1998). Maternal control of pattern formation in early Caenorhabditis elegans embryos. Curr Top Dev Biol *39*, 73-117.

Bowerman, B., Ingram, M.K., and Hunter, C.P. (1997). The maternal par genes and the segregation of cell fate specification activities in early Caenorhabditis elegans embryos. Development *124*, 3815-3826.

Branda, C.S., and Stern, M.J. (1999). Cell migration and axon growth cone guidance in Caenorhabditis elegans. Curr Opin Genet Dev *9*, 479-484.

Branda, C.S., and Stern, M.J. (2000). Mechanisms controlling sex myoblast migration in Caenorhabditis elegans hermaphrodites. Dev Biol *226*, 137-151.

Branicky, R., and Hekimi, S. (2005). Specification of muscle neurotransmitter sensitivity by a Paired-like homeodomain protein in Caenorhabditis elegans. Development *132*, 4999-5009.

Brenner, S. (1974). The genetics of Caenorhabditis elegans. Genetics *77*, 71-94.

Brockie, P.J., Madsen, D.M., Zheng, Y., Mellem, J., and Maricq, A.V. (2001). Differential expression of glutamate receptor subunits in the nervous system of Caenorhabditis elegans and their regulation by the homeodomain protein UNC-42. J Neurosci *21*, 1510-1522.

Broday, L., Kolotuev, I., Didier, C., Bhoumik, A., Gupta, B.P., Sternberg, P.W., Podbilewicz, B., and Ronai, Z. (2004). The small ubiquitin-like modifier (SUMO) is required for gonadal and uterine-vulval morphogenesis in Caenorhabditis elegans. Genes Dev *18*, 2380-2391.

Broitman-Maduro, G., Lin, K.T., Hung, W.W., and Maduro, M.F. (2006). Specification of the C. elegans MS blastomere by the T-box factor TBX-35. Development *133*, 3097-3106.

Broitman-Maduro, G., Owraghi, M., Hung, W.W., Kuntz, S., Sternberg, P.W., and Maduro, M.F. (2009). The NK-2 class homeodomain factor CEH-51 and the T-box factor TBX-35 have overlapping function in C. elegans mesoderm development. Development *136*, 2735-2746.

Brownlee, D., Holden-Dye, L., and Walker, R. (2000). The range and biological activity of FMRFamide-related peptides and classical neurotransmitters in nematodes. Adv Parasitol *45*, 109-180.

Brunschwig, K., Wittmann, C., Schnabel, R., Bürglin, T.R., Tobler, H., and Müller, F. (1999). Anterior organization of the Caenorhabditis elegans embryo by the labial-like Hox gene ceh-13. Development *126*, 1537-1546.

Bulik, D.A., and Robbins, P.W. (2002). The Caenorhabditis elegans sqv genes and functions of proteoglycans in development. Biochim Biophys Acta *1573*, 247-257.

Bulow, H.E., Berry, K.L., Topper, L.H., Peles, E., and Hobert, O. (2002). Heparan sulfate proteoglycan-dependent induction of axon branching and axon misrouting by the Kallmann syndrome gene kal-1. Proc Natl Acad Sci U S A *99*, 6346-6351.

Burdine, R.D., Branda, C.S., and Stern, M.J. (1998). EGL-17(FGF) expression coordinates the attraction of the migrating sex myoblasts with vulval induction in C. elegans. Development *125*, 1083-1093.

Bürglin, T.R. (1997). Analysis of TALE superclass homeobox genes (MEIS, PBC, KNOX, Iroquois, TGIF) reveals a novel domain conserved between plants and animals. Nucleic Acids Res *25*, 4173-4180.

Bürglin, T.R. (2008). Evolution of hedgehog and hedgehog-related genes, their origin from Hog proteins in ancestral eukaryotes and discovery of a novel Hint motif. BMC Genomics *9*, 127.

Bürglin, T.R., and Aspöck, G. (1999). Exon duplication from a fork head to a homeodomain protein. Dev Genes Evol *209*, 629-633.

Bürglin, T.R., and Cassata, G. (2002). Loss and gain of domains during evolution of cut superclass homeobox genes. Int J Dev Biol *46*, 115-123.

Bürglin, T.R., Finney, M., Coulson, A., and Ruvkun, G. (1989). Caenorhabditis elegans has scores of homoeobox-containing genes. Nature *341*, 239-243.

Bürglin, T.R., and Ruvkun, G. (1992). New motif in PBX genes. Nat Genet *1*, 319-320.

Bürglin, T.R., and Ruvkun, G. (1993). The Caenorhabditis elegans homeobox gene cluster. Curr Opin Genet Dev *3*, 615-620.

Bürglin, T.R., and Ruvkun, G. (2001). Regulation of ectodermal and excretory function by the C. elegans POU homeobox gene ceh-6. Development *128*, 779-790.

Bürglin, T.R., Ruvkun, G., Coulson, A., Hawkins, N.C., McGhee, J.D., Schaller, D., Wittmann, C., Müller, F., and Waterston, R.H. (1991). Nematode homeobox cluster. Nature *351*, 703.

Burr, A.H., and Gans, C. (1998). Mechanical significance of obliquely striated architecture in nematode muscle. Biol Bull *194*, 1-6.

Cai, Q., Sun, Y., Huang, X., Guo, C., Zhang, Y., Zhu, Z., and Zhang, H. (2008). The Caenorhabditis elegans PcG-like gene sop-2 regulates the temporal and sexual specificities of cell fates. Genetics *178*, 1445-1456.

Cameron, S., Clark, S.G., McDermott, J.B., Aamodt, E., and Horvitz, H.R. (2002). PAG-3, a Zn-finger transcription factor, determines neuroblast fate in C. elegans. Development *129*, 1763-1774.

Cassata, G., Kagoshima, H., Andachi, Y., Kohara, Y., Dürrenberger, M.B., Hall, D.H., and Bürglin, T.R. (2000). The LIM homeobox gene ceh-14 confers thermosensory function to the AFD neurons in Caenorhabditis elegans. Neuron *25*, 587-597.

Cassata, G., Kagoshima, H., Prétôt, R.F., Aspöck, G., Niklaus, G., and Bürglin, T.R. (1998). Rapid expression screening of Caenorhabditis elegans homeobox open reading frames using a two-step polymerase chain reaction promoter-gfp reporter construction technique. Gene *212*, 127-135.

Cassata, G., Kuhn, F., Witmer, A., Kirchhofer, R., and Bürglin, T.R. (2000). A steep thermal gradient thermotaxis assay for the nematode Caenorhabditis elegans. Genesis *27*, 141-144.

Cassata, G., Röhrig, S., Kuhn, F., Hauri, H.P., Baumeister, R., and Bürglin, T.R. (2000). The Caenorhabditis elegans Ldb/NLI/Clim orthologue ldb-1 is required for neuronal function. Dev Biol *226*, 45-56.

Cassata, G., Shemer, G., Morandi, P., Donhauser, R., Podbilewicz, B., and Baumeister, R. (2005). ceh-16/engrailed patterns the embryonic epidermis of Caenorhabditis elegans. Development *132*, 739-749.

Ch'ng, Q., and Kenyon, C. (1999). egl-27 generates anteroposterior patterns of cell fusion in C. elegans by regulating Hox gene expression and Hox protein function. Development *126*, 3303-3312.

Ch'ng, Q., Williams, L., Lie, Y.S., Sym, M., Whangbo, J., and Kenyon, C. (2003). Identification of genes that regulate a left-right asymmetric neuronal migration in Caenorhabditis elegans. Genetics *164*, 1355-1367.

Chalfie, M. (1982). Microtubule structure in Caenorhabditis elegans neurons. Cold Spring Harb Symp Quant Biol *46 Pt 1*, 255-261.

Chalfie, M. (1993). Homeobox genes in Caenorhabditis elegans. Curr Opin Genet Dev *3*, 275-277.

Chalfie, M. (1993). Touch receptor development and function in Caenorhabditis elegans. J Neurobiol *24*, 1433-1441.

Chalfie, M. (1995). The differentiation and function of the touch receptor neurons of Caenorhabditis elegans. Prog Brain Res *105*, 179-182.

Chalfie, M., and Au, M. (1989). Genetic control of differentiation of the Caenorhabditis elegans touch receptor neurons. Science *243*, 1027-1033.

Chalfie, M., Horvitz, H.R., and Sulston, J.E. (1981). Mutations that lead to reiterations in the cell lineages of C. elegans. Cell *24*, 59-69.

Chalfie, M., and Jorgensen, E.M. (1998). C. elegans neuroscience: genetics to genome. Trends Genet *14*, 506-512.

Chalfie, M., and Sulston, J. (1981). Developmental genetics of the mechanosensory neurons of Caenorhabditis elegans. Dev Biol *82*, 358-370.

Chalfie, M., Thomson, J.N., and Sulston, J.E. (1983). Induction of neuronal branching in Caenorhabditis elegans. Science *221*, 61-63.

Chalfie, M., Tu, Y., Euskirchen, G., Ward, W.W., and Prasher, D.C. (1994). Green fluorescent protein as a marker for gene expression. Science *263*, 802-805.

Chamberlin, H.M., Brown, K.B., Sternberg, P.W., and Thomas, J.H. (1999). Characterization of seven genes affecting Caenorhabditis elegans hindgut development. Genetics *153*, 731-742.

Chamberlin, H.M., Palmer, R.E., Newman, A.P., Sternberg, P.W., Baillie, D.L., and Thomas, J.H. (1997). The PAX gene egl-38 mediates developmental patterning in Caenorhabditis elegans. Development *124*, 3919-3928.

Chamberlin, H.M., and Sternberg, P.W. (1995). Mutations in the Caenorhabditis elegans gene vab-3 reveal distinct roles in fate specification and unequal cytokinesis in an asymmetric cell division. Dev Biol *170*, 679-689.

Chamberlin, H.M., and Thomas, J.H. (2000). The bromodomain protein LIN-49 and trithorax-related protein LIN-59 affect development and gene expression in Caenorhabditis elegans. Development *127*, 713-723.

Chang, S., Johnston, R.J., Jr., Frokjaer-Jensen, C., Lockery, S., and Hobert, O. (2004). MicroRNAs act sequentially and asymmetrically to control chemosensory laterality in the nematode. Nature *430*, 785-789.

Chang, S., Johnston, R.J., Jr., and Hobert, O. (2003). A transcriptional regulatory cascade that controls left/right asymmetry in chemosensory neurons of C. elegans. Genes Dev *17*, 2123-2137.

Chasnov, J.R., So, W.K., Chan, C.M., and Chow, K.L. (2007). The species, sex, and stage specificity of a Caenorhabditis sex pheromone. Proc Natl Acad Sci U S A *104*, 6730-6735.

Chen, Z., Eastburn, D.J., and Han, M. (2004). The Caenorhabditis elegans nuclear receptor gene nhr-25 regulates epidermal cell development. Mol Cell Biol *24*, 7345-7358.

Chen, Z., and Han, M. (2001). C. elegans Rb, NuRD, and Ras regulate lin-39-mediated cell fusion during vulval fate specification. Curr Biol *11*, 1874-1879.

Chen, Z., and Han, M. (2001). Role of C. elegans lin-40 MTA in vulval fate specification and morphogenesis. Development *128*, 4911-4921.

Chesney, M.A., Lam, N., Morgan, D.E., Phillips, B.T., and Kimble, J. (2009). C. elegans HLH-2/E/Daughterless controls key regulatory cells during gonadogenesis. Dev Biol *331*, 14-25.

Chi, C.A., Clark, D.A., Lee, S., Biron, D., Luo, L., Gabel, C.V., Brown, J., Sengupta, P., and Samuel, A.D. (2007). Temperature and food mediate long-term thermotactic behavioral plasticity by association-independent mechanisms in C. elegans. J Exp Biol *210*, 4043-4052.

Chiba, C.M., and Rankin, C.H. (1990). A developmental analysis of spontaneous and reflexive reversals in the nematode Caenorhabditis elegans. J Neurobiol *21*, 543-554.

Chin-Sang, I.D., and Chisholm, A.D. (2000). Form of the worm: genetics of epidermal morphogenesis in C. elegans. Trends Genet *16*, 544-551.

Chisholm, A. (1991). Control of cell fate in the tail region of C. elegans by the gene egl-5. Development *111*, 921-932.

Chisholm, A.D., and Hardin, J. (2005). Epidermal morphogenesis. WormBook, 1-22.

Chisholm, A.D., and Horvitz, H.R. (1995). Patterning of the Caenorhabditis elegans head region by the Pax-6 family member vab-3. Nature *377*, 52-55.

Chow, K.L., and Chan, K.W. (1999). Stress-induced phenocopy of C. elegans defines functional steps of sensory organ differentiation. Dev Growth Differ *41*, 629-637.

Chow, K.L., and Emmons, S.W. (1994). HOM-C/Hox genes and four interacting loci determine the morphogenetic properties of single cells in the nematode male tail. Development *120*, 2579-2592.

Chow, K.L., Hall, D.H., and Emmons, S.W. (1995). The mab-21 gene of Caenorhabditis elegans encodes a novel protein required for choice of alternate cell fates. Development *121*, 3615-3626.

Chu, K.W., Chan, S.K., and Chow, K.L. (2005). Improvement of heavy metal stress and toxicity assays by coupling a transgenic reporter in a mutant nematode strain. Aquat Toxicol *74*, 320-332.

Chung, S.H., Clark, D.A., Gabel, C.V., Mazur, E., and Samuel, A.D. (2006). The role of the AFD neuron in C. elegans thermotaxis analyzed using femtosecond laser ablation. BMC Neurosci *7*, 30.

Cinar, H., Keles, S., and Jin, Y. (2005). Expression profiling of GABAergic motor neurons in Caenorhabditis elegans. Curr Biol *15*, 340-346.

Cinar, H.N., and Chisholm, A.D. (2004). Genetic analysis of the Caenorhabditis elegans pax-6 locus: roles of paired domain-containing and nonpaired domain-containing isoforms. Genetics *168*, 1307-1322.

Cinar, H.N., Richards, K.L., Oommen, K.S., and Newman, A.P. (2003). The EGL-13 SOX domain transcription factor affects the uterine pi cell lineages in Caenorhabditis elegans. Genetics *165*, 1623-1628.

Ciosk, R., DePalma, M., and Priess, J.R. (2006). Translational regulators maintain totipotency in the Caenorhabditis elegans germline. Science *311*, 851-853.

Clandinin, T.R., Katz, W.S., and Sternberg, P.W. (1997). Caenorhabditis elegans HOM-C genes regulate the response of vulval precursor cells to inductive signal. Dev Biol *182*, 150-161.

Clandinin, T.R., and Mains, P.E. (1993). Genetic studies of mei-1 gene activity during the transition from meiosis to mitosis in Caenorhabditis elegans. Genetics *134*, 199-210.

Clark, D.A., Biron, D., Sengupta, P., and Samuel, A.D. (2006). The AFD sensory neurons encode multiple functions underlying thermotactic behavior in Caenorhabditis elegans. J Neurosci *26*, 7444-7451.

Clark, D.A., Gabel, C.V., Lee, T.M., and Samuel, A.D. (2007). Short-term adaptation and temporal processing in the cryophilic response of Caenorhabditis elegans. J Neurophysiol *97*, 1903-1910.

Clark, D.V., and Baillie, D.L. (1992). Genetic analysis and complementation by germ-line transformation of lethal mutations in the unc-22 IV region of Caenorhabditis elegans. Mol Gen Genet *232*, 97-105.

Clark, S.G., Chisholm, A.D., and Horvitz, H.R. (1993). Control of cell fates in the central body region of C. elegans by the homeobox gene lin-39. Cell *74*, 43-55.

Clark, S.G., and Chiu, C. (2003). C. elegans ZAG-1, a Zn-finger-homeodomain protein, regulates axonal development and neuronal differentiation. Development *130*, 3781-3794.

Collins, J.J., and Anderson, P. (1994). The Tc5 family of transposable elements in Caenorhabditis elegans. Genetics *137*, 771-781.

Colosimo, M.E., Brown, A., Mukhopadhyay, S., Gabel, C., Lanjuin, A.E., Samuel, A.D., and Sengupta, P. (2004). Identification of thermosensory and olfactory neuron-specific genes via expression profiling of single neuron types. Curr Biol *14*, 2245-2251.

Conradt, B., and Xue, D. (2005). Programmed cell death. WormBook, 1-13.

Corrigan, C., Subramanian, R., and Miller, M.A. (2005). Eph and NMDA receptors control Ca2+/calmodulin-dependent protein kinase II activation during C. elegans oocyte meiotic maturation. Development *132*, 5225-5237.

Corsi, A.K., Brodigan, T.M., Jorgensen, E.M., and Krause, M. (2002). Characterization of a dominant negative C. elegans Twist mutant protein with implications for human Saethre-Chotzen syndrome. Development *129*, 2761-2772.

Corsi, A.K., Kostas, S.A., Fire, A., and Krause, M. (2000). Caenorhabditis elegans twist plays an essential role in non-striated muscle development. Development *127*, 2041-2051.

Costa, M., Weir, M., Coulson, A., Sulston, J., and Kenyon, C. (1988). Posterior pattern formation in C. elegans involves position-specific expression of a gene containing a homeobox. Cell *55*, 747-756.

Coudreuse, D.Y., Roel, G., Betist, M.C., Destree, O., and Korswagen, H.C. (2006). Wnt gradient formation requires retromer function in Wnt-producing cells. Science *312*, 921-924.

Couillault, C., Pujol, N., Reboul, J., Sabatier, L., Guichou, J.F., Kohara, Y., and Ewbank, J.J. (2004). TLR-independent control of innate immunity in Caenorhabditis elegans by the TIR domain adaptor protein TIR-1, an ortholog of human SARM. Nat Immunol *5*, 488-494.

Cowing, D., and Kenyon, C. (1996). Correct Hox gene expression established independently of position in Caenorhabditis elegans. Nature *382*, 353-356.

Cowing, D.W., and Kenyon, C. (1992). Expression of the homeotic gene mab-5 during Caenorhabditis elegans embryogenesis. Development *116*, 481-490.

Cui, M., and Han, M. (2003). Cis regulatory requirements for vulval cell-specific expression of the Caenorhabditis elegans fibroblast growth factor gene egl-17. Dev Biol *257*, 104-116.

Culetto, E., Combes, D., Fedon, Y., Roig, A., Toutant, J.P., and Arpagaus, M. (1999). Structure and promoter activity of the 5' flanking region of ace-1, the gene encoding acetylcholinesterase of class A in Caenorhabditis elegans. J Mol Biol *290*, 951-966.

Culotti, J.G. (1994). Axon guidance mechanisms in Caenorhabditis elegans. Curr Opin Genet Dev *4*, 587-595.

Curran, S.P., and Ruvkun, G. (2007). Lifespan regulation by evolutionarily conserved genes essential for viability. PLoS Genet *3*, e56.

Cutter, A.D., and Payseur, B.A. (2003). Rates of deleterious mutation and the evolution of sex in Caenorhabditis. J Evol Biol *16*, 812-822.

Davidson, E.H., and Ruvkun, G. (1999). Themes from a NASA workshop on gene regulatory processes in development and evolution. J Exp Zool *285*, 104-115.

de Bono, M., Tobin, D.M., Davis, M.W., Avery, L., and Bargmann, C.I. (2002). Social feeding in Caenorhabditis elegans is induced by neurons that detect aversive stimuli. Nature *419*, 899-903.

Deng, H., Sun, Y., Zhang, Y., Luo, X., Hou, W., Yan, L., Chen, Y., Tian, E., Han, J., and Zhang, H. (2007). Transcription factor NFY globally represses the expression of the C. elegans Hox gene Abdominal-B homolog egl-5. Dev Biol *308*, 583-592.

Deplancke, B., Mukhopadhyay, A., Ao, W., Elewa, A.M., Grove, C.A., Martinez, N.J., Sequerra, R., Doucette-Stamm, L., Reece-Hoyes, J.S., Hope, I.A.*, et al.* (2006). A gene-centered C. elegans protein-DNA interaction network. Cell *125*, 1193-1205.

Desai, C., Garriga, G., McIntire, S.L., and Horvitz, H.R. (1988). A genetic pathway for the development of the Caenorhabditis elegans HSN motor neurons. Nature *336*, 638-646.

Desai, C., and Horvitz, H.R. (1989). Caenorhabditis elegans mutants defective in the functioning of the motor neurons responsible for egg laying. Genetics *121*, 703-721.

Didiano, D., and Hobert, O. (2006). Perfect seed pairing is not a generally reliable predictor for miRNA-target interactions. Nat Struct Mol Biol *13*, 849-851.

Didiano, D., and Hobert, O. (2008). Molecular architecture of a miRNA-regulated 3' UTR. Rna *14*, 1297-1317.

Ding, X.C., and Grosshans, H. (2009). Repression of C. elegans microRNA targets at the initiation level of translation requires GW182 proteins. Embo J *28*, 213-222.

Dixon, S.J., Alexander, M., Fernandes, R., Ricker, N., and Roy, P.J. (2006). FGF negatively regulates muscle membrane extension in Caenorhabditis elegans. Development *133*, 1263-1275.

Donohoe, D.R., Jarvis, R.A., Weeks, K., Aamodt, E.J., and Dwyer, D.S. (2009). Behavioral adaptation in C. elegans produced by antipsychotic drugs requires serotonin and is associated with calcium signaling and calcineurin inhibition. Neurosci Res *64*, 280-289.

Donohoe, D.R., Weeks, K., Aamodt, E.J., and Dwyer, D.S. (2008). Antipsychotic drugs alter neuronal development including ALM neuroblast migration and PLM axonal outgrowth in Caenorhabditis elegans. Int J Dev Neurosci *26*, 371-380.

Dozier, C., Kagoshima, H., Niklaus, G., Cassata, G., and Bürglin, T.R. (2001). The Caenorhabditis elegans Six/sine oculis class homeobox gene ceh-32 is required for head morphogenesis. Dev Biol *236*, 289-303.

Draper, B.W., Mello, C.C., Bowerman, B., Hardin, J., and Priess, J.R. (1996). MEX-3 is a KH domain protein that regulates blastomere identity in early C. elegans embryos. Cell *87*, 205-216.

Du, H., and Chalfie, M. (2001). Genes regulating touch cell development in Caenorhabditis elegans. Genetics *158*, 197-207.

Du, H., Gu, G., William, C.M., and Chalfie, M. (1996). Extracellular proteins needed for C. elegans mechanosensation. Neuron *16*, 183-194.

Duerr, J.S. (2006). Immunohistochemistry. WormBook, 1-61.

Dufourcq, P., Victor, M., Gay, F., Calvo, D., Hodgkin, J., and Shi, Y. (2002). Functional requirement for histone deacetylase 1 in Caenorhabditis elegans gonadogenesis. Mol Cell Biol *22*, 3024-3034.

Duggan, A., and Chalfie, M. (1995). Control of neuronal development in Caenorhabditis elegans. Curr Opin Neurobiol *5*, 6-9.

Duggan, A., Ma, C., and Chalfie, M. (1998). Regulation of touch receptor differentiation by the Caenorhabditis elegans mec-3 and unc-86 genes. Development *125*, 4107-4119.

Dutt, A., Canevascini, S., Froehli-Hoier, E., and Hajnal, A. (2004). EGF signal propagation during C. elegans vulval development mediated by ROM-1 rhomboid. PLoS Biol *2*, e334.

Eastman, C., Horvitz, H.R., and Jin, Y. (1999). Coordinated transcriptional regulation of the unc-25 glutamic acid decarboxylase and the unc-47 GABA vesicular transporter by the Caenorhabditis elegans UNC-30 homeodomain protein. J Neurosci *19*, 6225-6234.

Edgar, L.G., Carr, S., Wang, H., and Wood, W.B. (2001). Zygotic expression of the caudal homolog pal-1 is required for posterior patterning in Caenorhabditis elegans embryogenesis. Dev Biol *229*, 71-88.

Edgley, M.L., Baillie, D.L., Riddle, D.L., and Rose, A.M. (2006). Genetic balancers. WormBook, 1-32.

Egan, C.R., Chung, M.A., Allen, F.L., Heschl, M.F., Van Buskirk, C.L., and McGhee, J.D. (1995). A gut-to-pharynx/tail switch in embryonic expression of the Caenorhabditis elegans ges-1 gene centers on two GATA sequences. Dev Biol *170*, 397-419.

Eimer, S., Donhauser, R., and Baumeister, R. (2002). The Caenorhabditis elegans presenilin sel-12 is required for mesodermal patterning and muscle function. Dev Biol *251*, 178-192.

Eisenmann, D.M. (2005). Wnt signaling. WormBook, 1-17.

Eisenmann, D.M., Maloof, J.N., Simske, J.S., Kenyon, C., and Kim, S.K. (1998). The beta-catenin homolog BAR-1 and LET-60 Ras coordinately regulate the Hox gene lin-39 during Caenorhabditis elegans vulval development. Development *125*, 3667-3680.

Eizinger, A., and Sommer, R.J. (1997). The homeotic gene lin-39 and the evolution of nematode epidermal cell fates. Science *278*, 452-455.

Emmons, S.W. (1992). From cell fates to morphology: developmental genetics of the Caenorhabditis elegans male tail. Bioessays *14*, 309-316.

Emmons, S.W. (1996). Simple worms, complex genes. Nature *382*, 301-302.

Emmons, S.W. (2005). Male development. WormBook, 1-22.

Esmaeili, B., Ross, J.M., Neades, C., Miller, D.M., 3rd, and Ahringer, J. (2002). The C. elegans even-skipped homologue, vab-7, specifies DB motoneurone identity and axon trajectory. Development *129*, 853-862.

Etchberger, J.F., Flowers, E.B., Poole, R.J., Bashllari, E., and Hobert, O. (2009). Cis-regulatory mechanisms of left/right asymmetric neuron-subtype specification in C. elegans. Development *136*, 147-160.

Etchberger, J.F., and Hobert, O. (2008). Vector-free DNA constructs improve transgene expression in C. elegans. Nat Methods *5*, 3.

Etchberger, J.F., Lorch, A., Sleumer, M.C., Zapf, R., Jones, S.J., Marra, M.A., Holt, R.A., Moerman, D.G., and Hobert, O. (2007). The molecular signature and cis-regulatory architecture of a C. elegans gustatory neuron. Genes Dev *21*, 1653-1674.

Euling, S., and Ambros, V. (1996). Heterochronic genes control cell cycle progress and developmental competence of C. elegans vulva precursor cells. Cell *84*, 667-676.

Euling, S., and Ambros, V. (1996). Reversal of cell fate determination in Caenorhabditis elegans vulval development. Development *122*, 2507-2515.

Evans, T.C., and Hunter, C.P. (2005). Translational control of maternal RNAs. WormBook, 1-11.

Fares, H., and Greenwald, I. (2001). Genetic analysis of endocytosis in Caenorhabditis elegans: coelomocyte uptake defective mutants. Genetics *159*, 133-145.

Farley, B.M., Pagano, J.M., and Ryder, S.P. (2008). RNA target specificity of the embryonic cell fate determinant POS-1. Rna *14*, 2685-2697.

Faumont, S., Boulin, T., Hobert, O., and Lockery, S.R. (2006). Developmental regulation of whole cell capacitance and membrane current in identified interneurons in C. elegans. J Neurophysiol *95*, 3665-3673.

Fay, D.S., and Han, M. (2000). Mutations in cye-1, a Caenorhabditis elegans cyclin E homolog, reveal coordination between cell-cycle control and vulval development. Development *127*, 4049-4060.

Fay, D.S., Keenan, S., and Han, M. (2002). fzr-1 and lin-35/Rb function redundantly to control cell proliferation in C. elegans as revealed by a nonbiased synthetic screen. Genes Dev *16*, 503-517.

Feinberg, E.H., Vanhoven, M.K., Bendesky, A., Wang, G., Fetter, R.D., Shen, K., and Bargmann, C.I. (2008). GFP Reconstitution Across Synaptic Partners (GRASP) defines cell contacts and synapses in living nervous systems. Neuron *57*, 353-363.

Felix, M.A. (2005). An inversion in the wiring of an intercellular signal: evolution of Wnt signaling in the nematode vulva. Bioessays *27*, 765-769.

Felix, M.A. (2006). Oscheius tipulae. WormBook, 1-8.

Felix, M.A., and Sternberg, P.W. (1998). A gonad-derived survival signal for vulval precursor cells in two nematode species. Curr Biol *8*, 287-290.

Ferguson, E.L., and Horvitz, H.R. (1985). Identification and characterization of 22 genes that affect the vulval cell lineages of the nematode Caenorhabditis elegans. Genetics *110*, 17-72.

Ferguson, E.L., Sternberg, P.W., and Horvitz, H.R. (1987). A genetic pathway for the specification of the vulval cell lineages of Caenorhabditis elegans. Nature *326*, 259-267.

Fernandes, J.S., and Sternberg, P.W. (2007). The tailless ortholog nhr-67 regulates patterning of gene expression and morphogenesis in the C. elegans vulva. PLoS Genet *3*, e69.

Ferreira, H.B., Zhang, Y., Zhao, C., and Emmons, S.W. (1999). Patterning of Caenorhabditis elegans posterior structures by the Abdominal-B homolog, egl-5. Dev Biol *207*, 215-228.

Finney, M., and Ruvkun, G. (1990). The unc-86 gene product couples cell lineage and cell identity in C. elegans. Cell *63*, 895-905.

Finney, M., Ruvkun, G., and Horvitz, H.R. (1988). The C. elegans cell lineage and differentiation gene unc-86 encodes a protein with a homeodomain and extended similarity to transcription factors. Cell *55*, 757-769.

Fitch, D.H. (2005). Introduction to nematode evolution and ecology. WormBook, 1-8.

Fitzgerald, K., Wilkinson, H.A., and Greenwald, I. (1993). glp-1 can substitute for lin-12 in specifying cell fate decisions in Caenorhabditis elegans. Development *119*, 1019-1027.

Forrester, W.C., Kim, C., and Garriga, G. (2004). The Caenorhabditis elegans Ror RTK CAM-1 inhibits EGL-20/Wnt signaling in cell migration. Genetics *168*, 1951-1962.

Forrester, W.C., Perens, E., Zallen, J.A., and Garriga, G. (1998). Identification of Caenorhabditis elegans genes required for neuronal differentiation and migration. Genetics *148*, 151-165.

Fox, R.M., Von Stetina, S.E., Barlow, S.J., Shaffer, C., Olszewski, K.L., Moore, J.H., Dupuy, D., Vidal, M., and Miller, D.M., 3rd (2005). A gene expression fingerprint of C. elegans embryonic motor neurons. BMC Genomics *6*, 42.

Frank, C.A., Baum, P.D., and Garriga, G. (2003). HLH-14 is a C. elegans achaete-scute protein that promotes neurogenesis through asymmetric cell division. Development *130*, 6507-6518.

Fraser, A.G., Kamath, R.S., Zipperlen, P., Martinez-Campos, M., Sohrmann, M., and Ahringer, J. (2000). Functional genomic analysis of C. elegans chromosome I by systematic RNA interference. Nature *408*, 325-330.

Freyd, G., Kim, S.K., and Horvitz, H.R. (1990). Novel cysteine-rich motif and homeodomain in the product of the Caenorhabditis elegans cell lineage gene lin-11. Nature *344*, 876-879.

Friedman, L., Santa Anna-Arriola, S., Hodgkin, J., and Kimble, J. (2000). gon-4, a cell lineage regulator required for gonadogenesis in Caenorhabditis elegans. Dev Biol *228*, 350-362.

Frokjaer-Jensen, C., Ailion, M., and Lockery, S.R. (2008). Ammonium-acetate is sensed by gustatory and olfactory neurons in Caenorhabditis elegans. PLoS One *3*, e2467.

Fukushige, T., Brodigan, T.M., Schriefer, L.A., Waterston, R.H., and Krause, M. (2006). Defining the transcriptional redundancy of early bodywall muscle development in C. elegans: evidence for a unified theory of animal muscle development. Genes Dev *20*, 3395-3406.

Fukushige, T., Hawkins, M.G., and McGhee, J.D. (1998). The GATA-factor elt-2 is essential for formation of the Caenorhabditis elegans intestine. Dev Biol *198*, 286-302.

Fukushige, T., and Krause, M. (2005). The myogenic potency of HLH-1 reveals wide-spread developmental plasticity in early C. elegans embryos. Development *132*, 1795-1805.

Furuya, M., Qadota, H., Chisholm, A.D., and Sugimoto, A. (2005). The C. elegans eyes absent ortholog EYA-1 is required for tissue differentiation and plays partially redundant roles with PAX-6. Dev Biol *286*, 452-463.

Gabel, C.V., Gabel, H., Pavlichin, D., Kao, A., Clark, D.A., and Samuel, A.D. (2007). Neural circuits mediate electrosensory behavior in Caenorhabditis elegans. J Neurosci *27*, 7586-7596.

Garcia, S.M., Casanueva, M.O., Silva, M.C., Amaral, M.D., and Morimoto, R.I. (2007). Neuronal signaling modulates protein homeostasis in Caenorhabditis elegans post-synaptic muscle cells. Genes Dev *21*, 3006-3016.

Garcia-Anoveros, J., and Corey, D.P. (1997). The molecules of mechanosensation. Annu Rev Neurosci *20*, 567-594.

Garriga, G., Desai, C., and Horvitz, H.R. (1993). Cell interactions control the direction of outgrowth, branching and fasciculation of the HSN axons of Caenorhabditis elegans. Development *117*, 1071-1087.

Garriga, G., and Stern, M.J. (1994). Hams and Egls: genetic analysis of cell migration in Caenorhabditis elegans. Curr Opin Genet Dev *4*, 575-580.

Gems, D., and Riddle, D.L. (2000). Genetic, behavioral and environmental determinants of male longevity in Caenorhabditis elegans. Genetics *154*, 1597-1610.

Gengyo-Ando, K., Yoshina, S., Inoue, H., and Mitani, S. (2006). An efficient transgenic system by TA cloning vectors and RNAi for C. elegans. Biochem Biophys Res Commun *349*, 1345-1350.

Ghosh, R., and Emmons, S.W. (2008). Episodic swimming behavior in the nematode C. elegans. J Exp Biol *211*, 3703-3711.

Gilleard, J.S. (2004). The use of Caenorhabditis elegans in parasitic nematode research. Parasitology *128 Suppl 1*, S49-70.

Gissendanner, C.R., Kelley, K., Nguyen, T.Q., Hoener, M.C., Sluder, A.E., and Maina, C.V. (2008). The Caenorhabditis elegans NR4A nuclear receptor is required for spermatheca morphogenesis. Dev Biol *313*, 767-786.

Gladden, J.M., Farboud, B., and Meyer, B.J. (2007). Revisiting the X:A signal that specifies Caenorhabditis elegans sexual fate. Genetics *177*, 1639-1654.

Gladden, J.M., and Meyer, B.J. (2007). A ONECUT homeodomain protein communicates X chromosome dose to specify Caenorhabditis elegans sexual fate by repressing a sex switch gene. Genetics *177*, 1621-1637.

Gleason, J.E., Korswagen, H.C., and Eisenmann, D.M. (2002). Activation of Wnt signaling bypasses the requirement for RTK/Ras signaling during C. elegans vulval induction. Genes Dev *16*, 1281-1290.

Goldstein, B., Takeshita, H., Mizumoto, K., and Sawa, H. (2006). Wnt signals can function as positional cues in establishing cell polarity. Dev Cell *10*, 391-396.

Gomes, J.E., Encalada, S.E., Swan, K.A., Shelton, C.A., Carter, J.C., and Bowerman, B. (2001). The maternal gene spn-4 encodes a predicted RRM protein required for mitotic spindle orientation and cell fate patterning in early C. elegans embryos. Development *128*, 4301-4314.

Gonczy, P., and Rose, L.S. (2005). Asymmetric cell division and axis formation in the embryo. WormBook, 1-20.

Goodman, M.B. (2006). Mechanosensation. WormBook, 1-14.

Goodman, M.B., and Schwarz, E.M. (2003). Transducing touch in Caenorhabditis elegans. Annu Rev Physiol *65*, 429-452.

Govindan, J.A., Cheng, H., Harris, J.E., and Greenstein, D. (2006). Galphao/i and Galphas signaling function in parallel with the MSP/Eph receptor to control meiotic diapause in C. elegans. Curr Biol *16*, 1257-1268.

Gower, N.J., Temple, G.R., Schein, J.E., Marra, M., Walker, D.S., and Baylis, H.A. (2001). Dissection of the promoter region of the inositol 1,4,5-trisphosphate receptor gene, itr-1, in C. elegans: a molecular basis for cell-specific expression of IP3R isoforms. J Mol Biol *306*, 145-157.

Granato, M., Schnabel, H., and Schnabel, R. (1994). Genesis of an organ: molecular analysis of the pha-1 gene. Development *120*, 3005-3017.

Grandien, K., and Sommer, R.J. (2001). Functional comparison of the nematode Hox gene lin-39 in C. elegans and P. pacificus reveals evolutionary conservation of protein function despite divergence of primary sequences. Genes Dev *15*, 2161-2172.

Grant, B.D., and Wilkinson, H.A. (2003). Functional genomic maps in Caenorhabditis elegans. Curr Opin Cell Biol *15*, 206-212.

Grant, K., Hanna-Rose, W., and Han, M. (2000). sem-4 promotes vulval cell-fate determination in Caenorhabditis elegans through regulation of lin-39 Hox. Dev Biol *224*, 496-506.

Gravato-Nobre, M.J., and Hodgkin, J. (2008). The acyltransferase gene bus-1 exhibits conserved and specific expression in nematode rectal cells and reveals pathogen-induced cell swelling. Dev Dyn *237*, 3762-3776.

Gravato-Nobre, M.J., Nicholas, H.R., Nijland, R., O'Rourke, D., Whittington, D.E., Yook, K.J., and Hodgkin, J. (2005). Multiple genes affect sensitivity of Caenorhabditis elegans to the bacterial pathogen Microbacterium nematophilum. Genetics *171*, 1033-1045.

Gray, J.M., Hill, J.J., and Bargmann, C.I. (2005). A circuit for navigation in Caenorhabditis elegans. Proc Natl Acad Sci U S A *102*, 3184-3191.

Greenstein, D. (2005). Control of oocyte meiotic maturation and fertilization. WormBook, 1-12.

Greenstein, D., Hird, S., Plasterk, R.H., Andachi, Y., Kohara, Y., Wang, B., Finney, M., and Ruvkun, G. (1994). Targeted mutations in the Caenorhabditis elegans POU homeo box gene ceh-18 cause defects in oocyte cell cycle arrest, gonad migration, and epidermal differentiation. Genes Dev *8*, 1935-1948.

Guedes, S., and Priess, J.R. (1997). The C. elegans MEX-1 protein is present in germline blastomeres and is a P granule component. Development *124*, 731-739.

Guenther, C., and Garriga, G. (1996). Asymmetric distribution of the C. elegans HAM-1 protein in neuroblasts enables daughter cells to adopt distinct fates. Development *122*, 3509-3518.

Guerry, F., Marti, C.O., Zhang, Y., Moroni, P.S., Jaquiery, E., and Muller, F. (2007). The Mi-2 nucleosome-remodeling protein LET-418 is targeted via LIN-1/ETS to the promoter of lin-39/Hox during vulval development in C. elegans. Dev Biol *306*, 469-479.

Gupta, B.P., and Sternberg, P.W. (2002). Tissue-specific regulation of the LIM homeobox gene lin-11 during development of the Caenorhabditis elegans egg-laying system. Dev Biol *247*, 102-115.

Gupta, B.P., Wang, M., and Sternberg, P.W. (2003). The C. elegans LIM homeobox gene lin-11 specifies multiple cell fates during vulval development. Development *130*, 2589-2601.

Gutierrez, A., Knoch, L., Witte, H., and Sommer, R.J. (2003). Functional specificity of the nematode Hox gene mab-5. Development *130*, 983-993.

Hahn, A.C., and Emmons, S.W. (2003). The roles of an ephrin and a semaphorin in patterning cell-cell contacts in C. elegans sensory organ development. Dev Biol *256*, 379-388.

Hall, D.H., Winfrey, V.P., Blaeuer, G., Hoffman, L.H., Furuta, T., Rose, K.L., Hobert, O., and Greenstein, D. (1999). Ultrastructural features of the adult hermaphrodite gonad of Caenorhabditis elegans: relations between the germ line and soma. Dev Biol *212*, 101-123.

Hallam, S., Singer, E., Waring, D., and Jin, Y. (2000). The C. elegans NeuroD homolog cnd-1 functions in multiple aspects of motor neuron fate specification. Development *127*, 4239-4252.

Hallam, S.J., Goncharov, A., McEwen, J., Baran, R., and Jin, Y. (2002). SYD-1, a presynaptic protein with PDZ, C2 and rhoGAP-like domains, specifies axon identity in C. elegans. Nat Neurosci *5*, 1137-1146.

Hallem, E.A., and Sternberg, P.W. (2008). Acute carbon dioxide avoidance in Caenorhabditis elegans. Proc Natl Acad Sci U S A *105*, 8038-8043.

Hamelin, M., Scott, I.M., Way, J.C., and Culotti, J.G. (1992). The mec-7 beta-tubulin gene of Caenorhabditis elegans is expressed primarily in the touch receptor neurons. Embo J *11*, 2885-2893.

Hamilton, B., Dong, Y., Shindo, M., Liu, W., Odell, I., Ruvkun, G., and Lee, S.S. (2005). A systematic RNAi screen for longevity genes in C. elegans. Genes Dev *19*, 1544-1555.

Hammell, C.M., Lubin, I., Boag, P.R., Blackwell, T.K., and Ambros, V. (2009). nhl-2 Modulates microRNA activity in Caenorhabditis elegans. Cell *136*, 926-938.

Hanna-Rose, W., and Han, M. (1999). COG-2, a sox domain protein necessary for establishing a functional vulval-uterine connection in Caenorhabditis elegans. Development *126*, 169-179.

Harbinder, S., Tavernarakis, N., Herndon, L.A., Kinnell, M., Xu, S.Q., Fire, A., and Driscoll, M. (1997). Genetically targeted cell disruption in Caenorhabditis elegans. Proc Natl Acad Sci U S A *94*, 13128-13133.

Hardin, J., and King, R.S. (2008). The long and the short of Wnt signaling in C. elegans. Curr Opin Genet Dev *18*, 362-367.

Harfe, B.D., Branda, C.S., Krause, M., Stern, M.J., and Fire, A. (1998). MyoD and the specification of muscle and non-muscle fates during postembryonic development of the C. elegans mesoderm. Development *125*, 2479-2488.

Harfe, B.D., and Fire, A. (1998). Muscle and nerve-specific regulation of a novel NK-2 class homeodomain factor in Caenorhabditis elegans. Development *125*, 421-429.

Harfe, B.D., Vaz Gomes, A., Kenyon, C., Liu, J., Krause, M., and Fire, A. (1998). Analysis of a Caenorhabditis elegans Twist homolog identifies conserved and divergent aspects of mesodermal patterning. Genes Dev *12*, 2623-2635.

Harris, J., Honigberg, L., Robinson, N., and Kenyon, C. (1996). Neuronal cell migration in C. elegans: regulation of Hox gene expression and cell position. Development *122*, 3117-3131.

Harris, J.E., Govindan, J.A., Yamamoto, I., Schwartz, J., Kaverina, I., and Greenstein, D. (2006). Major sperm protein signaling promotes oocyte microtubule reorganization prior to fertilization in Caenorhabditis elegans. Dev Biol *299*, 105-121.

Hartman, P.S., and Herman, R.K. (1982). Radiation-sensitive mutants of Caenorhabditis elegans. Genetics *102*, 159-178.

Haun, C., Alexander, J., Stainier, D.Y., and Okkema, P.G. (1998). Rescue of Caenorhabditis elegans pharyngeal development by a vertebrate heart specification gene. Proc Natl Acad Sci U S A *95*, 5072-5075.

Hawkins, N., and Garriga, G. (1998). Asymmetric cell division: from A to Z. Genes Dev *12*, 3625-3638.

Hawkins, N.C., and McGhee, J.D. (1990). Homeobox containing genes in the nematode Caenorhabditis elegans. Nucleic Acids Res *18*, 6101-6106.

Haynes, C.M., Petrova, K., Benedetti, C., Yang, Y., and Ron, D. (2007). ClpP mediates activation of a mitochondrial unfolded protein response in C. elegans. Dev Cell *13*, 467-480.

Hedgecock, E.M., Culotti, J.G., Hall, D.H., and Stern, B.D. (1987). Genetics of cell and axon migrations in Caenorhabditis elegans. Development *100*, 365-382.

Hekimi, S. (2000). Crossroads of aging in the nematode Caenorhabditis elegans. Results Probl Cell Differ *29*, 81-112.

Henderson, M.A., Cronland, E., Dunkelbarger, S., Contreras, V., Strome, S., and Keiper, B.D. (2009). A germline-specific isoform of eIF4E (IFE-1) is required for efficient translation of stored mRNAs and maturation of both oocytes and sperm. J Cell Sci *122*, 1529-1539.

Herman, M. (2001). C. elegans POP-1/TCF functions in a canonical Wnt pathway that controls cell migration and in a noncanonical Wnt pathway that controls cell polarity. Development *128*, 581-590.

Herman, M.A. (2002). Control of cell polarity by noncanonical Wnt signaling in C. elegans. Semin Cell Dev Biol *13*, 233-241.

Herman, M.A. (2006). Hermaphrodite cell-fate specification. WormBook, 1-16.

Herman, M.A., Ch'ng, Q., Hettenbach, S.M., Ratliff, T.M., Kenyon, C., and Herman, R.K. (1999). EGL-27 is similar to a metastasis-associated factor and controls cell polarity and cell migration in C. elegans. Development *126*, 1055-1064.

Herman, M.A., and Wu, M. (2004). Noncanonical Wnt signaling pathways in C. elegans converge on POP-1/TCF and control cell polarity. Front Biosci *9*, 1530-1539.

Herman, R.K. (1978). Crossover suppressors and balanced recessive lethals in Caenorhabditis elegans. Genetics *88*, 49-65.

Herman, R.K. (1989). Mosaic analysis in the nematode Caenorhabditis elegans. J Neurogenet *5*, 1-24.

Herman, R.K. (1993). Neurobiology. Genes make worms behave. Nature *364*, 282-283.

Herman, R.K. (1996). Touch sensation in Caenorhabditis elegans. Bioessays *18*, 199-206.

Hermann, G.J., Leung, B., and Priess, J.R. (2000). Left-right asymmetry in C. elegans intestine organogenesis involves a LIN-12/Notch signaling pathway. Development *127*, 3429-3440.

Herr, W., Sturm, R.A., Clerc, R.G., Corcoran, L.M., Baltimore, D., Sharp, P.A., Ingraham, H.A., Rosenfeld, M.G., Finney, M., Ruvkun, G.*, et al.* (1988). The POU domain: a large conserved region in the mammalian pit-1, oct-1, oct-2, and Caenorhabditis elegans unc-86 gene products. Genes Dev *2*, 1513-1516.

Higashibata, A., Szewczyk, N.J., Conley, C.A., Imamizo-Sato, M., Higashitani, A., and Ishioka, N. (2006). Decreased expression of myogenic transcription factors and myosin heavy chains in Caenorhabditis elegans muscles developed during spaceflight. J Exp Biol *209*, 3209-3218.

Hill, R.J., and Sternberg, P.W. (1992). The gene lin-3 encodes an inductive signal for vulval development in C. elegans. Nature *358*, 470-476.

Hirose, T., Galvin, B.D., and Horvitz, H.R. (2010). Six and Eya promote apoptosis through direct transcriptional activation of the proapoptotic BH3-only gene egl-1 in Caenorhabditis elegans. Proc Natl Acad Sci U S A *107*, 15479-15484.

Hirotsu, T., and Iino, Y. (2005). Neural circuit-dependent odor adaptation in C. elegans is regulated by the Ras-MAPK pathway. Genes Cells *10*, 517-530.

Hobert, O. (2005). Specification of the nervous system. WormBook, 1-19.

Hobert, O., D'Alberti, T., Liu, Y., and Ruvkun, G. (1998). Control of neural development and function in a thermoregulatory network by the LIM homeobox gene lin-11. J Neurosci *18*, 2084-2096.

Hobert, O., Johnston, R.J., Jr., and Chang, S. (2002). Left-right asymmetry in the nervous system: the Caenorhabditis elegans model. Nat Rev Neurosci *3*, 629-640.

Hobert, O., Mori, I., Yamashita, Y., Honda, H., Ohshima, Y., Liu, Y., and Ruvkun, G. (1997). Regulation of interneuron function in the C. elegans thermoregulatory pathway by the ttx-3 LIM homeobox gene. Neuron *19*, 345-357.

Hobert, O., and Ruvkun, G. (1999). Pax genes in Caenorhabditis elegans: a new twist. Trends Genet *15*, 214-216.

Hobert, O., Tessmar, K., and Ruvkun, G. (1999). The Caenorhabditis elegans lim-6 LIM homeobox gene regulates neurite outgrowth and function of particular GABAergic neurons. Development *126*, 1547-1562.

Hodgkin, J. (1980). More sex-determination mutants of Caenorhabditis elegans. Genetics *96*, 649-664.

Hodgkin, J. (1983). Male Phenotypes and Mating Efficiency in CAENORHABDITIS ELEGANS. Genetics *103*, 43-64.

Hodgkin, J. (1987). Sex determination and dosage compensation in Caenorhabditis elegans. Annu Rev Genet *21*, 133-154.

Hodgkin, J. (1991). The interactive worm. Eighth international C. elegans Meeting, Madison, WI, USA, June 1-5, 1991. New Biol *3*, 951-954.

Hodgkin, J. (1993). Molecular cloning and duplication of the nematode sex-determining gene tra-1. Genetics *133*, 543-560.

Hodgkin, J. (1994). Epigenetics and the maintenance of gene activity states in Caenorhabditis elegans. Dev Genet *15*, 471-477.

Hodgkin, J. (2005). Genetic suppression. WormBook, 1-13.

Hodgkin, J., Horvitz, H.R., and Brenner, S. (1979). Nondisjunction Mutants of the Nematode CAENORHABDITIS ELEGANS. Genetics *91*, 67-94.

Hodgkin, J.A., and Brenner, S. (1977). Mutations causing transformation of sexual phenotype in the nematode Caenorhabditis elegans. Genetics *86*, 275-287.

Hofmann, E.R., Milstein, S., Boulton, S.J., Ye, M., Hofmann, J.J., Stergiou, L., Gartner, A., Vidal, M., and Hengartner, M.O. (2002). Caenorhabditis elegans HUS-1 is a DNA damage checkpoint protein required for genome stability and EGL-1-mediated apoptosis. Curr Biol *12*, 1908-1918.

Hoier, E.F., Mohler, W.A., Kim, S.K., and Hajnal, A. (2000). The Caenorhabditis elegans APC-related gene apr-1 is required for epithelial cell migration and Hox gene expression. Genes Dev *14*, 874-886.

Honigberg, L., and Kenyon, C. (2000). Establishment of left/right asymmetry in neuroblast migration by UNC-40/DCC, UNC-73/Trio and DPY-19 proteins in C. elegans. Development *127*, 4655-4668.

Horvitz, H.R. (1988). Genetic control of Caenorhabditis elegans cell lineage. Harvey Lect *84*, 65-77.

Horvitz, H.R., Chalfie, M., Trent, C., Sulston, J.E., and Evans, P.D. (1982). Serotonin and octopamine in the nematode Caenorhabditis elegans. Science *216*, 1012-1014.

Horvitz, H.R., and Sternberg, P.W. (1991). Multiple intercellular signalling systems control the development of the Caenorhabditis elegans vulva. Nature *351*, 535-541.

Horvitz, H.R., Sternberg, P.W., Greenwald, I.S., Fixsen, W., and Ellis, H.M. (1983). Mutations that affect neural cell lineages and cell fates during the development of the nematode Caenorhabditis elegans. Cold Spring Harb Symp Quant Biol *48 Pt 2*, 453-463.

Horvitz, H.R., and Sulston, J.E. (1980). Isolation and genetic characterization of cell-lineage mutants of the nematode Caenorhabditis elegans. Genetics *96*, 435-454.

Hoshi, K., and Shingai, R. (2006). Computer-driven automatic identification of locomotion states in Caenorhabditis elegans. J Neurosci Methods *157*, 355-363.

Howard, R.M., and Sundaram, M.V. (2002). C. elegans EOR-1/PLZF and EOR-2 positively regulate Ras and Wnt signaling and function redundantly with LIN-25 and the SUR-2 Mediator component. Genes Dev *16*, 1815-1827.

Howell, K., Arur, S., Schedl, T., and Sundaram, M.V. (2010). EOR-2 is an obligate binding partner of the BTB-zinc finger protein EOR-1 in Caenorhabditis elegans. Genetics *184*, 899-913.

Hsieh, J., Liu, J., Kostas, S.A., Chang, C., Sternberg, P.W., and Fire, A. (1999). The RING finger/B-box factor TAM-1 and a retinoblastoma-like protein LIN-35 modulate context-dependent gene silencing in Caenorhabditis elegans. Genes Dev *13*, 2958-2970.

Hu, E., and Rubin, C.S. (1991). Casein kinase II from Caenorhabditis elegans. Cloning, characterization, and developmental regulation of the gene encoding the beta subunit. J Biol Chem *266*, 19796-19802.

Huang, L., and Hanna-Rose, W. (2006). EGF signaling overcomes a uterine cell death associated with temporal mis-coordination of organogenesis within the C. elegans egg-laying apparatus. Dev Biol *300*, 599-611.

Huang, L.S., and Sternberg, P.W. (2006). Genetic dissection of developmental pathways. WormBook, 1-19.

Huang, M., and Chalfie, M. (1994). Gene interactions affecting mechanosensory transduction in Caenorhabditis elegans. Nature *367*, 467-470.

Huang, M., Gu, G., Ferguson, E.L., and Chalfie, M. (1995). A stomatin-like protein necessary for mechanosensation in C. elegans. Nature *378*, 292-295.

Huang, N.N., Mootz, D.E., Walhout, A.J., Vidal, M., and Hunter, C.P. (2002). MEX-3 interacting proteins link cell polarity to asymmetric gene expression in Caenorhabditis elegans. Development *129*, 747-759.

Huang, X., Powell-Coffman, J.A., and Jin, Y. (2004). The AHR-1 aryl hydrocarbon receptor and its co-factor the AHA-1 aryl hydrocarbon receptor nuclear translocator specify GABAergic neuron cell fate in C. elegans. Development *131*, 819-828.

Huang, X., Tian, E., Xu, Y., and Zhang, H. (2009). The C. elegans engrailed homolog ceh-16 regulates the self-renewal expansion division of stem cell-like seam cells. Dev Biol *333*, 337-347.

Huang, X.Y., Barrios, L.A., Vonkhorporn, P., Honda, S., Albertson, D.G., and Hecht, R.M. (1989). Genomic organization of the glyceraldehyde-3-phosphate dehydrogenase gene family of Caenorhabditis elegans. J Mol Biol *206*, 411-424.

Hubbard, E.J., and Greenstein, D. (2000). The Caenorhabditis elegans gonad: a test tube for cell and developmental biology. Dev Dyn *218*, 2-22.

Hueber, S.D., Weiller, G.F., Djordjevic, M.A., and Frickey, T. (2010). Improving Hox protein classification across the major model organisms. PLoS One *5*, e10820.

Hukema, R.K., Rademakers, S., Dekkers, M.P., Burghoorn, J., and Jansen, G. (2006). Antagonistic sensory cues generate gustatory plasticity in Caenorhabditis elegans. Embo J *25*, 312-322.

Hung, W., Hwang, C., Po, M.D., and Zhen, M. (2007). Neuronal polarity is regulated by a direct interaction between a scaffolding protein, Neurabin, and a presynaptic SAD-1 kinase in Caenorhabditis elegans. Development *134*, 237-249.

Hung, Y.C., Lee, J.H., Chen, H.M., and Huang, G.S. (2010). Effects of static magnetic fields on the development and aging of Caenorhabditis elegans. J Exp Biol *213*, 2079-2085.

Hunter, C.P., Harris, J.M., Maloof, J.N., and Kenyon, C. (1999). Hox gene expression in a single Caenorhabditis elegans cell is regulated by a caudal homolog and intercellular signals that inhibit wnt signaling. Development *126*, 805-814.

Hunter, C.P., and Kenyon, C. (1995). Specification of anteroposterior cell fates in Caenorhabditis elegans by Drosophila Hox proteins. Nature *377*, 229-232.

Hunter, C.P., and Kenyon, C. (1996). Spatial and temporal controls target pal-1 blastomere-specification activity to a single blastomere lineage in C. elegans embryos. Cell *87*, 217-226.

Hurd, D.D. (2008). A microcosm of the biomedical research experience for upper-level undergraduates. CBE Life Sci Educ *7*, 210-219.

Hurd, D.D., and Kemphues, K.J. (2003). PAR-1 is required for morphogenesis of the Caenorhabditis elegans vulva. Dev Biol *253*, 54-65.

Hutter, H. (2003). Extracellular cues and pioneers act together to guide axons in the ventral cord of C. elegans. Development *130*, 5307-5318.

Hutter, H., Wacker, I., Schmid, C., and Hedgecock, E.M. (2005). Novel genes controlling ventral cord asymmetry and navigation of pioneer axons in C. elegans. Dev Biol *284*, 260-272.

Hwang, H.Y., and Horvitz, H.R. (2002). The Caenorhabditis elegans vulval morphogenesis gene sqv-4 encodes a UDP-glucose dehydrogenase that is temporally and spatially regulated. Proc Natl Acad Sci U S A *99*, 14224-14229.

Hwang, S.B., and Lee, J. (2003). Neuron cell type-specific SNAP-25 expression driven by multiple regulatory elements in the nematode Caenorhabditis elegans. J Mol Biol *333*, 237-247.

Iino, Y., and Yoshida, K. (2009). Parallel use of two behavioral mechanisms for chemotaxis in Caenorhabditis elegans. J Neurosci *29*, 5370-5380.

Im, S.H., and Lee, J. (2003). Identification of HMG-5 as a double-stranded telomeric DNA-binding protein in the nematode Caenorhabditis elegans. FEBS Lett *554*, 455-461.

Inoue, T., Sherwood, D.R., Aspöck, G., Butler, J.A., Gupta, B.P., Kirouac, M., Wang, M., Lee, P.Y., Kramer, J.M., Hope, I.*, et al.* (2002). Gene expression markers for Caenorhabditis elegans vulval cells. Mech Dev *119 Suppl 1*, S203-209.

Inoue, T., and Sternberg, P.W. (2010). C. elegans BED domain transcription factor BED-3 controls lineage-specific cell proliferation during organogenesis. Dev Biol *338*, 226-236.

Inoue, T., Wang, M., Ririe, T.O., Fernandes, J.S., and Sternberg, P.W. (2005). Transcriptional network underlying Caenorhabditis elegans vulval development. Proc Natl Acad Sci U S A *102*, 4972-4977.

Irazoqui, J.E., Ng, A., Xavier, R.J., and Ausubel, F.M. (2008). Role for beta-catenin and HOX transcription factors in Caenorhabditis elegans and mammalian host epithelial-pathogen interactions. Proc Natl Acad Sci U S A *105*, 17469-17474.

Ishihara, T., Iino, Y., Mohri, A., Mori, I., Gengyo-Ando, K., Mitani, S., and Katsura, I. (2002). HEN-1, a secretory protein with an LDL receptor motif, regulates sensory integration and learning in Caenorhabditis elegans. Cell *109*, 639-649.

Ito, H., Inada, H., and Mori, I. (2006). Quantitative analysis of thermotaxis in the nematode Caenorhabditis elegans. J Neurosci Methods *154*, 45-52.

Jarriault, S., Schwab, Y., and Greenwald, I. (2008). A Caenorhabditis elegans model for epithelial-neuronal transdifferentiation. Proc Natl Acad Sci U S A *105*, 3790-3795.

Jia, Y., Xie, G., and Aamodt, E. (1996). pag-3, a Caenorhabditis elegans gene involved in touch neuron gene expression and coordinated movement. Genetics *142*, 141-147.

Jia, Y., Xie, G., McDermott, J.B., and Aamodt, E. (1997). The C. elegans gene pag-3 is homologous to the zinc finger proto-oncogene gfi-1. Development *124*, 2063-2073.

Jiang, L.I., and Sternberg, P.W. (1998). Interactions of EGF, Wnt and HOM-C genes specify the P12 neuroectoblast fate in C. elegans. Development *125*, 2337-2347.

Jiang, M., Ryu, J., Kiraly, M., Duke, K., Reinke, V., and Kim, S.K. (2001). Genome-wide analysis of developmental and sex-regulated gene expression profiles in Caenorhabditis elegans. Proc Natl Acad Sci U S A *98*, 218-223.

Jiang, Y., Horner, V., and Liu, J. (2005). The HMX homeodomain protein MLS-2 regulates cleavage orientation, cell proliferation and cell fate specification in the C. elegans postembryonic mesoderm. Development *132*, 4119-4130.

Jiang, Y., Shi, H., Amin, N.M., Sultan, I., and Liu, J. (2008). Mesodermal expression of the C. elegans HMX homolog mls-2 requires the PBC homolog CEH-20. Mech Dev *125*, 451-461.

Jiang, Y., Shi, H., and Liu, J. (2009). Two Hox cofactors, the Meis/Hth homolog UNC-62 and the Pbx/Exd homolog CEH-20, function together during C. elegans postembryonic mesodermal development. Dev Biol *334*, 535-546.

Jin, Y. (2005). Synaptogenesis. WormBook, 1-11.

Jin, Y., Hoskins, R., and Horvitz, H.R. (1994). Control of type-D GABAergic neuron differentiation by C. elegans UNC-30 homeodomain protein. Nature *372*, 780-783.

Johnsen, R.C., and Baillie, D.L. (1991). Genetic analysis of a major segment [LGV(left)] of the genome of Caenorhabditis elegans. Genetics *129*, 735-752.

Johnson, A.D., Fitzsimmons, D., Hagman, J., and Chamberlin, H.M. (2001). EGL-38 Pax regulates the ovo-related gene lin-48 during Caenorhabditis elegans organ development. Development *128*, 2857-2865.

Johnson, N.M., Behm, C.A., and Trowell, S.C. (2005). Heritable and inducible gene knockdown in C. elegans using Wormgate and the ORFeome. Gene *359*, 26-34.

Johnson, R.W., and Chamberlin, H.M. (2008). Positive and negative regulatory inputs restrict pax-6/vab-3 transcription to sensory organ precursors in Caenorhabditis elegans. Mech Dev *125*, 486-497.

Johnson, T.E., and Lithgow, G.J. (1992). The search for the genetic basis of aging: the identification of gerontogenes in the nematode Caenorhabditis elegans. J Am Geriatr Soc *40*, 936-945.

Johnston, R.J., and Hobert, O. (2003). A microRNA controlling left/right neuronal asymmetry in Caenorhabditis elegans. Nature *426*, 845-849.

Johnston, R.J., Jr., Chang, S., Etchberger, J.F., Ortiz, C.O., and Hobert, O. (2005). MicroRNAs acting in a double-negative feedback loop to control a neuronal cell fate decision. Proc Natl Acad Sci U S A *102*, 12449-12454.

Johnston, R.J., Jr., Copeland, J.W., Fasnacht, M., Etchberger, J.F., Liu, J., Honig, B., and Hobert, O. (2006). An unusual Zn-finger/FH2 domain protein controls a left/right asymmetric neuronal fate decision in C. elegans. Development *133*, 3317-3328.

Johnston, R.J., Jr., and Hobert, O. (2005). A novel C. elegans zinc finger transcription factor, lsy-2, required for the cell type-specific expression of the lsy-6 microRNA. Development *132*, 5451-5460.

Jorgensen, E.M., and Mango, S.E. (2002). The art and design of genetic screens: caenorhabditis elegans. Nat Rev Genet *3*, 356-369.

Jovelin, R. (2009). Rapid sequence evolution of transcription factors controlling neuron differentiation in Caenorhabditis. Mol Biol Evol *26*, 2373-2386.

Jovelin, R., Dunham, J.P., Sung, F.S., and Phillips, P.C. (2009). High nucleotide divergence in developmental regulatory genes contrasts with the structural elements of olfactory pathways in caenorhabditis. Genetics *181*, 1387-1397.

Jud, M.C., Czerwinski, M.J., Wood, M.P., Young, R.A., Gallo, C.M., Bickel, J.S., Petty, E.L., Mason, J.M., Little, B.A., Padilla, P.A.*, et al.* (2008). Large P body-like RNPs form in C. elegans oocytes in response to arrested ovulation, heat shock, osmotic stress, and anoxia and are regulated by the major sperm protein pathway. Dev Biol *318*, 38-51.

Jungblut, B., Pires-daSilva, A., and Sommer, R.J. (2001). Formation of the egg-laying system in Pristionchus pacificus requires complex interactions between gonadal, mesodermal and epidermal tissues and does not rely on single cell inductions. Development *128*, 3395-3404.

Jungblut, B., and Sommer, R.J. (1998). The Pristionchus pacificus mab-5 gene is involved in the regulation of ventral epidermal cell fates. Curr Biol *8*, 775-778.

Jungblut, B., and Sommer, R.J. (2000). Novel cell-cell interactions during vulva development in Pristionchus pacificus. Development *127*, 3295-3303.

Jungblut, B., and Sommer, R.J. (2001). The nematode even-skipped homolog vab-7 regulates gonad and vulva position in Pristionchus pacificus. Development *128*, 253-261.

Jurado, P., Kodama, E., Tanizawa, Y., and Mori, I. (2010). Distinct thermal migration behaviors in response to different thermal gradients in Caenorhabditis elegans. Genes Brain Behav *9*, 120-127.

Kage, E., Hayashi, Y., Takeuchi, H., Hirotsu, T., Kunitomo, H., Inoue, T., Arai, H., Iino, Y., and Kubo, T. (2005). MBR-1, a novel helix-turn-helix transcription factor, is required for pruning excessive neurites in Caenorhabditis elegans. Curr Biol *15*, 1554-1559.

Kagoshima, H., Cassata, G., and Bürglin, T.R. (1999). A Caenorhabditis elegans homeobox gene expressed in the male tail, a link between pattern formation and sexual dimorphism? Dev Genes Evol *209*, 59-62.

Kagoshima, H., Sommer, R., Reinhart, B.J., Cassata, G., Ruvkun, G., and Bürglin, T.R. (2000). Graded expression of ceh-14 reporters in the hypodermis is induced by a gonadal signal. Dev Genes Evol *210*, 564-569.

Kalb, J.M., Beaster-Jones, L., Fernandez, A.P., Okkema, P.G., Goszczynski, B., and McGhee, J.D. (2002). Interference between the PHA-4 and PEB-1 transcription factors in formation of the Caenorhabditis elegans pharynx. J Mol Biol *320*, 697-704.

Kalb, J.M., Lau, K.K., Goszczynski, B., Fukushige, T., Moons, D., Okkema, P.G., and McGhee, J.D. (1998). pha-4 is Ce-fkh-1, a fork head/HNF-3alpha,beta,gamma homolog that functions in organogenesis of the C. elegans pharynx. Development *125*, 2171-2180.

Kalis, A.K., Kroetz, M.B., Larson, K.M., and Zarkower, D. (2010). Functional genomic identification of genes required for male gonadal differentiation in Caenorhabditis elegans. Genetics *185*, 523-535.

Kalis, A.K., Murphy, M.W., and Zarkower, D. (2010). EGL-5/ABD-B plays an instructive role in male cell fate determination in the C. elegans somatic gonad. Dev Biol *344*, 827-835.

Kaltenbach, L.S., Updike, D.L., and Mango, S.E. (2005). Contribution of the amino and carboxyl termini for PHA-4/FoxA function in Caenorhabditis elegans. Dev Dyn *234*, 346-354.

Kamath, R.S., Fraser, A.G., Dong, Y., Poulin, G., Durbin, R., Gotta, M., Kanapin, A., Le Bot, N., Moreno, S., Sohrmann, M.*, et al.* (2003). Systematic functional analysis of the Caenorhabditis elegans genome using RNAi. Nature *421*, 231-237.

Kamath, R.S., Martinez-Campos, M., Zipperlen, P., Fraser, A.G., and Ahringer, J. (2001). Effectiveness of specific RNA-mediated interference through ingested double-stranded RNA in Caenorhabditis elegans. Genome Biol *2*, RESEARCH0002.

Kang, C., and Avery, L. (2009). Systemic regulation of starvation response in Caenorhabditis elegans. Genes Dev *23*, 12-17.

Katsura, I. (1993). In search of new mutants in cell-signaling systems of the nematode Caenorhabditis elegans. Review. Genetica *88*, 137-146.

Katz, W.S., Hill, R.J., Clandinin, T.R., and Sternberg, P.W. (1995). Different levels of the C. elegans growth factor LIN-3 promote distinct vulval precursor fates. Cell *82*, 297-307.

Katz, W.S., and Sternberg, P.W. (1992). A plethora of intercellular signals during Caenorhabditis elegans development. Curr Opin Cell Biol *4*, 939-947.

Kaufman, E.J., and Miska, E.A. (2010). The microRNAs of Caenorhabditis elegans. Semin Cell Dev Biol *21*, 728-737.

Kemphues, K. (2000). PARsing embryonic polarity. Cell *101*, 345-348.

Kennedy, B.P., Aamodt, E.J., Allen, F.L., Chung, M.A., Heschl, M.F., and McGhee, J.D. (1993). The gut esterase gene (ges-1) from the nematodes Caenorhabditis elegans and Caenorhabditis briggsae. J Mol Biol *229*, 890-908.

Kenyon, C. (1986). A gene involved in the development of the posterior body region of C. elegans. Cell *46*, 477-487.

Kenyon, C. (1988). The nematode Caenorhabditis elegans. Science *240*, 1448-1453.

Kenyon, C.J., Austin, J., Costa, M., Cowing, D.W., Harris, J.M., Honigberg, L., Hunter, C.P., Maloof, J.N., Muller-Immergluck, M.M., Salser, S.J.*, et al.* (1997). The dance of the Hox genes: patterning the anteroposterior body axis of Caenorhabditis elegans. Cold Spring Harb Symp Quant Biol *62*, 293-305.

Kerry, S., TeKippe, M., Gaddis, N.C., and Aballay, A. (2006). GATA transcription factor required for immunity to bacterial and fungal pathogens. PLoS One *1*, e77.

Kim, K., Colosimo, M.E., Yeung, H., and Sengupta, P. (2005). The UNC-3 Olf/EBF protein represses alternate neuronal programs to specify chemosensory neuron identity. Dev Biol *286*, 136-148.

Kim, K., Kim, R., and Sengupta, P. (2010). The HMX/NKX homeodomain protein MLS-2 specifies the identity of the AWC sensory neuron type via regulation of the ceh-36 Otx gene in C. elegans. Development *137*, 963-974.

Kim, K., and Li, C. (2004). Expression and regulation of an FMRFamide-related neuropeptide gene family in Caenorhabditis elegans. J Comp Neurol *475*, 540-550.

Kim, S., Ren, X.C., Fox, E., and Wadsworth, W.G. (1999). SDQR migrations in Caenorhabditis elegans are controlled by multiple guidance cues and changing responses to netrin UNC-6. Development *126*, 3881-3890.

Kim, S.H., Hwang, S.B., Chung, I.K., and Lee, J. (2003). Sequence-specific binding to telomeric DNA by CEH-37, a homeodomain protein in the nematode Caenorhabditis elegans. J Biol Chem *278*, 28038-28044.

Klerkx, E.P., Alarcon, P., Waters, K., Reinke, V., Sternberg, P.W., and Askjaer, P. (2009). Protein kinase VRK-1 regulates cell invasion and EGL-17/FGF signaling in Caenorhabditis elegans. Dev Biol *335*, 12-21.

Knobel, K.M., Jorgensen, E.M., and Bastiani, M.J. (1999). Growth cones stall and collapse during axon outgrowth in Caenorhabditis elegans. Development *126*, 4489-4498.

Kobayashi, T., Gengyo-Ando, K., Ishihara, T., Katsura, I., and Mitani, S. (2007). IFT-81 and IFT-74 are required for intraflagellar transport in C. elegans. Genes Cells *12*, 593-602.

Koga, M., and Ohshima, Y. (2004). The C. elegans ceh-36 gene encodes a putative homemodomain transcription factor involved in chemosensory functions of ASE and AWC neurons. J Mol Biol *336*, 579-587.

Koh, K., Bernstein, Y., and Sundaram, M.V. (2004). The nT1 translocation separates vulval regulatory elements from the egl-18 and elt-6 GATA factor genes. Dev Biol *267*, 252-263.

Koh, K., Peyrot, S.M., Wood, C.G., Wagmaister, J.A., Maduro, M.F., Eisenmann, D.M., and Rothman, J.H. (2002). Cell fates and fusion in the C. elegans vulval primordium are regulated by the EGL-18 and ELT-6 GATA factors -- apparent direct targets of the LIN-39 Hox protein. Development *129*, 5171-5180.

Kolotuev, I., and Podbilewicz, B. (2008). Changing of the cell division axes drives vulva evolution in nematodes. Dev Biol *313*, 142-154.

Konwerski, J., Senchuk, M., Petty, E., Lahaie, D., and Schisa, J.A. (2005). Cloning and expression analysis of pos-1 in the nematodes Caenorhabditis briggsae and Caenorhabditis remanei. Dev Dyn *233*, 1006-1012.

Korswagen, H.C. (2002). Canonical and non-canonical Wnt signaling pathways in Caenorhabditis elegans: variations on a common signaling theme. Bioessays *24*, 801-810.

Korswagen, H.C., Coudreuse, D.Y., Betist, M.C., van de Water, S., Zivkovic, D., and Clevers, H.C. (2002). The Axin-like protein PRY-1 is a negative regulator of a canonical Wnt pathway in C. elegans. Genes Dev *16*, 1291-1302.

Korswagen, H.C., Durbin, R.M., Smits, M.T., and Plasterk, R.H. (1996). Transposon Tc1-derived, sequence-tagged sites in Caenorhabditis elegans as markers for gene mapping. Proc Natl Acad Sci U S A *93*, 14680-14685.

Korswagen, H.C., Herman, M.A., and Clevers, H.C. (2000). Distinct beta-catenins mediate adhesion and signalling functions in C. elegans. Nature *406*, 527-532.

Kostas, S.A., and Fire, A. (2002). The T-box factor MLS-1 acts as a molecular switch during specification of nonstriated muscle in C. elegans. Genes Dev *16*, 257-269.

Krause, M. (1995). MyoD and myogenesis in C. elegans. Bioessays *17*, 219-228.

Kuchenthal, C.A., Chen, W., and Okkema, P.G. (2001). Multiple enhancers contribute to expression of the NK-2 homeobox gene ceh-22 in C. elegans pharyngeal muscle. Genesis *31*, 156-166.

Kuersten, S., and Goodwin, E.B. (2003). The power of the 3' UTR: translational control and development. Nat Rev Genet *4*, 626-637.

Kuhara, A., Inada, H., Katsura, I., and Mori, I. (2002). Negative regulation and gain control of sensory neurons by the C. elegans calcineurin TAX-6. Neuron *33*, 751-763.

Kuhara, A., Okumura, M., Kimata, T., Tanizawa, Y., Takano, R., Kimura, K.D., Inada, H., Matsumoto, K., and Mori, I. (2008). Temperature sensing by an olfactory neuron in a circuit controlling behavior of C. elegans. Science *320*, 803-807.

Kuntz, S.G., Schwarz, E.M., DeModena, J.A., De Buysscher, T., Trout, D., Shizuya, H., Sternberg, P.W., and Wold, B.J. (2008). Multigenome DNA sequence conservation identifies Hox cis-regulatory elements. Genome Res *18*, 1955-1968.

Kuwabara, P.E. (1997). Worming your way through the genome. Trends Genet *13*, 455-460.

Kuwabara, P.E. (2003). The multifaceted C. elegans major sperm protein: an ephrin signaling antagonist in oocyte maturation. Genes Dev *17*, 155-161.

Kwon, J.Y., and Lee, J. (2001). Biological significance of a universally conserved transcription mediator in metazoan developmental signaling pathways. Development *128*, 3095-3104.

Kwon, J.Y., Park, J.M., Gim, B.S., Han, S.J., Lee, J., and Kim, Y.J. (1999). Caenorhabditis elegans mediator complexes are required for developmental-specific transcriptional activation. Proc Natl Acad Sci U S A *96*, 14990-14995.

L'Hernault, S.W., Benian, G.M., and Emmons, R.B. (1993). Genetic and molecular characterization of the Caenorhabditis elegans spermatogenesis-defective gene spe-17. Genetics *134*, 769-780.

Labbe, J.C., and Goldstein, B. (2002). Embryonic development: a new SPN on cell fate specification. Curr Biol *12*, R396-398.

Labbe, J.C., Pacquelet, A., Marty, T., and Gotta, M. (2006). A genomewide screen for suppressors of par-2 uncovers potential regulators of PAR protein-dependent cell polarity in Caenorhabditis elegans. Genetics *174*, 285-295.

Lakso, M., Vartiainen, S., Moilanen, A.M., Sirvio, J., Thomas, J.H., Nass, R., Blakely, R.D., and Wong, G. (2003). Dopaminergic neuronal loss and motor deficits in Caenorhabditis elegans overexpressing human alpha-synuclein. J Neurochem *86*, 165-172.

Lalanne, A.I., Britos, L., Ehrlich, R., and Castillo, E. (2004). Mesocestoides corti: a LIM-homeobox gene upregulated during strobilar development. Exp Parasitol *108*, 169-175.

Lall, S., Grun, D., Krek, A., Chen, K., Wang, Y.L., Dewey, C.N., Sood, P., Colombo, T., Bray, N., Macmenamin, P.*, et al.* (2006). A genome-wide map of conserved microRNA targets in C. elegans. Curr Biol *16*, 460-471.

Lam, N., Chesney, M.A., and Kimble, J. (2006). Wnt signaling and CEH-22/tinman/Nkx2.5 specify a stem cell niche in C. elegans. Curr Biol *16*, 287-295.

Lambie, E.J. (2002). Cell proliferation and growth in C. elegans. Bioessays *24*, 38-53.

Landmann, F., Quintin, S., and Labouesse, M. (2004). Multiple regulatory elements with spatially and temporally distinct activities control the expression of the epithelial differentiation gene lin-26 in C. elegans. Dev Biol *265*, 478-490.

Lanjuin, A., and Sengupta, P. (2004). Specification of chemosensory neuron subtype identities in Caenorhabditis elegans. Curr Opin Neurobiol *14*, 22-30.

Lanjuin, A., VanHoven, M.K., Bargmann, C.I., Thompson, J.K., and Sengupta, P. (2003). Otx/otd homeobox genes specify distinct sensory neuron identities in C. elegans. Dev Cell *5*, 621-633.

Lannoy, V.J., Bürglin, T.R., Rousseau, G.G., and Lemaigre, F.P. (1998). Isoforms of hepatocyte nuclear factor-6 differ in DNA-binding properties, contain a bifunctional homeodomain, and define the new ONECUT class of homeodomain proteins. J Biol Chem *273*, 13552-13562.

Lecroisey, C., Martin, E., Mariol, M.C., Granger, L., Schwab, Y., Labouesse, M., Segalat, L., and Gieseler, K. (2008). DYC-1, a protein functionally linked to dystrophin in Caenorhabditis elegans is associated with the dense body, where it interacts with the muscle LIM domain protein ZYX-1. Mol Biol Cell *19*, 785-796.

Lee, K., and Portman, D.S. (2007). Neural sex modifies the function of a C. elegans sensory circuit. Curr Biol *17*, 1858-1863.

Lee, K.Z., Eizinger, A., Nandakumar, R., Schuster, S.C., and Sommer, R.J. (2003). Limited microsynteny between the genomes of Pristionchus pacificus and Caenorhabditis elegans. Nucleic Acids Res *31*, 2553-2560.

Lee, M.H., and Schedl, T. (2006). RNA-binding proteins. WormBook, 1-13.

Lee, S.K., Li, W., Ryu, S.E., Rhim, T., and Ahnn, J. (2010). Vacuolar (H+)-ATPases in Caenorhabditis elegans: what can we learn about giant H+ pumps from tiny worms? Biochim Biophys Acta *1797*, 1687-1695.

Lei, H., Liu, J., Fukushige, T., Fire, A., and Krause, M. (2009). Caudal-like PAL-1 directly activates the bodywall muscle module regulator hlh-1 in C. elegans to initiate the embryonic muscle gene regulatory network. Development *136*, 1241-1249.

Lesch, B.J., and Bargmann, C.I. (2010). The homeodomain protein hmbx-1 maintains asymmetric gene expression in adult C. elegans olfactory neurons. Genes Dev *24*, 1802-1815.

Lesch, B.J., Gehrke, A.R., Bulyk, M.L., and Bargmann, C.I. (2009). Transcriptional regulation and stabilization of left-right neuronal identity in C. elegans. Genes Dev *23*, 345-358.

Lewis, J.A., and Hodgkin, J.A. (1977). Specific neuroanatomical changes in chemosensory mutants of the nematode Caenorhabditis elegans. J Comp Neurol *172*, 489-510.

Lewis, J.A., Wu, C.H., Levine, J.H., and Berg, H. (1980). Levamisole-resistant mutants of the nematode Caenorhabditis elegans appear to lack pharmacological acetylcholine receptors. Neuroscience *5*, 967-989.

Li, C., and Chalfie, M. (1990). Organogenesis in C. elegans: positioning of neurons and muscles in the egg-laying system. Neuron *4*, 681-695.

Li, P.M., Reichert, J., Freyd, G., Horvitz, H.R., and Walsh, C.T. (1991). The LIM region of a presumptive Caenorhabditis elegans transcription factor is an iron-sulfur- and zinc-containing metallodomain. Proc Natl Acad Sci U S A *88*, 9210-9213.

Li, W., Feng, Z., Sternberg, P.W., and Xu, X.Z. (2006). A C. elegans stretch receptor neuron revealed by a mechanosensitive TRP channel homologue. Nature *440*, 684-687.

Li, W., Herman, R.K., and Shaw, J.E. (1992). Analysis of the Caenorhabditis elegans axonal guidance and outgrowth gene unc-33. Genetics *132*, 675-689.

Li, X., Kulkarni, R.P., Hill, R.J., and Chamberlin, H.M. (2009). HOM-C genes, Wnt signaling and axial patterning in the C. elegans posterior ventral epidermis. Dev Biol *332*, 156-165.

Li, Y., Ye, H., Du, M., Zhang, Y., Ye, B., Pu, Y., and Wang, D. (2009). Induction of chemotaxis to sodium chloride and diacetyl and thermotaxis defects by microcystin-LR exposure in nematode Caenorhabditis elegans. J Environ Sci (China) *21*, 971-979.

Liang, B., Moussaif, M., Kuan, C.J., Gargus, J.J., and Sze, J.Y. (2006). Serotonin targets the DAF-16/FOXO signaling pathway to modulate stress responses. Cell Metab *4*, 429-440.

Lichtsteiner, S., and Tjian, R. (1995). Synergistic activation of transcription by UNC-86 and MEC-3 in Caenorhabditis elegans embryo extracts. Embo J *14*, 3937-3945.

Lickteig, K.M., Duerr, J.S., Frisby, D.L., Hall, D.H., Rand, J.B., and Miller, D.M., 3rd (2001). Regulation of neurotransmitter vesicles by the homeodomain protein UNC-4 and its transcriptional corepressor UNC-37/groucho in Caenorhabditis elegans cholinergic motor neurons. J Neurosci *21*, 2001-2014.

Lin, B., and Reinke, V. (2008). The candidate MAP kinase phosphorylation substrate DPL-1 (DP) promotes expression of the MAP kinase phosphatase LIP-1 in C. elegans germ cells. Dev Biol *316*, 50-61.

Link, C.D., Ehrenfels, C.W., and Wood, W.B. (1988). Mutant expression of male copulatory bursa surface markers in Caenorhabditis elegans. Development *103*, 485-495.

Lints, R., and Emmons, S.W. (1999). Patterning of dopaminergic neurotransmitter identity among Caenorhabditis elegans ray sensory neurons by a TGFbeta family signaling pathway and a Hox gene. Development *126*, 5819-5831.

Lints, R., and Emmons, S.W. (2002). Regulation of sex-specific differentiation and mating behavior in C. elegans by a new member of the DM domain transcription factor family. Genes Dev *16*, 2390-2402.

Lints, R., Jia, L., Kim, K., Li, C., and Emmons, S.W. (2004). Axial patterning of C. elegans male sensilla identities by selector genes. Dev Biol *269*, 137-151.

Liu, H., Strauss, T.J., Potts, M.B., and Cameron, S. (2006). Direct regulation of egl-1 and of programmed cell death by the Hox protein MAB-5 and by CEH-20, a C. elegans homolog of Pbx1. Development *133*, 641-650.

Liu, J., and Fire, A. (2000). Overlapping roles of two Hox genes and the exd ortholog ceh-20 in diversification of the C. elegans postembryonic mesoderm. Development *127*, 5179-5190.

Liu, T., Zimmerman, K.K., and Patterson, G.I. (2004). Regulation of signaling genes by TGFbeta during entry into dauer diapause in C. elegans. BMC Dev Biol *4*, 11.

Locke, C.J., Kautu, B.B., Berry, K.P., Lee, S.K., Caldwell, K.A., and Caldwell, G.A. (2009). Pharmacogenetic analysis reveals a post-developmental role for Rac GTPases in Caenorhabditis elegans GABAergic neurotransmission. Genetics *183*, 1357-1372.

Loer, C.M., and Kenyon, C.J. (1993). Serotonin-deficient mutants and male mating behavior in the nematode Caenorhabditis elegans. J Neurosci *13*, 5407-5417.

Louvet-Vallee, S., Kolotuev, I., Podbilewicz, B., and Felix, M.A. (2003). Control of vulval competence and centering in the nematode Oscheius sp. 1 CEW1. Genetics *163*, 133-146.

Lu, B., Jan, L.Y., and Jan, Y.N. (1998). Asymmetric cell division: lessons from flies and worms. Curr Opin Genet Dev *8*, 392-399.

Luo, J., Shah, S., Riabowol, K., and Mains, P.E. (2009). The Caenorhabditis elegans ing-3 gene regulates ionizing radiation-induced germ-cell apoptosis in a p53-associated pathway. Genetics *181*, 473-482.

Luo, L., Clark, D.A., Biron, D., Mahadevan, L., and Samuel, A.D. (2006). Sensorimotor control during isothermal tracking in Caenorhabditis elegans. J Exp Biol *209*, 4652-4662.

Luo, L., Gabel, C.V., Ha, H.I., Zhang, Y., and Samuel, A.D. (2008). Olfactory behavior of swimming C. elegans analyzed by measuring motile responses to temporal variations of odorants. J Neurophysiol *99*, 2617-2625.

MacColl, G., Bouloux, P., and Quinton, R. (2002). Kallmann syndrome: adhesion, afferents, and anosmia. Neuron *34*, 675-678.

Macrae, M., Plasterk, R.H., and Coffino, P. (1995). The ornithine decarboxylase gene of Caenorhabditis elegans: cloning, mapping and mutagenesis. Genetics *140*, 517-525.

Maduro, M.F., Kasmir, J.J., Zhu, J., and Rothman, J.H. (2005). The Wnt effector POP-1 and the PAL-1/Caudal homeoprotein collaborate with SKN-1 to activate C. elegans endoderm development. Dev Biol *285*, 510-523.

Maduro, M.F., and Rothman, J.H. (2002). Making worm guts: the gene regulatory network of the Caenorhabditis elegans endoderm. Dev Biol *246*, 68-85.

Mah, A.K., Armstrong, K.R., Chew, D.S., Chu, J.S., Tu, D.K., Johnsen, R.C., Chen, N., Chamberlin, H.M., and Baillie, D.L. (2007). Transcriptional regulation of AQP-8, a Caenorhabditis elegans aquaporin exclusively expressed in the excretory system, by the POU homeobox transcription factor CEH-6. J Biol Chem *282*, 28074-28086.

Mah, A.K., Tu, D.K., Johnsen, R.C., Chu, J.S., Chen, N., and Baillie, D.L. (2010). Characterization of the octamer, a cis-regulatory element that modulates excretory cell gene-expression in Caenorhabditis elegans. BMC Mol Biol *11*, 19.

Maloof, J.N., and Kenyon, C. (1998). The Hox gene lin-39 is required during C. elegans vulval induction to select the outcome of Ras signaling. Development *125*, 181-190.

Maloof, J.N., Whangbo, J., Harris, J.M., Jongeward, G.D., and Kenyon, C. (1999). A Wnt signaling pathway controls hox gene expression and neuroblast migration in C. elegans. Development *126*, 37-49.

Mango, S.E. (2007). The C. elegans pharynx: a model for organogenesis. WormBook, 1-26.

Manning, G. (2005). Genomic overview of protein kinases. WormBook, 1-19.

Manser, J., and Wood, W.B. (1990). Mutations affecting embryonic cell migrations in Caenorhabditis elegans. Dev Genet *11*, 49-64.

Marri, S., and Gupta, B.P. (2009). Dissection of lin-11 enhancer regions in Caenorhabditis elegans and other nematodes. Dev Biol *325*, 402-411.

Maurer, C.W., Chiorazzi, M., and Shaham, S. (2007). Timing of the onset of a developmental cell death is controlled by transcriptional induction of the C. elegans ced-3 caspase-encoding gene. Development *134*, 1357-1368.

Maydan, J.S., Flibotte, S., Edgley, M.L., Lau, J., Selzer, R.R., Richmond, T.A., Pofahl, N.J., Thomas, J.H., and Moerman, D.G. (2007). Efficient high-resolution deletion discovery in Caenorhabditis elegans by array comparative genomic hybridization. Genome Res *17*, 337-347.

McCarter, J., Bartlett, B., Dang, T., and Schedl, T. (1997). Soma-germ cell interactions in Caenorhabditis elegans: multiple events of hermaphrodite germline development require the somatic sheath and spermathecal lineages. Dev Biol *181*, 121-143.

McGhee, J.D., Sleumer, M.C., Bilenky, M., Wong, K., McKay, S.J., Goszczynski, B., Tian, H., Krich, N.D., Khattra, J., Holt, R.A.*, et al.* (2007). The ELT-2 GATA-factor and the global regulation of transcription in the C. elegans intestine. Dev Biol *302*, 627-645.

McIntire, S.L., Jorgensen, E., and Horvitz, H.R. (1993). Genes required for GABA function in Caenorhabditis elegans. Nature *364*, 334-337.

McIntire, S.L., Jorgensen, E., Kaplan, J., and Horvitz, H.R. (1993). The GABAergic nervous system of Caenorhabditis elegans. Nature *364*, 337-341.

McKim, K.S., Matheson, C., Marra, M.A., Wakarchuk, M.F., and Baillie, D.L. (1994). The Caenorhabditis elegans unc-60 gene encodes proteins homologous to a family of actin-binding proteins. Mol Gen Genet *242*, 346-357.

McKim, K.S., Peters, K., and Rose, A.M. (1993). Two types of sites required for meiotic chromosome pairing in Caenorhabditis elegans. Genetics *134*, 749-768.

Mehta, N., Loria, P.M., and Hobert, O. (2004). A genetic screen for neurite outgrowth mutants in Caenorhabditis elegans reveals a new function for the F-box ubiquitin ligase component LIN-23. Genetics *166*, 1253-1267.

Meighan, C.M., and Schwarzbauer, J.E. (2007). Control of C. elegans hermaphrodite gonad size and shape by vab-3/Pax6-mediated regulation of integrin receptors. Genes Dev *21*, 1615-1620.

Melkman, T., and Sengupta, P. (2004). The worm's sense of smell. Development of functional diversity in the chemosensory system of Caenorhabditis elegans. Dev Biol *265*, 302-319.

Melkman, T., and Sengupta, P. (2005). Regulation of chemosensory and GABAergic motor neuron development by the C. elegans Aristaless/Arx homolog alr-1. Development *132*, 1935-1949.

Merritt, C., Rasoloson, D., Ko, D., and Seydoux, G. (2008). 3' UTRs are the primary regulators of gene expression in the C. elegans germline. Curr Biol *18*, 1476-1482.

Meyer, J.N., Boyd, W.A., Azzam, G.A., Haugen, A.C., Freedman, J.H., and Van Houten, B. (2007). Decline of nucleotide excision repair capacity in aging Caenorhabditis elegans. Genome Biol *8*, R70.

Michaux, G., Legouis, R., and Labouesse, M. (2001). Epithelial biology: lessons from Caenorhabditis elegans. Gene *277*, 83-100.

Miller, D.M., 3rd, and Niemeyer, C.J. (1995). Expression of the unc-4 homeoprotein in Caenorhabditis elegans motor neurons specifies presynaptic input. Development *121*, 2877-2886.

Miller, D.M., 3rd, Niemeyer, C.J., and Chitkara, P. (1993). Dominant unc-37 mutations suppress the movement defect of a homeodomain mutation in unc-4, a neural specificity gene in Caenorhabditis elegans. Genetics *135*, 741-753.

Miller, D.M., Shen, M.M., Shamu, C.E., Bürglin, T.R., Ruvkun, G., Dubois, M.L., Ghee, M., and Wilson, L. (1992). C. elegans unc-4 gene encodes a homeodomain protein that determines the pattern of synaptic input to specific motor neurons. Nature *355*, 841-845.

Miller, L.M., Gallegos, M.E., Morisseau, B.A., and Kim, S.K. (1993). lin-31, a Caenorhabditis elegans HNF-3/fork head transcription factor homolog, specifies three alternative cell fates in vulval development. Genes Dev *7*, 933-947.

Miller, M.A., Ruest, P.J., Kosinski, M., Hanks, S.K., and Greenstein, D. (2003). An Eph receptor sperm-sensing control mechanism for oocyte meiotic maturation in Caenorhabditis elegans. Genes Dev *17*, 187-200.

Minasaki, R., and Streit, A. (2007). MEL-47, a novel protein required for early cell divisions in the nematode Caenorhabditis elegans. Mol Genet Genomics *277*, 315-328.

Mitani, S., Du, H., Hall, D.H., Driscoll, M., and Chalfie, M. (1993). Combinatorial control of touch receptor neuron expression in Caenorhabditis elegans. Development *119*, 773-783.

Miura, M., and Yuan, J. (1996). Mechanisms of programmed cell death in Caenorhabditis elegans and vertebrates. Curr Top Dev Biol *32*, 139-174.

Moghal, N., Garcia, L.R., Khan, L.A., Iwasaki, K., and Sternberg, P.W. (2003). Modulation of EGF receptor-mediated vulva development by the heterotrimeric G-protein Galphaq and excitable cells in C. elegans. Development *130*, 4553-4566.

Moghal, N., and Sternberg, P.W. (2003). The epidermal growth factor system in Caenorhabditis elegans. Exp Cell Res *284*, 150-159.

Montell, D.J. (1999). The genetics of cell migration in Drosophila melanogaster and Caenorhabditis elegans development. Development *126*, 3035-3046.

Mootz, D., Ho, D.M., and Hunter, C.P. (2004). The STAR/Maxi-KH domain protein GLD-1 mediates a developmental switch in the translational control of C. elegans PAL-1. Development *131*, 3263-3272.

Morck, C., Axang, C., Goksor, M., and Pilon, M. (2006). Misexpression of acetylcholinesterases in the C. elegans pha-2 mutant accompanies ultrastructural defects in pharyngeal muscle cells. Dev Biol *297*, 446-460.

Morck, C., Axang, C., and Pilon, M. (2003). A genetic analysis of axon guidance in the C elegans pharynx. Dev Biol *260*, 158-175.

Morck, C., and Pilon, M. (2006). C. elegans feeding defective mutants have shorter body lengths and increased autophagy. BMC Dev Biol *6*, 39.

Morck, C., Rauthan, M., Wagberg, F., and Pilon, M. (2004). pha-2 encodes the C. elegans ortholog of the homeodomain protein HEX and is required for the formation of the pharyngeal isthmus. Dev Biol *272*, 403-418.

Mori, I. (1999). Genetics of chemotaxis and thermotaxis in the nematode Caenorhabditis elegans. Annu Rev Genet *33*, 399-422.

Mori, I., and Ohshima, Y. (1995). Neural regulation of thermotaxis in Caenorhabditis elegans. Nature *376*, 344-348.

Mori, I., and Ohshima, Y. (1997). Molecular neurogenetics of chemotaxis and thermotaxis in the nematode Caenorhabditis elegans. Bioessays *19*, 1055-1064.

Moss, E.G., Lee, R.C., and Ambros, V. (1997). The cold shock domain protein LIN-28 controls developmental timing in C. elegans and is regulated by the lin-4 RNA. Cell *88*, 637-646.

Much, J.W., Slade, D.J., Klampert, K., Garriga, G., and Wightman, B. (2000). The fax-1 nuclear hormone receptor regulates axon pathfinding and neurotransmitter expression. Development *127*, 703-712.

Muimo, R., and Isaac, R.E. (1993). Metabolism of 5-HT in the nematode Caenorhabditis elegans: identification of a 5-HT N-acetyltransferase that is developmentally regulated. Biochem Soc Trans *21 ( Pt 3)*, 295S.

Mukherjee, K., and Bürglin, T.R. (2007). Comprehensive analysis of animal TALE homeobox genes: new conserved motifs and cases of accelerated evolution. J Mol Evol *65*, 137-153.

Mukhopadhyay, S., Lu, Y., Qin, H., Lanjuin, A., Shaham, S., and Sengupta, P. (2007). Distinct IFT mechanisms contribute to the generation of ciliary structural diversity in C. elegans. Embo J *26*, 2966-2980.

Muzzini, D.M., Plevani, P., Boulton, S.J., Cassata, G., and Marini, F. (2008). Caenorhabditis elegans POLQ-1 and HEL-308 function in two distinct DNA interstrand cross-link repair pathways. DNA Repair (Amst) *7*, 941-950.

Naito, M., Kohara, Y., and Kurosawa, Y. (1992). Identification of a homeobox-containing gene located between lin-45 and unc-24 on chromosome IV in the nematode Caenorhabditis elegans. Nucleic Acids Res *20*, 2967-2969.

Nass, R., Hall, D.H., Miller, D.M., 3rd, and Blakely, R.D. (2002). Neurotoxin-induced degeneration of dopamine neurons in Caenorhabditis elegans. Proc Natl Acad Sci U S A *99*, 3264-3269.

Nehme, R., Grote, P., Tomasi, T., Loser, S., Holzkamp, H., Schnabel, R., and Conradt, B. (2010). Transcriptional upregulation of both egl-1 BH3-only and ced-3 caspase is required for the death of the male-specific CEM neurons. Cell Death Differ *17*, 1266-1276.

Nelms, B.L., and Hanna-Rose, W. (2006). C. elegans HIM-8 functions outside of meiosis to antagonize EGL-13 Sox protein function. Dev Biol *293*, 392-402.

Nelson, C.E., Hersh, B.M., and Carroll, S.B. (2004). The regulatory content of intergenic DNA shapes genome architecture. Genome Biol *5*, R25.

Neves, A., English, K., and Priess, J.R. (2007). Notch-GATA synergy promotes endoderm-specific expression of ref-1 in C. elegans. Development *134*, 4459-4468.

Newman, A.P., Acton, G.Z., Hartwieg, E., Horvitz, H.R., and Sternberg, P.W. (1999). The lin-11 LIM domain transcription factor is necessary for morphogenesis of C. elegans uterine cells. Development *126*, 5319-5326.

Newman, A.P., Inoue, T., Wang, M., and Sternberg, P.W. (2000). The Caenorhabditis elegans heterochronic gene lin-29 coordinates the vulval-uterine-epidermal connections. Curr Biol *10*, 1479-1488.

Newman, A.P., and Sternberg, P.W. (1996). Coordinated morphogenesis of epithelia during development of the Caenorhabditis elegans uterine-vulval connection. Proc Natl Acad Sci U S A *93*, 9329-9333.

Newman-Smith, E.D., and Rothman, J.H. (1998). The maternal-to-zygotic transition in embryonic patterning of Caenorhabditis elegans. Curr Opin Genet Dev *8*, 472-480.

Nicholas, H.R., and Hodgkin, J. (2004). The ERK MAP kinase cascade mediates tail swelling and a protective response to rectal infection in C. elegans. Curr Biol *14*, 1256-1261.

Nicholas, H.R., and Hodgkin, J. (2009). The C. elegans Hox gene egl-5 is required for correct development of the hermaphrodite hindgut and for the response to rectal infection by Microbacterium nematophilum. Dev Biol *329*, 16-24.

Nilsson, L., Li, X., Tiensuu, T., Auty, R., Greenwald, I., and Tuck, S. (1998). Caenorhabditis elegans lin-25: cellular focus, protein expression and requirement for sur-2 during induction of vulval fates. Development *125*, 4809-4819.

Nilsson, L., Tiensuu, T., and Tuck, S. (2000). Caenorhabditis elegans lin-25: a study of its role in multiple cell fate specification events involving Ras and the identification and characterization of evolutionarily conserved domains. Genetics *156*, 1083-1096.

Nishiwaki, K. (1999). Mutations affecting symmetrical migration of distal tip cells in Caenorhabditis elegans. Genetics *152*, 985-997.

Nonet, M.L. (1999). Visualization of synaptic specializations in live C. elegans with synaptic vesicle protein-GFP fusions. J Neurosci Methods *89*, 33-40.

O'Meara, M.M., Bigelow, H., Flibotte, S., Etchberger, J.F., Moerman, D.G., and Hobert, O. (2009). Cis-regulatory mutations in the Caenorhabditis elegans homeobox gene locus cog-1 affect neuronal development. Genetics *181*, 1679-1686.

Ogura, K., Kishimoto, N., Mitani, S., Gengyo-Ando, K., and Kohara, Y. (2003). Translational control of maternal glp-1 mRNA by POS-1 and its interacting protein SPN-4 in Caenorhabditis elegans. Development *130*, 2495-2503.

Okkema, P.G., and Fire, A. (1994). The Caenorhabditis elegans NK-2 class homeoprotein CEH-22 is involved in combinatorial activation of gene expression in pharyngeal muscle. Development *120*, 2175-2186.

Okkema, P.G., Ha, E., Haun, C., Chen, W., and Fire, A. (1997). The Caenorhabditis elegans NK-2 homeobox gene ceh-22 activates pharyngeal muscle gene expression in combination with pha-1 and is required for normal pharyngeal development. Development *124*, 3965-3973.

Okkema, P.G., and Krause, M. (2005). Transcriptional regulation. WormBook, 1-40.

Okochi, Y., Kimura, K.D., Ohta, A., and Mori, I. (2005). Diverse regulation of sensory signaling by C. elegans nPKC-epsilon/eta TTX-4. Embo J *24*, 2127-2137.

Oommen, K.S., and Newman, A.P. (2007). Co-regulation by Notch and Fos is required for cell fate specification of intermediate precursors during C. elegans uterine development. Development *134*, 3999-4009.

Oosterveen, T., Coudreuse, D.Y., Yang, P.T., Fraser, E., Bergsma, J., Dale, T.C., and Korswagen, H.C. (2007). Two functionally distinct Axin-like proteins regulate canonical Wnt signaling in C. elegans. Dev Biol *308*, 438-448.

Ortiz, C.O., Etchberger, J.F., Posy, S.L., Frokjaer-Jensen, C., Lockery, S., Honig, B., and Hobert, O. (2006). Searching for neuronal left/right asymmetry: genomewide analysis of nematode receptor-type guanylyl cyclases. Genetics *173*, 131-149.

Owraghi, M., Broitman-Maduro, G., Luu, T., Roberson, H., and Maduro, M.F. (2010). Roles of the Wnt effector POP-1/TCF in the C. elegans endomesoderm specification gene network. Dev Biol *340*, 209-221.

Pagano, J.M., Farley, B.M., Essien, K.I., and Ryder, S.P. (2009). RNA recognition by the embryonic cell fate determinant and germline totipotency factor MEX-3. Proc Natl Acad Sci U S A *106*, 20252-20257.

Palmer, R.E., Inoue, T., Sherwood, D.R., Jiang, L.I., and Sternberg, P.W. (2002). Caenorhabditis elegans cog-1 locus encodes GTX/Nkx6.1 homeodomain proteins and regulates multiple aspects of reproductive system development. Dev Biol *252*, 202-213.

Pan, C.L., Baum, P.D., Gu, M., Jorgensen, E.M., Clark, S.G., and Garriga, G. (2008). C. elegans AP-2 and retromer control Wnt signaling by regulating mig-14/Wntless. Dev Cell *14*, 132-139.

Park, D., Jia, H., Rajakumar, V., and Chamberlin, H.M. (2006). Pax2/5/8 proteins promote cell survival in C. elegans. Development *133*, 4193-4202.

Parry, D.H., Xu, J., and Ruvkun, G. (2007). A whole-genome RNAi Screen for C. elegans miRNA pathway genes. Curr Biol *17*, 2013-2022.

Peden, E., Kimberly, E., Gengyo-Ando, K., Mitani, S., and Xue, D. (2007). Control of sex-specific apoptosis in C. elegans by the BarH homeodomain protein CEH-30 and the transcriptional repressor UNC-37/Groucho. Genes Dev *21*, 3195-3207.

Perkins, L.A., Hedgecock, E.M., Thomson, J.N., and Culotti, J.G. (1986). Mutant sensory cilia in the nematode Caenorhabditis elegans. Dev Biol *117*, 456-487.

Perry, M.D., Li, W., Trent, C., Robertson, B., Fire, A., Hageman, J.M., and Wood, W.B. (1993). Molecular characterization of the her-1 gene suggests a direct role in cell signaling during Caenorhabditis elegans sex determination. Genes Dev *7*, 216-228.

Pflugrad, A., Meir, J.Y., Barnes, T.M., and Miller, D.M., 3rd (1997). The Groucho-like transcription factor UNC-37 functions with the neural specificity gene unc-4 to govern motor neuron identity in C. elegans. Development *124*, 1699-1709.

Phillips, B.T., Kidd, A.R., 3rd, King, R., Hardin, J., and Kimble, J. (2007). Reciprocal asymmetry of SYS-1/beta-catenin and POP-1/TCF controls asymmetric divisions in Caenorhabditis elegans. Proc Natl Acad Sci U S A *104*, 3231-3236.

Pierce-Shimomura, J.T., Faumont, S., Gaston, M.R., Pearson, B.J., and Lockery, S.R. (2001). The homeobox gene lim-6 is required for distinct chemosensory representations in C. elegans. Nature *410*, 694-698.

Pilon, M., and Morck, C. (2005). Development of Caenorhabditis elegans pharynx, with emphasis on its nervous system. Acta Pharmacol Sin *26*, 396-404.

Pires-daSilva, A., and Sommer, R.J. (2003). Finally, worm polycomb-like genes meet Hox regulation. Dev Cell *4*, 770-772.

Pocock, R., Ahringer, J., Mitsch, M., Maxwell, S., and Woollard, A. (2004). A regulatory network of T-box genes and the even-skipped homologue vab-7 controls patterning and morphogenesis in C. elegans. Development *131*, 2373-2385.

Podbilewicz, B. (2003). How does a cell anchor and invade an organ? Dev Cell *5*, 5-7.

Podbilewicz, B. (2006). Cell fusion. WormBook, 1-32.

Popovici, C., Berda, Y., Conchonaud, F., Harbis, A., Birnbaum, D., and Roubin, R. (2006). Direct and heterologous approaches to identify the LET-756/FGF interactome. BMC Genomics *7*, 105.

Portman, D.S., and Emmons, S.W. (2000). The basic helix-loop-helix transcription factors LIN-32 and HLH-2 function together in multiple steps of a C. elegans neuronal sublineage. Development *127*, 5415-5426.

Portman, D.S., and Emmons, S.W. (2004). Identification of C. elegans sensory ray genes using whole-genome expression profiling. Dev Biol *270*, 499-512.

Potts, M.B., Wang, D.P., and Cameron, S. (2009). Trithorax, Hox, and TALE-class homeodomain proteins ensure cell survival through repression of the BH3-only gene egl-1. Dev Biol *329*, 374-385.

Powell, J.R., Jow, M.M., and Meyer, B.J. (2005). The T-box transcription factor SEA-1 is an autosomal element of the X:A signal that determines C. elegans sex. Dev Cell *9*, 339-349.

Pradel, E., Zhang, Y., Pujol, N., Matsuyama, T., Bargmann, C.I., and Ewbank, J.J. (2007). Detection and avoidance of a natural product from the pathogenic bacterium Serratia marcescens by Caenorhabditis elegans. Proc Natl Acad Sci U S A *104*, 2295-2300.

Prahlad, V., Cornelius, T., and Morimoto, R.I. (2008). Regulation of the cellular heat shock response in Caenorhabditis elegans by thermosensory neurons. Science *320*, 811-814.

Prasad, B.C., Ye, B., Zackhary, R., Schrader, K., Seydoux, G., and Reed, R.R. (1998). unc-3, a gene required for axonal guidance in Caenorhabditis elegans, encodes a member of the O/E family of transcription factors. Development *125*, 1561-1568.

Priess, J.R. (2005). Notch signaling in the C. elegans embryo. WormBook, 1-16.

Pujol, N., Torregrossa, P., Ewbank, J.J., and Brunet, J.F. (2000). The homeodomain protein CePHOX2/CEH-17 controls antero-posterior axonal growth in C. elegans. Development *127*, 3361-3371.

Qin, H., and Powell-Coffman, J.A. (2004). The Caenorhabditis elegans aryl hydrocarbon receptor, AHR-1, regulates neuronal development. Dev Biol *270*, 64-75.

Raharjo, W.H., Logan, B.C., Wen, S., Kalb, J.M., and Gaudet, J. (2010). In vitro and in vivo characterization of Caenorhabditis elegans PHA-4/FoxA response elements. Dev Dyn *239*, 2219-2232.

Rajakumar, V., and Chamberlin, H.M. (2007). The Pax2/5/8 gene egl-38 coordinates organogenesis of the C. elegans egg-laying system. Dev Biol *301*, 240-253.

Rand, J.B., Duerr, J.S., and Frisby, D.L. (2000). Neurogenetics of vesicular transporters in C. elegans. Faseb J *14*, 2414-2422.

Rasmussen, J.P., English, K., Tenlen, J.R., and Priess, J.R. (2008). Notch signaling and morphogenesis of single-cell tubes in the C. elegans digestive tract. Dev Cell *14*, 559-569.

Rauthan, M., Morck, C., and Pilon, M. (2007). The C. elegans M3 neuron guides the growth cone of its sister cell M2 via the Kruppel-like zinc finger protein MNM-2. Dev Biol *311*, 185-199.

Ray, P., Schnabel, R., and Okkema, P.G. (2008). Behavioral and synaptic defects in C. elegans lacking the NK-2 homeobox gene ceh-28. Dev Neurobiol *68*, 421-433.

Reece-Hoyes, J.S., Deplancke, B., Barrasa, M.I., Hatzold, J., Smit, R.B., Arda, H.E., Pope, P.A., Gaudet, J., Conradt, B., and Walhout, A.J. (2009). The C. elegans Snail homolog CES-1 can activate gene expression in vivo and share targets with bHLH transcription factors. Nucleic Acids Res *37*, 3689-3698.

Reece-Hoyes, J.S., Shingles, J., Dupuy, D., Grove, C.A., Walhout, A.J., Vidal, M., and Hope, I.A. (2007). Insight into transcription factor gene duplication from Caenorhabditis elegans Promoterome-driven expression patterns. BMC Genomics *8*, 27.

Reiner, D.J., Ailion, M., Thomas, J.H., and Meyer, B.J. (2008). C. elegans anaplastic lymphoma kinase ortholog SCD-2 controls dauer formation by modulating TGF-beta signaling. Curr Biol *18*, 1101-1109.

Reiner, D.J., and Thomas, J.H. (1995). Reversal of a muscle response to GABA during C. elegans male development. J Neurosci *15*, 6094-6102.

Reinke, V. (2002). Functional exploration of the C. elegans genome using DNA microarrays. Nat Genet *32 Suppl*, 541-546.

Remy, J.J., and Hobert, O. (2005). An interneuronal chemoreceptor required for olfactory imprinting in C. elegans. Science *309*, 787-790.

Rhoads, R.E., Dinkova, T.D., and Korneeva, N.L. (2006). Mechanism and regulation of translation in C. elegans. WormBook, 1-18.

Riddle, D.L., and Brenner, S. (1978). Indirect suppression in Caenorhabditis elegans. Genetics *89*, 299-314.

Ririe, T.O., Fernandes, J.S., and Sternberg, P.W. (2008). The Caenorhabditis elegans vulva: a post-embryonic gene regulatory network controlling organogenesis. Proc Natl Acad Sci U S A *105*, 20095-20099.

Rockelein, I., Rohrig, S., Donhauser, R., Eimer, S., and Baumeister, R. (2000). Identification of amino acid residues in the Caenorhabditis elegans POU protein UNC-86 that mediate UNC-86-MEC-3-DNA ternary complex formation. Mol Cell Biol *20*, 4806-4813.

Roehl, H., and Kimble, J. (1993). Control of cell fate in C. elegans by a GLP-1 peptide consisting primarily of ankyrin repeats. Nature *364*, 632-635.

Rogalski, T.M., Moerman, D.G., and Baillie, D.L. (1982). Essential genes and deficiencies in the unc-22 IV region of Caenorhabditis elegans. Genetics *102*, 725-736.

Rohrig, S., Rockelein, I., Donhauser, R., and Baumeister, R. (2000). Protein interaction surface of the POU transcription factor UNC-86 selectively used in touch neurons. Embo J *19*, 3694-3703.

Rongo, C., Whitfield, C.W., Rodal, A., Kim, S.K., and Kaplan, J.M. (1998). LIN-10 is a shared component of the polarized protein localization pathways in neurons and epithelia. Cell *94*, 751-759.

Rose, A.M., and Baillie, D.L. (1979). A mutation in Caenorhabditis elegans that increases recombination frequency more than threefold. Nature *281*, 599-600.

Rose, K.L., Winfrey, V.P., Hoffman, L.H., Hall, D.H., Furuta, T., and Greenstein, D. (1997). The POU gene ceh-18 promotes gonadal sheath cell differentiation and function required for meiotic maturation and ovulation in Caenorhabditis elegans. Dev Biol *192*, 59-77.

Rosenbluth, R.E., and Baillie, D.L. (1981). The genetic analysis of a reciprocal translocation, eT1(III; V), in Caenorhabditis elegans. Genetics *99*, 415-428.

Rosenbluth, R.E., Cuddeford, C., and Baillie, D.L. (1985). Mutagenesis in Caenorhabditis elegans. II. A spectrum of mutational events induced with 1500 r of gamma-radiation. Genetics *109*, 493-511.

Ross, J.M., Kalis, A.K., Murphy, M.W., and Zarkower, D. (2005). The DM domain protein MAB-3 promotes sex-specific neurogenesis in C. elegans by regulating bHLH proteins. Dev Cell *8*, 881-892.

Ross, J.M., and Zarkower, D. (2003). Polycomb group regulation of Hox gene expression in C. elegans. Dev Cell *4*, 891-901.

Roy Chowdhuri, S., Crum, T., Woollard, A., Aslam, S., and Okkema, P.G. (2006). The T-box factor TBX-2 and the SUMO conjugating enzyme UBC-9 are required for ABa-derived pharyngeal muscle in C. elegans. Dev Biol *295*, 664-677.

Run, J.Q., Steven, R., Hung, M.S., van Weeghel, R., Culotti, J.G., and Way, J.C. (1996). Suppressors of the unc-73 gene of Caenorhabditis elegans. Genetics *143*, 225-236.

Ruvinsky, I., and Ruvkun, G. (2003). Functional tests of enhancer conservation between distantly related species. Development *130*, 5133-5142.

Ruvkun, G., and Hobert, O. (1998). The taxonomy of developmental control in Caenorhabditis elegans. Science *282*, 2033-2041.

Ryder, S.P., Frater, L.A., Abramovitz, D.L., Goodwin, E.B., and Williamson, J.R. (2004). RNA target specificity of the STAR/GSG domain post-transcriptional regulatory protein GLD-1. Nat Struct Mol Biol *11*, 20-28.

Ryu, W.S., and Samuel, A.D. (2002). Thermotaxis in Caenorhabditis elegans analyzed by measuring responses to defined Thermal stimuli. J Neurosci *22*, 5727-5733.

Sagasti, A., Hobert, O., Troemel, E.R., Ruvkun, G., and Bargmann, C.I. (1999). Alternative olfactory neuron fates are specified by the LIM homeobox gene lim-4. Genes Dev *13*, 1794-1806.

Salser, S.J., and Kenyon, C. (1992). Activation of a C. elegans Antennapedia homologue in migrating cells controls their direction of migration. Nature *355*, 255-258.

Salser, S.J., and Kenyon, C. (1994). Patterning C. elegans: homeotic cluster genes, cell fates and cell migrations. Trends Genet *10*, 159-164.

Salser, S.J., and Kenyon, C. (1996). A C. elegans Hox gene switches on, off, on and off again to regulate proliferation, differentiation and morphogenesis. Development *122*, 1651-1661.

Salser, S.J., Loer, C.M., and Kenyon, C. (1993). Multiple HOM-C gene interactions specify cell fates in the nematode central nervous system. Genes Dev *7*, 1714-1724.

Sarafi-Reinach, T.R., Melkman, T., Hobert, O., and Sengupta, P. (2001). The lin-11 LIM homeobox gene specifies olfactory and chemosensory neuron fates in C. elegans. Development *128*, 3269-3281.

Sarafi-Reinach, T.R., and Sengupta, P. (2000). The forkhead domain gene unc-130 generates chemosensory neuron diversity in C. elegans. Genes Dev *14*, 2472-2485.

Sarin, S., Antonio, C., Tursun, B., and Hobert, O. (2009). The C. elegans Tailless/TLX transcription factor nhr-67 controls neuronal identity and left/right asymmetric fate diversification. Development *136*, 2933-2944.

Sarin, S., O'Meara, M.M., Flowers, E.B., Antonio, C., Poole, R.J., Didiano, D., Johnston, R.J., Jr., Chang, S., Narula, S., and Hobert, O. (2007). Genetic screens for Caenorhabditis elegans mutants defective in left/right asymmetric neuronal fate specification. Genetics *176*, 2109-2130.

Satterlee, J.S., Ryu, W.S., and Sengupta, P. (2004). The CMK-1 CaMKI and the TAX-4 Cyclic nucleotide-gated channel regulate thermosensory neuron gene expression and function in C. elegans. Curr Biol *14*, 62-68.

Satterlee, J.S., Sasakura, H., Kuhara, A., Berkeley, M., Mori, I., and Sengupta, P. (2001). Specification of thermosensory neuron fate in C. elegans requires ttx-1, a homolog of otd/Otx. Neuron *31*, 943-956.

Savage-Dunn, C. (2005). TGF-beta signaling. WormBook, 1-12.

Schaller, D., Wittmann, C., Linning, R., Spicher, A., Muller, F., and Tobler, H. (1991). Cloning and expression in vitro of a gene encoding tRNAArgACG from the nematode Caenorhabditis elegans. Gene *97*, 273-276.

Schaller, D., Wittmann, C., Spicher, A., Muller, F., and Tobler, H. (1990). Cloning and analysis of three new homeobox genes from the nematode Caenorhabditis elegans. Nucleic Acids Res *18*, 2033-2036.

Schedin, P., Hunter, C.P., and Wood, W.B. (1991). Autonomy and nonautonomy of sex determination in triploid intersex mosaics of C. elegans. Development *112*, 863-879.

Schindelman, G., Whittaker, A.J., Thum, J.Y., Gharib, S., and Sternberg, P.W. (2006). Initiation of male sperm-transfer behavior in Caenorhabditis elegans requires input from the ventral nerve cord. BMC Biol *4*, 26.

Schlager, B., Roseler, W., Zheng, M., Gutierrez, A., and Sommer, R.J. (2006). HAIRY-like transcription factors and the evolution of the nematode vulva equivalence group. Curr Biol *16*, 1386-1394.

Schmid, C., Schwarz, V., and Hutter, H. (2006). AST-1, a novel ETS-box transcription factor, controls axon guidance and pharynx development in C. elegans. Dev Biol *293*, 403-413.

Schmitz, C., Kinge, P., and Hutter, H. (2007). Axon guidance genes identified in a large-scale RNAi screen using the RNAi-hypersensitive Caenorhabditis elegans strain nre-1(hd20) lin-15b(hd126). Proc Natl Acad Sci U S A *104*, 834-839.

Schmitz, C., Wacker, I., and Hutter, H. (2008). The Fat-like cadherin CDH-4 controls axon fasciculation, cell migration and hypodermis and pharynx development in Caenorhabditis elegans. Dev Biol *316*, 249-259.

Schnabel, H., Bauer, G., and Schnabel, R. (1991). Suppressors of the organ-specific differentiation gene pha-1 of Caenorhabditis elegans. Genetics *129*, 69-77.

Schnabel, H., and Schnabel, R. (1990). An Organ-Specific Differentiation Gene, pha-1, from Caenorhabditis elegans. Science *250*, 686-688.

Schnabel, R., and Schnabel, H. (1997). Hox genes misled by local environments. Nature *385*, 588-589.

Schroeder, D.F., and McGhee, J.D. (1998). Anterior-posterior patterning within the Caenorhabditis elegans endoderm. Development *125*, 4877-4887.

Schubert, C.M., Lin, R., de Vries, C.J., Plasterk, R.H., and Priess, J.R. (2000). MEX-5 and MEX-6 function to establish soma/germline asymmetry in early C. elegans embryos. Mol Cell *5*, 671-682.

Schuske, K., Beg, A.A., and Jorgensen, E.M. (2004). The GABA nervous system in C. elegans. Trends Neurosci *27*, 407-414.

Schwartz, H.T., and Horvitz, H.R. (2007). The C. elegans protein CEH-30 protects male-specific neurons from apoptosis independently of the Bcl-2 homolog CED-9. Genes Dev *21*, 3181-3194.

Seah, A., and Sternberg, P.W. (2009). The roles of EGF and Wnt signaling during patterning of the C. elegans Bgamma/delta Equivalence Group. BMC Dev Biol *9*, 74.

Segalat, L., and Neri, C. (2003). [C. elegans as a model for human inherited degenerative diseases]. Med Sci (Paris) *19*, 1218-1225.

Sengupta, P., and Bargmann, C.I. (1996). Cell fate specification and differentiation in the nervous system of Caenorhabditis elegans. Dev Genet *18*, 73-80.

Sewell, S.T., Zhang, G., Uttam, A., and Chamberlin, H.M. (2003). Developmental patterning in the Caenorhabditis elegans hindgut. Dev Biol *262*, 88-93.

Seydoux, G. (1996). Mechanisms of translational control in early development. Curr Opin Genet Dev *6*, 555-561.

Seydoux, G., Savage, C., and Greenwald, I. (1993). Isolation and characterization of mutations causing abnormal eversion of the vulva in Caenorhabditis elegans. Dev Biol *157*, 423-436.

Seydoux, G., and Schedl, T. (2001). The germline in C. elegans: origins, proliferation, and silencing. Int Rev Cytol *203*, 139-185.

Sha, K., and Fire, A. (2005). Imprinting capacity of gamete lineages in Caenorhabditis elegans. Genetics *170*, 1633-1652.

Shackleford, G.M., Shivakumar, S., Shiue, L., Mason, J., Kenyon, C., and Varmus, H.E. (1993). Two wnt genes in Caenorhabditis elegans. Oncogene *8*, 1857-1864.

Shaham, S., and Bargmann, C.I. (2002). Control of neuronal subtype identity by the C. elegans ARID protein CFI-1. Genes Dev *16*, 972-983.

Shaham, S., and Horvitz, H.R. (1996). Developing Caenorhabditis elegans neurons may contain both cell-death protective and killer activities. Genes Dev *10*, 578-591.

Shakir, M.A., Gill, J.S., and Lundquist, E.A. (2006). Interactions of UNC-34 Enabled with Rac GTPases and the NIK kinase MIG-15 in Caenorhabditis elegans axon pathfinding and neuronal migration. Genetics *172*, 893-913.

Shan, G., Kim, K., Li, C., and Walthall, W.W. (2005). Convergent genetic programs regulate similarities and differences between related motor neuron classes in Caenorhabditis elegans. Dev Biol *280*, 494-503.

Shan, G., and Walthall, W.W. (2008). Copulation in C. elegans males requires a nuclear hormone receptor. Dev Biol *322*, 11-20.

Shemer, G., Kishore, R., and Podbilewicz, B. (2000). Ring formation drives invagination of the vulva in Caenorhabditis elegans: Ras, cell fusion, and cell migration determine structural fates. Dev Biol *221*, 233-248.

Shemer, G., and Podbilewicz, B. (2000). Fusomorphogenesis: cell fusion in organ formation. Dev Dyn *218*, 30-51.

Shen, K., Fetter, R.D., and Bargmann, C.I. (2004). Synaptic specificity is generated by the synaptic guidepost protein SYG-2 and its receptor, SYG-1. Cell *116*, 869-881.

Shi, Y., and Mello, C. (1998). A CBP/p300 homolog specifies multiple differentiation pathways in Caenorhabditis elegans. Genes Dev *12*, 943-955.

Shibatohge, M., Kariya, K., Liao, Y., Hu, C.D., Watari, Y., Goshima, M., Shima, F., and Kataoka, T. (1998). Identification of PLC210, a Caenorhabditis elegans phospholipase C, as a putative effector of Ras. J Biol Chem *273*, 6218-6222.

Shtonda, B.B., and Avery, L. (2006). Dietary choice behavior in Caenorhabditis elegans. J Exp Biol *209*, 89-102.

Siddiqui, S.S. (1990). Mutations affecting axonal growth and guidance of motor neurons and mechanosensory neurons in the nematode Caenorhabditis elegans. Neurosci Res Suppl *13*, S171-190.

Siddiqui, S.S., and Culotti, J.G. (2007). Examination of neurons in wild type and mutants of Caenorhabditis elegans using antibodies to horseradish peroxidase. J Neurogenet *21*, 271-289.

Siegfried, K.R., Kidd, A.R., 3rd, Chesney, M.A., and Kimble, J. (2004). The sys-1 and sys-3 genes cooperate with Wnt signaling to establish the proximal-distal axis of the Caenorhabditis elegans gonad. Genetics *166*, 171-186.

Sigrist, C.B., and Sommer, R.J. (1999). Vulva formation in Pristionchus pacificus relies on continuous gonadal induction. Dev Genes Evol *209*, 451-459.

Sigurdson, D.C., Spanier, G.J., and Herman, R.K. (1984). Caenorhabditis elegans deficiency mapping. Genetics *108*, 331-345.

Silhankova, M., Jindra, M., and Asahina, M. (2005). Nuclear receptor NHR-25 is required for cell-shape dynamics during epidermal differentiation in Caenorhabditis elegans. J Cell Sci *118*, 223-232.

Simmer, F., Tijsterman, M., Parrish, S., Koushika, S.P., Nonet, M.L., Fire, A., Ahringer, J., and Plasterk, R.H. (2002). Loss of the putative RNA-directed RNA polymerase RRF-3 makes C. elegans hypersensitive to RNAi. Curr Biol *12*, 1317-1319.

Singhvi, A., Frank, C.A., and Garriga, G. (2008). The T-box gene tbx-2, the homeobox gene egl-5 and the asymmetric cell division gene ham-1 specify neural fate in the HSN/PHB lineage. Genetics *179*, 887-898.

Smith, C.J., Watson, J.D., Spencer, W.C., O'Brien, T., Cha, B., Albeg, A., Treinin, M., and Miller, D.M., 3rd (2010). Time-lapse imaging and cell-specific expression profiling reveal dynamic branching and molecular determinants of a multi-dendritic nociceptor in C. elegans. Dev Biol *345*, 18-33.

Smith, P.A., and Mango, S.E. (2007). Role of T-box gene tbx-2 for anterior foregut muscle development in C. elegans. Dev Biol *302*, 25-39.

Solari, F., Bateman, A., and Ahringer, J. (1999). The Caenorhabditis elegans genes egl-27 and egr-1 are similar to MTA1, a member of a chromatin regulatory complex, and are redundantly required for embryonic patterning. Development *126*, 2483-2494.

Sommer, R.J. (1997). Evolution and development--the nematode vulva as a case study. Bioessays *19*, 225-231.

Sommer, R.J. (2005). Evolution of development in nematodes related to C. elegans. WormBook, 1-17.

Sommer, R.J. (2006). Pristionchus pacificus. WormBook, 1-8.

Sommer, R.J., Carta, L.K., and Sternberg, P.W. (1994). The evolution of cell lineage in nematodes. Dev Suppl, 85-95.

Sommer, R.J., Eizinger, A., Lee, K.Z., Jungblut, B., Bubeck, A., and Schlak, I. (1998). The Pristionchus HOX gene Ppa-lin-39 inhibits programmed cell death to specify the vulva equivalence group and is not required during vulval induction. Development *125*, 3865-3873.

Stenico, M., Lloyd, A.T., and Sharp, P.M. (1994). Codon usage in Caenorhabditis elegans: delineation of translational selection and mutational biases. Nucleic Acids Res *22*, 2437-2446.

Sternberg, P.W. (1990). Genetic control of cell type and pattern formation in Caenorhabditis elegans. Adv Genet *27*, 63-116.

Sternberg, P.W. (1993). Intercellular signaling and signal transduction in C. elegans. Annu Rev Genet *27*, 497-521.

Sternberg, P.W. (2005). Vulval development. WormBook, 1-28.

Sternberg, P.W., and Felix, M.A. (1997). Evolution of cell lineage. Curr Opin Genet Dev *7*, 543-550.

Stewart, H.I., Rosenbluth, R.E., and Baillie, D.L. (1991). Most ultraviolet irradiation induced mutations in the nematode Caenorhabditis elegans are chromosomal rearrangements. Mutat Res *249*, 37-54.

Stoyanov, C.N., Fleischmann, M., Suzuki, Y., Tapparel, N., Gautron, F., Streit, A., Wood, W.B., and Muller, F. (2003). Expression of the C. elegans labial orthologue ceh-13 during male tail morphogenesis. Dev Biol *259*, 137-149.

Strange, K. (2003). From genes to integrative physiology: ion channel and transporter biology in Caenorhabditis elegans. Physiol Rev *83*, 377-415.

Streit, A., Kohler, R., Marty, T., Belfiore, M., Takacs-Vellai, K., Vigano, M.A., Schnabel, R., Affolter, M., and Muller, F. (2002). Conserved regulation of the Caenorhabditis elegans labial/Hox1 gene ceh-13. Dev Biol *242*, 96-108.

Stringham, E.G., and Candido, E.P. (1993). Targeted single-cell induction of gene products in Caenorhabditis elegans: a new tool for developmental studies. J Exp Zool *266*, 227-233.

Stroeher, V.L., Kennedy, B.P., Millen, K.J., Schroeder, D.F., Hawkins, M.G., Goszczynski, B., and McGhee, J.D. (1994). DNA-protein interactions in the Caenorhabditis elegans embryo: oocyte and embryonic factors that bind to the promoter of the gut-specific ges-1 gene. Dev Biol *163*, 367-380.

Struhl, G., Fitzgerald, K., and Greenwald, I. (1993). Intrinsic activity of the Lin-12 and Notch intracellular domains in vivo. Cell *74*, 331-345.

Su, C.W., Tharin, S., Jin, Y., Wightman, B., Spector, M., Meili, D., Tsung, N., Rhiner, C., Bourikas, D., Stoeckli, E.*, et al.* (2006). The short coiled-coil domain-containing protein UNC-69 cooperates with UNC-76 to regulate axonal outgrowth and normal presynaptic organization in Caenorhabditis elegans. J Biol *5*, 9.

Sulston, J.E. (1983). Neuronal cell lineages in the nematode Caenorhabditis elegans. Cold Spring Harb Symp Quant Biol *48 Pt 2*, 443-452.

Sulston, J.E., and Horvitz, H.R. (1981). Abnormal cell lineages in mutants of the nematode Caenorhabditis elegans. Dev Biol *82*, 41-55.

Sun, H., Nelms, B.L., Sleiman, S.F., Chamberlin, H.M., and Hanna-Rose, W. (2007). Modulation of Caenorhabditis elegans transcription factor activity by HIM-8 and the related Zinc-Finger ZIM proteins. Genetics *177*, 1221-1226.

Sundaram, M.V. (2006). RTK/Ras/MAPK signaling. WormBook, 1-19.

Suzuki, Y., and Han, M. (2006). Genetic redundancy masks diverse functions of the tumor suppressor gene PTEN during C. elegans development. Genes Dev *20*, 423-428.

Svendsen, P.C., and McGhee, J.D. (1995). The C. elegans neuronally expressed homeobox gene ceh-10 is closely related to genes expressed in the vertebrate eye. Development *121*, 1253-1262.

Swoboda, P., Adler, H.T., and Thomas, J.H. (2000). The RFX-type transcription factor DAF-19 regulates sensory neuron cilium formation in C. elegans. Mol Cell *5*, 411-421.

Sym, M., Robinson, N., and Kenyon, C. (1999). MIG-13 positions migrating cells along the anteroposterior body axis of C. elegans. Cell *98*, 25-36.

Szabo, E., Hargitai, B., Regos, A., Tihanyi, B., Barna, J., Borsos, E., Takacs-Vellai, K., and Vellai, T. (2009). TRA-1/GLI controls the expression of the Hox gene lin-39 during C. elegans vulval development. Dev Biol *330*, 339-348.

Sze, J.Y., Liu, Y., and Ruvkun, G. (1997). VP16-activation of the C. elegans neural specification transcription factor UNC-86 suppresses mutations in downstream genes and causes defects in neural migration and axon outgrowth. Development *124*, 1159-1168.

Sze, J.Y., and Ruvkun, G. (2003). Activity of the Caenorhabditis elegans UNC-86 POU transcription factor modulates olfactory sensitivity. Proc Natl Acad Sci U S A *100*, 9560-9565.

Sze, J.Y., Zhang, S., Li, J., and Ruvkun, G. (2002). The C. elegans POU-domain transcription factor UNC-86 regulates the tph-1 tryptophan hydroxylase gene and neurite outgrowth in specific serotonergic neurons. Development *129*, 3901-3911.

Takacs-Vellai, K., Vellai, T., Chen, E.B., Zhang, Y., Guerry, F., Stern, M.J., and Muller, F. (2007). Transcriptional control of Notch signaling by a HOX and a PBX/EXD protein during vulval development in C. elegans. Dev Biol *302*, 661-669.

Tan, P.L., Barr, T., Inglis, P.N., Mitsuma, N., Huang, S.M., Garcia-Gonzalez, M.A., Bradley, B.A., Coforio, S., Albrecht, P.J., Watnick, T.*, et al.* (2007). Loss of Bardet Biedl syndrome proteins causes defects in peripheral sensory innervation and function. Proc Natl Acad Sci U S A *104*, 17524-17529.

Tavernarakis, N., and Driscoll, M. (1997). Molecular modeling of mechanotransduction in the nematode Caenorhabditis elegans. Annu Rev Physiol *59*, 659-689.

Teng, Y., Girard, L., Ferreira, H.B., Sternberg, P.W., and Emmons, S.W. (2004). Dissection of cis-regulatory elements in the C. elegans Hox gene egl-5 promoter. Dev Biol *276*, 476-492.

Thoemke, K., Yi, W., Ross, J.M., Kim, S., Reinke, V., and Zarkower, D. (2005). Genome-wide analysis of sex-enriched gene expression during C. elegans larval development. Dev Biol *284*, 500-508.

Thomas, J.H., Birnby, D.A., and Vowels, J.J. (1993). Evidence for parallel processing of sensory information controlling dauer formation in Caenorhabditis elegans. Genetics *134*, 1105-1117.

Thor, S., and Thomas, J.B. (2002). Motor neuron specification in worms, flies and mice: conserved and 'lost' mechanisms. Curr Opin Genet Dev *12*, 558-564.

Tiensuu, T., Larsen, M.K., Vernersson, E., and Tuck, S. (2005). lin-1 has both positive and negative functions in specifying multiple cell fates induced by Ras/MAP kinase signaling in C. elegans. Dev Biol *286*, 338-351.

Tihanyi, B., Vellai, T., Regos, A., Ari, E., Muller, F., and Takacs-Vellai, K. (2010). The C. elegans Hox gene ceh-13 regulates cell migration and fusion in a non-colinear way. Implications for the early evolution of Hox clusters. BMC Dev Biol *10*, 78.

Toker, A.S., Teng, Y., Ferreira, H.B., Emmons, S.W., and Chalfie, M. (2003). The Caenorhabditis elegans spalt-like gene sem-4 restricts touch cell fate by repressing the selector Hox gene egl-5 and the effector gene mec-3. Development *130*, 3831-3840.

Toms, N., Cooper, J., Patchen, B., and Aamodt, E. (2001). High copy arrays containing a sequence upstream of mec-3 alter cell migration and axonal morphology in C. elegans. BMC Dev Biol *1*, 2.

Tong, Y.G., and Bürglin, T.R. (2010). Conditions for dye-filling of sensory neurons in Caenorhabditis elegans. J Neurosci Methods *188*, 58-61.

Torayama, I., Ishihara, T., and Katsura, I. (2007). Caenorhabditis elegans integrates the signals of butanone and food to enhance chemotaxis to butanone. J Neurosci *27*, 741-750.

Trachtulec, Z., and Forejt, J. (2001). Synteny of orthologous genes conserved in mammals, snake, fly, nematode, and fission yeast. Mamm Genome *12*, 227-231.

Trent, C., Tsuing, N., and Horvitz, H.R. (1983). Egg-laying defective mutants of the nematode Caenorhabditis elegans. Genetics *104*, 619-647.

Troemel, E.R., Sagasti, A., and Bargmann, C.I. (1999). Lateral signaling mediated by axon contact and calcium entry regulates asymmetric odorant receptor expression in C. elegans. Cell *99*, 387-398.

Tsalik, E.L., Niacaris, T., Wenick, A.S., Pau, K., Avery, L., and Hobert, O. (2003). LIM homeobox gene-dependent expression of biogenic amine receptors in restricted regions of the C. elegans nervous system. Dev Biol *263*, 81-102.

Tseng, R.J., Armstrong, K.R., Wang, X., and Chamberlin, H.M. (2007). The bromodomain protein LEX-1 acts with TAM-1 to modulate gene expression in C. elegans. Mol Genet Genomics *278*, 507-518.

Tsunozaki, M., Chalasani, S.H., and Bargmann, C.I. (2008). A behavioral switch: cGMP and PKC signaling in olfactory neurons reverses odor preference in C. elegans. Neuron *59*, 959-971.

Tucker, M., Sieber, M., Morphew, M., and Han, M. (2005). The Caenorhabditis elegans aristaless orthologue, alr-1, is required for maintaining the functional and structural integrity of the amphid sensory organs. Mol Biol Cell *16*, 4695-4704.

Uchida, O., Nakano, H., Koga, M., and Ohshima, Y. (2003). The C. elegans che-1 gene encodes a zinc finger transcription factor required for specification of the ASE chemosensory neurons. Development *130*, 1215-1224.

Updike, D.L., and Mango, S.E. (2006). Temporal regulation of foregut development by HTZ-1/H2A.Z and PHA-4/FoxA. PLoS Genet *2*, e161.

Updike, D.L., and Strome, S. (2009). A genomewide RNAi screen for genes that affect the stability, distribution and function of P granules in Caenorhabditis elegans. Genetics *183*, 1397-1419.

Van Auken, K., Weaver, D., Robertson, B., Sundaram, M., Saldi, T., Edgar, L., Elling, U., Lee, M., Boese, Q., and Wood, W.B. (2002). Roles of the Homothorax/Meis/Prep homolog UNC-62 and the Exd/Pbx homologs CEH-20 and CEH-40 in C. elegans embryogenesis. Development *129*, 5255-5268.

Van Auken, K., Weaver, D.C., Edgar, L.G., and Wood, W.B. (2000). Caenorhabditis elegans embryonic axial patterning requires two recently discovered posterior-group Hox genes. Proc Natl Acad Sci U S A *97*, 4499-4503.

Van Buskirk, C., and Sternberg, P.W. (2007). Epidermal growth factor signaling induces behavioral quiescence in Caenorhabditis elegans. Nat Neurosci *10*, 1300-1307.

Van Buskirk, C., and Sternberg, P.W. (2010). Paired and LIM class homeodomain proteins coordinate differentiation of the C. elegans ALA neuron. Development *137*, 2065-2074.

van der Linden, A.M., Nolan, K.M., and Sengupta, P. (2007). KIN-29 SIK regulates chemoreceptor gene expression via an MEF2 transcription factor and a class II HDAC. Embo J *26*, 358-370.

Van Hoffelen, S., and Herman, M.A. (2008). Analysis of Wnt signaling during Caenorhabditis elegans postembryonic development. Methods Mol Biol *469*, 87-102.

Varkey, J.P., Muhlrad, P.J., Minniti, A.N., Do, B., and Ward, S. (1995). The Caenorhabditis elegans spe-26 gene is necessary to form spermatids and encodes a protein similar to the actin-associated proteins kelch and scruin. Genes Dev *9*, 1074-1086.

Vastenhouw, N.L., Brunschwig, K., Okihara, K.L., Muller, F., Tijsterman, M., and Plasterk, R.H. (2006). Gene expression: long-term gene silencing by RNAi. Nature *442*, 882.

Vasudevan, S., Starostina, N.G., and Kipreos, E.T. (2007). The Caenorhabditis elegans cell-cycle regulator ZYG-11 defines a conserved family of CUL-2 complex components. EMBO Rep *8*, 279-286.

Vavouri, T., Semple, J.I., Garcia-Verdugo, R., and Lehner, B. (2009). Intrinsic protein disorder and interaction promiscuity are widely associated with dosage sensitivity. Cell *138*, 198-208.

Vella, M.C., and Slack, F.J. (2005). C. elegans microRNAs. WormBook, 1-9.

Venkatesan, K., McManus, H.R., Mello, C.C., Smith, T.F., and Hansen, U. (2003). Functional conservation between members of an ancient duplicated transcription factor family, LSF/Grainyhead. Nucleic Acids Res *31*, 4304-4316.

Vermeirssen, V., Barrasa, M.I., Hidalgo, C.A., Babon, J.A., Sequerra, R., Doucette-Stamm, L., Barabasi, A.L., and Walhout, A.J. (2007). Transcription factor modularity in a gene-centered C. elegans core neuronal protein-DNA interaction network. Genome Res *17*, 1061-1071.

Vilimas, T., Abraham, A., and Okkema, P.G. (2004). An early pharyngeal muscle enhancer from the Caenorhabditis elegans ceh-22 gene is targeted by the Forkhead factor PHA-4. Dev Biol *266*, 388-398.

Von Stetina, S.E., Fox, R.M., Watkins, K.L., Starich, T.A., Shaw, J.E., and Miller, D.M., 3rd (2007). UNC-4 represses CEH-12/HB9 to specify synaptic inputs to VA motor neurons in C. elegans. Genes Dev *21*, 332-346.

Voutev, R., Keating, R., Hubbard, E.J., and Vallier, L.G. (2009). Characterization of the Caenorhabditis elegans Islet LIM-homeodomain ortholog, lim-7. FEBS Lett *583*, 456-464.

Wacker, I., Schwarz, V., Hedgecock, E.M., and Hutter, H. (2003). zag-1, a Zn-finger homeodomain transcription factor controlling neuronal differentiation and axon outgrowth in C. elegans. Development *130*, 3795-3805.

Waddle, J.A., Cooper, J.A., and Waterston, R.H. (1993). The alpha and beta subunits of nematode actin capping protein function in yeast. Mol Biol Cell *4*, 907-917.

Wadsworth, W.G. (2005). Axon pruning: C. elegans makes the cut. Curr Biol *15*, R796-798.

Wadsworth, W.G., and Hedgecock, E.M. (1992). Guidance of neuroblast migrations and axonal projections in Caenorhabditis elegans. Curr Opin Neurobiol *2*, 36-41.

Waggoner, L.E., Dickinson, K.A., Poole, D.S., Tabuse, Y., Miwa, J., and Schafer, W.R. (2000). Long-term nicotine adaptation in Caenorhabditis elegans involves PKC-dependent changes in nicotinic receptor abundance. J Neurosci *20*, 8802-8811.

Wagmaister, J.A., Gleason, J.E., and Eisenmann, D.M. (2006). Transcriptional upregulation of the C. elegans Hox gene lin-39 during vulval cell fate specification. Mech Dev *123*, 135-150.

Wagmaister, J.A., Miley, G.R., Morris, C.A., Gleason, J.E., Miller, L.M., Kornfeld, K., and Eisenmann, D.M. (2006). Identification of cis-regulatory elements from the C. elegans Hox gene lin-39 required for embryonic expression and for regulation by the transcription factors LIN-1, LIN-31 and LIN-39. Dev Biol *297*, 550-565.

Wakabayashi, T., Kitagawa, I., and Shingai, R. (2004). Neurons regulating the duration of forward locomotion in Caenorhabditis elegans. Neurosci Res *50*, 103-111.

Walhout, A.J., Sordella, R., Lu, X., Hartley, J.L., Temple, G.F., Brasch, M.A., Thierry-Mieg, N., and Vidal, M. (2000). Protein interaction mapping in C. elegans using proteins involved in vulval development. Science *287*, 116-122.

Walker, A.K., Shi, Y., and Blackwell, T.K. (2004). An extensive requirement for transcription factor IID-specific TAF-1 in Caenorhabditis elegans embryonic transcription. J Biol Chem *279*, 15339-15347.

Walthall, W.W. (1995). Repeating patterns of motoneurons in nematodes: the origin of segmentation? Exs *72*, 61-75.

Walthall, W.W., and Chalfie, M. (1988). Cell-cell interactions in the guidance of late-developing neurons in Caenorhabditis elegans. Science *239*, 643-645.

Walthall, W.W., Li, L., Plunkett, J.A., and Hsu, C.Y. (1993). Changing synaptic specificities in the nervous system of Caenorhabditis elegans: differentiation of the DD motoneurons. J Neurobiol *24*, 1589-1599.

Walthall, W.W., and Plunkett, J.A. (1995). Genetic transformation of the synaptic pattern of a motoneuron class in Caenorhabditis elegans. J Neurosci *15*, 1035-1043.

Wang, B.B., Muller-Immergluck, M.M., Austin, J., Robinson, N.T., Chisholm, A., and Kenyon, C. (1993). A homeotic gene cluster patterns the anteroposterior body axis of C. elegans. Cell *74*, 29-42.

Wang, J., Chen, P.J., Wang, G.J., and Keller, L. (2010). Chromosome size differences may affect meiosis and genome size. Science *329*, 293.

Wang, J.C., Walker, A., Blackwell, T.K., and Yamamoto, K.R. (2004). The Caenorhabditis elegans ortholog of TRAP240, CeTRAP240/let-19, selectively modulates gene expression and is essential for embryogenesis. J Biol Chem *279*, 29270-29277.

Wang, L., and Way, J.C. (1996). Activation of the mec-3 promoter in two classes of stereotyped lineages in Caenorhabditis elegans. Mech Dev *56*, 165-181.

Wang, L., and Way, J.C. (1996). Promoter sequences for the establishment of mec-3 expression in the nematode Caenorhabditis elegans. Mech Dev *56*, 183-196.

Wang, P., Zhao, J., and Corsi, A.K. (2006). Identification of novel target genes of CeTwist and CeE/DA. Dev Biol *293*, 486-498.

Wang, X., Greenberg, J.F., and Chamberlin, H.M. (2004). Evolution of regulatory elements producing a conserved gene expression pattern in Caenorhabditis. Evol Dev *6*, 237-245.

Waring, D.A., and Kenyon, C. (1990). Selective silencing of cell communication influences anteroposterior pattern formation in C. elegans. Cell *60*, 123-131.

Waring, D.A., and Kenyon, C. (1991). Regulation of cellular responsiveness to inductive signals in the developing C. elegans nervous system. Nature *350*, 712-715.

Waring, D.A., Wrischnik, L., and Kenyon, C. (1992). Cell signals allow the expression of a pre-existent neural pattern in C. elegans. Development *116*, 457-466.

Watari-Goshima, N., Ogura, K., Wolf, F.W., Goshima, Y., and Garriga, G. (2007). C. elegans VAB-8 and UNC-73 regulate the SAX-3 receptor to direct cell and growth-cone migrations. Nat Neurosci *10*, 169-176.

Waterston, R., Ainscough, R., Anderson, K., Berks, M., Blair, D., Connell, M., Cooper, J., Coulson, A., Craxton, M., Dear, S.*, et al.* (1993). The genome of the nematode Caenorhabditis elegans. Cold Spring Harb Symp Quant Biol *58*, 367-376.

Waterston, R.H., Thomson, J.N., and Brenner, S. (1980). Mutants with altered muscle structure of Caenorhabditis elegans. Dev Biol *77*, 271-302.

Way, J.C., and Chalfie, M. (1988). mec-3, a homeobox-containing gene that specifies differentiation of the touch receptor neurons in C. elegans. Cell *54*, 5-16.

Way, J.C., and Chalfie, M. (1989). The mec-3 gene of Caenorhabditis elegans requires its own product for maintained expression and is expressed in three neuronal cell types. Genes Dev *3*, 1823-1833.

Way, J.C., Run, J.Q., and Wang, A.Y. (1992). Regulation of anterior cell-specific mec-3 expression during asymmetric cell division in C. elegans. Dev Dyn *194*, 289-302.

Way, J.C., Wang, L., Run, J.Q., and Hung, M.S. (1994). Cell polarity and the mechanism of asymmetric cell division. Bioessays *16*, 925-931.

Way, J.C., Wang, L., Run, J.Q., and Wang, A. (1991). The mec-3 gene contains cis-acting elements mediating positive and negative regulation in cells produced by asymmetric cell division in Caenorhabditis elegans. Genes Dev *5*, 2199-2211.

Weinshenker, D., Garriga, G., and Thomas, J.H. (1995). Genetic and pharmacological analysis of neurotransmitters controlling egg laying in C. elegans. J Neurosci *15*, 6975-6985.

Wenick, A.S., and Hobert, O. (2004). Genomic cis-regulatory architecture and trans-acting regulators of a single interneuron-specific gene battery in C. elegans. Dev Cell *6*, 757-770.

Westmoreland, J.J., McEwen, J., Moore, B.A., Jin, Y., and Condie, B.G. (2001). Conserved function of Caenorhabditis elegans UNC-30 and mouse Pitx2 in controlling GABAergic neuron differentiation. J Neurosci *21*, 6810-6819.

Whangbo, J., and Kenyon, C. (1999). A Wnt signaling system that specifies two patterns of cell migration in C. elegans. Mol Cell *4*, 851-858.

White, J.G., Southgate, E., and Thomson, J.N. (1992). Mutations in the Caenorhabditis elegans unc-4 gene alter the synaptic input to ventral cord motor neurons. Nature *355*, 838-841.

White, J.Q., Nicholas, T.J., Gritton, J., Truong, L., Davidson, E.R., and Jorgensen, E.M. (2007). The sensory circuitry for sexual attraction in C. elegans males. Curr Biol *17*, 1847-1857.

Whitten, S.J., and Miller, M.A. (2007). The role of gap junctions in Caenorhabditis elegans oocyte maturation and fertilization. Dev Biol *301*, 432-446.

Wightman, B., Baran, R., and Garriga, G. (1997). Genes that guide growth cones along the C. elegans ventral nerve cord. Development *124*, 2571-2580.

Wightman, B., Clark, S.G., Taskar, A.M., Forrester, W.C., Maricq, A.V., Bargmann, C.I., and Garriga, G. (1996). The C. elegans gene vab-8 guides posteriorly directed axon outgrowth and cell migration. Development *122*, 671-682.

Wightman, B., Ebert, B., Carmean, N., Weber, K., and Clever, S. (2005). The C. elegans nuclear receptor gene fax-1 and homeobox gene unc-42 coordinate interneuron identity by regulating the expression of glutamate receptor subunits and other neuron-specific genes. Dev Biol *287*, 74-85.

Williams, B.D., Schrank, B., Huynh, C., Shownkeen, R., and Waterston, R.H. (1992). A genetic mapping system in Caenorhabditis elegans based on polymorphic sequence-tagged sites. Genetics *131*, 609-624.

Williams, S.N., Locke, C.J., Braden, A.L., Caldwell, K.A., and Caldwell, G.A. (2004). Epileptic-like convulsions associated with LIS-1 in the cytoskeletal control of neurotransmitter signaling in Caenorhabditis elegans. Hum Mol Genet *13*, 2043-2059.

Wilson, R., Ainscough, R., Anderson, K., Baynes, C., Berks, M., Bonfield, J., Burton, J., Connell, M., Copsey, T., Cooper, J.*, et al.* (1994). 2.2 Mb of contiguous nucleotide sequence from chromosome III of C. elegans. Nature *368*, 32-38.

Winnier, A.R., Meir, J.Y., Ross, J.M., Tavernarakis, N., Driscoll, M., Ishihara, T., Katsura, I., and Miller, D.M., 3rd (1999). UNC-4/UNC-37-dependent repression of motor neuron-specific genes controls synaptic choice in Caenorhabditis elegans. Genes Dev *13*, 2774-2786.

Witten, T.M., and Bonchev, D. (2007). Predicting aging/longevity-related genes in the nematode Caenorhabditis elegans. Chem Biodivers *4*, 2639-2655.

Wittenburg, N., Eimer, S., Lakowski, B., Rohrig, S., Rudolph, C., and Baumeister, R. (2000). Presenilin is required for proper morphology and function of neurons in C. elegans. Nature *406*, 306-309.

Wittmann, C., Bossinger, O., Goldstein, B., Fleischmann, M., Kohler, R., Brunschwig, K., Tobler, H., and Muller, F. (1997). The expression of the C. elegans labial-like Hox gene ceh-13 during early embryogenesis relies on cell fate and on anteroposterior cell polarity. Development *124*, 4193-4200.

Wolf, F.W., Hung, M.S., Wightman, B., Way, J., and Garriga, G. (1998). vab-8 is a key regulator of posteriorly directed migrations in C. elegans and encodes a novel protein with kinesin motor similarity. Neuron *20*, 655-666.

Wolinsky, E., and Way, J. (1990). The behavioral genetics of Caenorhabditis elegans. Behav Genet *20*, 169-189.

Wong, Y.F., Sheng, Q., Chung, J.W., Chan, J.K., and Chow, K.L. (2010). mab-31 and the TGF-beta pathway act in the ray lineage to pattern C. elegans male sensory rays. BMC Dev Biol *10*, 82.

Wood, W.B., and Edgar, L.G. (1994). Patterning in the C. elegans embryo. Trends Genet *10*, 49-54.

Woollard, A., and Hodgkin, J. (2000). The caenorhabditis elegans fate-determining gene mab-9 encodes a T-box protein required to pattern the posterior hindgut. Genes Dev *14*, 596-603.

Wrischnik, L.A., and Kenyon, C.J. (1997). The role of lin-22, a hairy/enhancer of split homolog, in patterning the peripheral nervous system of C. elegans. Development *124*, 2875-2888.

Xue, D., Finney, M., Ruvkun, G., and Chalfie, M. (1992). Regulation of the mec-3 gene by the C.elegans homeoproteins UNC-86 and MEC-3. Embo J *11*, 4969-4979.

Xue, D., Tu, Y., and Chalfie, M. (1993). Cooperative interactions between the Caenorhabditis elegans homeoproteins UNC-86 and MEC-3. Science *261*, 1324-1328.

Yamada, Y., and Ohshima, Y. (2003). Distribution and movement of Caenorhabditis elegans on a thermal gradient. J Exp Biol *206*, 2581-2593.

Yanagi, K., Mizuno, T., Tsuyama, T., Tada, S., Iida, Y., Sugimoto, A., Eki, T., Enomoto, T., and Hanaoka, F. (2005). Caenorhabditis elegans geminin homologue participates in cell cycle regulation and germ line development. J Biol Chem *280*, 19689-19694.

Yanai, I., Baugh, L.R., Smith, J.J., Roehrig, C., Shen-Orr, S.S., Claggett, J.M., Hill, A.A., Slonim, D.K., and Hunter, C.P. (2008). Pairing of competitive and topologically distinct regulatory modules enhances patterned gene expression. Mol Syst Biol *4*, 163.

Yandell, M.D., Edgar, L.G., and Wood, W.B. (1994). Trimethylpsoralen induces small deletion mutations in Caenorhabditis elegans. Proc Natl Acad Sci U S A *91*, 1381-1385.

Yang, L., Sym, M., and Kenyon, C. (2005). The roles of two C. elegans HOX co-factor orthologs in cell migration and vulva development. Development *132*, 1413-1428.

Yang, Y., Sun, Y., Luo, X., Zhang, Y., Chen, Y., Tian, E., Lints, R., and Zhang, H. (2007). Polycomb-like genes are necessary for specification of dopaminergic and serotonergic neurons in Caenorhabditis elegans. Proc Natl Acad Sci U S A *104*, 852-857.

Yanowitz, J.L. (2008). Genome integrity is regulated by the Caenorhabditis elegans Rad51D homolog rfs-1. Genetics *179*, 249-262.

Yanowitz, J.L., Shakir, M.A., Hedgecock, E., Hutter, H., Fire, A.Z., and Lundquist, E.A. (2004). UNC-39, the C. elegans homolog of the human myotonic dystrophy-associated homeodomain protein Six5, regulates cell motility and differentiation. Dev Biol *272*, 389-402.

Yi, B., Bumbarger, D., and Sommer, R.J. (2009). Genetic evidence for pax-3 function in myogenesis in the nematode Pristionchus pacificus. Evol Dev *11*, 669-679.

Yi, B., and Sommer, R.J. (2007). The pax-3 gene is involved in vulva formation in Pristionchus pacificus and is a target of the Hox gene lin-39. Development *134*, 3111-3119.

Yi, W., Ross, J.M., and Zarkower, D. (2000). Mab-3 is a direct tra-1 target gene regulating diverse aspects of C. elegans male sexual development and behavior. Development *127*, 4469-4480.

Yin, X., Gower, N.J., Baylis, H.A., and Strange, K. (2004). Inositol 1,4,5-trisphosphate signaling regulates rhythmic contractile activity of myoepithelial sheath cells in Caenorhabditis elegans. Mol Biol Cell *15*, 3938-3949.

Yochem, J., Sundaram, M., and Bucher, E.A. (2000). Mosaic analysis in Caenorhabditis elegans. Methods Mol Biol *135*, 447-462.

Yoda, A., Kouike, H., Okano, H., and Sawa, H. (2005). Components of the transcriptional Mediator complex are required for asymmetric cell division in C. elegans. Development *132*, 1885-1893.

Yoshimura, S., Murray, J.I., Lu, Y., Waterston, R.H., and Shaham, S. (2008). mls-2 and vab-3 Control glia development, hlh-17/Olig expression and glia-dependent neurite extension in C. elegans. Development *135*, 2263-2275.

Yu, H., Prétôt, R.F., Bürglin, T.R., and Sternberg, P.W. (2003). Distinct roles of transcription factors EGL-46 and DAF-19 in specifying the functionality of a polycystin-expressing sensory neuron necessary for C. elegans male vulva location behavior. Development *130*, 5217-5227.

Yu, H., Seah, A., and Sternberg, P.W. (2010). Re-programming of C. elegans male epidermal precursor fates by Wnt, Hox, and LIN-12/Notch activities. Dev Biol *345*, 1-11.

Yuan, J.Y., Finney, M., Tsung, N., and Horvitz, H.R. (1991). Tc4, a Caenorhabditis elegans transposable element with an unusual fold-back structure. Proc Natl Acad Sci U S A *88*, 3334-3338.

Yuan, J.Y., and Horvitz, H.R. (1990). The Caenorhabditis elegans genes ced-3 and ced-4 act cell autonomously to cause programmed cell death. Dev Biol *138*, 33-41.

Zahreddine, H., Zhang, H., Diogon, M., Nagamatsu, Y., and Labouesse, M. (2010). CRT-1/calreticulin and the E3 ligase EEL-1/HUWE1 control hemidesmosome maturation in C. elegans development. Curr Biol *20*, 322-327.

Zallen, J.A., Kirch, S.A., and Bargmann, C.I. (1999). Genes required for axon pathfinding and extension in the C. elegans nerve ring. Development *126*, 3679-3692.

Zariwala, H.A., Miller, A.C., Faumont, S., and Lockery, S.R. (2003). Step response analysis of thermotaxis in Caenorhabditis elegans. J Neurosci *23*, 4369-4377.

Zarkower, D. (2006). Somatic sex determination. WormBook, 1-12.

Zarkower, D., De Bono, M., Aronoff, R., and Hodgkin, J. (1994). Regulatory rearrangements and smg-sensitive alleles of the C. elegans sex-determining gene tra-1. Dev Genet *15*, 240-250.

Zetka, M.C., and Rose, A.M. (1992). The meiotic behavior of an inversion in Caenorhabditis elegans. Genetics *131*, 321-332.

Zhang, G., Sleiman, S.F., Tseng, R.J., Rajakumar, V., Wang, X., and Chamberlin, H.M. (2005). Alteration of the DNA binding domain disrupts distinct functions of the C. elegans Pax protein EGL-38. Mech Dev *122*, 887-899.

Zhang, H., Azevedo, R.B., Lints, R., Doyle, C., Teng, Y., Haber, D., and Emmons, S.W. (2003). Global regulation of Hox gene expression in C. elegans by a SAM domain protein. Dev Cell *4*, 903-915.

Zhang, H., and Emmons, S.W. (2000). A C. elegans mediator protein confers regulatory selectivity on lineage-specific expression of a transcription factor gene. Genes Dev *14*, 2161-2172.

Zhang, H., and Emmons, S.W. (2001). The novel C. elegans gene sop-3 modulates Wnt signaling to regulate Hox gene expression. Development *128*, 767-777.

Zhang, H., and Emmons, S.W. (2009). Regulation of the Caenorhabditis elegans posterior Hox gene egl-5 by microRNA and the polycomb-like gene sop-2. Dev Dyn *238*, 595-603.

Zhang, M., Chung, S.H., Fang-Yen, C., Craig, C., Kerr, R.A., Suzuki, H., Samuel, A.D., Mazur, E., and Schafer, W.R. (2008). A self-regulating feed-forward circuit controlling C. elegans egg-laying behavior. Curr Biol *18*, 1445-1455.

Zhang, M., Poplawski, M., Yen, K., Cheng, H., Bloss, E., Zhu, X., Patel, H., and Mobbs, C.V. (2009). Role of CBP and SATB-1 in aging, dietary restriction, and insulin-like signaling. PLoS Biol *7*, e1000245.

Zhang, S., Sokolchik, I., Blanco, G., and Sze, J.Y. (2004). Caenorhabditis elegans TRPV ion channel regulates 5HT biosynthesis in chemosensory neurons. Development *131*, 1629-1638.

Zhang, T., Sun, Y., Tian, E., Deng, H., Zhang, Y., Luo, X., Cai, Q., Wang, H., Chai, J., and Zhang, H. (2006). RNA-binding proteins SOP-2 and SOR-1 form a novel PcG-like complex in C. elegans. Development *133*, 1023-1033.

Zhang, Y., and Chalfie, M. (2002). MTD-1, a touch-cell-specific membrane protein with a subtle effect on touch sensitivity. Mech Dev *119*, 3-7.

Zhang, Y., and Emmons, S.W. (1995). Specification of sense-organ identity by a Caenorhabditis elegans Pax-6 homologue. Nature *377*, 55-59.

Zhang, Y., Ferreira, H.B., Greenstein, D., Chisholm, A., and Emmons, S.W. (1998). Regulated nuclear entry of the C. elegans Pax-6 transcription factor. Mech Dev *78*, 179-187.

Zhang, Y., Ma, C., Delohery, T., Nasipak, B., Foat, B.C., Bounoutas, A., Bussemaker, H.J., Kim, S.K., and Chalfie, M. (2002). Identification of genes expressed in C. elegans touch receptor neurons. Nature *418*, 331-335.

Zhang, Y., and Maduzia, L.L. (2010). Mutations in Caenorhabditis elegans eIF2beta permit translation initiation from non-AUG start codons. Genetics *185*, 141-152.

Zhao, B., Khare, P., Feldman, L., and Dent, J.A. (2003). Reversal frequency in Caenorhabditis elegans represents an integrated response to the state of the animal and its environment. J Neurosci *23*, 5319-5328.

Zhao, H., and Nonet, M.L. (2000). A retrograde signal is involved in activity-dependent remodeling at a C. elegans neuromuscular junction. Development *127*, 1253-1266.

Zhao, Z., Boyle, T.J., Liu, Z., Murray, J.I., Wood, W.B., and Waterston, R.H. (2010). A negative regulatory loop between microRNA and Hox gene controls posterior identities in Caenorhabditis elegans. PLoS Genet *6*.

Zhao, Z., Flibotte, S., Murray, J.I., Blick, D., Boyle, T.J., Gupta, B., Moerman, D.G., and Waterston, R.H. (2010). New tools for investigating the comparative biology of Caenorhabditis briggsae and C. elegans. Genetics *184*, 853-863.

Zheng, X., Chung, S., Tanabe, T., and Sze, J.Y. (2005). Cell-type specific regulation of serotonergic identity by the C. elegans LIM-homeodomain factor LIM-4. Dev Biol *286*, 618-628.

Zhou, H.M., and Walthall, W.W. (1998). UNC-55, an orphan nuclear hormone receptor, orchestrates synaptic specificity among two classes of motor neurons in Caenorhabditis elegans. J Neurosci *18*, 10438-10444.

Ziel, J.W., Hagedorn, E.J., Audhya, A., and Sherwood, D.R. (2009). UNC-6 (netrin) orients the invasive membrane of the anchor cell in C. elegans. Nat Cell Biol *11*, 183-189.

Zinovyeva, A.Y., Yamamoto, Y., Sawa, H., and Forrester, W.C. (2008). Complex network of Wnt signaling regulates neuronal migrations during Caenorhabditis elegans development. Genetics *179*, 1357-1371.

Zwaal, R.R., Broeks, A., van Meurs, J., Groenen, J.T., and Plasterk, R.H. (1993). Target-selected gene inactivation in Caenorhabditis elegans by using a frozen transposon insertion mutant bank. Proc Natl Acad Sci U S A *90*, 7431-7435.
